# Recurrent *CTNNB1* mutations in craniofacial osteomas

**DOI:** 10.1038/s41379-021-00956-x

**Published:** 2021-11-01

**Authors:** Daniel Baumhoer, Ruth Berthold, Ilka Isfort, Lorena Heinst, Baptiste Ameline, Inga Grünewald, Florian M. Thieringer, Claudia Rudack, Eva Wardelmann, Volker Vieth, Jan Sperveslage, Marcel Trautmann, Wolfgang Hartmann

**Affiliations:** 1 Bone Tumor Reference Centre at the Institute of Pathology, University Hospital Basel and University of Basel, Schoenbeinstrasse 40, 4031 Basel, Switzerland; 2 Division of Translational Pathology, Gerhard-Domagk-Institute of Pathology, University Hospital Münster, Albert-Schweitzer-Campus 1, 48149 Münster, Germany; 3 Gerhard-Domagk-Institute of Pathology, University Hospital Münster, Albert-Schweitzer-Campus 1, 48149 Münster, Germany; 4 Oral and Cranio-Maxillofacial Surgery, University Hospital Basel, Spitalstraße 21, 4031 Basel, Switzerland; 5 Medical Additive Manufacturing Research Group (Swiss MAM), Department of Biomedical Engineering, University of Basel, Gewerbestrasse 14, 4123 Allschwil, Switzerland; 6 Department of Otorhinolaryngology-Head and Neck Surgery, University Hospital Münster, Albert-Schweitzer-Campus 1, 48149 Münster, Germany; 7 Department of Radiology, Klinikum Ibbenbüren, Grosse Strasse 41, 49477 Ibbenbüren, Germany

**Keywords:** Mutation, Gene expression

## Abstract

Osteoma is a benign bone forming tumor predominantly arising on the surface of craniofacial bones. While the vast majority of osteomas develops sporadically, a small subset of cases is associated with Gardner syndrome, a phenotypic variant of familial adenomatous polyposis caused by mutations in the *APC* gene resulting in aberrant activation of WNT/β-catenin signaling. In a sequencing analysis on a cohort of sporadic, non-syndromal osteomas, we identified hotspot mutations in the *CTNNB1* gene (encoding β-catenin) in 22 of 36 cases (61.1%), harbouring allelic frequencies ranging from 0.04 to 0.53, with the known S45P variant representing the most frequent alteration. Based on NanoString multiplex expression profiling performed in a subset of cases, *CTNNB1*-mutated osteomas segregated in a defined “*WNT*-cluster”, substantiating functionality of *CTNNB1* mutations which are associated with β-catenin stabilization. Our findings for the first time convincingly show that osteomas represent genetically-driven neoplasms and provide evidence that aberrant WNT/β-catenin signaling plays a fundamental role in their pathogenesis, in line with the well-known function of WNT/β-catenin in osteogenesis. Our study contributes to a better understanding of the molecular pathogenesis underlying osteoma development and establishes a helpful diagnostic molecular marker for morphologically challenging cases.

## Introduction

Osteoma is a benign bone forming tumor arising predominantly on the surface of bones formed by membranous ossification, i.e., the craniofacial, and jaw bones [1]. While their prevalence has been reported to reach 6.4% in selected series [2], the true incidence of osteomas is unknown, and it seems likely that many lesions remain clinically undetected due to the lack of specific symptoms. In advanced cases, osteomas can lead to deformation of the affected bone and compression of adjacent structures, including obstruction of the paranasal sinuses, associated with headache and paraesthesia [1]. Histologically, osteomas are predominantly composed of mature lamellar bone and can be divided into compact and spongious subtypes which most frequently show low cellularity and inconspicuous osteoblastic activity. However, in sinuorbital tumors, prominent osteoblastic and osteoclastic activity may be present and mimic features of osteoblastoma [3, 4]. Different hypotheses on the aetiology of osteomas have been proposed, including trauma, secondary changes of paranasal polyps, metaplasia, and dysontogenic or hereditary factors, respectively [1]. Osteoma is rarely associated with Gardner syndrome (representing a phenotypic variant of familial adenomatous polyposis (FAP)), characterized by *APC* mutations and aberrant activation of WNT/β-catenin signaling [5, 6]. The central key mediator of the canonical WNT/β-catenin signaling pathway is β-catenin, which in the pathway´s inactive state is degraded through a multiprotein complex including axis inhibition protein (AXIN), adenomatous polyposis coli (APC), casein-kinase-1 (CK1) and glycogen synthase kinase-3beta (GSK-3β) via sequential phosphorylation, ubiquitination, and proteasomal degradation. Binding of WNT ligands to frizzled receptors and co-receptors of the LRP family leads to inactivation of the ubiquitination/degradation function of the multiprotein destruction complex and nuclear accumulation of β-catenin, where it interacts with transcription factors of the T-cell factor and lymphoid enhancer factor family and induces transcriptional programs [7]. Given well-known examples of neoplasms arising in the setting of FAP (e.g., desmoid fibromatosis, hepatoblastoma) that occur independently from *APC* mutations in the sporadic setting and the established role of WNT/β-catenin signaling in osteogenesis (reviewed in [8, 9]), we set out to analyze a cohort of craniofacial osteomas for the presence of *CTNNB1* gene mutations.

## Materials and methods

### Patients and samples

Cases of interest with sporadic osteoma were identified by searching the histopathology archives at the Basel Bone Tumor Reference Centre (Basel, Switzerland) and the Gerhard-Domagk-Institute of Pathology (Münster, Germany). 19 tumors were classified as compact, 14 as spongious, and three as osteoblastoma-like osteomas. Thorough investigation of the clinical history and radiological findings was performed to assess a syndromal predisposition with no evidence found in the selected cohort. The study was approved by the Ethical committees in Münster (reference 2020-886fS) and Basel (reference 274/12).

### Next-generation sequencing (NGS)

Two customized NGS gene panels were applied to sequence the complete exonic region of human *CTNNB1* (encoding β-catenin). The first sequencing approach was conducted using a hybrid capture-based hot spot panel (Illumina, covering 0.013 Mb, selected exons of 19 genes), including exon 3 of the *CTNNB1* gene (RefSeq: NM_001098210; Ensembl transcript ID: ENST00000396183; UniProt/Swiss-Prot: P35222). All *CTNNB1* exon 3 wild type samples were additionally analyzed employing a second NGS gene panel (Qiagen, covering 0.0638 Mb, 27 genes) to assess exons 2-15 of the *CTNNB1 gene*. Target enrichment was processed by means of the Nextera DNA Flex Pre-Enrichment Library Prep and Enrichment Reagents kit (Illumina) and the GeneRead DNAseq Panel PCR V2 kit (Qiagen). All purification and size selection steps were performed employing Agencourt AMPure XP magnetic beads (Beckman Coulter). IDT for Nextera DNA Unique Dual Indexes (Illumina) and NEXTflex-96 DNA barcodes (Bioo Scientific) were used. Sequencing was performed on the Illumina MiniSeq system (MiniSeq High Output Reagent Kit; 300-cycles). The Quantitative Multiplex FFPE Reference Standard (Horizon Discovery, ref. no. HD200) was applied as isogenic quality control (11 somatic mutations verified at 0.8–24.5% allelic frequency in genomic DNA) for routine performance monitoring and evaluation of NGS workflow integrity (pre-analytical DNA extraction, NGS workflow and post-analytical bioinformatics). NGS data analysis was performed by means of the CLC Genomics Workbench 20 software (CLC bio, Qiagen).

### Immunohistochemistry (IHC)

Immunohistochemical stainings were performed on cases with acceptable preservation of morphology after decalcification using a β-catenin antibody (monoclonal mouse, clone 14, CellMarque, Rocklin, CA, USA) on a BenchMark ULTRA Autostainer (VENTANA/Roche, Basel, Switzerland) using 3 μm tissue sections. In brief, the staining procedure included heat-induced epitope retrieval pretreatment employing CC1 buffer (95 °C; 24 min) followed by incubation with the primary antibody for 32 min and signal detection using the OptiView DAB IHC Detection Kit (VENTANA/Roche, Basel, Switzerland) according to the manufacturer’s instructions. Positive and negative control stainings using an appropriate IgG subtype (DCS) were included. Due to the limited quality of the tissue, nuclear immunoreactivity was assessed as negative or positive, independent from the staining intensity.

### Multiplex gene expression profiling

Formalin‐fixed and paraffin‐embedded (FFPE)-derived RNA samples were analyzed employing the NanoString nCounter FLEX gene expression analysis system with DX enablement according to the manufacturer´s instructions. This system was chosen with respect to the limited quality of decalcified tissue as it does not require enzymatic reverse transcription nor amplification, but yields information on the relative abundance of each mRNA transcript of interest through multiplexed hybridization and digital readout of fluorescent barcoded probes hybridized to each transcript [10]. The human PanCancer Pathways CodeSet (XT-CSO-PATH1-1) was used, covering 770 genes including 40 internal reference controls/housekeeping genes from 13 essential cancer hallmark-associated canonical pathways (MAPK, STAT, PI3K, RAS, Cell Cycle, Apoptosis, Hedgehog, WNT, Notch, DNA Damage Control, Transcriptional Regulation, Chromatin Modification, and TGF-β). The CodeSet containing capture and reporter probes was hybridized to 180 ng of total RNA for 18 h at 65 °C. Hybridized samples were loaded into the nCounter Prep Station for post‐hybridization processing. Expression of target mRNA was assessed with the nCounter Digital Analyzer. The NanoString nCounter nSolver software (version 4.0), with an additional NanoString Advanced Analysis Module (version 2.0.134) was used to perform quality control assessment, normalization, differential expression analysis, and pathway analyses. Data quality was assessed by default parameters according to the NanoString gene expression data analysis guidelines. No background subtraction or thresholding parameters were selected. The geometric means were used to compute normalization factors for the mRNA content of 6 internal positive control probes and 40 internal housekeeping genes. In the fold-change estimation settings, the ratio building option was selected and samples were partitioned based on their respective *CTNNB1* mutational status. Housekeeping gene normalization was computed with an open-source R statistical package using the geNORM algorithm [11, 12]. Normalized log2 transformed mRNA counts were generated according to the gene expression normalization factor based on the geometric mean of the most stable housekeeping genes and *Z* score transformation gene expression values were used for subsequent data analysis. P-value adjustments were performed using the Benjamini–Yekutieli method [13]. Expression data is shown in Supplementary Table S1.

## Results

**Fig. 1:**
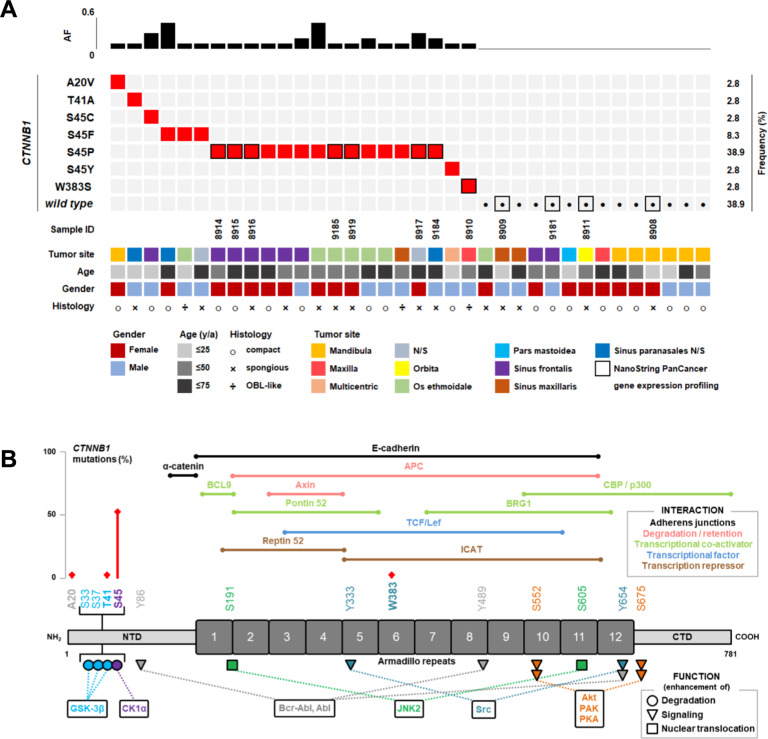
Results of the mutational analysis of the *CTNNB1* gene in 36 osteomas. **A** Clustered mutational profile of osteomas (*n* = 36). Alterations in *CTNNB1* (rows) are indicated for each sample (columns). Clinicopathological information is summarized according to the legend. Cases with indicated sample IDs (framed squares) were included in the NanoString analysis. AF allelic frequency. **B** Schematic overview of β-catenin indicating relevant protein domains, phosphorylation sites, interaction partners and associated cellular functions. Detected mutations in the N-terminal domain and the Armadillo repeat region are indicated in red along with their frequency. NTD N-terminal domain, CTD C-terminal domain.

**Fig. 2:**
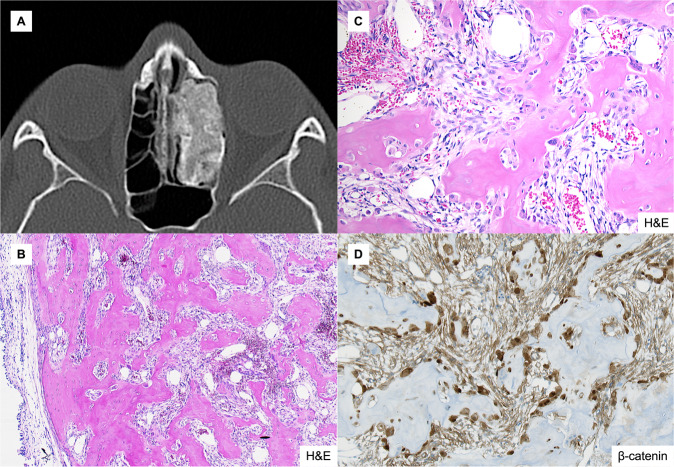
Radiological and histological features of an example of osteoblastoma-like osteoma. **A** CT scan and (**B**, **C**) H&E stained aspects of an osteoma with osteoblastoma-like features arising in the ethmoidal sinus involving the nasal cavity. Conspicuous osteoblastic and osteoclastic activity is observed against the background of a well-vascularized and moderately cellular and fibrous stroma in the marrow spaces (**B**, original magnification 10x; **C**, original magnification 20x). **D** Osteoid matrix-rimming osteoblasts display nuclear β-catenin positivity (original magnification 20x).

**Fig. 3:**
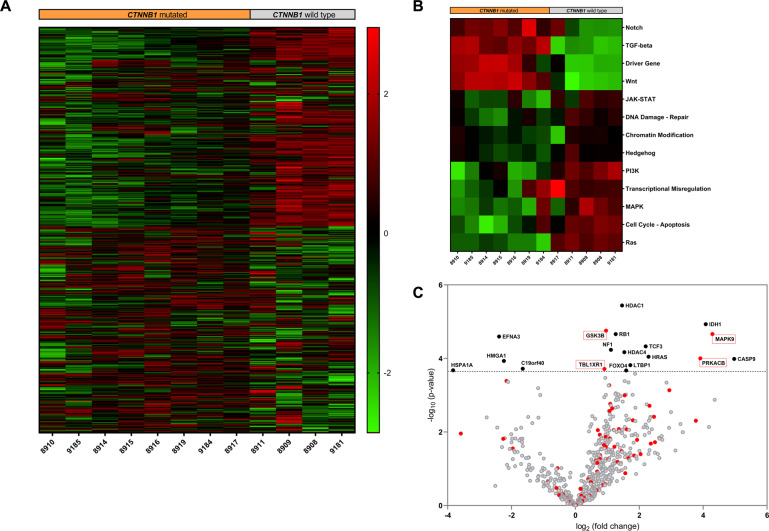
NanoString multiplex gene expression profiling employing the human PanCancer Pathways CodeSet covering 770 genes. **A** Exploratory heatmap of the normalized expression data generated via unsupervised clustering clearly distinguishes *CTNNB1* mutated and *CTNNB1* wild type osteomas according to their mRNA expression profile (red indicates high expression; green indicates low expression). **B** Heatmap of pathway scores presenting an overview of 13 essential hallmark-associated canonical pathways (MAPK, STAT, PI3K, RAS, Cell Cycle, Apoptosis, Hedgehog, WNT, Notch, DNA Damage Control, Transcriptional Regulation, Chromatin Modification, and TGF-β), indicating that *CTNNB1* mutated osteomas exhibit, among others, similar WNT/β-catenin pathway activation profiles, as compared to the *CTNNB1* wild type group (red indicates high pathway scores; green indicates low scores). **C** Volcano plot displaying each gene’s –log10 (*p* value) and log2 fold change as calculated from the differential expression profile in the set of *CTNNB1* mutated osteomas vs. baseline expression in the set of *CTNNB1* wild type cases. Statistically significantly regulated genes are shown in the upper part of the plot above the horizontal line, differentially expressed genes fall to either side. Genes related to WNT/β-catenin pathway activation profile are highlighted in red.

We analyzed 36 samples from 36 individuals with histologically confirmed, sporadic craniofacial osteoma. The patient cohort included 19 females (52.8%) and 17 males (47.2%) ranging from 12 to 71 years of age (median 38.5 years). Twenty-five lesions (69.4%) involved the paranasal sinuses, nine (25%) were located in the jaws, and two (5.6%) involved the orbital and mastoid processes, respectively. Radiologically, all cases presented with characteristic imaging features, i.e., well-demarcated round, dense, well-defined ivory-like tumors attached to the underlying bone without cortical invasion. Thorough assessment of the clinical files revealed no evidence of multiple osteomas or accompanying skin lesions, congenital hypertrophy of the retinal pigmented epithelium, desmoid tumors or polyposis coli as indicators of a syndromal association. In 19 out of 36 cases (52.8%), classic (functionally activating) mutations affecting S45 in exon 3 of the *CTNNB1* gene were detected, with S45P representing the most frequent alteration (Fig. 1A, B). One tumor (2.8%) displayed the well-known T41A *CTNNB1* gene mutation. Two further osteomas were detected to carry rarer *CTNNB1* mutations: (i) W383S, lying within Amardillo repeat 6 of β-catenin (Fig. 1B); though W383S has not been functionally described yet, the related W383R alteration has been shown to confer increased function to β-catenin as indicated by enhanced transcriptional activity compared to wild type β-catenin in cultured cells [14], and (ii) an A20V mutation that has been described before in anaplastic thyroid carcinoma where it was found not to be associated with nuclear accumulation of β-catenin, thus being of questionable functional relevance [15]. No significant difference regarding the *CTNNB1* mutation frequency was detected between the histological subtypes of osteomas (Fig. 1A). As a significant number of cases was incidentally detected and aggressive acid decalcification (accompanied by protein denaturation) is regularly employed in tissue work-up of bone-containing specimens derived from routine surgery of the paranasal sinuses submitted without clinically suspected atypia or malignancy, immunohistochemical characterization was not informative, i.e., technically unfeasible, in a larger subset of the lesions (15 out of 36 cases). In 14 out of 16 *CTNNB1*-mutated cases, nuclear β-catenin immunoreactivity was detected, however, considerable variability with regard to the percentage of stained nuclei and staining intensity was observed. In selected cases, β-catenin was consistently detected in the nuclei of osteoblasts rimming lesional bone (Fig. 2). Contrarily, in 7 out of 7 *CTNNB1*-wildtype cases, no nuclear β-catenin staining was discernible. Given the methodical limitation of immunohistochemistry in acid-decalcified tissue, prediction of *CTNNB1* mutational status through evaluation of nuclear β-catenin accumulation appeared not sufficiently reliable. In order to get deeper insights into the biological differences of osteomas according to the *CTNNB1* mutational status, we performed multiplex gene expression profiling employing the NanoString nCounter system. This system was used as it does not require enzymatic reverse transcription nor amplification steps, but yields information on the relative abundance of each mRNA transcript of interest through multiplexed hybridization. After RNA extraction and quality control, based on an mRNA expression analysis covering 770 genes from 13 essential cancer hallmark-associated canonical pathways, eight *CTNNB1* mutated and four *CTNNB1* wild type osteomas were comparatively analyzed. Though the extractable information on individual genes is naturally limited, this approach provides evidence of distinct gene sets being expressed and biological pathways being activated in osteomas with and without *CTNNB1* mutation, respectively (Fig. 3A, B). Pathway scores derived from this approach indicated an activation of the Notch, TGF-beta, and WNT signaling pathways in the *CTNNB1* mutated group as compared to the *CTNNB1* wild type group (Fig. 3B). The distribution of mRNA expression (fold change) of genes derived from the comparison of the group of *CTNNB1* mutated to the group of *CTNNB1* wild type cases is depicted in Fig. 3C. The relative expression of WNT/β-catenin-associated genes is summarized in Supplementary Fig. 1.

## Discussion

The aetiology of osteomas is an ongoing matter of debate. Some authors favour trauma, secondary changes of paranasal polyps, or inflammatory processes as the initiating trigger, therefore implying a reactive character for at least a subset of lesions fulfilling the diagnostic criteria of osteoma [1]. On the contrary, rare cases of (multiple) osteomas arising in a context of genetic syndromes, particularly FAP, point to a true neoplastic origin of these lesions [5, 6]. The current WHO classification of soft tissue and bone tumors classifies them as benign tumors, stating that the pathogenesis of sporadic osteomas is unknown [16]. In this focused analysis of a cohort of sporadic, non-syndromal osteomas, we have identified hotspot mutations in the *CTNNB1* gene (encoding β-catenin) in 22 of 36 cases (61.1%). The β-catenin T41 and S45 mutations primarily detected here represent major regulatory sites for interaction with GSK-3β (phosphorylation at T41, S33 and S37) and CK1 (S45). These variants are known to prohibit β-catenin ubiquitination and proteasomal degradation, eventually leading to dysregulated WNT/β-catenin signaling [17–19].

According to the clinical data available, none of the patients (including those with a *CTNNB1* wild type osteoma), had multiple osteomas or stigmata implying a syndromal association and thus potential involvement of the *APC* gene. This is in line with the results of our NanoString mRNA expression approach, providing evidence of distinct gene sets being expressed in osteomas with and without *CTNNB1* mutation, respectively. While in *CTNNB1* mutated osteomas a strong WNT pathway activation was discernible, *CTNNB1* wild type osteomas did not show a relevant WNT signature activation. This makes alternative hits leading to WNT activation (including *APC* mutations) unlikely, given the findings of Crago and colleagues in desmoid-type fibromatosis in which *CTNNB1* mutated and wild type cases were indiscernible in unsupervised clustering of U133A-derived gene expression profiles; in their analysis, the *CTNNB1* wild type group of desmoid-type fibromatoses was shown to comprise cases with *APC* losses [20]. Alternatively, a mechanism of “selective apoptosis” of β-catenin mutated cells, similar to the supposed mechanism of “vanishing” GNAS mutations in long-standing fibrous dysplasia might be postulated to explain the *CTNNB1* wild type status of a subset of osteomas. Eventually, a number of lesions that appear clinically and histologically similar to osteomas may indeed represent reactive compact new bone formations resulting from a possible inflammatory stimulus. Assessing the anatomic distribution of *CTNNB1* wild type cases, it is noticeable that mandibular lesions are overrepresented in this group and that the only detectable *CTNNB1* variant was the A20V mutation lacking evidence of functional relevance [15]. This might point to mandibular lesions being biologically different, but the size of the cohort is too limited for statistical analyses. Nonetheless, the NanoString mRNA expression analysis convincingly documenting distinct expression profiles according to *CTNNB1* status can be regarded as a strong argument for the assumption that *CTNNB1* wild type and *CTNNB1* mutated cases are biologically distinct. Independent from localization, morphological re-evaluation of *CTNNB1* wild type and *CTNNB1* mutated cases revealed no significantly different histological patterns.

The frequency of classic *CTNNB1* mutations in sporadic osteomas opposed to a well-known syndromal FAP-association of rare cases is reminiscent to other diseases rarely arising in the context of FAP, i.e., desmoid fibromatosis and hepatoblastoma [21, 22]. Desmoid fibromatosis has been reported to carry *CTNNB1* mutations in up to 89% of sporadic cases [20, 23–27], while *APC* mutations have only rarely been detected in the sporadic context [20, 28]. In hepatoblastoma, *CTNNB1* mutations have been found in up to 67% of cases, all affecting exon 3 which encodes the degradation targeting box of β-catenin, resulting in nuclear β-catenin accumulation [29–31]. The latter observation is of particular interest as WNT/β-catenin signaling is known to play a pivotal role in developmental hepatoblast proliferation, liver organogenesis and regeneration (reviewed in [32]). A comparably essential role of canonical WNT/β-catenin signaling is well established also in osteogenesis (reviewed in [8, 9]). Clinical evidence is derived from the finding that loss of function mutations in the *LRP5* gene, encoding a coreceptor of canonical WNT signaling, is linked to a decrease of bone density, associated with defects in osteoblast differentiation and proliferation [33]. Consistently, *Lrp5*^−/−^ mice display a reduction in bone mass and a disruption of osteoblast proliferation and bone matrix deposition [34]. On the contrary, gain of function mutations in *LRP5* are associated with an increase in bone mass (reviewed in [35]), and mice expressing the *LRP5* G171V mutation in osteoblasts display increased bone density and elevated numbers of active osteoblasts [36]. Mice deficient in *Axin2*, a negative regulator of canonical WNT signaling, exhibit craniosynostosis due to an increased proliferation of osteoblastic progenitors in the skull sutures [37], and haploid deficiency of β-catenin was shown to alleviate craniofacial bone defects caused by *Axin2* deficiency [38]. Elaborate experiments examining the conditional inactivation of β-catenin in skeletal progenitors revealed that β-catenin activity is crucial for the differentiation of mature osteoblasts and thus for both endochondral and membranous ossification, the latter being particularly important for the development of the craniofacial bones [39, 40].

Our finding of recurrent *CTNNB1* mutations in osteomas contributes to the concept of an outstanding role of WNT/β-catenin in bone biology and disease. We show osteoma to represent the first bone tumor associated with the relatively “simple” and recurrent genetic background of single nucleotide variants in the *CTNNB1* gene and thus provide evidence for a genetically defined, true neoplasm. Beyond this conceptual aspect, our findings establish *CTNNB1* mutations as a helpful diagnostic marker which may be of use in morphologically challenging cases. Given the limitations of immunohistochemistry in larger subsets of such lesions in which, however, tissue quality after decalcification may still be sufficient for highly sensitive NGS approaches, molecular confirmation of *CTNNB1* mutations appears to be the most suitable tool to be employed.

## Supplementary information

Supplemental Figures and Tables

## Data Availability

The datasets used and/or analyzed during the current study are available from the corresponding author on reasonable request. All original mRNA expression data are available (Supplementary Table S1).
