# Supplementary material for: Recurrent CTNNB1 mutations in craniofacial osteomas
Source: Mod Pathol. 2021 Nov 1;35(4):489–94. doi: 10.1038/s41379-021-00956-x (PMC8964415; doi:10.1038/s41379-021-00956-x)
Supplement: Supplementary file 1 — Supplemental Figures and Tables [file 41379_2021_956_MOESM1_ESM.pdf]

Daniel Baumhoer *et al.*: **Recurrent *CTNNB1* mutations in craniofacial osteomas**

Legends to Supplemental Figures & Tables

Supplemental Figure S1: Focused heatmap of normalized WNT/β-catenin pathway expression data generated via unsupervised clustering to compare *CTNNB1* mutated and *CTNNB1* wild type osteomas according to their mRNA expression profile (red indicates high expression; green indicates low expression).

Supplemental Table S1: Original data derived from the NanoString multiplex gene expression profiling employing the human PanCancer Pathways CodeSet covering 770 genes.

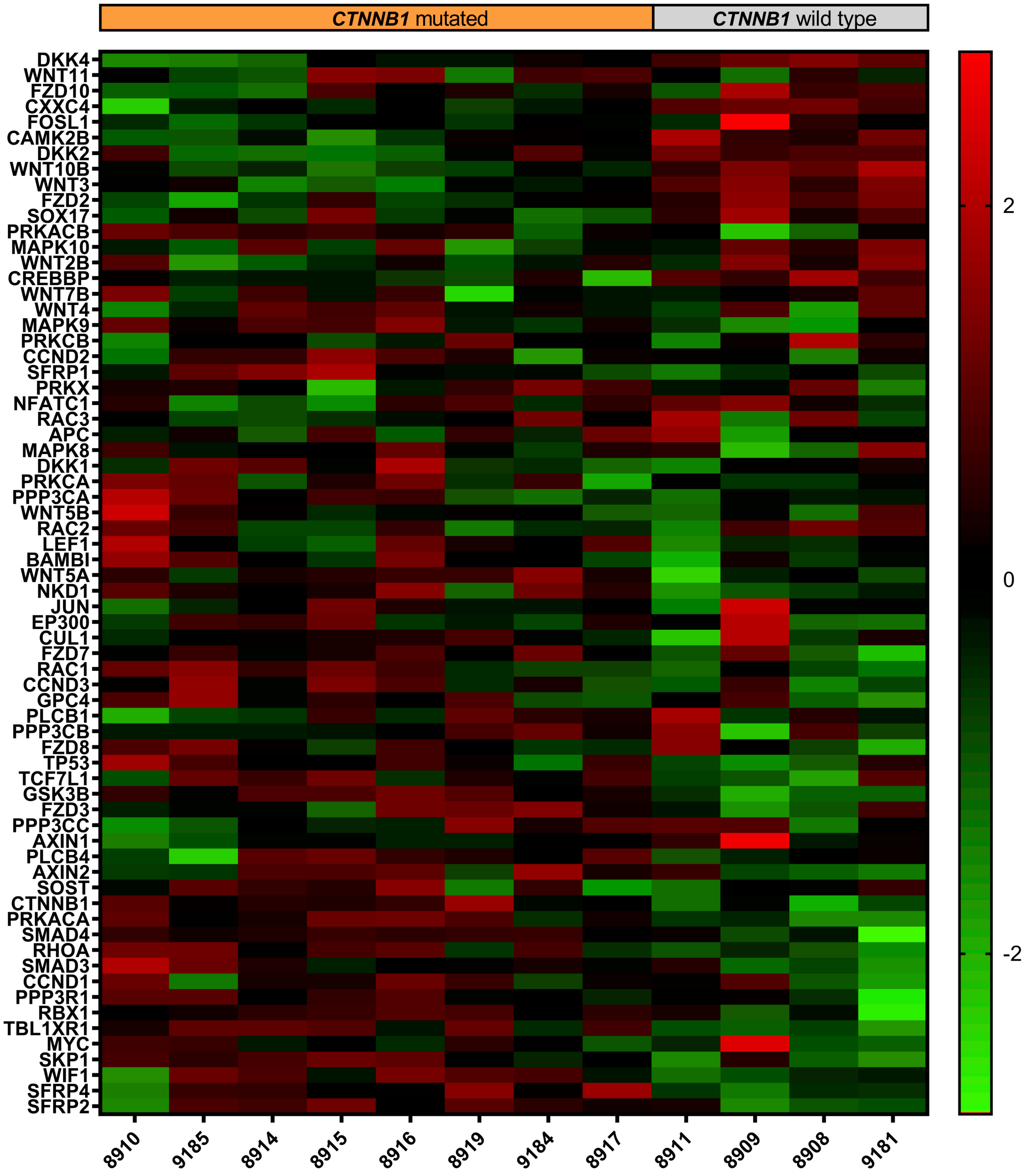

CTNNB1 mutated
CTNNB1 wild type
DKK4
WNT11
FZD10
CXXC4
FOSL1
CAMK2B
DKK2
WNT10B
WNT3
FZD2
SOX17
PRKACB
MAPK10
WNT2B
CREBBP
WNT7B
WNT4
MAPK9
PRKCB
CCND2
SFRP1
PRKX
NFATC1
RAC3
APC
MAPK8
DKK1
PRKCA
PPP3CA
WNT5B
RAC2
LEF1
BAMBI
WNT5A
NKD1
JUN
EP300
CUL1
FZD7
RAC1
CCND3
GPC4
PLCB1
PPP3CB
FZD8
TP53
TCF7L1
GSK3B
FZD3
PPP3CC
AXIN1
PLCB4
AXIN2
SOST
CTNNB1
PRKACA
SMAD4
RHOA
SMAD3
CCND1
PPP3R1
RBX1
TBL1XR1
MYC
SKP1
WIF1
SFRP4
SFRP2
8910
9185
8914
8915
8916
8919
9184
8917
8911
8909
8908
9181
2
0
-2

**Manuscript title**	Recurrent CTNNB1 mutations in craniofacial osteomas
**Authors**	Daniel Baumhoer, Ruth Berthold, Ilka Isfort, Lorena Heinst, Baptiste Ameline, Inga Grünewald, Florian M. Thieringer, Claudia Rudack, Eva Wardelmann, Volker Vieth, Jan Sperveslage, Marcel Trautmann, and Wolfgang Hartm

Supplementary material / Supporting information
nSolver Analysis Software 4.0 (NanoString Technology) dataset

	Sheet	Description
1	mRNA normalized data	mRNA - normalized log2 and linear count data per sample
2	heatmap normalizedData	normalized data of all genes per sample / heatmap
3	heatmap probe.annot	probe annotation / heatmap
4	selected housekeepers	housekeeping (HK) genes selected by geNorm
5	mRNA normalization summary	mRNA normalization factors per sample

ıann

mRNA - normalized log2 count data

	CALML3-mRNA	PIK3R2-mRNA
**8908-Osteoma_WT**	6.38065783336908	7.38065783336908
**8910-Osteoma**	2.95455944847005	7.59014802226117
**8911-Osteoma_WT**	5.82345634331618	8.26202935705581
**8916-Osteoma**	3.18674553538371	7.97499163449361
**8917-Osteoma**	5.53432269152217	7.46861944447134
**8919-Osteoma**	2.89032295348009	7.34244977149416
**9184-Osteoma**	4.54299311181193	7.92818610015731
**8909-Osteoma_WT**	6.73027730452541	6.3469486649739
**8914-Osteoma**	4.29503997741407	7.04294104926143
**8915-Osteoma**	5.03083651890339	6.66732932026463
**9181-Osteoma_WT**	5.67206923366443	6.95217715285717
**9185-Osteoma**	2.81510827152818	6.48919666272232

mRNA - normalized linear count data

	CALML3-mRNA	PIK3R2-mRNA
**8908-Osteoma_WT**	83.3238637150305	166.647727430061
**8910-Osteoma**	7.75195098360235	192.691353020973
**8911-Osteoma_WT**	56.6284973631495	306.986064652863
**8916-Osteoma**	9.10554604170814	251.600614310357
**8917-Osteoma**	46.3443863318307	177.124435432613
**8919-Osteoma**	7.41436404659761	162.292190797748
**9184-Osteoma**	23.3118745189016	243.568895835421
**8909-Osteoma_WT**	106.173307777122	81.3995359624602
**8914-Osteoma**	19.6307035998722	131.867117660011
**8915-Osteoma**	32.6913379702309	101.640341689263
**9181-Osteoma_WT**	50.9874129446101	123.826574294053
**9185-Osteoma**	7.03772075516361	89.8344355217944

IL11RA-mRNA	BAMBI-mRNA	PLA2G10-mRNA	ETV1-mRNA
7.86608466053932	6.73680164359436	4.73680164359436	4.73680164359436
7.32711361642738	10.1714207638333	1.14720452641244	3.26268174383238
10.2690157873719	4.7454538313149	2.57552882987259	3.89745692475995
6.8988199540082	9.65048499550447	3.79679901706769	5.39824964057742
8.36130642032871	6.52440722265709	2.51442313408447	4.80392975127945
8.59230318969697	7.78109388372534	1.30536045275894	5.25955676314581
8.83221704162659	7.70182040437092	2.26997461740552	4.63920842707124
6.77758301930376	8.05220539941277	5.14531480380425	5.82338670891689
7.74014481455226	7.82404607216121	5.67836861696557	5.86951010431758
8.65035624166092	6.78863561648676	3.41940180682104	6.06965576779392
6.95217715285717	7.38827626766384	4.44967681232799	6.86471431160683
8.04505804404271	9.02226617916947	-0.27235456972216	5.10268486162476

IL11RA-mRNA	BAMBI-mRNA	PLA2G10-mRNA	ETV1-mRNA
233.306818402085	106.654545555239	26.6636363888098	26.6636363888098
160.576127517477	1153.19499394161	2.2148431381721	9.59765359874576
1233.90515307073	26.824025066755	5.9608944592789	14.9022361481972
119.330577072912	803.684247997082	13.8979386952387	42.1730553510693
328.854686573813	92.0539180563761	5.71369146556817	27.9336027205555
385.958839536775	219.959466715729	2.4714546821992	38.3075475740876
455.787339731628	208.199155186053	4.82314645218654	24.9195900029638
109.712418036359	265.433269442805	35.391102592374	56.6257641477984
213.803967468173	226.606600250699	51.2105311301014	58.4653563735324
401.806263052292	110.55616113569	10.6989833357119	67.165839829747
123.826574294053	167.530071103719	21.8517484048329	116.542658159109
264.121520105552	519.963368734441	0.827967147666308	34.3606366281518

POLD4-mRNA	NFKBIZ-mRNA	SMAD2-mRNA	IFNG-mRNA
6.38065783336908	8.54415656565196	8.60716636317776	4.32176414431551
6.81962986838394	6.07794186397533	8.71198914519597	-1.43775797430871
6.89745692475995	7.24795417184409	8.89745692475995	3.16049133059375
7.12369336484841	7.2875461761712	8.62181260562181	0.523780522661279
6.89908698431979	8.40519406432971	8.7064419063774	-0.655501867357843
6.90030704205272	9.01960597042506	8.49518501163896	0.720397952037781
6.8649212066993	9.40781964785391	9.05133433093018	2.00694021157172
6.57827421108036	9.74819921252267	6.63074163097449	4.40834920963804
6.91613626418894	7.5129450077582	8.64338325901624	-0.228521978642947
6.49740431882232	8.18316746033096	8.71708235546173	0.834439306099887
7.77160490721535	7.7226953067344	6.95217715285717	4.44967681232799
6.08519743489592	7.59183157493212	8.67601266186252	0.312607930998996

POLD4-mRNA	NFKBIZ-mRNA	SMAD2-mRNA	IFNG-mRNA
83.3238637150305	373.290909443336	389.955682186343	19.9977272916073
112.957000046777	67.552715714249	419.343634160584	0.369140523028683
119.217889185578	152.002808711612	476.871556742312	8.94134168891835
139.45862621774	156.232000505098	393.934676120215	1.43771779605918
119.352666169646	339.012360290378	417.734331593762	0.634854607285352
119.453642972962	519.005483261833	360.832383601084	1.64763645479947
116.559372594508	679.259792016272	530.54610974052	4.01928871015545
95.5559769994098	860.003792994688	99.0950872586472	21.2346615554244
120.771502581822	182.650894364028	399.868897240875	0.853508852168356
90.3469703904561	290.655713953507	420.826677871335	1.78316388928532
218.517484048329	211.233567913384	123.826574294053	21.8517484048329
67.8933061086372	192.91634540625	409.015770947156	1.24195072149946

TNFRSF10C-mRNA	AKT2-mRNA	KITLG-mRNA	ALKBH3-mRNA
4.05872973848172	8.79208407909555	6.78119576295281	5.8242644848447
2.46913262129981	8.74587740716451	6.61752446119248	5.40773207663566
3.89745692475995	10.0990907859296	5.89745692475995	4.57552882987259
4.86955535950301	8.87545596094269	7.72998091049514	6.26973490005474
4.20247912776973	9.24988513766029	7.42131372969299	5.83635122897183
4.30536045275894	9.65187418592457	7.29782678007353	6.13825046692368
4.14444373532166	9.40952596980431	7.00694021157172	5.75110130714213
5.40834920963804	8.21570413169565	8.37797556059452	5.9933117103592
2.94140302279937	9.23602377169099	8.30480775366289	6.24721145232345
4.49740431882232	8.96200380581855	7.76122945914611	5.75727144557743
2.86471431160683	9.6196018137703	6.86471431160683	6.18664240649419
3.68184174066471	8.62397783418778	8.33682416841982	5.75001324330629

TNFRSF10C-mRNA	AKT2-mRNA	KITLG-mRNA	ALKBH3-mRNA
16.6647727430061	443.282954963962	109.98750010384	56.6602273262207
5.53710784543025	429.310428282358	98.1913791256297	42.4511601482985
14.9022361481972	1096.80458050732	59.608944592789	23.8435778371156
29.2335951865367	469.654480045999	212.302994551406	77.1575217218427
18.4107836112752	608.825568386653	171.410743967045	57.1369146556817
19.7716374575936	804.458499055841	157.349281433349	70.4364584426773
17.684870324684	680.063649758303	128.617238724975	53.8584687160831
42.4693231108488	297.285261775942	332.676364368315	63.7039846662732
7.68157966951521	603.004004056944	316.225029728376	75.9622878429837
22.586742597614	498.691501036794	216.951606529714	54.0893046416547
7.2839161349443	786.662942573984	116.542658159109	72.8391613494429
12.8334907888278	394.526345862995	323.321171163693	53.81786459831

JAK1-mRNA	BCL2A1-mRNA	CCR7-mRNA	FGF11-mRNA
7.84532610037253	5.64369223920288	4.32176414431551	5.64369223920288
8.38401600766186	1.36959694774889	0.884170120578651	6.13209763402224
8.31699581627374	2.57552882987259	3.57552882987259	6.0990907859296
8.46237997799713	2.74617294399773	2.63925774008122	4.893014332327
8.60424139633294	2.92946063336331	3.15185305469976	5.07241858720536
9.14035812988567	2.96832546548137	3.11271537481654	4.6272885476463
8.40952596980431	2.68501211668436	4.20857407274137	4.85493711812667
9.13626966420124	5.73027730452541	4.14531480380425	5.40834920963804
8.50109876491009	2.35644052207821	2.23090963999435	4.0568802402193
8.72115201977078	1.57140490026609	2.94991652351982	4.80406565705637
7.95217715285717	5.67206923366443	4.44967681232799	6.32414593024413
8.44189094794396	2.53500035233544	2.72764543027784	4.40007077224934

JAK1-mRNA	BCL2A1-mRNA	CCR7-mRNA	FGF11-mRNA
229.973863853484	49.9943182290183	19.9977272916073	49.9943182290183
334.072173340958	2.58398366120078	1.84570261514342	70.1366993754498
318.907853571421	5.9608944592789	11.9217889185578	68.5502862817074
352.720099299852	6.70934971494284	6.23011044958978	29.7128344518897
389.165874265921	7.61825528742422	8.88796450199493	33.6472941861237
564.315485768818	7.82627316029748	8.65009138769722	24.714546821992
340.031824879151	6.43086193624873	18.4887280667151	28.9388787131193
562.718531218747	53.086653888561	17.695551296187	42.4693231108488
362.314507745467	5.12105311301014	4.69429868692596	16.643422617283
422.015453797526	2.97193981547553	7.72704352023638	27.93623426547
247.653148588106	50.9874129446101	21.8517484048329	80.1230774843872
347.74620201985	5.79577003366415	6.62373718133046	21.1131622654908

HSPA6-mRNA	ZBTB16-mRNA	INHBA-mRNA	NRAS-mRNA
7.26036359965137	4.32176414431551	6.38065783336908	5.43724136173545
4.86602277386839	4.05409512202096	6.58460983871974	5.49297936325418
5.7454538313149	7.00179358457469	6.38288375193019	4.89745692475995
5.56817464201973	4.82146107130196	8.52940507185516	5.47797683304815
6.95182844639177	6.55395149827111	7.05874365030828	5.07241858720536
7.33510779615299	8.01271958483982	7.44149714074497	4.94921664253366
7.74570804837192	9.45319644146129	9.01592899479898	5.29972196079957
9.02305905375325	7.37797556059453	9.09951111419113	6.28281832755419
6.23090963999435	6.78270527678031	5.53966234613398	4.79384583438551
6.24947680537873	9.91481272256391	5.89333299515346	5.18021414294162
7.03463931304914	7.38827626766384	4.44967681232799	4.86471431160683
6.30507425831359	10.2614618197112	6.24334526856188	5.34235527439305

HSPA6-mRNA	ZBTB16-mRNA	INHBA-mRNA	NRAS-mRNA
153.315909235656	19.9977272916073	83.3238637150305	43.3284091318159
29.162101319266	16.6113235362907	95.9765359874576	45.0351438094993
53.6480501335101	128.159230874496	83.4525224299046	29.8044722963945
47.4446872699529	28.2751166558305	369.49347358721	44.5692516778346
123.796648420644	93.9584818782321	133.319467529924	33.6472941861237
161.468372570348	258.267014289817	173.825645981344	30.89318352749
214.630017122301	700.963951051111	517.684385868023	39.3890293595235
520.249208107898	166.338182184158	548.562090181797	77.8604257032228
75.1087789908154	110.102641929718	46.5162324431754	27.7390376954716
76.0816592761736	965.286052066454	59.4387963095106	36.2576657488015
131.110490428997	167.530071103719	21.8517484048329	29.1356645397772
79.0708626021324	1227.4612964153	75.7589940114672	40.5703902356491

**MEN1-mRNA**	**EPO-mRNA**	**ITGB8-mRNA**	**CDC14B-mRNA**
7.16306639829646	2.73680164359436	5.73680164359436	6.78119576295281
7.65235444535558	-0.437757974308711	4.29016248025449	6.85686277458291
7.7454538313149	3.16049133059375	5.38288375193019	5.82345634331618
7.16281969613823	-1.06118197805988	6.43866390902333	5.83363578524807
7.94441097482928	0.344498132642157	6.20247912776973	4.77076288734425
6.98249279740796	2.04232604692514	6.17982957067508	6.18800350212078
6.63920842707124	2.85493711812667	4.97041433554661	6.07732953946312
7.91084955016723	4.14531480380425	6.68136770404446	6.73027730452541
7.06149686828967	0.356440522078209	7.22680524166161	6.00029671185293
7.22675672887865	2.57140490026609	7.13822005427699	6.11984152496214
7.11264182505042	5.18664240649419	6.95217715285717	7.18664240649419
7.01304764914009	0.72764543027784	4.63453602588636	6.75001324330629

**MEN1-mRNA**	**EPO-mRNA**	**ITGB8-mRNA**	**CDC14B-mRNA**
143.317045589852	6.66590909720244	53.3272727776195	109.98750010384
201.181585050632	0.738281046057366	19.5644477205202	115.910124231006
214.59220053404	8.94134168891835	41.7262612149523	56.6284973631495
143.292540340565	0.47923926535306	86.7423070289039	57.0294725770142
246.323587626716	1.2697092145707	73.6431344451008	27.2987481132701
126.456097905859	4.11909113699867	72.4960040111767	72.9079131248765
99.6783600118552	7.23471967827982	31.3504519392126	67.5240503306117
240.659497628143	17.695551296187	102.634197517885	106.173307777122
133.574135364348	1.28026327825253	149.790803555547	64.0131639126267
149.785766699967	5.94387963095106	140.86994725354	69.5433916821275
138.394406563942	36.4195806747215	123.826574294053	145.678322698886
129.162875035944	1.65593429533262	24.8390144299892	107.63572919662

SETBP1-mRNA	CASP12-mRNA	IL13-mRNA	KAT2B-mRNA
9.06771852170898	4.54415656565196	4.54415656565196	6.38065783336908
8.66945910128267	1.14720452641244	-0.437757974308711	6.3887905129822
10.6416180203304	4.16049133059375	3.89745692475995	7.50626616743548
8.59345405046809	2.74617294399773	-1.06118197805988	5.98321214129858
9.64141433952144	4.38889225200061	2.80392975127945	7.32177805614207
9.60685664774149	2.6272885476463	1.52775287409539	7.60304100139962
9.27372675226663	3.68501211668436	1.68501211668436	6.46637183020902
7.8006666324168	4.63074163097449	4.63074163097449	6.21570413169565
8.6723448293378	4.12903002597514	-0.228521978642947	6.83756721181483
8.66521857366882	4.05683172743634	2.41940180682104	6.95683593745961
8.69760432577157	4.44967681232799	5.67206923366443	6.44967681232799
7.26680424138587	2.53500035233544	0.312607930998996	6.62851223825859

SETBP1-mRNA	CASP12-mRNA	IL13-mRNA	KAT2B-mRNA
536.605682324797	23.3306818402085	23.3306818402085	83.3238637150305
407.161996900637	2.2148431381721	0.738281046057366	83.7948987275111
1597.51971508674	17.8826833778367	14.9022361481972	181.807281008006
386.266847874566	6.70934971494284	0.47923926535306	63.2595830266039
798.647095964972	20.9502020404166	6.98340068013887	159.983361035909
779.743952233849	6.17863670549801	2.88336379589907	194.421101666337
618.97046136394	12.8617238724975	3.21543096812436	88.42435162342
222.963946331956	24.7737718146618	24.7737718146618	74.3213154439854
407.977231336474	17.4969314694513	0.853508852168356	114.37018619056
405.966978793957	16.642862966663	5.34949166785596	124.227084286877
415.183219691825	21.8517484048329	50.9874129446101	87.4069936193316
154.001889465933	5.79577003366415	1.24195072149946	98.9420741461238

GNG4-mRNA	BMP6-mRNA	PPARG-mRNA	PBX1-mRNA
6.98472915703794	6.54415656565196	6.64369223920288	8.43724136173545
8.04404145735704	5.31712952785476	5.22045350844308	7.62023374845046
1.57552882987259	5.27596854801368	6.89745692475995	9.79469735033475
6.40642357202312	6.44661266213882	5.12864258082014	7.48571248182776
5.95920797675736	5.09938563480562	5.92946063336331	8.87001894173723
6.0513148301524	6.32772826578739	6.44831840660098	9.67095384908529
5.11127687138646	4.97041433554661	5.68501211668436	8.10286463157026
5.40834920963804	8.4380965530321	6.77758301930376	6.52382642705798
5.29503997741407	6.03826456205195	5.57883294341466	9.18404786816226
6.11984152496214	7.33693964662907	6.21526109004082	8.86602564915772
6.18664240649419	6.67206923366443	6.18664240649419	9.20456431449145
4.70492535377776	5.81510827152818	6.14549794516374	8.24531481841165

GNG4-mRNA	BMP6-mRNA	PPARG-mRNA	PBX1-mRNA
126.652272846846	93.3227273608341	99.9886364580366	346.627273054526
263.935473965509	39.8671764870978	37.283192825897	196.751898774288
2.98044722963945	38.7458139853129	119.217889185578	888.173274432556
84.8253499674916	87.2215462942569	34.9844663707734	179.235485242045
62.2157515139645	34.282148793409	60.9460422993938	467.887845569305
66.3173673056786	80.3222771714741	87.3247321043718	815.168136012038
34.5658829073369	31.3504519392126	51.4468954899898	274.919347774633
42.4693231108488	346.832805405265	109.712418036359	92.0168667401724
39.2614071997444	65.7201816169634	47.796495721428	581.666282752735
69.5433916821275	161.673525961869	74.2984953868883	466.594551029658
72.8391613494429	101.97482588922	72.8391613494429	589.997206930488
26.0809651514887	56.3017660413089	70.7911911254693	303.449959619701

PRKACA-mRNA	IL2RA-mRNA	PRL-mRNA	PAX3-mRNA
6.94625500922331	5.54415656565196	5.05872973848172	4.05872973848172
8.26268174383238	3.42022302081886	4.69152504263625	2.36959694774889
7.43350982500016	3.89745692475995	3.57552882987259	3.16049133059375
8.38176151778885	4.39824964057742	2.74617294399773	7.59345405046809
7.86806008869917	4.77076288734425	2.66642622752952	8.41596049519878
8.18596435684718	3.72039795203778	1.04232604692514	8.86887453421606
7.44656334912884	4.26997461740552	3.14444373532166	9.40440093762644
7.52382642705798	5.73027730452541	5.63074163097449	4.9933117103592
7.88782198259452	2.57883294341466	-0.228521978642947	7.66022127025531
8.34750888833926	4.05683172743634	1.24947680537873	8.15335865111491
6.95217715285717	5.18664240649419	4.44967681232799	7.7226953067344
7.69342971493993	4.01304764914009	-1.27235456972216	6.7219988671367

PRKACA-mRNA	IL2RA-mRNA	PRL-mRNA	PAX3-mRNA
123.319318298245	46.6613636804171	33.3295454860122	16.6647727430061
307.124915159864	10.7050751678318	25.8398366120078	5.16796732240156
172.865939319088	14.9022361481972	11.9217889185578	8.94134168891835
333.55052868573	21.0865276755346	6.70934971494284	193.133423937283
233.626495481009	27.2987481132701	6.34854607285352	341.551778719519
291.219743385806	13.1810916383958	2.05954556849934	467.516844049349
174.437130020747	19.2925858087462	8.842435162342	677.65207653221
184.033733480345	53.086653888561	49.5475436293236	31.8519923331366
236.848706476719	5.9745619651785	0.853508852168356	202.2815979639
325.724603776119	16.642862966663	2.37755185238043	284.711834322556
123.826574294053	36.4195806747215	21.8517484048329	211.233567913384
206.991786916577	16.145359379493	0.413983573833154	105.565811327454

BIRC3-mRNA	PRKAA2-mRNA	PRKCG-mRNA	UBB-mRNA
5.32176414431551	5.19623326223166	2.73680164359436	11.0129260488686
2.26268174383238	1.7321670271336	0.147204526412445	10.4691326212998
4.7454538313149	2.57552882987259	1.57552882987259	10.2229872563275
4.91609794544004	4.06810103888509	0.523780522661279	12.3589098265424
4.83635122897183	3.92946063336331	0.344498132642157	11.5736173533008
4.96832546548137	2.30536045275894	2.04232604692514	11.5154229359992
5.54299311181193	2.85493711812667	1.68501211668436	11.7866595667151
4.40834920963804	5.73027730452541	1.82338670891689	10.588258299653
4.88000247813522	4.980931386986	-1.22852197864295	11.186955729055
4.80406565705637	5.58932680826336	2.05683172743634	11.2942124314356
5.18664240649419	5.67206923366443	2.86471431160683	7.67206923366443
3.97557294372143	1.89757043172015	0.72764543027784	10.4787710136451

BIRC3-mRNA	PRKAA2-mRNA	PRKCG-mRNA	UBB-mRNA
39.9954545832146	36.6625000346134	6.66590909720244	2066.43182013276
4.79882679937288	3.32226470725815	1.10742156908605	1417.49960843014
26.824025066755	5.9608944592789	2.98044722963945	1195.15933908542
30.1920737172428	16.7733742873571	1.43771779605918	5252.94158753489
28.5684573278408	15.2365105748484	1.2697092145707	3047.93696957698
31.3050926411899	4.94290936439841	4.11909113699867	2927.43807106496
46.6237490378033	7.23471967827982	3.21543096812436	3532.95477622664
21.2346615554244	53.086653888561	3.5391102592374	1539.51296276827
29.4460553998083	31.5798275302292	0.426754426084178	2331.35942969786
27.93623426547	48.1454250107036	4.16071574166575	2511.28914407682
36.4195806747215	50.9874129446101	7.2839161349443	203.94965177844
15.7313758056598	3.72585216449838	1.65593429533262	1427.00137900288

HDAC6-mRNA	FZD9-mRNA	PIK3CA-mRNA	SMARCA4-mRNA
6.59478263872193	5.19623326223166	6.54415656565196	6.78119576295281
6.70690026852317	2.46913262129981	6.81490745814154	7.67858598692876
7.16049133059375	4.89745692475995	7.06738192620227	6.66299167112293
7.20560456263502	3.63925774008121	6.83363578524807	7.32252231441417
7.78328998522042	3.25138872825068	6.87587959315847	7.03798509014148
7.96118928419974	2.04232604692514	7.41388490953711	6.83933902476129
7.34322359943616	3.38545183482545	7.26997461740552	6.45319644146129
6.28281832755419	6.07131422236047	6.91084955016723	7.10878892777914
7.3489068493928	2.57883294341466	7.13342179509229	7.05224879148766
7.42440248787941	3.05683172743634	7.09496685632311	6.99094379177988
7.6196018137703	5.18664240649419	7.18664240649419	6.77160490721535
6.61028847963968	2.72764543027784	7.03142617845494	6.57313548122221

HDAC6-mRNA	FZD9-mRNA	PIK3CA-mRNA	SMARCA4-mRNA
96.6556819094353	36.6625000346134	93.3227273608341	109.98750010384
104.466768017117	5.53710784543025	112.587859523748	204.872990280919
143.061467022694	29.8044722963945	134.120125333775	101.335205807741
147.605693728743	12.4602208991796	114.058945154028	160.065914627922
220.294548728017	9.52281910928028	117.44810234779	131.414903708068
249.20501378842	4.11909113699867	170.530373071745	114.510733608563
162.37926389028	10.4501506464042	154.340686469969	87.6204938813889
77.8604257032228	67.2430949255106	120.329748814072	138.025300110259
163.020190764156	5.9745619651785	140.402206181695	132.72062651218
171.778121334486	8.32143148333149	136.709231511874	127.199024102353
196.665735643496	36.4195806747215	145.678322698886	109.258742024164
97.7001234246243	6.62373718133046	130.818809331277	95.2162219816253

ABL1-mRNA	COL24A1-mRNA	TSLP-mRNA	RASA4-mRNA
7.05872973848172	5.8242644848447	4.05872973848172	7.8242644848447
7.37602321690833	8.164012814099	1.88417012057865	7.89091695301924
7.70481184681756	6.70481184681756	3.57552882987259	9.22658052105152
7.29637002655821	7.8636305255459	2.26074611682749	7.36927057360565
7.68434813552678	6.49424525214684	2.51442313408447	7.227141182004
8.00348630506178	6.29782678007353	1.30536045275894	6.25955676314581
7.80395318940787	6.61574945424725	1.68501211668436	6.97041433554661
9.04255522937905	4.40834920963804	4.82338670891689	8.09017324961179
7.40083464143666	5.84829361840788	2.67836861696557	7.495991874477
8.07602529266964	5.69242030122746	3.70890842401603	8.21526109004082
6.18664240649419	4.86471431160683	5.18664240649419	7.7226953067344
7.08079225577592	5.70492535377776	2.42808514841893	7.96126510703754

ABL1-mRNA	COL24A1-mRNA	TSLP-mRNA	RASA4-mRNA
133.318181944049	56.6602273262207	16.6647727430061	226.640909304883
166.113235362907	286.822186393287	3.69140523028683	237.357356307443
208.631306074761	104.315653037381	11.9217889185578	599.06989315753
157.190479035804	232.910282961587	4.7923926535306	165.337546546806
205.692892760454	90.14935423452	5.71369146556817	149.825687319343
256.619377835018	78.6746407166747	2.4714546821992	76.6150951481753
223.472452284643	98.0706445277931	3.21543096812436	125.40180775685
527.327428626372	21.2346615554244	28.3128820738992	272.51148996128
168.994752729335	57.6118475213641	6.40131639126267	180.517122233607
269.852135245178	51.7117527892742	13.0765351880923	297.193981547553
72.8391613494429	29.1356645397772	36.4195806747215	211.233567913384
135.372628643441	52.1619303029774	5.381786459831	249.218111447558

STAT1-mRNA	IKBKG-mRNA	MAPT-mRNA	LAMA3-mRNA
6.90672664503667	7.94625500922331	4.73680164359436	5.32176414431551
7.4540257289096	6.88417012057865	2.02167364432859	5.1915986457709
5.96784625265135	8.45817187923443	4.7454538313149	5.27596854801368
8.55720352419873	7.25170097722448	3.39824964057742	4.18674553538371
6.95182844639177	7.92946063336331	3.51442313408447	4.29869444302903
7.51481381838789	7.8236857604498	3.17982957067508	4.52775287409538
7.41293257124756	7.7724749579347	3.38545183482545	3.85493711812667
7.03284007454584	7.46724289869161	3.40834920963804	5.9933117103592
8.16164697755724	6.99064654181922	2.47191773949815	5.60436803552179
7.63749409072387	7.13211985474057	2.83443930609989	6.06965576779392
7.38827626766384	7.18664240649419	2.86471431160683	4.44967681232799
7.81775784994213	6.48253293244131	1.72764543027784	4.21949852660751

STAT1-mRNA	IKBKG-mRNA	MAPT-mRNA	LAMA3-mRNA
119.986363749644	246.63863659649	26.6636363888098	39.9954545832146
175.341748438624	118.124967369179	4.06054575331551	36.5449117798396
62.5893918224285	351.692773097455	26.824025066755	38.7458139853129
376.682062567505	152.398086382273	10.5432638377673	18.2110920834163
123.796648420644	243.784169197575	11.4273829311363	19.6804928258459
182.887646482741	226.550012534927	9.06200050139708	23.0669103671926
170.417841310591	218.649305832457	10.4501506464042	14.4694393565596
130.947079591784	176.95551296187	10.6173307777122	63.7039846662732
286.352219902484	127.172818973085	5.54780753909432	48.6500045735963
199.119967636861	140.275559290445	7.13265555714128	67.165839829747
167.530071103719	145.678322698886	7.2839161349443	21.8517484048329
225.621047739069	89.4204519479612	3.31186859066523	18.6292608224919

CDK4-mRNA	PPP3CA-mRNA	ITGA2-mRNA	FIGF-mRNA
5.05872973848172	5.98472915703794	5.32176414431551	5.19623326223166
6.62293795737884	7.51060925727597	6.49887996469386	2.02167364432859
5.96784625265135	5.38288375193019	5.7454538313149	1.57552882987259
6.35667053682602	6.57544264248377	7.31819638901138	3.58267421171485
5.77076288734425	5.88365694375019	6.0859651190433	3.15185305469976
6.07794995665587	5.57837894716535	6.12127738831997	3.11271537481654
4.97041433554661	5.38545183482545	5.91383080718024	2.68501211668436
5.40834920963804	6.07131422236047	5.63074163097449	3.82338670891689
5.84829361840788	6.20610624899378	6.70221535891994	1.94140302279937
6.16834004265333	6.61579901962455	5.80406565705637	3.77303876143574
5.86471431160683	6.03463931304914	6.56515402974792	5.44967681232799
6.37869712145677	6.94681395074	6.58562642540541	1.89757043172015

CDK4-mRNA	PPP3CA-mRNA	ITGA2-mRNA	FIGF-mRNA
33.3295454860122	63.3261364234232	39.9954545832146	36.6625000346134
98.5605196486584	182.355418376169	90.4394281420274	4.06054575331551
62.5893918224285	41.7262612149523	53.6480501335101	2.98044722963945
81.9499143753733	95.3686138052589	159.586675362569	11.9809816338265
54.5974962265403	59.0414784775377	67.9294429795327	8.88796450199493
67.5530946467782	47.7814571891846	69.6126402152776	8.65009138769722
31.3504519392126	41.8006025856167	60.2893306523318	6.43086193624873
42.4693231108488	67.2430949255106	49.5475436293236	14.1564410369496
57.6118475213641	73.8285157125628	104.12807996454	3.8407898347576
71.9209435345079	98.0740139106926	55.87246853094	13.6709231511874
58.2713290795544	65.5552452144987	94.6909097542759	43.7034968096658
83.2106983404639	123.36710500228	96.0441891292917	3.72585216449838

MMP9-mRNA	CACNA1D-mRNA	SSX1-mRNA	HELLS-mRNA
10.3770465798167	4.73680164359436	4.90672664503667	5.19623326223166
12.3878964959902	5.05409512202096	1.36959694774889	4.60663614504974
9.05126226083899	5.38288375193019	3.89745692475995	4.38288375193019
13.2948572629241	6.21494242721436	1.74617294399773	4.91609794544004
10.5156749302939	6.04493785078325	0.929460633363313	5.45302258942033
8.9310692958234	4.33510779615299	1.04232604692514	4.96832546548137
10.4753606134363	5.46637183020902	-0.314987883315639	4.7724749579347
12.2621785614951	2.82338670891689	3.40834920963804	5.63074163097449
7.47538159480172	6.37880833510666	0.356440522078209	6.4509581208625
7.78085826589504	6.21526109004082	1.57140490026609	6.01766113015566
7.25703173438559	5.67206923366443	5.86471431160683	5.18664240649419
9.20337886124424	3.42808514841893	-1.27235456972216	4.312607930999

MMP9-mRNA	CACNA1D-mRNA	SSX1-mRNA	HELLS-mRNA
1329.84886489189	26.6636363888098	29.996590937411	36.6625000346134
5359.55125385345	33.2226470725815	2.58398366120078	24.3632745198931
530.519606875822	41.7262612149523	14.9022361481972	20.8631306074761
10049.6473944537	74.2820861297243	3.35467485747142	30.1920737172428
1463.97472440002	66.0248791576766	1.90456382185606	43.8049679026893
488.112299734343	20.1835465712935	2.05954556849934	31.3050926411899
1423.63206113706	44.21217581171	0.803857742031091	27.3311632290571
4912.28503982151	7.0782205184748	10.6173307777122	49.5475436293236
177.956595677102	83.2171130864147	1.28026327825253	87.4846573472566
219.923546345189	74.2984953868883	2.97193981547553	64.7882879773666
152.96223883383	50.9874129446101	58.2713290795544	36.4195806747215
589.512609138412	10.763572919662	0.413983573833154	19.8712115439914

TNC-mRNA	COL4A6-mRNA	MFNG-mRNA	HDAC2-mRNA
5.43724136173545	2.73680164359436	5.90672664503667	6.69099795398123
5.93728145703821	1.36959694774889	4.42022302081886	7.96738348882763
4.7454538313149	2.57552882987259	3.16049133059375	6.48241942548111
6.25170097722448	3.39824964057742	4.82146107130196	7.73323388829023
4.62990035150441	2.92946063336331	5.34449813264216	6.92192696067791
5.81843003499831	1.30536045275894	5.16334144788651	7.091085358845
5.83475923618904	2.00694021157172	5.81429513362933	6.29972196079957
7.57827421108036	3.40834920963804	4.40834920963804	7.18093871353497
6.09340611624442	4.65412107071889	5.09340611624442	7.39718686442152
5.72521023634513	5.29387092473718	6.0043643075422	7.69656003158838
6.18664240649419	4.44967681232799	5.86471431160683	6.77160490721535
6.74445371796439	1.0495735251652	5.72764543027784	8.01304764914009

TNC-mRNA	COL4A6-mRNA	MFNG-mRNA	HDAC2-mRNA
43.3284091318159	6.66590909720244	59.9931818748219	103.321591006638
61.2773268227614	2.58398366120078	21.4101503356636	250.277274613447
26.824025066755	5.9608944592789	8.94134168891835	89.4134168891835
76.1990431911366	10.5432638377673	28.2751166558305	212.782233816759
24.7593296841287	7.61825528742422	40.6306948662625	121.257229991502
56.4315485768818	2.4714546821992	35.8360928918884	136.341916634656
57.0738996842075	4.01928871015545	56.2700419421764	78.7780587190469
191.11195399882	10.6173307777122	21.2346615554244	145.103520628733
68.2807081734685	25.1785111389665	34.1403540867343	168.56799830325
52.9005287154645	39.229605564277	64.1939000142715	207.441399120192
72.8391613494429	21.8517484048329	58.2713290795544	109.258742024164
107.221745622787	2.06991786916577	52.9898974506437	258.325750071888

BMP7-mRNA	IL11-mRNA	RASGRF1-mRNA	PRKDC-mRNA
6.43724136173545	4.90672664503667	1.73680164359436	8.50498596837128
7.53378557964206	3.56224202569129	1.14720452641244	8.23289827480973
5.82345634331618	4.16049133059375	3.16049133059375	8.95056826121952
8.46433883103519	4.82146107130196	0.938818021940123	9.11499117104761
6.81210368272515	2.92946063336331	0.344498132642157	8.55638642718816
6.29782678007353	2.04232604692514	0.720397952037781	8.72461841835598
7.58587892466511	3.7724749579347	0.685012116684361	9.08589155296654
8.18093871353497	6.73027730452541	1.82338670891689	7.52382642705798
7.50957028097754	1.35644052207821	2.77147802135705	8.27331520625935
6.92190214735023	2.57140490026609	1.57140490026609	8.39668173032096
8.38827626766384	5.18664240649419	4.44967681232799	7.11264182505042
7.01766427721046	1.312607930999	1.312607930999	7.89003675903475

BMP7-mRNA	IL11-mRNA	RASGRF1-mRNA	PRKDC-mRNA
86.6568182636317	29.996590937411	3.33295454860122	363.292045797533
185.308542560399	11.8124967369179	2.2148431381721	300.849526268377
56.6284973631495	17.8826833778367	8.94134168891835	494.754240120149
353.199338565205	28.2751166558305	1.91695706141224	554.479830013491
112.369265489507	7.61825528742422	1.2697092145707	376.468782120214
78.6746407166747	4.11909113699867	1.64763645479947	423.030659769764
192.122000345431	13.6655816145285	1.60771548406218	543.407833613017
290.207041257467	106.173307777122	3.5391102592374	184.033733480345
182.224139937944	2.56052655650507	6.82807081734685	309.396958911029
121.255144471402	5.94387963095106	2.97193981547553	337.017975074925
335.060142207438	36.4195806747215	21.8517484048329	138.394406563942
129.576858609777	2.48390144299892	2.48390144299892	237.212587806397

GRIN1-mRNA	PRKX-mRNA	TGFB1-mRNA	KDM6A-mRNA
4.54415656565196	5.98472915703794	9.98946707604461	6.38065783336908
-0.437757974308711	5.61752446119248	10.9217170745408	6.89763238038521
3.16049133059375	5.38288375193019	10.2690157873719	6.03496044850989
-0.061181978059877	5.36508277664222	11.4319229144793	6.64617715402101
2.66642622752952	5.78744162849088	9.76445831049005	6.39978056814335
-0.279602047962219	5.73162520746104	10.5001173071812	6.44149714074497
1.68501211668436	6.06005154803129	10.792883030964	6.50519107909955
4.82338670891689	5.40834920963804	10.6047464224415	7.10878892777914
1.09340611624442	5.48572353902318	9.49257721006424	7.16808280253891
0.834439306099887	4.57140490026609	9.49621740387187	6.87152862483511
2.86471431160683	4.86471431160683	8.69760432577157	6.18664240649419
-0.27235456972216	5.65838276784073	10.3773506957493	7.44189094794396

GRIN1-mRNA	PRKX-mRNA	TGFB1-mRNA	KDM6A-mRNA
23.3306818402085	63.3261364234232	1016.55113732337	83.3238637150305
0.738281046057366	49.0956895628149	1939.83344851573	119.232388938265
8.94134168891835	41.7262612149523	1233.90515307073	65.5698390520679
0.95847853070612	41.2145768203632	2762.81436476039	100.16100645879
6.34854607285352	55.2323508338256	869.750811980932	84.4356627689518
0.823818227399735	53.1362756672829	1448.27244376873	86.912822990672
3.21543096812436	66.7201925885805	1774.11403666262	90.8359248495133
28.3128820738992	42.4693231108488	1557.20851406446	138.025300110259
2.13377213042089	44.8092147388387	720.361471230093	143.816241590368
1.78316388928532	23.7755185238043	722.181375160554	117.094428729736
7.2839161349443	29.1356645397772	415.183219691825	72.8391613494429
0.827967147666308	50.5059960076448	1330.12922272592	173.873101009925

ETV7-mRNA	SOST-mRNA	TMPRSS2-mRNA	RAC3-mRNA
5.54415656565196	9.48499449318382	4.54415656565196	6.98472915703794
3.02167364432859	9.41399106710735	0.147204526412445	6.01345313752362
3.89745692475995	7.82345634331618	4.16049133059375	7.43350982500016
3.58267421171485	12.0137956842413	5.97224102347757	5.86955535950301
3.73681555542092	7.09938563480562	5.01692347461365	6.15185305469976
5.09543738338471	7.59998120165057	1.30536045275894	6.06912610626886
3.85493711812667	10.6231214429036	2.49236703874197	6.98879286486146
5.28281832755419	9.84019499660344	5.52382642705798	4.9933117103592
3.90076103830202	10.6317890762765	7.18510595038123	5.38618786547226
3.15636740098725	10.3903065761517	5.93597733256195	5.62451623672566
5.44967681232799	10.6328986363838	7.11264182505042	5.44967681232799
3.58562642540541	11.2485096132666	1.89757043172015	5.41414595746106

ETV7-mRNA	SOST-mRNA	TMPRSS2-mRNA	RAC3-mRNA
46.6613636804171	716.585227949263	23.3306818402085	126.652272846846
8.12109150663103	682.171686557007	1.10742156908605	64.5995915300195
14.9022361481972	226.513989452598	17.8826833778367	172.865939319088
11.9809816338265	4135.35562073155	62.7803437612509	58.4671903730733
13.3319467529924	137.128595173636	32.3775849715529	71.1037160159594
34.188456437089	194.009192552638	2.4714546821992	67.1411855330784
14.4694393565596	1577.168889865	5.62700419421764	127.009523240912
38.9302128516114	916.629557142488	46.0084333700862	31.8519923331366
14.9364049129462	1586.67295618097	145.523259294705	41.8219337562495
8.9158194464266	1342.12802066875	61.221960198796	49.3342009368938
43.7034968096658	1587.89371741786	138.394406563942	43.7034968096658
12.0055236411615	2432.98146341745	3.72585216449838	42.6403081048148

LAT-mRNA	RUNX1T1-mRNA	HRAS-mRNA	ITGA7-mRNA
6.78119576295281	7.80289083405213	4.73680164359436	6.43724136173545
5.63905762274212	6.39513203985603	4.79106071618717	5.43260674527469
6.16049133059375	9.21938501964732	4.7454538313149	6.16049133059375
6.56817464201973	6.13849036677649	5.18674553538371	5.20560456263502
6.41058732309993	8.39162204475618	5.04493785078325	6.70205013726024
6.32772826578739	7.70623988904112	5.30536045275894	7.25567332865858
6.88468446152073	7.94710696205454	4.43989961884783	6.45319644146129
5.91084955016723	6.28281832755419	4.82338670891689	6.28281832755419
6.59165698377224	7.50618764158289	4.65412107071889	6.341333629688
5.80406565705637	7.78475218199953	4.57140490026609	7.81553084354982
6.18664240649419	6.95217715285717	5.18664240649419	6.18664240649419
6.09396764452366	7.23147116827359	4.45556588484104	6.35700205035745

LAT-mRNA	RUNX1T1-mRNA	HRAS-mRNA	ITGA7-mRNA
109.98750010384	223.307954756282	26.6636363888098	86.6568182636317
49.8339706088722	84.1640392505397	27.6855392271512	43.1894411943559
71.5307335113468	596.08944592789	26.824025066755	71.5307335113468
94.8893745399059	70.4481720068998	36.4221841668326	36.9014234321856
85.0705173762372	335.838087253951	33.0124395788383	104.116155594798
80.3222771714741	208.837920645833	39.5432749151873	152.818281182651
118.16708807857	246.784326803545	21.7041590348394	87.6204938813889
60.1648744070358	77.8604257032228	28.3128820738992	77.8604257032228
96.4465002950243	181.79738551186	25.1785111389665	81.0833409559939
55.87246853094	220.517934308284	23.7755185238043	225.273038013045
72.8391613494429	123.826574294053	36.4195806747215	72.8391613494429
68.3072896824704	150.276037301435	21.9411294131571	81.9687476189644

HDAC1-mRNA	CCNE1-mRNA	IRAK2-mRNA	CASP3-mRNA
6.49168914575783	5.43724136173545	3.73680164359436	6.26036359965137
7.1584317818357	4.36959694774889	3.77169539132024	5.89315890380591
6.33041633203606	4.38288375193019	4.38288375193019	5.03496044850989
7.54984281924748	4.49340687361776	4.58267421171485	6.167636712436
7.42664717399603	4.04493785078325	5.07241858720536	5.88365694375019
7.69194150598855	3.76479207139623	4.72039795203778	5.93956647249994
7.06005154803129	3.7724749579347	4.20857407274137	5.5179021308491
6.57827421108036	5.9933117103592	7.68136770404446	6.14531480380425
7.64799496792205	2.35644052207821	4.12903002597514	6.24721145232345
7.6631047344029	3.15636740098725	4.05683172743634	6.2381614921509
6.44967681232799	4.44967681232799	4.44967681232799	5.44967681232799
7.27453989016548	2.81510827152818	4.56053544444258	5.5090051438025

HDAC1-mRNA	CCNE1-mRNA	IRAK2-mRNA	CASP3-mRNA
89.9897728122329	43.3284091318159	13.3318181944049	76.6579546178281
142.8573824121	20.6718692896062	13.6581993520613	59.431624207618
80.4720752002651	20.8631306074761	20.8631306074761	32.784919526034
187.382552753047	22.5242454715938	23.961963267653	71.885889802959
172.04559857433	16.5062197894191	33.6472941861237	59.0414784775377
206.778375077333	13.5930007520956	26.3621832767915	61.3744579412802
133.440385177161	13.6655816145285	18.4887280667151	45.8198912957722
95.5559769994098	63.7039846662732	205.268395035769	70.782205184748
200.574580259564	5.12105311301014	17.4969314694513	75.9622878429837
202.686295415431	8.9158194464266	16.642862966663	75.4872713130785
87.4069936193316	21.8517484048329	21.8517484048329	43.7034968096658
154.8298566136	7.03772075516361	23.5970637084898	45.5381931216469

HHEX-mRNA	SMO-mRNA	MYC-mRNA	RPS27A-mRNA
6.86608466053932	6.19623326223166	7.40922698556585	10.6224980169338
4.67076648246946	6.43875897225629	9.13209763402224	12.1745346198008
5.38288375193019	6.7454538313149	7.87930957804969	10.7529483678618
6.03685010490065	5.91609794544004	7.84570861754864	12.2927918437583
6.04493785078325	6.47378114958712	7.35012268183604	11.1506449359627
4.74276576506624	6.12978888817548	8.948013895452	11.1276657162825
4.14444373532166	5.87483667556438	8.28492495887149	10.8271191739869
6.73027730452541	6.40834920963804	11.0713142223605	12.0242853139553
5.32606687303469	5.99064654181921	8.0568802402193	12.3867628047222
5.78863561648676	5.96372232304485	8.38133376598752	12.7006879172111
6.32414593024413	5.86471431160683	7.18664240649419	10.0346393130491
5.38585691302963	5.85692844722281	9.0393937452828	12.7151759254421

HHEX-mRNA	SMO-mRNA	MYC-mRNA	RPS27A-mRNA
116.653409201043	73.3250000692268	169.980681978662	1576.48750148838
25.4706960889791	86.7480229117405	561.093595003599	4622.7467698882
41.7262612149523	107.29610026702	235.455331141517	1725.67894596124
65.6557793533692	60.3841474344856	230.034847369469	5017.63510824654
66.0248791576766	88.8796450199493	163.157634072336	2273.41434868885
26.7740923904914	70.0245493289774	493.87902732614	2237.49030561768
17.684870324684	58.6816151682697	311.896803908063	1816.71849699027
106.173307777122	84.9386462216976	2151.77903761634	4165.53277512242
40.1149160519128	63.5864094865425	266.294761876527	5355.34129293035
55.2780805678449	62.4107361249862	333.451647296355	6657.14518666519
80.1230774843872	58.2713290795544	145.678322698886	1048.88392343198
41.8123409571485	57.9577003366415	526.173122341938	6724.33518977191

COL2A1-mRNA	IL10-mRNA	AR-mRNA	RAD50-mRNA
5.54415656565196	2.73680164359436	5.32176414431551	5.8242644848447
6.00518552154002	1.36959694774889	5.48110526296588	5.9285642399371
2.57552882987259	1.57552882987259	6.16049133059375	3.89745692475995
7.62181260562181	2.52378052266128	4.79679901706769	5.84570861754864
4.34449813264216	2.80392975127945	5.64827888081926	5.78744162849088
2.30536045275894	2.89032295348009	5.54057691445297	5.30536045275894
5.75110130714213	2.26997461740552	5.32886830645909	5.14444373532166
4.9933117103592	3.82338670891689	5.52382642705798	5.28281832755419
2.77147802135705	0.771478021357053	6.48572353902318	6.25529379862131
3.41940180682104	2.24947680537873	6.65035624166092	6.13211985474057
6.03463931304914	4.44967681232799	5.86471431160683	6.03463931304914
4.53500035233544	1.0495735251652	6.9273177751142	6.23544007047654

COL2A1-mRNA	IL10-mRNA	AR-mRNA	RAD50-mRNA
46.6613636804171	6.66590909720244	39.9954545832146	56.6602273262207
64.2304510069908	2.58398366120078	44.6660032864707	60.9081862997327
5.9608944592789	2.98044722963945	71.5307335113468	14.9022361481972
196.967338060108	5.75087118423672	27.7958773904775	57.5087118423672
20.3153474331313	6.98340068013887	50.1535139755428	55.2323508338256
4.94290936439841	7.41436404659761	46.545729848085	39.5432749151873
53.8584687160831	4.82314645218654	40.1928871015545	35.369740649368
31.8519923331366	14.1564410369496	46.0084333700862	38.9302128516114
6.82807081734685	1.70701770433671	89.6184294776774	76.3890422690679
10.6989833357119	4.75510370476085	100.451565763073	70.1377796452226
65.5552452144987	21.8517484048329	58.2713290795544	65.5552452144987
23.1830801346566	2.06991786916577	121.711170706947	75.345010437634

IGFBP3-mRNA	JAG1-mRNA	OSM-mRNA	JAK2-mRNA
7.43724136173545	6.05872973848172	5.19623326223166	8.49168914575783
8.33373149519189	6.9065379336071	0.147204526412445	7.41711040895152
8.56421351664476	6.27596854801368	3.57552882987259	8.73540016665098
11.8641870135146	7.86065895901461	3.02628086319046	8.34608578618485
9.89332004110091	7.37792113417961	1.34449813264216	8.31028241730424
9.26729241192542	7.22024383912099	2.52775287409539	8.67168266700475
10.4507126043355	6.97963286557599	3.59190271229288	8.62165005568693
9.0132112677969	7.18093871353497	5.63074163097449	7.57827421108036
8.20610624899378	7.27132390844026	2.09340611624442	8.53966234613398
9.95510919274015	7.87152862483511	2.57140490026609	8.24383024223759
7.90910843096528	6.44967681232799	5.44967681232799	8.22226631622491
9.26194831273247	7.89506357610958	2.0495735251652	7.88751676705623

IGFBP3-mRNA	JAG1-mRNA	OSM-mRNA	JAK2-mRNA
173.313636527263	66.6590909720244	36.6625000346134	359.959091248932
322.628817127069	119.970669984322	1.10742156908605	170.91206216228
378.51679816421	77.4916279706257	11.9217889185578	426.203953838441
3728.00224518145	232.431043696234	8.14706751100202	325.403461174728
951.012201713457	166.331907108762	2.53941842914141	317.427303642676
616.216034095001	149.111099159352	5.76672759179814	407.790022562868
1399.51632887613	126.205665498881	12.0578661304664	393.890293595234
516.71009784866	145.103520628733	49.5475436293236	191.11195399882
295.314062850252	154.485102242473	4.26754426084178	372.129859545404
992.627898368828	234.188857459472	5.94387963095106	303.137861178504
240.369232453162	87.4069936193316	43.7034968096658	298.640561532716
613.937639994567	238.040554954063	4.13983573833154	236.798604232564

TLR2-mRNA	IL3-mRNA	BRCA1-mRNA	HSP90B1-mRNA
6.98472915703794	4.32176414431551	5.64369223920288	6.64369223920288
5.33042635046821	0.884170120578651	5.20609821546601	8.5636502200841
7.24795417184409	4.57552882987259	5.82345634331618	6.38288375193019
6.09868935871851	1.93881802194012	5.02628086319046	8.45845427478334
6.15185305469976	2.80392975127945	5.25138872825068	7.64370615102944
6.46186493843893	2.17982957067508	5.65113528960067	7.66876518362246
7.22417092779239	3.00694021157172	5.29972196079957	7.16074554765076
7.55130716348009	5.40834920963804	5.40834920963804	8.53763222658301
5.76016270812922	0.356440522078209	5.31063683246508	7.341333629688
6.19199131071797	1.57140490026609	4.77303876143574	7.99094379177988
8.22226631622491	6.18664240649419	5.44967681232799	5.44967681232799
6.29750103860879	1.312607930999	4.83616988705601	7.35700205035745

TLR2-mRNA	IL3-mRNA	BRCA1-mRNA	HSP90B1-mRNA
126.652272846846	19.9977272916073	49.9943182290183	99.9886364580366
40.2363170101264	1.84570261514342	36.9140523028683	378.3690361044
152.002808711612	23.8435778371156	56.6284973631495	83.4525224299046
68.5312149454876	3.83391412282448	32.5882700440081	351.761620769146
71.1037160159594	6.98340068013887	38.0912764371211	199.979201294886
88.1485503317716	4.53100025069854	50.2529118713838	203.483102167734
149.517540017783	8.03857742031091	39.3890293595235	143.086678081534
187.572843739582	42.4693231108488	42.4693231108488	371.606577219927
54.1978121126906	1.28026327825253	39.6881616258286	162.166681911988
73.1097194606981	2.97193981547553	27.3418463023749	254.398048204705
298.640561532716	72.8391613494429	43.7034968096658	43.7034968096658
78.6568790282992	2.48390144299892	28.5648665944876	163.937495237929

BRCA2-mRNA	CDKN1C-mRNA	ETV4-mRNA	TRAF7-mRNA
4.32176414431551	5.64369223920288	4.90672664503667	8.87635299599315
3.46913262129981	6.78624369988939	2.14720452641244	8.5873815879698
4.57552882987259	5.89745692475995	3.16049133059375	9.42101888081697
4.72017773546478	6.96672401851001	3.10874302338244	8.92323648074126
4.38889225200061	6.72820242511621	3.25138872825068	8.80802250591334
4.80786079328812	6.50175766556244	1.04232604692514	8.84063582937974
4.38545183482545	6.32886830645909	2.00694021157172	8.75915357943687
5.40834920963804	6.40834920963804	4.9933117103592	8.48159819166868
3.57883294341466	7.85894086260739	2.77147802135705	8.03122128504783
4.24947680537873	7.94991652351982	3.05683172743634	8.13211985474057
4.44967681232799	6.56515402974792	4.44967681232799	7.77160490721535
2.89757043172015	7.17472865648749	2.42808514841893	7.4589644613029

BRCA2-mRNA	CDKN1C-mRNA	ETV4-mRNA	TRAF7-mRNA
19.9977272916073	49.9943182290183	29.996590937411	469.946591352772
11.0742156908605	110.373016385576	4.4296862763442	384.644424995888
23.8435778371156	59.608944592789	8.94134168891835	685.502862817074
26.3581595944183	125.081448257149	8.62630677635508	485.469375802649
20.9502020404166	106.020719416654	9.52281910928028	448.207352743459
28.009819731591	90.6200050139708	2.05954556849934	458.454843547953
20.9003012928084	80.3857742031091	4.01928871015545	433.279322954758
42.4693231108488	84.9386462216976	31.8519923331366	357.450136182977
11.949123930357	232.154407789793	6.82807081734685	261.600463189601
19.0204148190434	247.265392647564	8.32143148333149	280.55111858089
21.8517484048329	94.6909097542759	21.8517484048329	218.517484048329
7.45170432899677	144.480267267771	5.381786459831	175.94301887909

PRLR-mRNA	TFDP1-mRNA	LEP-mRNA	HSPA1A-mRNA
5.32176414431551	7.8242644848447	4.05872973848172	13.6361585665175
2.95455944847005	7.50475653103053	0.884170120578651	6.84301279582189
3.16049133059375	7.72527594937727	1.57552882987259	9.16049133059375
4.18674553538371	8.20326062216672	1.52378052266128	8.66673847650332
2.80392975127945	7.71953756398908	-0.655501867357843	8.63221051219161
2.6272885476463	7.69481254184331	1.04232604692514	8.8637811660276
2.85493711812667	8.92580344884632	2.26997461740552	9.69484073405247
5.28281832755419	6.3469486649739	5.63074163097449	12.7999508323322
5.27927266155575	7.83756721181483	1.57883294341466	8.3658026252819
6.27184461840719	7.95683593745961	2.24947680537873	8.27461636765724
5.67206923366443	6.03463931304914	5.18664240649419	10.1684950597839
1.53500035233544	7.87230367310972	1.0495735251652	7.69631222347305

PRLR-mRNA	TFDP1-mRNA	LEP-mRNA	HSPA1A-mRNA
39.9954545832146	226.640909304883	16.6647727430061	12731.8863756567
7.75195098360235	181.617137330112	1.84570261514342	114.80270266192
8.94134168891835	211.611753304401	2.98044722963945	572.245868090774
18.2110920834163	294.732148192132	2.87543559211836	406.394897019395
6.98340068013887	210.771729618737	0.634854607285352	396.784129553345
6.17863670549801	207.190284191033	2.05954556849934	465.869207594549
7.23471967827982	486.33393392881	4.82314645218654	828.777332034055
38.9302128516114	81.3995359624602	49.5475436293236	7131.30717236336
38.8346527736602	228.740372381119	2.98728098258925	329.88117136307
77.2704352023638	248.454168573755	4.75510370476085	309.67612877255
50.9874129446101	65.5552452144987	36.4195806747215	1150.8587493212
2.89788501683208	234.314702789565	2.06991786916577	207.40577049041

UBE2T-mRNA	IL23A-mRNA	TGFB3-mRNA	EYA1-mRNA
5.54415656565196	5.05872973848172	7.19623326223166	7.54415656565196
4.26268174383238	1.36959694774889	7.69666834591222	6.11683087736893
4.16049133059375	4.38288375193019	9.1902386739878	5.27596854801368
4.06810103888509	1.93881802194012	8.7843080728845	6.51624684997587
4.34449813264216	2.51442313408447	8.09604219173126	7.83233816646521
3.04232604692514	0.720397952037781	8.02189414702033	7.57526633529802
2.85493711812667	3.14444373532166	8.0730294020295	7.53050216762874
5.14531480380425	4.40834920963804	6.40834920963804	6.21570413169565
2.67836861696557	1.77147802135705	6.76016270812922	7.70221535891994
2.57140490026609	1.83443930609989	7.93247138906041	7.99094379177988
4.86471431160683	4.86471431160683	6.32414593024413	6.86471431160683
2.63453602588636	1.89757043172015	7.8230424530704	6.67015993561708

UBE2T-mRNA	IL23A-mRNA	TGFB3-mRNA	EYA1-mRNA
46.6613636804171	33.3295454860122	146.650000138454	186.645454721668
19.1953071974915	2.58398366120078	207.45697394212	69.3984183293924
17.8826833778367	20.8631306074761	584.167657009333	38.7458139853129
16.7733742873571	3.83391412282448	440.900124124816	91.5346996824345
20.3153474331313	5.71369146556817	273.622335739987	227.912804015441
8.23818227399735	1.64763645479947	259.914650744616	190.713919643038
7.23471967827982	8.842435162342	269.292343580416	184.887280667151
35.391102592374	21.2346615554244	84.9386462216976	74.3213154439854
6.40131639126267	3.41403540867343	108.395624225381	208.256159929079
5.94387963095106	3.56632777857064	244.293452832089	254.398048204705
29.1356645397772	29.1356645397772	80.1230774843872	116.542658159109
6.20975360749731	3.72585216449838	226.449014886735	101.839959162956

IL1A-mRNA	SMC1A-mRNA	CACNB2-mRNA	IL6R-mRNA
4.54415656565196	6.78119576295281	5.64369223920288	4.73680164359436
1.36959694774889	6.95455944847005	3.64970486694163	3.60663614504974
4.89745692475995	6.89745692475995	3.16049133059375	4.7454538313149
1.74617294399773	7.55352786605533	4.72017773546478	4.22422024080237
0.929460633363313	7.94068788878657	4.62990035150441	4.70205013726024
0.720397952037781	7.87521606108989	3.72039795203778	4.55328796620252
2.00694021157172	7.32163673722801	3.49236703874197	4.26997461740552
5.40834920963804	6.28281832755419	5.82338670891689	5.14531480380425
-1.22852197864295	7.42968950410885	4.38618786547226	4.92122514086174
0.834439306099887	7.29932535482929	5.22675672887865	4.94991652351982
5.44967681232799	6.18664240649419	6.18664240649419	5.67206923366443
1.312607930999	6.68184174066471	4.93709879590679	3.312607930999

IL1A-mRNA	SMC1A-mRNA	CACNB2-mRNA	IL6R-mRNA
23.3306818402085	109.98750010384	49.9943182290183	26.6636363888098
2.58398366120078	124.031215737637	12.5507777829752	12.1816372599465
29.8044722963945	119.217889185578	8.94134168891835	26.824025066755
3.35467485747142	187.861792018399	26.3581595944183	18.6903313487693
1.90456382185606	245.688733019431	24.7593296841287	26.0290388986994
1.64763645479947	234.788194808924	13.1810916383958	23.4788194808924
4.01928871015545	159.967690664187	11.2540083884353	19.2925858087462
42.4693231108488	77.8604257032228	56.6257641477984	35.391102592374
0.426754426084178	172.408788138008	20.9109668781247	30.2995642519767
1.78316388928532	157.512810220203	37.4464416749917	30.9081740809455
43.7034968096658	72.8391613494429	72.8391613494429	50.9874129446101
2.48390144299892	102.667926310622	30.6347844636534	9.93560577199569

NCOR1-mRNA	BMP8A-mRNA	TNN-mRNA	CACNA1G-mRNA
8.12911906637312	5.64369223920288	6.12911906637312	6.54415656565196
8.24874255287451	8.85456365849333	5.14720452641244	6.42642817034557
8.93308083449067	5.48241942548111	6.38288375193019	9.33041633203606
8.35667053682602	7.64617715402101	3.69370552410359	4.74617294399773
8.90864762262789	6.42131372969299	2.66642622752952	6.57331682313804
8.99069327850982	7.22819259223648	1.72039795203778	6.70339152673209
8.63920842707124	6.60387535395896	3.49236703874197	8.01592899479898
8.36254552002492	5.9933117103592	5.28281832755419	4.14531480380425
8.85761424638436	7.43325611912904	3.01940553480064	5.97115036619342
8.68201870576699	6.27184461840719	4.05683172743634	6.3787598223237
8.29097906630893	6.18664240649419	6.03463931304914	6.44967681232799
8.03598446041725	9.25022696153267	3.63453602588636	5.89757043172015

NCOR1-mRNA	BMP8A-mRNA	TNN-mRNA	CACNA1G-mRNA
279.968182082502	49.9943182290183	69.9920455206256	93.3227273608341
304.171790975635	462.902215877969	35.4374902107536	86.0097418656832
488.79334566087	44.7067084445918	83.4525224299046	643.776601602121
327.799657501493	200.322012917579	12.9394601645326	26.8373988597714
480.584937715012	85.7053719835225	6.34854607285352	95.2281910928028
508.707755419336	149.934917386752	3.29527290959894	104.213005766066
398.713440047421	97.266786785762	11.2540083884353	258.842192934012
329.137254109078	63.7039846662732	38.9302128516114	17.695551296187
463.882061153502	172.835542564092	8.10833409559939	62.7329006343742
410.722082498718	77.2704352023638	16.642862966663	83.2143148333149
313.208393802605	72.8391613494429	65.5552452144987	87.4069936193316
262.465585810219	608.96983710857	12.4195072149946	59.6136346319741

MYCN-mRNA	HPGD-mRNA	CDK2-mRNA	GZMB-mRNA
5.19623326223166	5.54415656565196	6.05872973848172	4.90672664503667
2.26268174383238	0.147204526412445	5.40773207663566	1.88417012057865
3.89745692475995	3.16049133059375	6.33041633203606	1.57552882987259
3.33113544471888	0.938818021940123	5.84570861754864	3.63925774008121
4.04493785078325	2.66642622752952	5.53432269152217	4.04493785078325
3.04232604692514	-0.279602047962219	5.52775287409538	3.72039795203778
2.26997461740552	2.68501211668436	5.81429513362933	4.14444373532166
5.63074163097449	5.40834920963804	4.9933117103592	4.9933117103592
2.35644052207821	2.35644052207821	4.72567433174393	4.0568802402193
3.24947680537873	2.41940180682104	5.29387092473718	3.24947680537873
4.44967681232799	5.18664240649419	4.86471431160683	5.67206923366443
1.72764543027784	-1.27235456972216	3.97557294372143	3.0495735251652

MYCN-mRNA	HPGD-mRNA	CDK2-mRNA	GZMB-mRNA
36.6625000346134	46.6613636804171	66.6590909720244	29.996590937411
4.79882679937288	1.10742156908605	42.4511601482985	3.69140523028683
14.9022361481972	8.94134168891835	80.4720752002651	2.98044722963945
10.0640245724143	1.91695706141224	57.5087118423672	12.4602208991796
16.5062197894191	6.34854607285352	46.3443863318307	16.5062197894191
8.23818227399735	0.823818227399735	46.1338207343851	13.1810916383958
4.82314645218654	6.43086193624873	56.2700419421764	17.684870324684
49.5475436293236	42.4693231108488	31.8519923331366	31.8519923331366
5.12105311301014	5.12105311301014	26.458774417219	16.643422617283
9.5102074095217	5.34949166785596	39.229605564277	9.5102074095217
21.8517484048329	36.4195806747215	29.1356645397772	50.9874129446101
3.31186859066523	0.413983573833154	15.7313758056598	8.27967147666308

NOTCH3-mRNA	LIG4-mRNA	ATR-mRNA	WNT4-mRNA
8.35151148770957	6.19623326223166	7.26036359965137	1.73680164359436
8.95025931103642	5.34360173921595	6.6811830984148	2.14720452641244
8.31699581627374	5.38288375193019	7.40841884403733	3.16049133059375
9.95701820075335	6.03685010490065	7.61829812144557	5.39824964057742
9.10272134736888	6.12585784616682	7.44778594105418	3.73681555542092
9.98776988330306	6.4346434697039	7.39282329400928	3.72039795203778
8.77512453634865	5.93293963012795	7.32163673722801	4.38545183482545
8.09017324961179	6.07131422236047	6.28281832755419	5.14531480380425
9.06954058907607	6.00988276068213	7.16808280253891	5.45797854854027
10.1623661416087	5.77303876143574	7.01102803782321	4.94991652351982
7.25703173438559	7.25703173438559	7.50857050138155	5.44967681232799
9.89882222792961	5.26680424138587	6.08519743489592	3.53500035233544

NOTCH3-mRNA	LIG4-mRNA	ATR-mRNA	WNT4-mRNA
326.629545762919	73.3250000692268	153.315909235656	3.33295454860122
494.648300858436	40.6054575331551	102.621065401974	4.4296862763442
318.907853571421	41.7262612149523	169.885492089449	8.94134168891835
993.942236342247	65.6557793533692	196.488098794755	42.1730553510693
549.784089909115	69.8340068013888	174.585017003472	13.3319467529924
1015.35596527017	86.5009138769721	168.058918389546	13.1810916383958
438.102469406944	61.0931883943629	159.967690664187	20.9003012928084
272.51148996128	67.2430949255106	77.8604257032228	35.391102592374
537.28382243998	64.4399183387109	143.816241590368	43.9557058866704
1145.97999284737	54.6836926047498	128.982187991638	30.9081740809455
152.96223883383	152.96223883383	182.097903373607	43.7034968096658
954.646121259252	38.5004723664833	67.8933061086372	11.5915400673283

CRLF2-mRNA	RELN-mRNA	CCNO-mRNA	JAG2-mRNA
2.73680164359436	4.90672664503667	3.32176414431551	7.49168914575783
0.562242025691289	4.95455944847005	0.884170120578651	7.6417268095181
2.57552882987259	4.16049133059375	3.57552882987259	6.52972514025947
2.26074611682749	6.81533496850512	1.93881802194012	8.25849014288712
3.04493785078325	5.20247912776973	2.34449813264216	7.09938563480562
2.6272885476463	1.89032295348009	1.72039795203778	6.82892240881595
3.14444373532166	5.85493711812667	3.00694021157172	7.14444373532166
2.82338670891689	5.28281832755419	4.63074163097449	6.07131422236047
1.77147802135705	3.72567433174393	1.77147802135705	7.57883294341466
1.57140490026609	4.0043643075422	1.57140490026609	7.7453318322659
5.44967681232799	6.56515402974792	5.67206923366443	6.56515402974792
2.81510827152818	3.0495735251652	1.89757043172015	6.44189094794396

CRLF2-mRNA	RELN-mRNA	CCNO-mRNA	JAG2-mRNA
6.66590909720244	29.996590937411	9.99886364580366	179.979545624466
1.47656209211473	31.0078039344094	1.84570261514342	199.705022958518
5.9608944592789	17.8826833778367	11.9217889185578	92.3938641188229
4.7923926535306	112.621227357969	3.83391412282448	306.233890560606
8.25310989470958	36.8215672225504	5.07883685828282	137.128595173636
6.17863670549801	3.70718202329881	3.29527290959894	113.686915381163
8.842435162342	57.8777574262385	8.03857742031091	141.478962597472
7.0782205184748	38.9302128516114	24.7737718146618	67.2430949255106
3.41403540867343	13.2293872086095	3.41403540867343	191.185982885712
2.97193981547553	16.0484750035679	2.97193981547553	214.574054677333
43.7034968096658	94.6909097542759	50.9874129446101	94.6909097542759
7.03772075516361	8.27967147666308	3.72585216449838	86.9365505049623

BAIAP3-mRNA	PPP2R2C-mRNA	AMH-mRNA	DTX4-mRNA
4.54415656565196	5.54415656565196	5.19623326223166	6.86608466053932
1.14720452641244	0.147204526412445	2.46913262129981	6.43875897225629
1.57552882987259	3.16049133059375	6.03496044850989	6.38288375193019
6.63230497943945	1.26074611682749	2.84570861754864	6.87545596094269
6.88365694375019	0.929460633363313	2.92946063336331	6.00270961539395
4.92985131766673	2.17982957067508	1.72039795203778	5.27498680371542
3.14444373532166	2.85493711812667	3.85493711812667	6.06005154803129
5.52382642705798	5.52382642705798	5.91084955016723	6.14531480380425
7.610681809454	0.771478021357053	2.77147802135705	6.65412107071889
5.24947680537873	1.24947680537873	2.41940180682104	5.65886774151643
7.77160490721535	5.86471431160683	4.86471431160683	6.77160490721535
2.89757043172015	-1.27235456972216	3.1199628530566	5.78292786577903

BAIAP3-mRNA	PPP2R2C-mRNA	AMH-mRNA	DTX4-mRNA
23.3306818402085	46.6613636804171	36.6625000346134	116.653409201043
2.2148431381721	1.10742156908605	5.53710784543025	86.7480229117405
2.98044722963945	8.94134168891835	65.5698390520679	83.4525224299046
99.2025279280834	2.3961963267653	7.1885889802959	117.4136200115
118.082956955075	1.90456382185606	7.61825528742422	64.1203153358206
30.4812744137902	4.53100025069854	3.29527290959894	38.7194566877875
8.842435162342	7.23471967827982	14.4694393565596	66.7201925885805
46.0084333700862	46.0084333700862	60.1648744070358	70.782205184748
195.453527146554	1.70701770433671	6.82807081734685	100.714044555866
38.0408296380868	2.37755185238043	5.34949166785596	50.522976863084
218.517484048329	58.2713290795544	29.1356645397772	109.258742024164
7.45170432899677	0.413983573833154	8.69365505049623	55.0598153198095

RB1-mRNA	CCNA2-mRNA	CREB3L3-mRNA	WNT5B-mRNA
6.26036359965137	5.8242644848447	4.05872973848172	5.43724136173545
7.27648754335741	4.56224202569129	-0.437757974308711	6.92418579942653
5.82345634331618	5.03496044850989	3.57552882987259	5.48241942548111
7.14827138756907	4.79679901706769	0.523780522661279	5.88133252727936
7.11928519224333	5.04493785078325	1.92946063336331	5.51442313408447
7.23215060580516	5.29025356036873	0.720397952037781	6.03328090732214
6.5179021308491	5.14444373532166	0.685012116684361	6.00694021157172
6.3469486649739	4.82338670891689	3.82338670891689	5.91084955016723
7.03826456205196	4.52636552352052	0.356440522078209	6.00988276068213
7.2381614921509	4.29387092473718	-0.750523194621269	5.75727144557743
6.77160490721535	6.95217715285717	2.86471431160683	6.32414593024413
6.93709879590679	4.63453602588636	-1.27235456972216	6.2114612075421

RB1-mRNA	CCNA2-mRNA	CREB3L3-mRNA	WNT5B-mRNA
76.6579546178281	56.6602273262207	16.6647727430061	43.3284091318159
155.039019672047	23.6249934738357	0.738281046057366	121.447232076437
56.6284973631495	32.784919526034	11.9217889185578	44.7067084445918
141.854822544506	27.7958773904775	1.43771779605918	58.9464296384264
139.033158995492	33.0124395788383	3.80912764371211	45.7095317245453
150.346826500452	39.1313658014874	1.64763645479947	65.4935490782789
91.6397825915443	35.369740649368	1.60771548406218	64.3086193624873
81.3995359624602	28.3128820738992	14.1564410369496	60.1648744070358
131.440363233927	23.0447390085456	1.28026327825253	64.4399183387109
150.974542626157	19.6148027821385	0.594387963095106	54.0893046416547
109.258742024164	123.826574294053	7.2839161349443	80.1230774843872
122.539137854613	24.8390144299892	0.413983573833154	74.1030597161345

LEFTY2-mRNA	BMP2-mRNA	EZH2-mRNA	PBRM1-mRNA
6.26036359965137	5.43724136173545	6.32176414431551	6.05872973848172
2.64970486694163	7.81727059551002	5.0858039817483	6.52224395775937
6.16049133059375	5.03496044850989	4.57552882987259	5.16049133059375
2.52378052266128	8.02893044160441	5.49340687361776	6.20560456263502
5.53432269152217	6.36686594567061	5.64827888081926	5.70205013726024
3.96832546548137	5.47528545420125	5.00580017090003	5.33510779615299
3.85493711812667	7.06871640915841	5.14444373532166	5.20857407274137
6.86778082827534	10.3741334943001	7.63074163097449	6.3469486649739
4.01940553480064	5.74875794485697	4.92122514086174	6.45797854854027
4.45893017100768	6.27184461840719	3.77303876143574	6.64179422815749
6.67206923366443	6.32414593024413	5.67206923366443	5.18664240649419
1.0495735251652	6.95164710447594	3.93709879590679	6.57313548122221

LEFTY2-mRNA	BMP2-mRNA	EZH2-mRNA	PBRM1-mRNA
76.6579546178281	43.3284091318159	79.9909091664293	66.6590909720244
6.27538889148761	225.544859570525	33.9609281186388	91.9159902341421
71.5307335113468	32.784919526034	23.8435778371156	35.7653667556734
5.75087118423672	261.185399617418	45.0484909431877	73.8028468643712
46.3443863318307	82.5310989470958	50.1535139755428	52.0580777973989
15.652546320595	44.4861842795857	32.1289108685897	40.367093142587
14.4694393565596	134.244242919192	35.369740649368	36.9774561334302
116.790638554834	1327.16634721403	198.190174517294	81.3995359624602
16.2166681911988	53.7710576866065	30.2995642519767	87.9114117733407
21.9923546345189	77.2704352023638	13.6709231511874	99.8571777999779
101.97482588922	80.1230774843872	50.9874129446101	36.4195806747215
2.06991786916577	123.781088576113	15.3173922318267	95.2162219816253

HDAC4-mRNA	CD14-mRNA	NGF-mRNA	AKT1-mRNA
5.64369223920288	7.26036359965137	4.32176414431551	8.81361724064519
6.63905762274212	7.42952076540095	4.0858039817483	9.5720706430594
5.89745692475995	6.0990907859296	3.57552882987259	9.21938501964732
6.07836937433892	8.09868935871851	5.92750270871229	9.76298123168428
6.62990035150441	7.0239782321476	3.59242564608574	8.90674055686323
6.51481381838789	5.93956647249994	5.4346434697039	9.07684992225954
6.34322359943616	7.42647910308551	3.00694021157172	9.16882789394862
5.14531480380425	7.55130716348009	3.40834920963804	8.91084955016723
6.19774277605915	6.17235745763924	3.67836861696557	8.95014287236353
7.05683172743634	7.32093916793535	4.80406565705637	9.23247038007304
6.32414593024413	5.67206923366443	4.86471431160683	7.77160490721535
6.39298134746302	7.52206129662795	3.97557294372143	8.86591723045006

HDAC4-mRNA	CD14-mRNA	NGF-mRNA	AKT1-mRNA
49.9943182290183	153.315909235656	19.9977272916073	449.948864061165
99.6679412177445	172.388624254395	16.9804640593194	761.167758485145
59.608944592789	68.5502862817074	11.9217889185578	596.08944592789
67.5727364147815	274.12485978195	60.8633866998386	868.860788085099
99.0373187365149	130.145194493497	12.0622375384217	479.950083107726
91.4438232413706	61.3744579412802	43.2504569384861	540.012848060527
81.1896319451402	172.025556794654	8.03857742031091	575.562143294261
35.391102592374	187.572843739582	10.6173307777122	481.318995256287
73.4017612864787	72.1214980082261	12.8026327825253	494.608379831563
133.142903733304	159.890362072584	27.93623426547	601.520618652248
80.1230774843872	50.9874129446101	29.1356645397772	218.517484048329
84.0386654881302	183.80870678192	15.7313758056598	466.559487709965

IFNA2-mRNA	TET2-mRNA	CACNA2D2-mRNA	DNMT1-mRNA
5.43724136173545	6.54415656565196	1.73680164359436	6.73680164359436
1.14720452641244	7.05409512202096	3.0858039817483	6.64439106704516
3.16049133059375	7.13011768155023	5.03496044850989	6.82345634331618
1.26074611682749	6.68028500834127	3.18674553538371	6.97224102347757
3.59242564608574	7.26931063624794	3.73681555542092	6.72820242511621
1.30536045275894	7.0104167989704	2.72039795203778	6.71475138889664
2.49236703874197	6.19280675688306	4.32886830645909	6.5676551660462
4.40834920963804	6.07131422236047	2.82338670891689	6.73027730452541
0.356440522078209	7.59165698377224	3.47191773949814	6.71399252669629
0.834439306099887	7.0242638649799	4.1074578005063	6.33693964662907
5.67206923366443	6.56515402974792	4.44967681232799	6.77160490721535
1.0495735251652	6.72764543027784	2.0495735251652	6.18707704891514

IFNA2-mRNA	TET2-mRNA	CACNA2D2-mRNA	DNMT1-mRNA
43.3284091318159	93.3227273608341	3.33295454860122	106.654545555239
2.2148431381721	132.890588290326	8.49023202965971	100.037081740773
8.94134168891835	140.081019793054	32.784919526034	113.256994726299
2.3961963267653	102.557202785555	9.10554604170814	125.560687522502
12.0622375384217	154.269669570341	13.3319467529924	106.020719416654
2.4714546821992	128.927552588058	6.59054581919788	105.036823993466
5.62700419421764	73.1510545248293	20.0964435507773	94.8552135596687
21.2346615554244	67.2430949255106	7.0782205184748	106.173307777122
1.28026327825253	192.893000590049	11.0956150781886	104.981588816708
1.78316388928532	130.170963917828	17.2372509297581	80.8367629809345
50.9874129446101	94.6909097542759	21.8517484048329	109.258742024164
2.06991786916577	105.979794901287	4.13983573833154	72.8611089946351

PRKAR2A-mRNA	KRAS-mRNA	EFNA5-mRNA	CACNB4-mRNA
7.16306639829646	5.90672664503667	5.54415656565196	5.05872973848172
8.04202228972039	7.10913648557893	4.02167364432859	3.46913262129981
7.57552882987259	5.57552882987259	5.89745692475995	4.38288375193019
8.31168308205271	6.68028500834127	4.96118583496858	3.46237997799714
7.41596049519878	5.77076288734425	4.62990035150441	4.25138872825068
7.63328728826774	5.36425414181251	6.06024795492241	2.96832546548137
7.69063666587824	5.41293257124756	6.25486772501531	3.59190271229288
7.68136770404446	6.14531480380425	5.40834920963804	4.9933117103592
7.51967087094651	6.08436097664141	5.78270527678031	3.62945901648462
7.65461826851508	6.36841787810224	6.15636740098725	3.49740431882232
7.50857050138155	6.32414593024413	5.18664240649419	5.67206923366443
7.34969724973422	6.73326997947172	7.32010246754592	2.42808514841893

PRKAR2A-mRNA	KRAS-mRNA	EFNA5-mRNA	CACNB4-mRNA
143.317045589852	59.9931818748219	46.6613636804171	33.3295454860122
263.56633344248	138.058555612728	16.2421830132621	11.0742156908605
190.748622696925	47.6871556742312	59.608944592789	20.8631306074761
317.735632929079	102.557202785555	31.1505522479489	11.0225031031204
170.77588935976	54.5974962265403	24.7593296841287	19.0456382185606
198.540192803336	41.1909113699867	66.7292764193785	7.82627316029748
206.59143970199	42.6044603276478	76.3664854929536	12.0578661304664
205.268395035769	70.782205184748	42.4693231108488	31.8519923331366
183.504403216197	67.8539537473844	55.051320964859	12.3758783564412
201.497519489241	82.6199268702198	71.3265555714128	11.293371298807
182.097903373607	80.1230774843872	36.4195806747215	50.9874129446101
163.109528090263	106.393778475121	159.797659499597	5.381786459831

CACNA2D4-mRNA	PIK3CG-mRNA	WNT7B-mRNA	E2F1-mRNA
4.73680164359436	6.43724136173545	4.90672664503667	4.32176414431551
0.884170120578651	4.49297936325418	6.52224395775937	3.60663614504974
2.57552882987259	3.89745692475995	3.89745692475995	3.16049133059375
2.10874302338244	5.14827138756907	5.38176151778885	3.18674553538371
2.80392975127945	5.12585784616682	4.04493785078325	3.15185305469976
2.04232604692514	5.34975457211739	0.720397952037781	3.17982957067508
3.14444373532166	5.32886830645909	4.20857407274137	2.85493711812667
5.40834920963804	6.28281832755419	4.40834920963804	4.40834920963804
-1.22852197864295	5.0568802402193	5.48572353902318	1.57883294341466
0.834439306099887	5.29387092473718	4.05683172743634	2.24947680537873
5.18664240649419	6.32414593024413	6.03463931304914	5.18664240649419
1.0495735251652	4.79373462073561	3.312607930999	1.72764543027784

CACNA2D4-mRNA	PIK3CG-mRNA	WNT7B-mRNA	E2F1-mRNA
26.6636363888098	86.6568182636317	29.996590937411	19.9977272916073
1.84570261514342	22.5175719047497	91.9159902341421	12.1816372599465
5.9608944592789	14.9022361481972	14.9022361481972	8.94134168891835
4.31315338817754	35.4637056361264	41.6938160857162	9.10554604170814
6.98340068013887	34.9170034006944	16.5062197894191	8.88796450199493
4.11909113699867	40.7790022562869	1.64763645479947	9.06200050139708
8.842435162342	40.1928871015545	18.4887280667151	7.23471967827982
42.4693231108488	77.8604257032228	21.2346615554244	21.2346615554244
0.426754426084178	33.2868452345659	44.8092147388387	2.98728098258925
1.78316388928532	39.229605564277	16.642862966663	4.75510370476085
36.4195806747215	80.1230774843872	65.5552452144987	36.4195806747215
2.06991786916577	27.7368994468213	9.93560577199569	3.31186859066523

PAK3-mRNA	MAP3K1-mRNA	FOXO4-mRNA	NOTCH1-mRNA
2.73680164359436	5.8242644848447	6.05872973848172	7.19623326223166
0.562242025691289	6.51643833607816	6.1769518698065	6.26960115777217
2.57552882987259	6.0990907859296	5.96784625265135	7.03496044850989
2.10874302338244	6.83363578524807	6.79056706335618	7.67352764216596
2.80392975127945	6.89139259252979	6.77076288734425	7.78744162849089
2.30536045275894	6.60914120093604	6.52129785195809	7.22819259223648
3.26997461740552	6.07732953946312	6.91383080718024	6.81429513362933
4.40834920963804	5.28281832755419	5.82338670891689	6.86778082827534
2.77147802135705	7.48572353902318	6.60436803552179	6.73147995342513
3.64179422815749	7.23247038007304	7.11984152496214	7.38902815777752
2.86471431160683	6.56515402974792	5.67206923366443	4.44967681232799
1.89757043172015	6.79910779283446	6.49582975505477	6.34969724973422

PAK3-mRNA	MAP3K1-mRNA	FOXO4-mRNA	NOTCH1-mRNA
6.66590909720244	56.6602273262207	66.6590909720244	146.650000138454
1.47656209211473	91.5468497111134	72.3515425136219	77.1503693129947
5.9608944592789	68.5502862817074	62.5893918224285	131.139678104136
4.31315338817754	114.058945154028	110.704270296557	204.155927040403
6.98340068013887	118.717811562361	109.194992453081	220.929403335303
4.94290936439841	97.6224599468686	91.8557323550704	149.934917386752
9.64629290437309	67.5240503306117	120.578661304664	112.540083884353
21.2346615554244	38.9302128516114	56.6257641477984	116.790638554834
6.82807081734685	179.236858955355	97.3000091471926	106.26185209496
12.4821472249972	150.380154663062	139.086783364255	167.61740559282
7.2839161349443	94.6909097542759	50.9874129446101	21.8517484048329
3.72585216449838	111.361581361118	90.2484190956275	81.5547640451313

PML-mRNA	COL5A1-mRNA	SMARCB1-mRNA	PLA2G4A-mRNA
9.77572063288666	9.10312385784017	9.05872973848172	5.32176414431551
8.57067064776187	13.0675573818275	7.83370505359566	4.34360173921595
9.67881663828461	9.79469735033475	9.01015705750931	3.89745692475995
9.02229734927197	11.6620523228335	9.37240346309062	5.893014332327
9.33318281941432	10.5386393715053	9.0604601228973	3.80392975127945
9.47026737943462	10.4698484176077	8.84322594684545	4.24395990809479
10.0046842376314	9.86616437354993	9.25865930417768	3.85493711812667
9.52382642705798	8.70602975827873	8.46724289869161	4.14531480380425
8.93386935011396	9.26733304824422	8.66781042526699	4.12903002597514
9.17725476746107	9.66732932026463	8.54179843818077	4.24947680537873
8.7226953067344	8.22226631622491	8.32414593024413	3.86471431160683
8.67015993561708	10.7727224822128	8.03370711970618	2.53500035233544

PML-mRNA	COL5A1-mRNA	SMARCB1-mRNA	PLA2G4A-mRNA
876.56704628212	549.937500519201	533.272727776195	39.9954545832146
380.214738719543	8584.73200355505	228.128843231726	20.3027287665776
819.622988150849	888.173274432556	515.617370727625	14.9022361481972
519.974602908071	3240.61591231739	662.787903983282	59.4256689037794
645.012281001918	1487.46434486958	533.912724726981	13.9668013602777
709.307493791171	1418.20307846864	459.278661775352	18.9478192301939
1027.33019431573	933.278838498097	612.539599427691	14.4694393565596
736.134933921379	417.615010590013	353.91102592374	17.695551296187
489.060572292469	616.233391265553	406.696968058222	17.4969314694513
578.933876054633	813.122733514105	372.681252860632	19.0204148190434
422.467135826769	298.640561532716	320.492309937549	14.5678322698886
407.359836651823	1749.49458301891	262.051602236387	5.79577003366415

**SIX1-mRNA**	**RET-mRNA**	**HSPB1-mRNA**	**RBX1-mRNA**
6.78119576295281	4.32176414431551	10.7647076401642	8.19623326223165
5.61752446119248	2.02167364432859	7.9175931221161	8.39986995886269
7.80434752036847	1.57552882987259	9.78498219550154	8.52972514025947
6.5312750592082	2.26074611682749	9.41455145290652	8.93033986801582
7.47892445286308	2.34449813264216	8.88365694375019	8.65511091430169
6.51481381838789	1.89032295348009	9.53658163347012	8.83022860631657
7.76182771373519	2.49236703874197	9.90175797487967	8.37501008810381
7.37797556059453	4.14531480380425	8.95266972586185	7.65627672308163
6.99064654181922	1.94140302279937	9.33562751134279	8.66022127025531
7.40429491443084	3.49740431882232	9.44053601991039	8.7413299017084
7.03463931304914	5.18664240649419	8.86471431160683	6.56515402974792
5.81510827152818	1.0495735251652	8.71916727635353	8.49251702101393

**SIX1-mRNA**	**RET-mRNA**	**HSPB1-mRNA**	**RBX1-mRNA**
109.98750010384	19.9977272916073	1739.80227436984	293.300000276907
49.0956895628149	4.06054575331551	241.787042583787	337.763578571245
223.533542222959	2.98044722963945	882.212379973277	369.575456475292
92.4931782131406	4.7923926535306	682.436713862758	487.865572129415
178.394144647184	5.07883685828282	472.331827820302	403.132675626199
91.4438232413706	3.70718202329881	742.672132000862	455.159570638353
217.041590348395	5.62700419421764	956.590713016999	331.993247458841
166.338182184158	17.695551296187	495.475436293236	201.729284776532
127.172818973085	3.8407898347576	646.106201091446	404.563195927801
169.400569482105	11.293371298807	694.839528858179	427.959333428476
131.110490428997	36.4195806747215	466.170632636435	94.6909097542759
56.3017660413089	2.06991786916577	421.43527816215	360.165709234844

SOCS1-mRNA	TSHR-mRNA	PLCB4-mRNA	ANGPT1-mRNA
4.73680164359436	1.73680164359436	7.05872973848172	6.43724136173545
5.1915986457709	-0.437757974308711	6.62293795737884	6.10913648557892
3.57552882987259	3.57552882987259	6.48241942548111	4.16049133059375
6.11872711195506	2.52378052266128	7.4023423952113	5.08856514144481
6.1773881468069	0.929460633363313	7.65738108792651	6.26336136991675
5.99652235731202	1.30536045275894	7.2940451395311	6.17982957067508
5.26997461740552	2.49236703874197	7.16882789394862	7.47942798303447
5.73027730452541	1.82338670891689	6.82338670891689	5.28281832755419
5.23090963999435	-0.228521978642947	7.65106127096984	6.62945901648462
4.94991652351982	0.249476805378731	7.78475218199953	8.75727144557743
4.86471431160683	4.44967681232799	7.18664240649419	5.18664240649419
5.08519743489592	0.312607930998996	5.61028847963968	7.07637358450892

SOCS1-mRNA	TSHR-mRNA	PLCB4-mRNA	ANGPT1-mRNA
26.6636363888098	3.33295454860122	133.318181944049	86.6568182636317
36.5449117798396	0.738281046057366	98.5605196486584	69.0292778063637
11.9217889185578	11.9217889185578	89.4134168891835	17.8826833778367
69.4896934761937	5.75087118423672	169.17146066963	34.0259878400673
72.3734252305301	1.90456382185606	201.883765116742	76.8174074815276
63.8459126234794	2.4714546821992	156.937372319649	72.4960040111767
38.5851716174924	5.62700419421764	143.890535823565	178.456418730902
53.086653888561	3.5391102592374	113.251528295597	38.9302128516114
37.5543894954077	0.853508852168356	201.001334685648	99.0070268515293
30.9081740809455	1.18877592619021	220.517934308284	432.714437133237
29.1356645397772	21.8517484048329	145.678322698886	36.4195806747215
33.9466530543186	1.24195072149946	48.8500617123122	134.958645069608

FZD2-mRNA	GNAQ-mRNA	BCL2-mRNA	COMP-mRNA
4.90672664503667	7.75916945662281	6.32176414431551	6.90672664503667
3.36959694774889	8.1695723394409	5.00518552154002	3.7321670271336
4.57552882987259	7.66299167112293	5.48241942548111	8.45817187923443
3.39824964057742	8.28089268993926	5.50867363027107	7.49340687361776
4.29869444302903	8.15827932385919	6.07241858720536	3.86806008869917
3.6272885476463	7.78917623002319	6.22819259223648	5.59076267162119
4.07732953946312	8.28492495887149	6.83475923618904	9.73486066613492
5.73027730452541	8.21570413169565	6.07131422236047	6.63074163097449
3.57883294341466	8.42252971253598	6.99547969555516	5.14651745270398
4.74132990170841	8.7453318322659	6.64179422815749	4.83443930609989
5.44967681232799	7.7226953067344	7.18664240649419	6.32414593024413
2.312607930999	8.52854533019815	6.34969724973422	6.19525098036084

FZD2-mRNA	GNAQ-mRNA	BCL2-mRNA	COMP-mRNA
29.996590937411	216.642045659079	79.9909091664293	119.986363749644
10.3359346448031	287.929607962373	32.1152255034954	13.2890588290326
23.8435778371156	202.670411615483	44.7067084445918	351.692773097455
10.5432638377673	311.026283214136	45.5277302085407	180.193963772751
19.6804928258459	285.684573278408	67.2945883722473	14.6016559675631
12.357273410996	221.195194056829	74.9674586933758	48.1933663028845
16.8810125826529	311.896803908063	114.147799368415	852.089206552957
53.086653888561	297.285261775942	67.2430949255106	99.0950872586472
11.949123930357	343.110558571679	127.599573399169	35.4206173649868
26.7474583392798	429.148109354666	99.8571777999779	28.5306222285651
43.7034968096658	211.233567913384	145.678322698886	80.1230774843872
4.96780288599784	369.273347859173	81.5547640451313	73.2750925684682

FLNA-mRNA	IL19-mRNA	RAD51-mRNA	PLA2G5-mRNA
8.02220386245661	1.73680164359436	1.73680164359436	4.73680164359436
10.1349422521786	-1.43775797430871	2.46913262129981	1.7321670271336
8.06738192620227	1.57552882987259	1.57552882987259	1.57552882987259
10.1628196961382	0.938818021940123	-1.06118197805988	1.93881802194012
9.29286536422683	0.344498132642157	-0.655501867357843	2.51442313408447
10.6314153598348	0.305360452758937	0.720397952037781	1.72039795203778
9.89690041123037	-0.314987883315639	-0.314987883315639	2.68501211668436
9.07131422236047	4.14531480380425	1.82338670891689	5.14531480380425
9.93512769737288	1.57883294341466	0.771478021357053	1.57883294341466
11.2648918577654	2.05683172743634	0.249476805378731	2.57140490026609
8.973238768385	5.18664240649419	3.86471431160683	4.86471431160683
10.8694332719488	-0.27235456972216	0.72764543027784	2.42808514841893

FLNA-mRNA	IL19-mRNA	RAD51-mRNA	PLA2G5-mRNA
259.970454790895	3.33295454860122	3.33295454860122	26.6636363888098
1124.40203314537	0.369140523028683	5.53710784543025	3.32226470725815
268.240250667551	2.98044722963945	2.98044722963945	2.98044722963945
1146.34032272452	1.91695706141224	0.47923926535306	3.83391412282448
627.236351997928	1.2697092145707	0.634854607285352	5.71369146556817
1586.26199685819	1.2357273410996	1.64763645479947	3.29527290959894
953.375282048874	0.803857742031091	0.803857742031091	6.43086193624873
537.944759404085	17.695551296187	3.5391102592374	35.391102592374
978.974653437104	2.98728098258925	1.70701770433671	2.98728098258925
2460.76616721374	4.16071574166575	1.18877592619021	5.94387963095106
502.590213311156	36.4195806747215	14.5678322698886	29.1356645397772
1870.79177015202	0.827967147666308	1.65593429533262	5.381786459831

PLA2G4C-mRNA	NR4A3-mRNA	CIC-mRNA	RELA-mRNA
5.43724136173545	7.5696916577591	8.17974513944309	6.69099795398123
5.53952194919121	3.64970486694163	7.68894649853448	7.40773207663566
5.38288375193019	7.86093104873484	9.5585224045669	6.96784625265135
5.0049072123979	4.4306711182698	8.35667053682602	7.60059611971211
4.83635122897183	7.65283716278156	8.17422086772821	6.57331682313804
4.96832546548137	6.82892240881595	8.07794995665586	6.69194150598855
4.81429513362933	8.79092062525552	8.55537683626777	7.44656334912884
4.9933117103592	7.60474642244155	8.12716745709399	7.97313382842157
4.70221535891994	6.59802650864797	7.67836861696557	7.07068603974433
5.15636740098725	8.8174328807942	7.75330254337448	7.40429491443084
5.44967681232799	7.81891062199371	7.56515402974792	6.56515402974792
4.87739254978252	6.09396764452366	7.47918948936694	7.52206129662795

PLA2G4C-mRNA	NR4A3-mRNA	CIC-mRNA	RELA-mRNA
43.3284091318159	189.978409270269	289.967045728306	103.321591006638
46.5117059016141	12.5507777829752	206.349552373034	169.804640593194
41.7262612149523	232.474883911877	754.053149098781	125.178783644857
32.109030778655	21.5657669408877	327.799657501493	194.091902467989
28.5684573278408	201.248910509457	288.858846314835	95.2281910928028
31.3050926411899	113.686915381163	270.212378587113	103.389187538667
28.1350209710882	442.925615859131	376.20542327055	174.437130020747
31.8519923331366	194.651064258057	279.589710479755	251.276828405855
26.0320199911349	96.8732547211085	204.842124520405	134.427644216516
35.6632777857064	451.140463989186	215.762830603524	169.400569482105
43.7034968096658	225.801400183273	189.381819508552	94.6909097542759
29.3928337421539	68.3072896824704	178.426920322089	183.80870678192

TCL1B-mRNA	SP1-mRNA	PLCE1-mRNA	ACVR1C-mRNA
1.73680164359436	6.49168914575783	5.73680164359436	4.54415656565196
-0.437757974308711	7.24874255287451	5.36959694774889	2.64970486694163
2.57552882987259	6.89745692475995	8.76535338875261	4.16049133059375
-0.061181978059877	7.04734247871829	5.70700234671705	1.74617294399773
0.929460633363313	6.89908698431979	6.35572538806541	2.34449813264216
-0.279602047962219	6.22024383912099	6.23609779032182	2.30536045275894
1.68501211668436	6.79353657346253	5.70737992971282	2.26997461740552
4.82338670891689	6.28281832755419	5.82338670891689	4.9933117103592
-0.228521978642947	7.08436097664141	6.79938401792694	2.23090963999435
0.834439306099887	6.97739725994193	6.57140490026609	2.24947680537873
2.86471431160683	6.03463931304914	6.56515402974792	5.44967681232799
-1.27235456972216	7.38585691302964	5.1199628530566	2.312607930999

TCL1B-mRNA	SP1-mRNA	PLCE1-mRNA	ACVR1C-mRNA
3.33295454860122	89.9897728122329	53.3272727776195	23.3306818402085
0.738281046057366	152.085895487817	41.3437385792125	6.27538889148761
5.9608944592789	119.217889185578	435.14529552736	17.8826833778367
0.95847853070612	132.270037237445	52.2370799234835	3.35467485747142
1.90456382185606	119.352666169646	81.8962443398104	5.07883685828282
0.823818227399735	74.555549579676	75.3793678070757	4.94290936439841
3.21543096812436	110.932368400291	52.2507532320209	4.82314645218654
28.3128820738992	77.8604257032228	56.6257641477984	31.8519923331366
0.853508852168356	135.707907494769	111.382905207971	4.69429868692596
1.78316388928532	126.010248176163	95.102074095217	4.75510370476085
7.2839161349443	65.5552452144987	94.6909097542759	43.7034968096658
0.413983573833154	167.249363828594	34.7746202019849	4.96780288599784

IL20RA-mRNA	SHC2-mRNA	IL5RA-mRNA	TGFB2-mRNA
5.54415656565196	6.43724136173545	5.19623326223166	3.73680164359436
4.11683087736893	5.88417012057865	0.884170120578651	5.40773207663566
1.57552882987259	7.95056826121951	4.57552882987259	5.16049133059375
3.98321214129858	6.4306711182698	5.85768125921472	6.64617715402101
3.80392975127945	6.9143537409731	5.1773881468069	5.227141182004
1.52775287409539	7.63926118931238	2.42083767017887	5.29025356036873
2.49236703874197	7.79875428273355	3.14444373532166	5.00694021157172
3.82338670891689	4.82338670891689	5.14531480380425	6.57827421108036
4.41533421113178	5.91102937375585	6.49939847592025	7.18933053624295
2.70890842401603	6.32629240242956	3.70890842401603	5.43930136425875
5.67206923366443	7.18664240649419	6.18664240649419	7.18664240649419
1.72764543027784	5.41414595746106	1.0495735251652	7.93221657452704

IL20RA-mRNA	SHC2-mRNA	IL5RA-mRNA	TGFB2-mRNA
46.6613636804171	86.6568182636317	36.6625000346134	13.3318181944049
17.3496045823481	59.0624836845893	1.84570261514342	42.4511601482985
2.98044722963945	247.377120060074	23.8435778371156	35.7653667556734
15.814895756651	86.2630677635508	57.9879511077203	100.16100645879
13.9668013602777	120.622375384217	36.1867126152651	37.4564218298358
2.88336379589907	199.364011030736	5.35481847809827	39.1313658014874
5.62700419421764	222.668594542612	8.842435162342	32.1543096812436
14.1564410369496	28.3128820738992	35.391102592374	95.5559769994098
21.3377213042089	60.1723740778691	90.4719383298458	145.950013720789
6.53826759404617	80.2423750178394	13.0765351880923	43.3903213059428
50.9874129446101	145.678322698886	72.8391613494429	145.678322698886
3.31186859066523	42.6403081048148	2.06991786916577	244.250308561561

VEGFC-mRNA	SFRP4-mRNA	CCND2-mRNA	GNG12-mRNA
6.59478263872193	7.43724136173545	4.54415656565196	7.09435364821244
6.5452356003856	6.40773207663566	4.64970486694163	7.60936593780531
5.89745692475995	7.35688854339725	5.96784625265135	8.2048854499522
6.7003692543846	7.90748481513533	6.53873086412725	7.90460230660221
5.86806008869917	9.87197513870255	5.98835432241688	8.0239782321476
6.29782678007353	9.66217919431631	6.13825046692368	8.50175766556244
6.50519107909955	8.2280439369396	4.26997461740552	7.99335114682377
6.3469486649739	6.52382642705798	5.73027730452541	8.24965146361899
6.18086895749475	8.63101280773971	6.32606687303469	8.45447260503874
6.96372232304485	8.07919954046479	7.19784403696341	8.81933241370968
6.86471431160683	7.38827626766384	6.03463931304914	6.77160490721535
6.25902689079415	8.70492535377776	6.34235527439305	8.33127177526403

VEGFC-mRNA	SFRP4-mRNA	CCND2-mRNA	GNG12-mRNA
96.6556819094353	173.313636527263	23.3306818402085	136.65113649265
93.3925523262568	84.9023202965971	25.1015555659504	195.275336682173
59.608944592789	163.92459763017	62.5893918224285	295.064275734306
103.994920581614	240.098871941883	92.9724174784937	239.61963267653
58.4066238702524	937.04540035318	63.4854607285352	260.290388986994
78.6746407166747	810.225226647639	70.4364584426773	362.480020055883
90.8359248495133	299.838937777597	19.2925858087462	254.822904223856
81.3995359624602	92.0168667401724	53.086653888561	304.363482294416
72.5482524343103	396.454861832202	80.2298321038255	350.792138241195
124.821472249972	270.446523208273	146.813826884491	451.734851952281
116.542658159109	167.530071103719	65.5552452144987	109.258742024164
76.5869611591334	417.295442423819	81.1407804712981	322.079220442194

**MAP2K1-mRNA**	**SHC1-mRNA**	**PLCB1-mRNA**	**GRIN2B-mRNA**
5.90672664503667	8.22865473992403	7.38065783336908	1.73680164359436
7.32046524041801	9.18612351570475	5.1769518698065	-0.437757974308711
5.89745692475995	9.28977434753871	8.71508018227138	1.57552882987259
7.4023423952113	10.0368501049007	6.52378052266128	0.523780522661279
6.35572538806541	8.73466708884234	7.29869444302903	-0.655501867357843
6.34244977149416	9.59614730345784	7.95641214393787	-1.27960204796222
6.25486772501531	9.56152906324936	7.44656334912884	0.685012116684361
7.88947589937466	7.88947589937466	6.40834920963804	2.82338670891689
6.67836861696557	8.81860193347108	6.44390336332855	0.356440522078209
6.49740431882232	9.42689634336797	7.57140490026609	-0.750523194621269
6.44967681232799	8.41930316328447	6.77160490721535	3.86471431160683
7.01304764914009	9.60799423843387	6.25120738633485	0.312607930998996

**MAP2K1-mRNA**	**SHC1-mRNA**	**PLCB1-mRNA**	**GRIN2B-mRNA**
59.9931818748219	299.96590937411	166.647727430061	3.33295454860122
159.83784647142	582.503745339262	36.1757712568109	0.738281046057366
59.608944592789	625.893918224285	420.243059379163	2.98044722963945
169.17146066963	1050.49246965391	92.0139389477875	1.43771779605918
81.8962443398104	425.987441488471	157.443942606767	0.634854607285352
81.1460953988739	773.977224642051	248.38119556102	0.411909113699867
76.3664854929536	755.626277509225	174.437130020747	1.60771548406218
237.120387368906	237.120387368906	84.9386462216976	7.0782205184748
102.421062260203	451.506182797061	87.0579029211723	1.28026327825253
90.3469703904561	688.301261264133	190.204148190434	0.594387963095106
87.4069936193316	342.344058342382	109.258742024164	14.5678322698886
129.162875035944	780.359036675496	76.1729775853003	1.24195072149946

FANCA-mRNA	FLNC-mRNA	HIST1H3H-mRNA	SOX9-mRNA
2.73680164359436	5.54415656565196	6.54415656565196	5.54415656565196
3.14720452641244	5.7321670271336	7.38560926573752	5.76191437052765
4.16049133059375	5.89745692475995	7.13011768155023	7.27596854801368
2.52378052266128	4.33113544471888	7.53500777808453	6.35667053682602
3.25138872825068	4.92946063336331	5.68434813552678	6.31028241730424
2.80786079328812	4.94921664253366	6.25177941255409	6.07794995665587
2.00694021157172	5.75110130714213	6.00694021157172	4.7724749579347
3.40834920963804	8.37797556059452	6.68136770404446	8.09017324961179
0.356440522078209	5.37139086354418	6.51967087094651	5.67836861696557
1.83443930609989	7.18021414294162	6.43930136425875	5.87883342545834
2.86471431160683	4.44967681232799	6.44967681232799	4.86471431160683
1.72764543027784	5.25120738633485	6.69918898422861	5.57313548122221

FANCA-mRNA	FLNC-mRNA	HIST1H3H-mRNA	SOX9-mRNA
6.66590909720244	46.6613636804171	93.3227273608341	46.6613636804171
8.85937255268839	53.1562353161304	167.220656931993	54.2636568852164
17.8826833778367	59.608944592789	140.081019793054	154.983255941251
5.75087118423672	20.1280491448285	185.465595691634	81.9499143753733
9.52281910928028	30.4730211496969	51.4232231901135	79.356825910669
7.00245493289774	30.89318352749	76.2031860344754	67.5530946467782
4.01928871015545	53.8584687160831	64.3086193624873	27.3311632290571
10.6173307777122	332.676364368315	102.634197517885	272.51148996128
1.28026327825253	41.3951793301653	91.7522016080983	51.2105311301014
3.56632777857064	145.030662995206	86.7806426118856	58.8444083464155
7.2839161349443	21.8517484048329	87.4069936193316	29.1356645397772
3.31186859066523	38.0864887926502	103.909877032122	47.6081109908127

LIF-mRNA	DTX1-mRNA	NASP-mRNA	FOSL1-mRNA
5.73680164359436	6.19623326223166	6.19623326223166	5.90672664503667
4.86602277386839	4.20609821546601	4.29016248025449	4.1769518698065
3.57552882987259	4.57552882987259	4.57552882987259	4.16049133059375
6.08856514144481	3.93881802194012	4.22422024080237	5.10874302338244
5.59242564608574	3.98835432241688	4.66642622752952	4.80392975127945
5.25955676314581	2.80786079328812	4.6976778755377	4.00580017090003
6.04256412130245	4.00694021157172	4.72940623604281	5.20857407274137
9.38562913313796	5.40834920963804	6.63074163097449	9.91084955016723
4.980931386986	2.57883294341466	4.81587214071551	4.01940553480064
5.51626334607363	3.24947680537873	5.05683172743634	5.22675672887865
4.86471431160683	6.03463931304914	6.56515402974792	5.18664240649419
3.97557294372143	2.53500035233544	4.81510827152818	2.97557294372143

LIF-mRNA	DTX1-mRNA	NASP-mRNA	FOSL1-mRNA
53.3272727776195	73.3250000692268	73.3250000692268	59.9931818748219
29.162101319266	18.4570261514341	19.5644477205202	18.0878856284055
11.9217889185578	23.8435778371156	23.8435778371156	17.8826833778367
68.0519756801345	15.3356564912979	18.6903313487693	34.5052271054203
48.2489501536867	15.8713651821338	25.3941842914141	27.9336027205555
38.3075475740876	7.00245493289774	25.9502741630916	16.0644554342948
65.9163348465495	16.0771548406218	26.527305487026	36.9774561334302
668.891838995869	42.4693231108488	99.0950872586472	962.637990512573
31.5798275302292	5.9745619651785	28.1657921215558	16.2166681911988
45.7678731583232	9.5102074095217	33.2857259333259	37.4464416749917
29.1356645397772	65.5552452144987	94.6909097542759	36.4195806747215
15.7313758056598	5.79577003366415	28.1508830206545	7.86568790282992

ERCC6-mRNA	WNT6-mRNA	GNG7-mRNA	CACNG6-mRNA
5.73680164359436	5.73680164359436	5.98472915703794	5.73680164359436
5.0858039817483	2.26268174383238	4.44488507505313	1.7321670271336
5.7454538313149	3.57552882987259	5.89745692475995	5.16049133059375
5.26074611682749	5.52378052266128	4.14827138756907	6.09868935871851
5.4319609738925	2.34449813264216	5.27523547020504	5.32177805614207
6.12127738831997	2.96832546548137	4.9102225109178	2.42083767017887
5.5676551660462	4.97041433554661	5.00694021157172	2.68501211668436
5.82338670891689	5.73027730452541	6.68136770404446	5.28281832755419
5.88000247813522	2.77147802135705	4.94140302279937	5.96130258023707
5.89333299515346	3.49740431882232	5.13211985474057	4.24947680537873
6.67206923366443	5.67206923366443	6.56515402974792	6.67206923366443
5.08519743489592	1.72764543027784	4.81510827152818	3.58562642540541

ERCC6-mRNA	WNT6-mRNA	GNG7-mRNA	CACNG6-mRNA
53.3272727776195	53.3272727776195	63.3261364234232	53.3272727776195
33.9609281186388	4.79882679937288	21.7792908586923	3.32226470725815
53.6480501335101	11.9217889185578	59.608944592789	35.7653667556734
38.3391412282448	46.0069694738938	17.7318528180632	68.5312149454876
43.1701132954039	5.07883685828282	38.7261310444065	39.9958402589772
69.6126402152776	7.82627316029748	30.0693653000903	5.35481847809827
47.4276067798344	31.3504519392126	32.1543096812436	6.43086193624873
56.6257641477984	53.086653888561	102.634197517885	38.9302128516114
58.8921107996166	6.82807081734685	30.7263186780608	62.30614620829
59.4387963095106	11.293371298807	35.0688898226113	19.0204148190434
101.97482588922	50.9874129446101	94.6909097542759	101.97482588922
33.9466530543186	3.31186859066523	28.1508830206545	12.0055236411615

ID2-mRNA	MAP2K6-mRNA	TP53-mRNA	WNT10B-mRNA
6.43724136173545	6.12911906637312	6.12911906637312	5.73680164359436
8.27304845939064	5.88417012057865	7.71198914519597	4.20609821546601
6.16049133059375	6.52972514025947	6.27596854801368	5.03496044850989
8.19856128563091	5.26074611682749	7.11872711195506	3.46237997799714
6.75388906877986	6.03099865982538	7.0859651190433	3.86806008869917
7.36064288826013	6.28264037625885	6.83933902476129	3.47528545420125
6.46637183020902	5.75110130714213	5.98879286486146	4.20857407274137
9.20709100139094	4.40834920963804	5.82338670891689	6.14531480380425
7.48915444442345	5.81587214071551	6.78270527678031	3.81587214071551
8.10434518863897	5.65886774151643	6.78085826589504	2.70890842401603
7.11264182505042	6.03463931304914	6.95217715285717	6.77160490721535
6.68764736234592	5.75001324330629	7.17472865648749	3.312607930999

ID2-mRNA	MAP2K6-mRNA	TP53-mRNA	WNT10B-mRNA
86.6568182636317	69.9920455206256	69.9920455206256	53.3272727776195
309.339758298036	59.0624836845893	209.671817080292	18.4570261514341
71.5307335113468	92.3938641188229	77.4916279706257	32.784919526034
293.773669661426	38.3391412282448	138.979386952387	11.0225031031204
107.92528323851	65.3900245503913	135.858885959065	14.6016559675631
164.351736366247	77.8508224892749	114.510733608563	11.1215460698964
88.42435162342	53.8584687160831	63.5047616204562	18.4887280667151
591.031413292645	21.2346615554244	56.6257641477984	70.782205184748
179.663613381439	56.3315842431115	110.102641929718	14.0828960607779
275.201626913034	50.522976863084	109.961773172595	6.53826759404617
138.394406563942	65.5552452144987	123.826574294053	109.258742024164
103.081909884455	53.81786459831	144.480267267771	9.93560577199569

CREB3L1-mRNA	PDGFRA-mRNA	AMER1-mRNA	SOS2-mRNA
8.59478263872193	8.27596045470239	5.73680164359436	7.6194446929562
11.4032165955132	9.57626249600622	5.6811830984148	7.78865421848007
8.64161802033036	8.63081126537378	6.66299167112293	7.84231537056749
10.769333228917	9.0460350975315	6.04734247871829	7.62881599335957
8.95920797675736	8.73896082725247	5.49424525214684	7.66191074640703
7.63627733087355	8.70053752967694	5.4346434697039	7.58148485803318
8.51156060397528	9.57527639370547	6.06005154803129	7.66229204018428
7.40834920963804	7.18093871353497	5.40834920963804	8.18093871353497
8.5129450077582	9.04760242663129	5.49939847592025	7.30675339797786
8.03083651890339	9.03247501429914	5.70890842401603	7.63318109785278
7.90910843096528	7.44967681232799	4.44967681232799	7.56515402974792
8.11134972275189	8.92854403531629	5.56053544444258	7.98503327297049

CREB3L1-mRNA	PDGFRA-mRNA	AMER1-mRNA	SOS2-mRNA
386.622727637742	309.964773019913	53.3272727776195	196.644318367472
2708.38401746145	763.382601623316	51.310532700987	221.115173294181
399.379928771686	396.399481542047	101.335205807741	229.494436682238
1745.38940441584	528.600909684425	66.1350186187223	197.925816590814
497.726012111716	427.257150703042	45.07467711726	202.518619724027
198.952101917036	416.028204836866	43.2504569384861	191.537737870438
364.951414882115	762.860997187506	66.7201925885805	202.572150991835
169.877292443395	145.103520628733	42.4693231108488	290.207041257467
365.301788728056	529.175488344381	45.2359691649229	158.32589207723
261.530703761847	523.655795486789	52.3061407523694	198.525579673765
240.369232453162	174.813987238663	21.8517484048329	189.381819508552
276.541027320547	487.258666401622	47.1941274169795	253.35794718589

PTPRR-mRNA	PAX5-mRNA	TNF-mRNA	NGFR-mRNA
5.64369223920288	5.32176414431551	5.32176414431551	7.38065783336908
1.88417012057865	1.14720452641244	1.88417012057865	6.24874255287451
4.38288375193019	3.16049133059375	3.16049133059375	7.06738192620227
3.84570861754864	-1.06118197805988	3.39824964057742	6.97773701123243
1.92946063336331	2.15185305469976	4.55395149827111	7.61596116054653
1.89032295348009	2.6272885476463	3.11271537481654	8.17160906387011
3.00694021157172	2.85493711812667	4.32886830645909	6.68501211668436
6.28281832755419	4.82338670891689	5.40834920963804	6.63074163097449
1.94140302279937	0.771478021357053	3.01940553480064	7.23500239462823
3.05683172743634	1.24947680537873	3.49740431882232	7.68410503301546
5.86471431160683	5.44967681232799	5.44967681232799	5.67206923366443
0.72764543027784	1.312607930999	3.42808514841893	6.99443197097274

PTPRR-mRNA	PAX5-mRNA	TNF-mRNA	NGFR-mRNA
49.9943182290183	39.9954545832146	39.9954545832146	166.647727430061
3.69140523028683	2.2148431381721	3.69140523028683	76.0429477439087
20.8631306074761	8.94134168891835	8.94134168891835	134.120125333775
14.3771779605918	0.47923926535306	10.5432638377673	126.039926787855
3.80912764371211	4.44398225099746	23.489620469558	196.170073651174
3.70718202329881	6.17863670549801	8.65009138769722	288.336379589907
8.03857742031091	7.23471967827982	20.0964435507773	102.89379097998
77.8604257032228	28.3128820738992	42.4693231108488	99.0950872586472
3.8407898347576	1.70701770433671	8.10833409559939	150.644312407715
8.32143148333149	2.37755185238043	11.293371298807	205.658235230907
58.2713290795544	43.7034968096658	43.7034968096658	50.9874129446101
1.65593429533262	2.48390144299892	10.763572919662	127.506940740611

RAD52-mRNA	PIK3R1-mRNA	DLL1-mRNA	TLX1-mRNA
7.09435364821244	7.73680164359436	5.43724136173545	5.64369223920288
6.64970486694163	7.32046524041801	4.36959694774889	1.7321670271336
7.68405328665076	9.76040417278087	6.43350982500016	3.89745692475995
6.68701087152958	8.07580913402035	6.50867363027107	0.523780522661279
7.74537756892434	8.62990035150441	6.00270961539395	3.59242564608574
7.9895246311872	10.0213224430141	5.51481381838789	1.89032295348009
7.5676551660462	10.1718471382474	6.82456346908315	2.00694021157172
6.63074163097449	7.73027730452541	6.86778082827534	5.28281832755419
7.18933053624295	9.86026626007396	6.4509581208625	2.09340611624442
6.73329258264299	9.79637126526637	7.05037670529904	1.83443930609989
6.95217715285717	8.32414593024413	6.44967681232799	5.67206923366443
6.48919666272232	9.9938463427768	5.10268486162476	0.312607930998996

RAD52-mRNA	PIK3R1-mRNA	DLL1-mRNA	TLX1-mRNA
136.65113649265	213.309091110478	43.3284091318159	49.9943182290183
100.406222263802	159.83784647142	20.6718692896062	3.32226470725815
205.650858845122	867.310143825079	86.432969659544	14.9022361481972
103.036442050908	269.811706393773	91.0554604170814	1.43771779605918
214.580857262449	396.14927494606	64.1203153358206	12.0622375384217
254.147923152818	1039.24669386477	45.7219116206853	3.70718202329881
189.710427119337	1153.53585981462	113.343941626384	4.01928871015545
99.0950872586472	212.346615554244	116.790638554834	38.9302128516114
145.950013720789	929.47114001134	87.4846573472566	4.26754426084178
106.395445394024	889.20439279028	132.548515770209	3.56632777857064
123.826574294053	320.492309937549	87.4069936193316	50.9874129446101
89.8344355217944	1019.64154235106	34.3606366281518	1.24195072149946

CSF3-mRNA	GLI1-mRNA	GADD45G-mRNA	THBS4-mRNA
3.73680164359436	6.32176414431551	5.32176414431551	8.291390495272
0.147204526412445	4.44488507505313	4.26268174383238	6.78141054615345
4.38288375193019	6.48241942548111	6.43350982500016	4.16049133059375
1.74617294399773	4.66673847650332	7.26074611682748	10.0381658323433
2.51442313408447	5.45302258942033	5.04493785078325	8.13240069203359
-1.27960204796222	4.14666270673988	6.74830394860767	7.3678563784927
1.26997461740552	5.79353657346253	8.06871640915841	6.95179865737926
5.28281832755419	4.40834920963804	8.95266972586185	7.63074163097449
-1.22852197864295	4.0568802402193	6.29503997741407	5.00029671185293
-0.750523194621269	3.70890842401603	5.67574156008083	4.92190214735023
4.86471431160683	5.67206923366443	7.11264182505042	5.86471431160683
-1.27235456972216	5.0495735251652	5.99443197097274	4.28223428195548

CSF3-mRNA	GLI1-mRNA	GADD45G-mRNA	THBS4-mRNA
13.3318181944049	79.9909091664293	39.9954545832146	313.297727568515
1.10742156908605	21.7792908586923	19.1953071974915	110.003875862548
20.8631306074761	89.4134168891835	86.432969659544	17.8826833778367
3.35467485747142	25.3996810637122	153.356564912979	1051.45094818461
5.71369146556817	43.8049679026893	33.0124395788383	280.605736420126
0.411909113699867	17.7120918890943	107.508278675665	165.175554593647
2.41157322609327	55.4661842001453	268.488485838384	123.794092272788
38.9302128516114	21.2346615554244	495.475436293236	198.190174517294
0.426754426084178	16.643422617283	78.5228143994888	32.0065819563134
0.594387963095106	13.0765351880923	51.1173648261791	30.3137861178504
29.1356645397772	50.9874129446101	138.394406563942	58.2713290795544
0.413983573833154	33.1186859066523	63.7534703703057	19.4572279701582

IFNA17-mRNA	MAPK9-mRNA	DUSP6-mRNA	CACNA2D1-mRNA
7.12911906637312	4.05872973848172	6.73680164359436	7.40922698556585
1.7321670271336	5.76191437052765	6.52802631035338	7.31043487528075
4.38288375193019	4.7454538313149	4.89745692475995	9.64161802033036
1.26074611682749	5.96118583496858	7.24715705207953	7.06810103888509
2.66642622752952	5.227141182004	7.23324138154042	7.37792113417961
2.17982957067508	4.9102225109178	6.45510757226362	7.98014121572856
3.85493711812667	4.72940623604281	6.00694021157172	7.2007119549684
7.65627672308163	4.14531480380425	6.95266972586185	6.52382642705798
1.09340611624442	5.60436803552179	7.18510595038123	7.1766194844934
2.70890842401603	5.57140490026609	7.11366295003301	7.58486716007266
6.86471431160683	5.18664240649419	6.67206923366443	6.95217715285717
1.89757043172015	5.21949852660751	6.70492535377776	6.67015993561708

IFNA17-mRNA	MAPK9-mRNA	DUSP6-mRNA	CACNA2D1-mRNA
139.984091041251	16.6647727430061	106.654545555239	169.980681978662
3.32226470725815	54.2636568852164	92.2851307571708	158.730424902334
20.8631306074761	26.824025066755	29.8044722963945	798.759857543372
2.3961963267653	62.3011044958978	151.91884711692	134.186994298857
6.34854607285352	37.4564218298358	150.460541926628	166.331907108762
4.53100025069854	30.0693653000903	87.7366412180717	252.500286698019
14.4694393565596	26.527305487026	64.3086193624873	147.10596679169
201.729284776532	17.695551296187	123.868859073309	92.0168667401724
2.13377213042089	48.6500045735963	145.523259294705	144.669750442536
6.53826759404617	47.5510370476085	138.49239540116	191.987312079719
116.542658159109	36.4195806747215	101.97482588922	123.826574294053
3.72585216449838	37.2585216449838	104.323860605955	101.839959162956

PPP3R1-mRNA	TNFSF10-mRNA	ITGB7-mRNA	ITGA6-mRNA
7.75916945662281	6.73680164359436	4.05872973848172	6.43724136173545
8.7582293237198	4.79106071618717	3.14720452641244	6.40144581378823
8.13011768155023	5.38288375193019	5.66299167112293	5.82345634331618
8.69870620516196	7.82756127083838	3.33113544471888	6.99410045744131
7.83233816646521	7.62062253791639	4.34449813264216	7.47378114958712
8.04907287936573	7.36425414181251	4.24395990809479	7.53738157529316
8.11546466834989	6.47942798303447	3.68501211668436	7.63337934826904
8.23277764505459	6.63074163097449	4.14531480380425	6.40834920963804
8.16164697755724	8.82404607216121	4.47191773949814	7.61382836477086
8.49265078885168	8.16834004265333	2.70890842401603	8.25790542744931
6.44967681232799	7.03463931304914	3.86471431160683	7.7226953067344
8.76519238424002	6.45556588484104	3.63453602588636	7.56999577369165

PPP3R1-mRNA	TNFSF10-mRNA	ITGB7-mRNA	ITGA6-mRNA
216.642045659079	106.654545555239	16.6647727430061	86.6568182636317
433.001833512645	27.6855392271512	8.85937255268839	84.5331797735684
280.162039586108	41.7262612149523	50.6676029038706	56.6284973631495
415.500443061103	227.15941177735	10.0640245724143	127.477644583914
227.912804015441	196.804928258459	20.3153474331313	177.759290039899
264.857560109015	164.763645479947	18.9478192301939	185.77101027864
277.330921000727	89.2282093654511	12.8617238724975	198.552862281679
300.824372035179	99.0950872586472	17.695551296187	84.9386462216976
286.352219902484	453.213200501397	22.1912301563773	195.880281572638
360.199105635635	287.683774138032	6.53826759404617	306.10980099398
87.4069936193316	131.110490428997	14.5678322698886	211.233567913384
435.096736098644	87.7645176526286	12.4195072149946	190.018460389417

FOS-mRNA	IL12B-mRNA	WNT3-mRNA	DKK1-mRNA
11.3770465798167	3.73680164359436	4.73680164359436	6.05872973848172
8.98640831450939	-0.437757974308711	4.20609821546601	5.59566502722874
9.95056826121952	3.57552882987259	5.03496044850989	4.7454538313149
11.2786680248247	0.523780522661279	3.10874302338244	7.93881802194012
11.1344392610331	0.929460633363313	4.29869444302903	5.07241858720536
8.73859813085101	0.305360452758937	4.14666270673988	5.54057691445297
11.2347967846322	2.26997461740552	4.00694021157172	5.68501211668436
11.9253623798661	5.28281832755419	5.52382642705798	6.21570413169565
11.2645829138962	1.35644052207821	3.01940553480064	7.07525876953416
12.0453135463124	0.834439306099887	3.41940180682104	6.03083651890339
10.0545388704868	2.86471431160683	5.44967681232799	6.44967681232799
8.80312457976586	-0.27235456972216	4.5090051438025	7.33497574402745

FOS-mRNA	IL12B-mRNA	WNT3-mRNA	DKK1-mRNA
2659.69772978377	13.3318181944049	26.6636363888098	66.6590909720244
507.19907864141	0.738281046057366	18.4570261514341	48.3574085167575
989.508480240298	11.9217889185578	32.784919526034	26.824025066755
2484.37635159026	1.43771779605918	8.62630677635508	245.370503860767
2248.02016439743	1.90456382185606	19.6804928258459	33.6472941861237
427.149750906762	1.2357273410996	17.7120918890943	46.545729848085
2409.96551060921	4.82314645218654	16.0771548406218	51.4468954899898
3889.4821749019	38.9302128516114	46.0084333700862	74.3213154439854
2460.23926637529	2.56052655650507	8.10833409559939	134.8543986426
4226.6928055693	1.78316388928532	10.6989833357119	65.3826759404617
1063.45175570187	7.2839161349443	43.7034968096658	87.4069936193316
446.688276165973	0.827967147666308	22.7690965608235	161.45359379493

GSK3B-mRNA	DUSP10-mRNA	HDAC11-mRNA	FGF1-mRNA
6.43724136173545	4.73680164359436	5.64369223920288	4.90672664503667
6.87512498097564	3.42022302081886	5.00518552154002	5.62833121614906
6.57552882987259	5.48241942548111	6.0990907859296	4.7454538313149
7.04734247871829	4.46237997799714	5.74617294399773	4.4306711182698
6.82023156360855	4.80392975127945	4.89908698431979	5.34449813264216
6.96832546548137	4.78648714249555	5.56588800298216	6.18800350212078
6.74029455218555	5.04256412130245	5.91383080718024	5.239600968362
6.21570413169565	7.46724289869161	5.73027730452541	5.91084955016723
6.95138711137199	4.62945901648462	6.04760242663129	5.57883294341466
6.95683593745961	4.29387092473718	5.93597733256195	5.03083651890339
6.44967681232799	5.44967681232799	6.18664240649419	4.86471431160683
6.71063900497215	3.25120738633485	5.96605016960292	6.79910779283446

GSK3B-mRNA	DUSP10-mRNA	HDAC11-mRNA	FGF1-mRNA
86.6568182636317	26.6636363888098	49.9943182290183	29.996590937411
117.386686323121	10.7050751678318	32.1152255034954	49.4648300858435
95.3743113484624	44.7067084445918	68.5502862817074	26.824025066755
132.270037237445	22.0450062062408	53.6747977195427	21.5657669408877
113.004120096793	27.9336027205555	29.8381665424116	40.6306948662625
125.22037056476	27.5979106178911	47.3695480754847	72.9079131248765
106.913079690135	32.9581674232747	60.2893306523318	37.7813138754613
74.3213154439854	176.95551296187	53.086653888561	60.1648744070358
123.758783564412	24.7517567128823	66.1469360430476	47.796495721428
124.227084286877	19.6148027821385	61.221960198796	32.6913379702309
87.4069936193316	43.7034968096658	72.8391613494429	29.1356645397772
104.737844179788	9.52162219816254	62.5115196488062	111.361581361118

**CHEK1-mRNA**	**RAF1-mRNA**	**CACNB3-mRNA**	**CDC6-mRNA**
6.12911906637312	6.64369223920288	5.32176414431551	5.90672664503667
4.23466736766278	7.71958896105413	5.57346928111454	3.7321670271336
4.7454538313149	7.50626616743548	5.7454538313149	5.16049133059375
4.63925774008121	7.5608698413965	4.86955535950301	5.12864258082014
3.34449813264216	7.83233816646521	6.1773881468069	3.66642622752952
3.76479207139623	7.9102225109178	5.47528545420125	4.55328796620252
4.20857407274137	7.65079640134645	5.91383080718024	4.54299311181193
5.91084955016723	7.97313382842157	5.40834920963804	6.3469486649739
2.47191773949815	7.60120075644311	5.55283773488171	2.77147802135705
3.05683172743634	7.54409755427036	6.06965576779392	3.89333299515346
6.03463931304914	7.56515402974792	6.32414593024413	6.56515402974792
2.18707704891514	7.46911241667899	4.75001324330629	2.53500035233544

**CHEK1-mRNA**	**RAF1-mRNA**	**CACNB3-mRNA**	**CDC6-mRNA**
69.9920455206256	99.9886364580366	39.9954545832146	59.9931818748219
18.8261666744628	210.779238649378	47.6191274707001	13.2890588290326
26.824025066755	181.807281008006	53.6480501335101	35.7653667556734
24.9204417983591	188.820270549106	29.2335951865367	34.9844663707734
10.1576737165656	227.912804015441	72.3734252305301	12.697092145707
13.5930007520956	240.554922400723	44.4861842795857	23.4788194808924
18.4887280667151	200.964435507773	60.2893306523318	23.3118745189016
60.1648744070358	251.276828405855	42.4693231108488	81.3995359624602
5.54780753909432	194.173263868301	46.9429868692596	6.82807081734685
8.32143148333149	186.637820411863	67.165839829747	14.8596990773777
65.5552452144987	189.381819508552	80.1230774843872	94.6909097542759
4.55381931216469	177.18496960059	26.908932299155	5.79577003366415

CDKN2D-mRNA	IL1B-mRNA	IGF1R-mRNA	H2AFX-mRNA
6.05872973848172	4.54415656565196	8.84532610037253	5.43724136173545
5.0858039817483	-1.43775797430871	8.49887996469386	5.53952194919121
5.03496044850989	2.57552882987259	9.05126226083899	4.7454538313149
5.20560456263502	2.74617294399773	9.44562145973577	5.41455145290652
4.80392975127945	4.20247912776973	9.40789321393067	4.59242564608574
4.24395990809479	2.04232604692514	9.83414011808697	4.44831840660098
4.43989961884783	3.14444373532166	9.95764190166073	4.49236703874196
6.68136770404446	7.57827421108036	8.55130716348009	7.57827421108036
4.29503997741407	1.77147802135705	9.88652167151876	4.77147802135705
3.77303876143574	1.83443930609989	9.44423365980098	4.97739725994193
5.67206923366443	5.18664240649419	9.44967681232799	5.86471431160683
4.63453602588636	1.312607930999	8.50736478542125	4.83616988705601

CDKN2D-mRNA	IL1B-mRNA	IGF1R-mRNA	H2AFX-mRNA
66.6590909720244	23.3306818402085	459.947727706969	43.3284091318159
33.9609281186388	0.369140523028683	361.75771256811	46.5117059016141
32.784919526034	5.9608944592789	530.519606875822	26.824025066755
36.9014234321856	6.70934971494284	697.293131088702	42.6522946164223
27.9336027205555	18.4107836112752	679.294429795327	24.1244750768434
18.9478192301939	4.11909113699867	912.790595958907	21.831183026093
21.7041590348394	8.842435162342	994.37202689246	22.5080167768705
102.634197517885	191.11195399882	375.145687479164	191.11195399882
19.6307035998722	3.41403540867343	946.541317054707	27.3122832693874
13.6709231511874	3.56632777857064	696.622692747464	31.5025620440406
50.9874129446101	36.4195806747215	699.255948954653	58.2713290795544
24.8390144299892	2.48390144299892	363.891561399342	28.5648665944876

IDH1-mRNA	PIK3CD-mRNA	ENDOG-mRNA	IBSP-mRNA
4.54415656565196	6.86608466053932	5.73680164359436	8.53121750994447
6.28334121439847	5.63905762274212	5.78141054615345	11.4549745583031
4.7454538313149	7.1902386739878	6.38288375193019	7.66299167112293
5.85768125921472	5.80918274152353	6.1772227612652	9.44363700957296
5.15185305469976	6.98835432241688	5.86806008869917	9.12749634156257
5.29025356036873	7.33878345429639	5.17982957067508	8.29025356036873
5.00694021157172	7.18485800376757	4.85493711812667	7.29972196079957
2.82338670891689	6.68136770404446	5.40834920963804	9.19842614026381
5.74875794485697	6.47883715343794	5.29503997741407	7.76016270812922
5.87883342545834	6.26070406080199	5.67574156008083	7.13211985474057
5.18664240649419	6.86471431160683	5.44967681232799	5.18664240649419
5.59801014986124	5.86719678267663	5.48253293244131	9.06972009827698

IDH1-mRNA	PIK3CD-mRNA	ENDOG-mRNA	IBSP-mRNA
23.3306818402085	116.653409201043	53.3272727776195	369.957954894736
77.8886503590521	49.8339706088722	55.0019379312738	2807.31367763313
26.824025066755	146.041914252333	83.4525224299046	202.670411615483
57.9879511077203	56.070994046308	72.365129068312	696.334652557996
35.5518580079797	126.97092145707	58.4066238702524	559.306909018395
39.1313658014874	161.880281684048	36.2480020055883	313.050926411899
32.1543096812436	145.498251307627	28.9388787131193	157.556117438094
7.0782205184748	102.634197517885	42.4693231108488	587.492303033408
53.7710576866065	89.1916750515932	39.2614071997444	216.791248450762
58.8444083464155	76.6760472392687	51.1173648261791	140.275559290445
36.4195806747215	116.542658159109	43.7034968096658	36.4195806747215
48.436078138479	58.3716839104747	44.7102259739806	537.350678835433

THEM4-mRNA	LAMB4-mRNA	TBL1XR1-mRNA	ATM-mRNA
6.78119576295281	1.73680164359436	8.05872973848172	5.90672664503667
5.57346928111454	1.88417012057865	8.51643833607816	6.83836643096553
6.27596854801368	2.57552882987259	7.98491976601029	6.43350982500016
6.26074611682749	1.52378052266128	8.27420837663405	6.03685010490065
7.1837019207391	1.34449813264216	8.68434813552678	5.61128467333706
7.30536045275894	1.04232604692514	8.87394998374589	5.46186493843893
6.47942798303447	-0.314987883315639	8.17285215050741	6.04256412130245
5.73027730452541	3.40834920963804	7.88947589937466	6.07131422236047
5.980931386986	0.356440522078209	8.84829361840788	5.8049010228945
5.97739725994193	-0.750523194621269	8.802145902893	6.28289980691618
7.32414593024413	2.86471431160683	7.6196018137703	6.03463931304914
5.70492535377776	-0.27235456972216	8.85305890076116	7.23544007047654

THEM4-mRNA	LAMB4-mRNA	TBL1XR1-mRNA	ATM-mRNA
109.98750010384	3.33295454860122	266.636363888097	59.9931818748219
47.6191274707001	3.69140523028683	366.187398844454	114.433562138892
77.4916279706257	5.9608944592789	253.338014519353	86.432969659544
76.6782824564896	2.87543559211836	309.588565418076	65.6557793533692
145.381705068346	2.53941842914141	411.385785520908	48.8838047609721
158.173099660749	2.05954556849934	469.164480504148	44.0742751658858
89.2282093654511	0.803857742031091	288.584929389162	65.9163348465495
53.086653888561	10.6173307777122	237.120387368906	67.2430949255106
63.1596550604584	1.28026327825253	460.894780170913	55.9048298170273
63.0051240880813	0.594387963095106	446.385360284425	77.8648231654589
160.246154968774	7.2839161349443	196.665735643496	65.5552452144987
52.1619303029774	0.827967147666308	462.419651971633	150.690020875268

MLH1-mRNA	RXRG-mRNA	FEN1-mRNA	MYB-mRNA
7.59478263872193	5.05872973848172	4.32176414431551	4.32176414431551
6.89763238038521	1.7321670271336	3.60663614504974	0.562242025691289
7.93308083449067	4.7454538313149	2.57552882987259	3.16049133059375
7.32252231441417	1.74617294399773	3.74617294399773	5.68028500834127
7.03099865982538	3.80392975127945	2.51442313408447	5.01692347461365
7.78379303332629	2.42083767017887	3.30536045275894	2.72039795203778
7.81429513362933	2.49236703874197	3.14444373532166	4.63920842707124
6.73027730452541	6.07131422236047	5.9933117103592	5.28281832755419
6.97115036619342	2.35644052207821	2.94140302279937	6.56589388770716
6.97739725994193	3.94991652351982	2.83443930609989	4.64179422815749
7.32414593024413	4.86471431160683	5.44967681232799	4.86471431160683
6.88246353932994	3.1199628530566	2.63453602588636	4.08519743489592

MLH1-mRNA	RXRG-mRNA	FEN1-mRNA	MYB-mRNA
193.311363818871	33.3295454860122	19.9977272916073	19.9977272916073
119.232388938265	3.32226470725815	12.1816372599465	1.47656209211473
244.396672830435	26.824025066755	5.9608944592789	8.94134168891835
160.065914627922	3.35467485747142	13.4186994298857	51.2786013927774
130.780049100783	13.9668013602777	5.71369146556817	32.3775849715529
220.371375829429	5.35481847809827	9.88581872879682	6.59054581919788
225.080167768706	5.62700419421764	8.842435162342	24.9195900029638
106.173307777122	67.2430949255106	63.7039846662732	38.9302128516114
125.465801268748	5.12105311301014	7.68157966951521	94.7394825906876
126.010248176163	15.4540870404728	7.13265555714128	24.9642944499945
160.246154968774	29.1356645397772	43.7034968096658	29.1356645397772
117.985318542449	8.69365505049623	6.20975360749731	16.9733265271593

**MAPK10-mRNA**	**MAP3K12-mRNA**	**DDIT4-mRNA**	**CHUK-mRNA**
5.32176414431551	6.05872973848172	4.90672664503667	7.12911906637312
4.71198914519597	5.57346928111454	9.00518552154002	6.62293795737884
4.7454538313149	7.00179358457469	6.78498219550154	6.78498219550154
5.80918274152353	6.56817464201973	7.68365185943967	7.18674553538371
4.80392975127945	7.27523547020504	6.96654995209853	7.37240412921204
3.6272885476463	7.16334144788651	9.99419355125205	7.23609779032182
4.38545183482545	7.29972196079957	9.21053292577943	7.38545183482545
5.82338670891689	4.63074163097449	9.38562913313796	7.24965146361899
5.71399252669629	6.41533421113178	9.76229110051066	6.666295784665
4.41940180682104	6.48788154470381	9.58932680826335	6.90052849655766
6.03463931304914	5.86471431160683	8.64607402513149	6.03463931304914
4.18707704891514	5.96605016960292	10.6182897138969	6.37150162005256

**MAPK10-mRNA**	**MAP3K12-mRNA**	**DDIT4-mRNA**	**CHUK-mRNA**
39.9954545832146	66.6590909720244	29.996590937411	139.984091041251
26.2089771350365	47.6191274707001	513.843608055927	98.5605196486584
26.824025066755	128.159230874496	110.27654749666	110.27654749666
56.070994046308	94.8893745399059	205.593644836463	145.68873666733
27.9336027205555	154.904524177626	125.066357635214	165.697052501477
12.357273410996	143.344371567554	1019.88696552087	150.758735614151
20.9003012928084	157.556117438094	592.443155876915	167.202410342467
56.6257641477984	24.7737718146618	668.891838995869	152.181741147208
52.4907944083539	85.3508852168356	868.445257081303	101.567553408034
21.3979666714238	89.752582427361	770.326800171257	119.471980582116
65.5552452144987	58.2713290795544	400.615387421936	65.5552452144987
18.2152772486588	62.5115196488062	1571.89562984448	82.7967147666307

IL8-mRNA	MGMT-mRNA	MAPK12-mRNA	SOX17-mRNA
5.73680164359436	7.19623326223166	5.32176414431551	4.73680164359436
1.88417012057865	7.40773207663566	3.91979403030937	3.69152504263625
5.03496044850989	7.45817187923443	5.16049133059375	4.89745692475995
7.50106044616119	7.26973490005474	4.86955535950301	3.98321214129858
6.01692347461365	8.0239782321476	4.98835432241688	3.73681555542092
6.04232604692514	8.60914120093604	4.98718449273268	4.39282329400928
6.09440305282206	7.66229204018428	5.38545183482545	3.49236703874197
15.8134906727744	6.63074163097449	5.28281832755419	5.91084955016723
4.35644052207821	7.85362706271092	4.0568802402193	3.81587214071551
5.92190214735023	7.60702880999682	4.41940180682104	5.51626334607363
6.18664240649419	7.77160490721535	3.86471431160683	5.18664240649419
3.48253293244131	7.66428336928041	4.0495735251652	4.68184174066471

IL8-mRNA	MGMT-mRNA	MAPK12-mRNA	SOX17-mRNA
53.3272727776195	146.650000138454	39.9954545832146	26.6636363888098
3.69140523028683	169.804640593194	15.134761444176	12.9199183060039
32.784919526034	175.846386548728	35.7653667556734	29.8044722963945
181.152442303457	154.315043443685	29.2335951865367	15.814895756651
64.7551699431059	260.290388986994	31.7427303642676	13.3319467529924
65.9054581919788	390.489839787474	31.7170017548898	21.0073647986932
68.3279080726427	202.572150991835	41.8006025856167	11.2540083884353
57588.402138311	99.0950872586472	38.9302128516114	60.1648744070358
20.4842124520406	231.300898937624	16.643422617283	14.0828960607779
60.6275722357008	194.959251895195	21.3979666714238	45.7678731583232
72.8391613494429	218.517484048329	14.5678322698886	36.4195806747215
11.1775564934951	202.851951178245	16.5593429533262	25.6669815776555

MLF1-mRNA	FGF8-mRNA	ALK-mRNA	ARNT2-mRNA
5.05872973848172	4.05872973848172	4.54415656565196	5.43724136173545
5.52802631035338	1.14720452641244	1.14720452641244	2.95455944847005
4.16049133059375	2.57552882987259	4.89745692475995	4.57552882987259
6.61829812144557	0.523780522661279	1.26074611682749	3.79679901706769
5.29869444302903	0.929460633363313	2.34449813264216	3.66642622752952
4.57837894716535	-1.27960204796222	2.42083767017887	3.57837894716535
4.14444373532166	2.00694021157172	2.26997461740552	4.59190271229288
6.3469486649739	3.40834920963804	1.82338670891689	5.52382642705798
7.15949530670219	-0.228521978642947	2.67836861696557	3.90076103830202
5.77303876143574	1.24947680537873	2.41940180682104	4.57140490026609
6.56515402974792	2.86471431160683	4.86471431160683	6.18664240649419
5.28223428195548	-1.27235456972216	1.72764543027784	3.72764543027784

MLF1-mRNA	FGF8-mRNA	ALK-mRNA	ARNT2-mRNA
33.3295454860122	16.6647727430061	23.3306818402085	43.3284091318159
46.1425653785854	2.2148431381721	2.2148431381721	7.75195098360235
17.8826833778367	5.9608944592789	29.8044722963945	23.8435778371156
98.2440493973773	1.43771779605918	2.3961963267653	13.8979386952387
39.3609856516918	1.90456382185606	5.07883685828282	12.697092145707
23.8907285945923	0.411909113699867	5.35481847809827	11.9453642972962
17.684870324684	4.01928871015545	4.82314645218654	24.1157322609327
81.3995359624602	10.6173307777122	3.5391102592374	46.0084333700862
142.9627327382	0.853508852168356	6.40131639126267	14.9364049129462
54.6836926047498	2.37755185238043	5.34949166785596	23.7755185238043
94.6909097542759	7.2839161349443	29.1356645397772	72.8391613494429
38.9144559403165	0.413983573833154	3.31186859066523	13.2474743626609

MET-mRNA	HOXA10-mRNA	SIN3A-mRNA	CTNNB1-mRNA
6.59478263872193	5.90672664503667	5.90672664503667	9.06771852170898
4.39513203985603	2.26268174383238	6.13209763402224	11.1185085692743
5.82345634331618	5.38288375193019	6.21938501964731	9.59233711755915
7.06293933376931	2.84570861754864	6.26973490005474	10.8438288843304
6.41058732309993	3.51442313408447	6.11268245741908	10.5037387830538
5.29025356036873	3.42083767017887	6.25955676314581	11.6123714710633
5.54299311181193	4.07732953946312	5.83475923618904	10.3015609604634
6.77758301930376	5.73027730452541	6.82338670891689	10.3508637149773
6.84829361840788	2.47191773949815	6.4081026419007	10.7448166421574
7.17428930898451	2.41940180682104	6.73329258264299	10.7053177145051
5.18664240649419	7.03463931304914	7.32414593024413	9.89813731314428
5.84658650300135	2.18707704891514	6.48919666272232	10.5656674885824

MET-mRNA	HOXA10-mRNA	SIN3A-mRNA	CTNNB1-mRNA
96.6556819094353	59.9931818748219	59.9931818748219	536.605682324797
21.0410098126349	4.79882679937288	70.1366993754498	2223.33337020176
56.6284973631495	41.7262612149523	74.5111807409862	771.935832476618
133.707755033504	7.1885889802959	77.1575217218427	1837.88258262898
85.0705173762372	11.4273829311363	69.1991521941034	1451.9124868616
39.1313658014874	10.7096369561965	76.6150951481753	3130.92117323269
46.6237490378033	16.8810125826529	57.0738996842075	1262.05665498881
109.712418036359	53.086653888561	113.251528295597	1305.9316856586
115.223695042728	5.54780753909432	84.9241307907515	1715.97954728448
144.436275032111	5.34949166785596	106.395445394024	1669.63578833415
36.4195806747215	131.110490428997	160.246154968774	954.193013677703
57.5437167628084	4.55381931216469	89.8344355217944	1515.59386380318

**WNT7A-mRNA**	**CDKN2A-mRNA**	**ARID1A-mRNA**	**FGF2-mRNA**
4.54415656565196	5.05872973848172	5.8242644848447	4.54415656565196
2.02167364432859	3.26268174383238	6.61752446119248	4.34360173921595
5.03496044850989	4.89745692475995	5.96784625265135	7.30344928443579
1.93881802194012	3.18674553538371	6.47797683304815	4.893014332327
3.34449813264216	2.80392975127945	6.0859651190433	5.29869444302903
2.96832546548137	3.67459426242466	5.85994930443657	5.99652235731202
2.85493711812667	3.38545183482545	6.37151264386758	5.63920842707124
4.82338670891689	4.14531480380425	6.46724289869161	4.9933117103592
0.771478021357053	2.35644052207821	6.56589388770716	5.666295784665
2.41940180682104	2.57140490026609	6.60702880999682	6.82690563341448
5.44967681232799	5.44967681232799	5.67206923366443	5.44967681232799
2.0495735251652	3.0495735251652	6.44874461898502	5.44189094794396

**WNT7A-mRNA**	**CDKN2A-mRNA**	**ARID1A-mRNA**	**FGF2-mRNA**
23.3306818402085	33.3295454860122	56.6602273262207	23.3306818402085
4.06054575331551	9.59765359874576	98.1913791256297	20.3027287665776
32.784919526034	29.8044722963945	62.5893918224285	157.963703170891
3.83391412282448	9.10554604170814	89.1385033556691	29.7128344518897
10.1576737165656	6.98340068013887	67.9294429795327	39.3609856516918
7.82627316029748	12.7691825246959	58.0791850316813	63.8459126234794
7.23471967827982	10.4501506464042	82.7973474292024	49.8391800059276
28.3128820738992	17.695551296187	88.477756480935	31.8519923331366
1.70701770433671	5.12105311301014	94.7394825906876	50.7837767040172
5.34949166785596	5.94387963095106	97.4796259475975	113.528100951165
43.7034968096658	43.7034968096658	50.9874129446101	43.7034968096658
4.13983573833154	8.27967147666308	87.3505340787954	43.4682752524811

PLA2G2A-mRNA	MLLT3-mRNA	PLA2G3-mRNA	HIST1H3B-mRNA
5.8242644848447	3.32176414431551	4.90672664503667	6.78119576295281
3.0858039817483	5.67076648246946	0.562242025691289	7.34360173921595
6.33041633203606	5.57552882987259	3.16049133059375	5.16049133059375
2.63925774008122	4.96118583496858	0.523780522661279	7.25623063570499
5.80392975127945	5.34449813264216	2.80392975127945	5.51442313408447
4.67459426242466	4.76479207139623	2.04232604692514	5.52775287409538
3.14444373532166	5.11127687138646	0.685012116684361	4.68501211668436
6.46724289869161	4.9933117103592	4.14531480380425	6.63074163097449
3.47191773949814	5.18086895749475	2.23090963999435	4.32606687303469
4.33693964662907	5.24947680537873	1.83443930609989	4.64179422815749
5.67206923366443	5.86471431160683	6.18664240649419	5.44967681232799
1.53500035233544	3.58562642540541	0.72764543027784	5.57313548122221

PLA2G2A-mRNA	MLLT3-mRNA	PLA2G3-mRNA	HIST1H3B-mRNA
56.6602273262207	9.99886364580366	29.996590937411	109.98750010384
8.49023202965971	50.9413921779583	1.47656209211473	162.421830132621
80.4720752002651	47.6871556742312	8.94134168891835	35.7653667556734
6.23011044958978	31.1505522479489	1.43771779605918	152.877325647626
55.867205441111	40.6306948662625	6.98340068013887	45.7095317245453
25.5383650493918	27.1860015041912	4.11909113699867	46.1338207343851
8.842435162342	34.5658829073369	1.60771548406218	25.7234477449949
88.477756480935	31.8519923331366	17.695551296187	99.0950872586472
11.0956150781886	36.2741262171551	4.69429868692596	20.0574580259564
20.2091907452336	38.0408296380868	3.56632777857064	24.9642944499945
50.9874129446101	58.2713290795544	72.8391613494429	43.7034968096658
2.89788501683208	12.0055236411615	1.65593429533262	47.6081109908127

CHAD-mRNA	PDGFA-mRNA	NOTCH2-mRNA	PPP2CB-mRNA
10.1755934961726	6.26036359965137	8.07665164647898	6.43724136173545
13.2594268089158	6.91097017992237	9.47961410516813	8.25049233482447
8.30344928443579	5.82345634331618	9.0990907859296	7.16049133059375
8.94022621633293	6.50867363027107	8.66333187506007	8.02893044160441
10.3081167500861	5.25138872825068	8.21486285222556	7.44253021560268
10.4251661914004	5.09543738338471	9.19308879128056	7.92252177586824
8.77776925760421	5.79353657346253	8.81943843690529	7.65079640134645
10.0328400745458	6.40834920963804	7.77758301930376	8.12716745709399
10.2984662267891	5.666295784665	9.10239489947167	8.15734042199851
8.6609877933908	5.29387092473718	8.8117192295998	8.38902815777752
8.38827626766384	6.03463931304914	8.03463931304914	7.81891062199371
11.5036671455307	5.88751676705623	9.81444511590129	8.76793515130356

CHAD-mRNA	PDGFA-mRNA	NOTCH2-mRNA	PPP2CB-mRNA
1156.53522836462	76.6579546178281	269.969318436699	86.6568182636317
9805.84885373394	120.339810507351	713.917771537473	304.540931498663
315.927406341782	56.6284973631495	548.402290253659	143.061467022694
491.220246986886	91.0554604170814	405.436418488689	261.185399617418
1267.80465074885	38.0912764371211	297.111956209545	173.950162396187
1374.95262153016	34.188456437089	585.322850567512	242.614467969222
438.906327148975	55.4661842001453	451.768051021473	200.964435507773
1047.57663673427	84.9386462216976	219.424836072719	279.589710479755
1259.35231137441	50.7837767040172	549.659700796422	285.498711050315
404.778202867767	39.229605564277	449.3573000999	335.23481118564
335.060142207438	65.5552452144987	262.220980857995	225.801400183273
2903.68078686574	59.199651058141	900.41427308711	435.924703246311

SFRP2-mRNA	IKBKB-mRNA	FGF12-mRNA	RIN1-mRNA
6.38065783336908	7.40922698556585	5.05872973848172	5.8242644848447
5.22045350844308	6.63370438824791	3.0858039817483	5.55092671246345
8.97640826615477	7.59789664290104	4.38288375193019	6.70481184681756
8.36298431075822	7.20560456263502	4.10874302338244	6.27866802482475
8.81006453745156	7.70205013726024	4.95920797675736	5.88365694375019
10.2272013898334	7.78379303332629	3.80786079328812	5.77568038753897
9.31982316685608	7.81429513362933	2.49236703874197	6.34322359943616
5.14531480380425	5.63074163097449	5.52382642705798	5.14531480380425
9.6866104703077	7.36766777750146	3.29503997741407	5.86951010431758
10.8045451967121	7.2381614921509	3.77303876143574	5.57140490026609
6.44967681232799	3.86471431160683	5.18664240649419	5.67206923366443
9.94802375797307	6.48919666272232	2.97557294372143	5.06749543316246

SFRP2-mRNA	IKBKB-mRNA	FGF12-mRNA	RIN1-mRNA
83.3238637150305	169.980681978662	33.3295454860122	56.6602273262207
37.283192825897	99.2988006947157	8.49023202965971	46.8808464246427
503.695581809067	193.729069926564	20.8631306074761	104.315653037381
329.237375297552	147.605693728743	17.2526135527102	77.6367609871957
448.842207350744	208.232311189595	31.1078757569823	59.0414784775377
1198.65552086661	220.371375829429	14.0049098657955	54.7839121220824
639.066904914717	225.080167768706	5.62700419421764	81.1896319451402
35.391102592374	49.5475436293236	46.0084333700862	35.391102592374
824.062796768548	165.153962894577	9.8153517999361	58.4653563735324
1788.51338095318	150.974542626157	13.6709231511874	47.5510370476085
87.4069936193316	14.5678322698886	36.4195806747215	50.9874129446101
987.764807165905	89.8344355217944	7.86568790282992	33.5326694804854

WNT5A-mRNA	MAP2K4-mRNA	GNGT1-mRNA	FGF6-mRNA
7.12911906637312	6.38065783336908	4.05872973848172	2.73680164359436
7.70179337809008	6.69152504263626	0.884170120578651	2.14720452641244
4.38288375193019	6.52972514025947	3.57552882987259	4.38288375193019
7.85768125921472	6.46237997799714	0.938818021940123	1.74617294399773
7.5392549870644	5.92946063336331	1.92946063336331	0.929460633363313
7.86250500934033	5.77568038753897	1.30536045275894	2.04232604692514
8.93293963012795	6.46637183020902	1.26997461740552	1.26997461740552
6.68136770404446	6.28281832755419	4.9933117103592	3.40834920963804
7.47538159480172	6.610681809454	2.23090963999435	1.09340611624442
7.67574156008083	6.68410503301546	-0.750523194621269	-0.750523194621269
6.18664240649419	6.03463931304914	4.86471431160683	5.18664240649419
6.37150162005256	6.58562642540541	0.72764543027784	0.312607930998996

WNT5A-mRNA	MAP2K4-mRNA	GNGT1-mRNA	FGF6-mRNA
139.984091041251	83.3238637150305	16.6647727430061	6.66590909720244
208.195254988177	103.359346448031	1.84570261514342	4.4296862763442
20.8631306074761	92.3938641188229	11.9217889185578	20.8631306074761
231.951804430881	88.1800248249631	1.91695706141224	3.35467485747142
186.012399934608	60.9460422993938	3.80912764371211	1.90456382185606
232.728649240425	54.7839121220824	2.4714546821992	4.11909113699867
488.745507154903	88.42435162342	2.41157322609327	2.41157322609327
102.634197517885	77.8604257032228	31.8519923331366	10.6173307777122
177.956595677102	97.7267635732768	4.69429868692596	2.13377213042089
204.469459304717	102.829117615453	0.594387963095106	0.594387963095106
72.8391613494429	65.5552452144987	29.1356645397772	36.4195806747215
82.7967147666307	96.0441891292917	1.65593429533262	1.24195072149946

CCND3-mRNA	FST-mRNA	UTY-mRNA	NR4A1-mRNA
6.38065783336908	626.036.359.965.137	373.680.164.359.436	935.151.148.770.957
7.01756924599585	639.513.203.985.603	136.959.694.774.889	511.683.087.736.893
6.57552882987259	738.288.375.193.019	257.552.882.987.259	100.059.813.815.381
7.32252231441417	552.378.052.266.128	152.378.052.266.128	701.563.361.899.095
6.60188597533481	724.536.494.062.291	166.642.622.752.952	897.203.201.711.495
6.77024650148834	880.520.633.984.214	130.536.045.275.894	717.982.957.067.508
7.11127687138646	82.007.119.549.684	249.236.703.874.197	115.131.486.008.785
7.24965146361899	728.281.832.755.419	463.074.163.097.449	861.780.257.526.699
6.93134935813544	712.903.002.597.514	-0.228521978642947	918.086.895.749.476
7.53487902424098	682.690.563.341.448	0.249476805378731	954.639.301.225.802
6.67206923366443	586.471.431.160.683	518.664.240.649.419	968.489.327.402.202
7.67015993561708	824.137.302.623.028	0.72764543027784	824.924.587.000.157

CCND3-mRNA	FST-mRNA	UTY-mRNA	NR4A1-mRNA
83.3238637150305	766.579.546.178.281	133.318.181.944.049	653.259.091.525.839
129.568323583068	841.640.392.505.397	258.398.366.120.078	346.992.091.646.962
95.3743113484624	166.905.044.859.809	59.608.944.592.789	102.825.429.422.561
160.065914627922	460.069.694.738.938	287.543.559.211.836	129.394.601.645.326
97.1327549146589	151.730.251.141.199	317.427.303.642.676	502.169.994.362.713
109.155915130465	447.333.297.478.056	24.714.546.821.992	144.992.008.022.353
138.263531629348	29.421.193.358.338	562.700.419.421.764	292.282.675.002.505
152.181741147208	155.720.851.406.446	247.737.718.146.618	392.841.238.775.351
122.051765860075	13.997.545.175.561	0.853508852168356	580.386.019.474.483
185.449044485673	113.528.100.951.165	118.877.592.619.021	747.740.057.573.643
101.97482588922	582.713.290.795.544	364.195.806.747.215	823.082.523.248.706
203.679918325912	302.621.992.472.036	165.593.429.533.262	304.277.926.767.368

MCM2-mRNA	PRDM1-mRNA	APH1B-mRNA	SPP1-mRNA
543.724.136.173.545	619.623.326.223.166	598.472.915.703.794	107.619.412.058.729
50.858.039.817.483	549.297.936.325.418	567.076.648.246.946	142.702.481.545.874
316.049.133.059.375	389.745.692.475.995	538.288.375.193.019	107.849.821.955.015
508.856.514.144.481	67.843.080.728.845	534.820.895.807.782	137.616.377.779.631
447.378.114.958.712	570.205.013.726.024	483.635.122.897.183	126.162.529.382.886
36.272.885.476.463	674.276.576.506.624	487.014.507.154.246	126.799.471.602.684
393.293.963.012.795	706.871.640.915.841	563.920.842.707.124	11.813.650.929.537
49.933.117.103.592	714.531.480.380.425	614.531.480.380.425	122.527.934.504.309
435.644.052.207.821	621.442.151.720.578	524.721.145.232.345	1.174.947.338.997
457.140.490.026.609	620.367.311.576.561	539.922.392.488.341	108.456.665.615.231
518.664.240.649.419	618.664.240.649.419	644.967.681.232.799	9.973.238.768.385
475.001.324.330.629	506.749.543.316.246	601.304.764.914.009	128.920.810.163.425

MCM2-mRNA	PRDM1-mRNA	APH1B-mRNA	SPP1-mRNA
433.284.091.318.159	733.250.000.692.268	633.261.364.234.232	173.646.931.982.123
339.609.281.186.388	450.351.438.094.993	509.413.921.779.583	197.593.539.166.793
894.134.168.891.835	149.022.361.481.972	417.262.612.149.523	176.442.475.994.655
340.259.878.400.673	110.225.031.031.204	407.353.375.550.101	13.888.833.149.197
222.199.112.549.873	520.580.777.973.989	285.684.573.278.408	627.871.206.605.213
12.357.273.410.996	107.096.369.561.965	292.455.470.726.906	656.212.409.035.259
152.732.970.985.907	134.244.242.919.192	498.391.800.059.276	359.967.496.881.522
318.519.923.331.366	141.564.410.369.496	70.782.205.184.748	488.043.304.748.837
204.842.124.520.406	74.255.270.138.647	379.811.439.214.919	344.305.470.964.715
237.755.185.238.043	737.041.074.237.932	422.015.453.797.526	184.022.513.374.245
364.195.806.747.215	728.391.613.494.429	874.069.936.193.316	100.518.042.662.231
26.908.932.299.155	335.326.694.804.854	64.581.437.517.972	760.156.638.272.437

ACVR1B-mRNA	SHC3-mRNA	IL7-mRNA	WEE1-mRNA
532.176.414.431.551	432.176.414.431.551	454.415.656.565.196	58.242.644.848.447
423.466.736.766.278	226.268.174.383.238	302.167.364.432.859	514.720.452.641.244
389.745.692.475.995	389.745.692.475.995	257.552.882.987.259	516.049.133.059.375
529.637.002.655.821	326.074.611.682.749	302.628.086.319.046	704.734.247.871.829
492.946.063.336.331	266.642.622.752.952	386.806.008.869.917	703.099.865.982.538
421.225.104.836.746	376.479.207.139.623	289.032.295.348.009	874.137.789.094.199
338.545.183.482.545	349.236.703.874.197	226.997.461.740.552	867.369.680.345.653
440.834.920.963.804	49.933.117.103.592	482.338.670.891.689	718.093.871.353.497
549.939.847.592.025	223.090.963.999.435	294.140.302.279.937	836.953.052.151.865
551.626.334.607.363	205.683.172.743.634	224.947.680.537.873	878.863.561.648.676
567.206.923.366.443	518.664.240.649.419	386.471.431.160.683	822.226.631.622.491
470.492.535.377.776	2.312.607.930.999	218.707.704.891.514	773.326.997.947.172

ACVR1B-mRNA	SHC3-mRNA	IL7-mRNA	WEE1-mRNA
399.954.545.832.146	199.977.272.916.073	233.306.818.402.085	566.602.273.262.207
188.261.666.744.628	479.882.679.937.288	812.109.150.663.103	354.374.902.107.536
149.022.361.481.972	149.022.361.481.972	59.608.944.592.789	357.653.667.556.734
392.976.197.589.509	95.847.853.070.612	814.706.751.100.202	132.270.037.237.445
304.730.211.496.969	634.854.607.285.352	146.016.559.675.631	130.780.049.100.783
18.535.910.116.494	135.930.007.520.956	741.436.404.659.761	427.973.569.134.162
104.501.506.464.042	112.540.083.884.353	482.314.645.218.654	408.359.732.951.794
212.346.615.554.244	318.519.923.331.366	283.128.820.738.992	145.103.520.628.733
452.359.691.649.229	469.429.868.692.596	768.157.966.951.521	330.734.680.215.238
457.678.731.583.232	416.071.574.166.575	475.510.370.476.085	442.224.644.542.759
509.874.129.446.101	364.195.806.747.215	145.678.322.698.886	298.640.561.532.716
260.809.651.514.887	496.780.288.599.784	455.381.931.216.469	212.787.556.950.241

**FANCE-mRNA**	**ALKBH2-mRNA**	**FN1-mRNA**	**EIF4EBP1-mRNA**
58.242.644.848.447	532.176.414.431.551	822.865.473.992.403	58.242.644.848.447
475.206.658.457.131	482.902.856.638.619	111.681.844.653.167	706.606.776.368.704
71.902.386.739.878	538.288.375.193.019	898.491.976.601.029	67.454.538.313.149
508.856.514.144.481	458.267.421.171.485	114.351.725.105.047	691.609.794.544.004
605.874.365.030.828	420.247.912.776.973	952.937.347.555.044	654.417.047.747.852
580.786.079.328.812	457.837.894.716.535	106.742.306.341.831	651.481.381.838.789
543.989.961.884.783	463.920.842.707.124	947.454.576.165.472	566.229.204.018.428
668.136.770.404.446	49.933.117.103.592	102.869.110.821.881	573.027.730.452.541
55.129.450.077.582	435.644.052.207.821	994.015.013.948.928	599.064.654.181.921
560.702.880.999.682	420.367.311.576.561	101.159.830.176.049	633.693.964.662.907
544.967.681.232.799	386.471.431.160.683	767.206.923.366.443	486.471.431.160.683
434.235.527.439.305	518.707.704.891.514	112.376.677.890.916	580.446.102.732.867

**FANCE-mRNA**	**ALKBH2-mRNA**	**FN1-mRNA**	**EIF4EBP1-mRNA**
566.602.273.262.207	399.954.545.832.146	29.996.590.937.411	566.602.273.262.207
269.472.581.810.939	284.238.202.732.086	230.122.202.056.081	133.998.009.859.412
146.041.914.252.333	417.262.612.149.523	506.676.029.038.707	10.729.610.026.702
340.259.878.400.673	23.961.963.267.653	276.904.447.520.998	120.768.294.868.971
66.659.733.764.962	184.107.836.112.752	738.970.762.880.149	933.236.272.709.468
560.196.394.631.819	238.907.285.945.923	163.404.345.404.737	914.438.232.413.706
434.083.180.696.789	249.195.900.029.638	711.414.101.697.515	506.430.377.479.587
102.634.197.517.885	318.519.923.331.366	12.493.059.215.108	53.086.653.888.561
456.627.235.910.071	204.842.124.520.406	982.388.688.845.778	635.864.094.865.425
487.398.129.737.987	184.260.268.559.483	110.972.232.709.856	808.367.629.809.345
437.034.968.096.658	145.678.322.698.886	20.394.965.177.844	291.356.645.397.772
202.851.951.178.245	364.305.544.973.175	241.476.618.616.879	558.877.824.674.758

**GNA11-mRNA**	**CDC7-mRNA**	**CSF1R-mRNA**	**LIFR-mRNA**
866.753.898.115.724	626.036.359.965.137	473.680.164.359.436	932.925.868.086.244
862.833.121.614.906	477.169.539.132.024	542.022.302.081.886	10.347.285.740.471
958.675.608.529.584	527.596.854.801.368	257.552.882.987.259	856.421.351.664.476
904.472.653.051.128	4.893.014.332.327	531.385.745.328.705	105.658.958.624.575
899.554.982.382.108	561.128.467.333.706	292.946.063.336.331	942.398.291.646.897
905.578.830.673.171	452.775.287.409.538	272.039.795.203.778	959.076.267.162.119
932.706.380.961.234	500.694.021.157.172	400.694.021.157.172	100.965.231.046.964
849.581.205.088.838	59.933.117.103.592	382.338.670.891.689	971.212.995.781.515
893.889.616.718.879	429.503.997.741.407	294.140.302.279.937	948.572.353.902.318
917.428.930.898.451	445.893.017.100.768	357.140.490.026.609	983.443.930.609.989
856.515.402.974.792	603.463.931.304.914	386.471.431.160.683	901.446.143.111.151
85.253.069.561.316	393.709.879.590.679	506.749.543.316.246	105.301.617.953.991

**GNA11-mRNA**	**CDC7-mRNA**	**CSF1R-mRNA**	**LIFR-mRNA**
406.620.454.929.349	766.579.546.178.281	266.636.363.888.098	643.260.227.880.035
395.718.640.686.748	273.163.987.041.226	428.203.006.713.272	130.269.690.576.822
768.955.385.246.978	387.458.139.853.129	59.608.944.592.789	37.851.679.816.421
528.121.670.419.072	297.128.344.518.897	39.776.859.024.304	151.583.379.631.173
510.423.104.257.423	488.838.047.609.721	761.825.528.742.422	686.912.685.082.751
532.186.574.900.229	230.669.103.671.926	659.054.581.919.788	771.093.860.846.151
642.282.335.882.841	321.543.096.812.436	160.771.548.406.218	109.485.424.464.635
360.989.246.442.214	637.039.846.662.732	141.564.410.369.496	838.769.131.439.264
490.767.589.996.805	196.307.035.998.722	768.157.966.951.521	71.694.743.582.142
577.745.100.128.444	219.923.546.345.189	118.877.592.619.021	912.979.911.314.084
378.763.639.017.103	655.552.452.144.987	145.678.322.698.886	517.158.045.581.045
368.445.380.711.507	153.173.922.318.267	335.326.694.804.854	147.874.932.573.203

MSH6-mRNA	CDC25A-mRNA	CYLD-mRNA	FZD7-mRNA
590.672.664.503.667	519.623.326.223.166	845.104.716.126.048	612.911.906.637.312
693.292.943.249.851	188.417.012.057.865	76.310.203.036.767	723.819.905.863.304
616.049.133.059.375	357.552.882.987.259	821.938.501.964.732	621.938.501.964.731
654.614.833.568.973	333.113.544.471.888	843.267.347.118.094	828.975.720.348.656
644.253.021.560.268	351.442.313.408.447	773.681.555.542.092	748.915.637.547.404
644.149.714.074.497	289.032.295.348.009	799.652.235.731.202	742.775.708.411.866
585.493.711.812.667	249.236.703.874.197	82.280.439.369.396	869.623.937.210.762
682.338.670.891.689	607.131.422.236.047	940.834.920.963.804	861.780.257.526.699
671.984.525.294.173	329.503.997.741.407	785.096.280.518.387	714.216.542.816.427
665.035.624.166.092	283.443.930.609.989	787.883.342.545.834	772.115.201.977.078
686.471.431.160.683	444.967.681.232.799	732.414.593.024.413	486.471.431.160.683
672.764.543.027.784	172.764.543.027.784	73.163.600.658.601	808.299.652.670.265

MSH6-mRNA	CDC25A-mRNA	CYLD-mRNA	FZD7-mRNA
599.931.818.748.219	366.625.000.346.134	349.960.227.603.128	699.920.455.206.256
122.185.513.122.494	369.140.523.028.683	198.228.460.866.403	150.978.473.918.731
715.307.335.113.468	119.217.889.185.578	298.044.722.963.945	745.111.807.409.862
934.516.567.438.467	100.640.245.724.143	345.531.510.319.556	312.943.240.275.549
869.750.811.980.932	114.273.829.311.363	213.311.148.047.878	179.663.853.861.755
86.912.822.990.672	741.436.404.659.761	255.383.650.493.918	172.178.009.526.545
578.777.574.262.385	562.700.419.421.764	299.838.937.777.597	414.790.594.888.043
113.251.528.295.597	672.430.949.255.106	67.950.916.977.358	392.841.238.775.351
105.408.343.242.792	98.153.517.999.361	230.874.144.511.541	141.255.715.033.863
100.451.565.763.073	713.265.555.714.128	235.377.633.385.662	211.007.726.898.763
116.542.658.159.109	218.517.484.048.329	160.246.154.968.774	291.356.645.397.772
105.979.794.901.287	331.186.859.066.523	159.383.675.925.764	271.159.240.860.715

SOCS3-mRNA	FGF17-mRNA	FGF10-mRNA	DLL4-mRNA
902.220.386.245.661	405.872.973.848.172	598.472.915.703.794	659.478.263.872.193
81.415.579.632.713	326.268.174.383.238	0.884170120578651	484.764.424.455.354
557.552.882.987.259	316.049.133.059.375	516.049.133.059.375	666.299.167.112.293
941.859.828.596.922	346.237.997.799.714	339.824.964.057.742	743.467.304.882.729
831.028.241.730.424	486.806.008.869.917	280.392.975.127.945	729.286.536.422.683
725.177.941.255.409	442.083.767.017.887	280.786.079.328.812	672.039.795.203.778
797.963.286.557.599	468.501.211.668.436	443.989.961.884.783	734.322.359.943.616
101.984.261.402.638	463.074.163.097.449	382.338.670.891.689	695.266.972.586.185
610.239.489.947.167	367.836.861.696.557	519.774.277.605.915	541.533.421.113.178
938.518.609.148.313	357.140.490.026.609	601.766.113.015.566	682.690.563.341.448
781.891.062.199.371	567.206.923.366.443	518.664.240.649.419	586.471.431.160.683
641.414.595.746.106	20.495.735.251.652	397.557.294.372.143	537.150.162.005.256

SOCS3-mRNA	FGF17-mRNA	FGF10-mRNA	DLL4-mRNA
51.994.090.958.179	166.647.727.430.061	633.261.364.234.232	966.556.819.094.353
282.392.500.116.943	959.765.359.874.576	184.570.261.514.342	287.929.607.962.373
476.871.556.742.312	894.134.168.891.835	357.653.667.556.734	101.335.205.807.741
684.353.670.924.169	110.225.031.031.204	105.432.638.377.673	173.005.374.792.455
317.427.303.642.676	292.033.119.351.262	698.340.068.013.887	156.809.087.999.482
152.406.372.068.951	214.192.739.123.931	700.245.493.289.774	105.448.733.107.166
252.411.330.997.762	257.234.477.449.949	217.041.590.348.394	16.237.926.389.028
117.498.460.606.682	247.737.718.146.618	141.564.410.369.496	123.868.859.073.309
687.074.625.995.527	128.026.327.825.253	367.008.806.432.393	426.754.426.084.178
668.686.458.481.995	118.877.592.619.021	647.882.879.773.666	113.528.100.951.165
225.801.400.183.273	509.874.129.446.101	364.195.806.747.215	582.713.290.795.544
852.806.162.096.297	413.983.573.833.154	157.313.758.056.598	413.983.573.833.154

**TCF3-mRNA**	**WNT16-mRNA**	**POLD1-mRNA**	**FANCL-mRNA**
505.872.973.848.172	532.176.414.431.551	373.680.164.359.436	605.872.973.848.172
657.905.031.337.784	0.562242025691289	384.764.424.455.354	576.191.437.052.765
557.552.882.987.259	778.498.219.550.154	389.745.692.475.995	57.454.538.313.149
642.263.379.920.438	0.938818021940123	346.237.997.799.714	604.734.247.871.829
650.436.946.942.055	166.642.622.752.952	280.392.975.127.945	686.806.008.869.917
599.652.235.731.202	0.720397952037781	36.272.885.476.463	688.026.928.881.617
653.050.216.762.874	0.685012116684361	426.997.461.740.552	632.886.830.645.909
573.027.730.452.541	463.074.163.097.449	463.074.163.097.449	482.338.670.891.689
636.393.505.862.513	0.356440522078209	267.836.861.696.557	677.710.257.055.093
658.932.680.826.336	124.947.680.537.873	257.140.490.026.609	632.629.240.242.956
544.967.681.232.799	444.967.681.232.799	486.471.431.160.683	686.471.431.160.683
661.028.847.963.968	0.312607930998996	3.312.607.930.999	613.703.636.641.554

**TCF3-mRNA**	**WNT16-mRNA**	**POLD1-mRNA**	**FANCL-mRNA**
333.295.454.860.122	399.954.545.832.146	133.318.181.944.049	666.590.909.720.244
95.607.395.464.429	147.656.209.211.473	143.964.803.981.186	542.636.568.852.164
476.871.556.742.312	220.553.094.993.319	149.022.361.481.972	536.480.501.335.101
857.838.284.981.977	191.695.706.141.224	110.225.031.031.204	661.350.186.187.223
907.842.088.418.053	317.427.303.642.676	698.340.068.013.887	116.813.247.740.505
638.459.126.234.794	164.763.645.479.947	12.357.273.410.996	117.806.006.518.162
924.436.403.335.754	160.771.548.406.218	192.925.858.087.462	803.857.742.031.091
53.086.653.888.561	247.737.718.146.618	247.737.718.146.618	283.128.820.738.992
823.636.042.342.464	128.026.327.825.253	640.131.639.126.267	109.675.887.503.634
962.908.500.214.072	237.755.185.238.043	594.387.963.095.106	802.423.750.178.394
437.034.968.096.658	218.517.484.048.329	291.356.645.397.772	116.542.658.159.109
977.001.234.246.243	124.195.072.149.946	993.560.577.199.569	703.772.075.516.361

HOXA11-mRNA	IFNA7-mRNA	MNAT1-mRNA	COL4A5-mRNA
605.872.973.848.172	473.680.164.359.436	590.672.664.503.667	543.724.136.173.545
-0.437757974308711	-143.775.797.430.871	552.802.631.035.338	426.268.174.383.238
316.049.133.059.375	257.552.882.987.259	438.288.375.193.019	157.552.882.987.259
252.378.052.266.128	-106.118.197.805.988	577.170.803.610.486	568.028.500.834.127
192.946.063.336.331	280.392.975.127.945	512.585.784.616.682	520.247.912.776.973
104.232.604.692.514	0.305360452758937	506.024.795.492.241	304.232.604.692.514
326.997.461.740.552	-0.314987883315639	514.444.373.532.166	300.694.021.157.172
563.074.163.097.449	414.531.480.380.425	614.531.480.380.425	463.074.163.097.449
-0.228521978642947	0.356440522078209	610.239.489.947.167	686.423.516.227.691
0.249476805378731	157.140.490.026.609	599.094.379.177.988	705.037.670.529.904
544.967.681.232.799	567.206.923.366.443	632.414.593.024.413	518.664.240.649.419
0.312607930998996	0.72764543027784	596.605.016.960.292	281.510.827.152.818

HOXA11-mRNA	IFNA7-mRNA	MNAT1-mRNA	COL4A5-mRNA
666.590.909.720.244	266.636.363.888.098	599.931.818.748.219	433.284.091.318.159
0.738281046057366	0.369140523028683	461.425.653.785.854	191.953.071.974.915
894.134.168.891.835	59.608.944.592.789	208.631.306.074.761	298.044.722.963.945
575.087.118.423.672	0.47923926535306	546.332.762.502.488	512.786.013.927.774
380.912.764.371.211	698.340.068.013.887	349.170.034.006.944	368.215.672.225.504
205.954.556.849.934	12.357.273.410.996	333.646.382.096.892	823.818.227.399.735
964.629.290.437.309	0.803857742031091	35.369.740.649.368	803.857.742.031.091
495.475.436.293.236	17.695.551.296.187	70.782.205.184.748	247.737.718.146.618
0.853508852168356	128.026.327.825.253	687.074.625.995.527	116.503.958.320.981
118.877.592.619.021	297.193.981.547.553	635.995.120.511.764	132.548.515.770.209
437.034.968.096.658	509.874.129.446.101	801.230.774.843.872	364.195.806.747.215
124.195.072.149.946	165.593.429.533.262	625.115.196.488.062	703.772.075.516.361

CARD11-mRNA	GDF6-mRNA	WHSC1L1-mRNA	RASAL1-mRNA
543.724.136.173.545	454.415.656.565.196	778.119.576.295.281	405.872.973.848.172
264.970.486.694.163	0.884170120578651	776.925.634.586.882	-143.775.797.430.871
457.552.882.987.259	416.049.133.059.375	803.496.044.850.989	157.552.882.987.259
384.570.861.754.864	263.925.774.008.122	775.259.921.315.716	126.074.611.682.749
520.247.912.776.973	266.642.622.752.952	818.684.847.605.596	0.929460633363313
484.968.096.898.275	130.536.045.275.894	814.875.812.474.207	104.232.604.692.514
500.694.021.157.172	268.501.211.668.436	789.933.123.748.513	200.694.021.157.172
440.834.920.963.804	563.074.163.097.449	788.947.589.937.466	382.338.670.891.689
419.774.277.605.915	329.503.997.741.407	831.063.683.246.508	209.340.611.624.442
324.947.680.537.873	370.890.842.401.603	818.021.414.294.162	0.834439306099887
486.471.431.160.683	518.664.240.649.419	807.416.767.723.578	386.471.431.160.683
218.707.704.891.514	172.764.543.027.784	763.152.727.601.402	0.72764543027784

CARD11-mRNA	GDF6-mRNA	WHSC1L1-mRNA	RASAL1-mRNA
433.284.091.318.159	233.306.818.402.085	21.997.500.020.768	166.647.727.430.061
627.538.889.148.761	184.570.261.514.342	218.162.049.109.952	0.369140523028683
238.435.778.371.156	178.826.833.778.367	262.279.356.208.272	298.044.722.963.945
143.771.779.605.918	623.011.044.958.978	215.657.669.408.877	23.961.963.267.653
368.215.672.225.504	634.854.607.285.352	291.398.264.743.976	190.456.382.185.606
288.336.379.589.907	24.714.546.821.992	283.805.379.339.209	205.954.556.849.934
321.543.096.812.436	643.086.193.624.873	238.745.749.383.234	401.928.871.015.545
212.346.615.554.244	495.475.436.293.236	237.120.387.368.906	141.564.410.369.496
183.504.403.216.197	98.153.517.999.361	317.505.293.006.629	426.754.426.084.178
95.102.074.095.217	130.765.351.880.923	290.061.325.990.412	178.316.388.928.532
291.356.645.397.772	364.195.806.747.215	269.504.896.992.939	145.678.322.698.886
455.381.931.216.469	331.186.859.066.523	198.298.131.866.081	165.593.429.533.262

IRAK3-mRNA	FLT3-mRNA	SETD2-mRNA	PITX2-mRNA
654.415.656.565.196	405.872.973.848.172	735.151.148.770.957	573.680.164.359.436
553.952.194.919.121	0.562242025691289	757.067.064.776.187	320.609.821.546.601
657.552.882.987.259	157.552.882.987.259	796.784.625.265.135	661.992.294.923.104
572.017.773.546.478	126.074.611.682.749	757.906.295.816.247	374.617.294.399.773
6.227.141.182.004	304.493.785.078.325	791.814.532.013.548	386.806.008.869.917
677.568.038.753.897	372.039.795.203.778	790.527.329.494.606	482.892.240.881.595
726.997.461.740.552	326.997.461.740.552	774.029.455.218.555	420.857.407.274.137
673.027.730.452.541	382.338.670.891.689	821.570.413.169.565	514.531.480.380.425
638.618.786.547.226	285.894.086.260.739	783.756.721.181.483	235.644.052.207.821
689.333.299.515.346	377.303.876.143.574	767.574.156.008.083	241.940.180.682.104
703.463.931.304.914	386.471.431.160.683	686.471.431.160.683	486.471.431.160.683
657.313.548.122.221	348.253.293.244.131	726.292.080.689.864	2.312.607.930.999

IRAK3-mRNA	FLT3-mRNA	SETD2-mRNA	PITX2-mRNA
933.227.273.608.341	166.647.727.430.061	16.331.477.288.146	533.272.727.776.195
465.117.059.016.141	147.656.209.211.473	190.107.369.359.772	922.851.307.571.707
953.743.113.484.624	298.044.722.963.945	250.357.567.289.714	983.547.585.781.019
527.163.191.888.366	23.961.963.267.653	191.216.466.875.871	134.186.994.298.857
749.128.436.596.716	825.310.989.470.958	241.879.605.375.719	146.016.559.675.631
109.567.824.244.165	131.810.916.383.958	239.731.104.173.323	284.217.288.452.908
154.340.686.469.969	964.629.290.437.309	21.382.615.938.027	184.887.280.667.151
106.173.307.777.122	141.564.410.369.496	297.285.261.775.942	35.391.102.592.374
836.438.675.124.989	725.482.524.343.103	228.740.372.381.119	512.105.311.301.014
118.877.592.619.021	136.709.231.511.874	204.469.459.304.717	534.949.166.785.596
131.110.490.428.997	145.678.322.698.886	116.542.658.159.109	291.356.645.397.772
952.162.219.816.253	111.775.564.934.951	1.535.879.058.921	496.780.288.599.784

**MYD88-mRNA**	**GNAS-mRNA**	**TCF7L1-mRNA**	**BRAF-mRNA**
649.168.914.575.783	888.654.876.309.904	573.680.164.359.436	784.532.610.037.253
779.106.071.618.717	105.153.471.762.826	627.648.754.335.741	721.687.805.421.926
70.990.907.859.296	901.847.232.572.132	638.288.375.193.019	755.280.875.337.251
78.636.305.255.459	102.618.727.822.818	647.019.948.245.643	780.918.274.152.353
704.493.785.078.325	895.182.844.639.177	7.227.141.182.004	777.912.636.027.888
69.102.225.109.178	912.127.738.831.996	701.501.870.092.941	798.484.055.226.438
704.256.412.130.245	876.716.115.803.823	672.940.623.604.281	788.468.446.152.072
663.074.163.097.449	931.523.980.524.656	621.570.413.169.565	784.575.452.194.534
691.102.937.375.585	944.567.028.950.274	718.086.895.749.476	771.692.185.773.496
706.325.799.659.577	967.048.966.115.796	752.560.121.065.297	757.140.490.026.609
744.967.681.232.799	790.910.843.096.528	732.414.593.024.413	750.857.050.138.155
746.573.768.989.833	991.437.871.940.671	745.215.928.339.779	73.088.460.122.028

**MYD88-mRNA**	**GNAS-mRNA**	**TCF7L1-mRNA**	**BRAF-mRNA**
899.897.728.122.329	473.279.545.901.373	533.272.727.776.195	229.973.863.853.484
22.148.431.381.721	146.364.217.380.873	775.195.098.360.234	148.763.630.780.559
137.100.572.563.415	518.597.817.957.265	834.525.224.299.046	187.768.175.467.285
232.910.282.961.587	122.781.099.783.454	886.592.640.903.161	224.283.976.185.232
132.049.758.315.353	495.186.593.682.575	149.825.687.319.343	219.659.694.120.732
120.277.461.200.361	55.690.112.172.222	129.339.461.701.758	253.324.104.925.418
131.832.669.693.099	435.690.896.180.851	106.109.221.948.104	236.334.176.157.141
990.950.872.586.472	637.039.846.662.732	743.213.154.439.854	230.042.166.850.431
120.344.748.155.738	697.316.732.221.547	145.096.504.868.621	2.103.899.320.595
133.737.291.696.399	814.905.897.403.392	184.260.268.559.483	190.204.148.190.434
174.813.987.238.663	240.369.232.453.162	160.246.154.968.774	182.097.903.373.607
176.770.986.026.757	964.995.710.605.081	175.115.051.731.424	158.555.708.778.098

PTPN5-mRNA	IL2RB-mRNA	NTHL1-mRNA	NSD1-mRNA
454.415.656.565.196	619.623.326.223.166	654.415.656.565.196	643.724.136.173.545
136.959.694.774.889	356.224.202.569.129	633.042.635.046.821	710.913.648.557.893
257.552.882.987.259	389.745.692.475.995	703.496.044.850.989	71.902.386.739.878
126.074.611.682.749	599.410.045.744.131	626.973.490.005.474	715.313.714.274.089
351.442.313.408.447	549.424.525.214.684	686.806.008.869.917	638.889.225.200.061
217.982.957.067.508	544.831.840.660.098	681.843.003.499.831	657.214.699.345.384
126.997.461.740.552	57.724.749.579.347	671.843.511.822.181	698.879.286.486.146
482.338.670.891.689	528.281.832.755.419	63.469.486.649.739	614.531.480.380.425
223.090.963.999.435	542.968.950.410.885	674.302.157.530.783	697.604.916.560.626
357.140.490.026.609	494.991.652.351.982	642.938.589.539.367	71.074.578.005.063
386.471.431.160.683	544.967.681.232.799	718.664.240.649.419	632.414.593.024.413
1.312.607.930.999	546.911.241.667.899	657.313.548.122.221	729.369.946.844.893

PTPN5-mRNA	IL2RB-mRNA	NTHL1-mRNA	NSD1-mRNA
233.306.818.402.085	733.250.000.692.268	933.227.273.608.341	866.568.182.636.317
258.398.366.120.078	118.124.967.369.179	804.726.340.202.529	138.058.555.612.728
59.608.944.592.789	149.022.361.481.972	131.139.678.104.136	146.041.914.252.333
23.961.963.267.653	63.738.822.291.957	771.575.217.218.427	142.334.061.809.859
114.273.829.311.363	4.507.467.711.726	116.813.247.740.505	838.008.081.616.665
453.100.025.069.854	436.623.660.521.859	112.863.097.153.764	951.510.052.646.693
241.157.322.609.327	546.623.264.581.142	105.305.364.206.073	127.009.523.240.912
283.128.820.738.992	389.302.128.516.114	813.995.359.624.602	70.782.205.184.748
469.429.868.692.596	43.102.197.034.502	107.115.360.947.129	125.892.555.694.833
118.877.592.619.021	309.081.740.809.455	861.862.546.487.904	137.898.007.438.065
145.678.322.698.886	437.034.968.096.658	145.678.322.698.886	801.230.774.843.872
248.390.144.299.892	442.962.424.001.475	952.162.219.816.253	156.899.774.482.765

LRP2-mRNA	PDGFC-mRNA	FGFR3-mRNA	NPM2-mRNA
405.872.973.848.172	654.415.656.565.196	914.619.257.973.206	173.680.164.359.436
188.417.012.057.865	823.289.827.480.973	105.233.299.156.886	202.167.364.432.859
416.049.133.059.375	713.011.768.155.023	10.844.655.509.022	389.745.692.475.995
174.617.294.399.773	886.659.598.402.246	956.269.951.195.358	284.570.861.754.864
134.449.813.264.216	759.242.564.608.574	942.664.717.399.603	404.493.785.078.325
204.232.604.692.514	738.573.386.922.296	970.481.641.083.892	352.775.287.409.538
314.444.373.532.166	85.336.350.571.137	887.976.897.110.661	338.545.183.482.545
463.074.163.097.449	728.281.832.755.419	718.093.871.353.497	414.531.480.380.425
357.883.294.341.466	758.846.164.461.243	884.159.896.583.388	285.894.086.260.739
370.890.842.401.603	744.423.365.980.098	876.320.440.133.117	283.443.930.609.989
544.967.681.232.799	795.217.715.285.717	93.565.674.079.365	518.664.240.649.419
172.764.543.027.784	768.184.174.066.471	924.629.858.595.123	153.500.035.233.544

LRP2-mRNA	PDGFC-mRNA	FGFR3-mRNA	NPM2-mRNA
166.647.727.430.061	933.227.273.608.341	566.602.273.262.207	333.295.454.860.122
369.140.523.028.683	300.849.526.268.377	147.176.326.531.536	406.054.575.331.551
178.826.833.778.367	140.081.019.793.054	183.893.594.068.754	149.022.361.481.972
335.467.485.747.142	46.677.904.445.388	756.239.560.727.129	71.885.889.802.959
253.941.842.914.141	192.995.800.614.747	688.182.394.297.321	165.062.197.894.191
411.909.113.699.867	167.235.100.162.146	834.527.864.355.931	115.334.551.835.963
8.842.435.162.342	370.578.419.076.333	47.106.063.683.022	104.501.506.464.042
247.737.718.146.618	155.720.851.406.446	145.103.520.628.733	17.695.551.296.187
11.949.123.930.357	192.466.246.163.964	458.761.008.040.492	725.482.524.343.103
130.765.351.880.923	174.155.673.186.866	434.497.601.022.523	713.265.555.714.128
437.034.968.096.658	247.653.148.588.106	655.552.452.144.986	364.195.806.747.215
331.186.859.066.523	205.335.852.621.244	607.313.902.813.237	289.788.501.683.208

SKP2-mRNA	SYK-mRNA	MAML2-mRNA	NODAL-mRNA
698.472.915.703.794	632.176.414.431.551	519.623.326.223.166	58.242.644.848.447
664.439.106.704.516	572.211.336.246.968	603.797.545.665.769	17.321.670.271.336
686.093.104.873.484	582.345.634.331.618	582.345.634.331.618	389.745.692.475.995
701.028.038.449.675	555.352.786.605.533	638.176.151.778.885	174.617.294.399.773
719.624.717.405.821	483.635.122.897.183	690.674.055.686.323	398.835.432.241.688
74.346.434.697.039	521.225.104.836.746	635.702.257.258.143	311.271.537.481.654
705.133.433.093.018	520.857.407.274.137	572.940.623.604.281	368.501.211.668.436
540.834.920.963.804	482.338.670.891.689	614.531.480.380.425	591.084.955.016.723
708.436.097.664.141	507.525.876.953.416	6.341.333.629.688	109.340.611.624.442
704.389.267.172.884	453.487.902.424.098	657.140.490.026.609	0.249476805378731
618.664.240.649.419	286.471.431.160.683	656.515.402.974.792	486.471.431.160.683
681.510.827.152.818	542.808.514.841.893	649.582.975.505.477	0.312607930998996

SKP2-mRNA	SYK-mRNA	MAML2-mRNA	NODAL-mRNA
126.652.272.846.846	799.909.091.664.293	366.625.000.346.134	566.602.273.262.207
100.037.081.740.773	527.870.947.931.017	657.070.130.991.056	332.226.470.725.815
116.237.441.955.939	566.284.973.631.495	566.284.973.631.495	149.022.361.481.972
128.915.362.379.973	469.654.480.045.999	833.876.321.714.324	335.467.485.747.142
146.651.414.282.916	285.684.573.278.408	119.987.520.776.932	158.713.651.821.338
173.001.827.753.944	370.718.202.329.881	819.699.136.262.736	865.009.138.769.722
13.263.652.743.513	369.774.561.334.302	53.054.610.974.052	128.617.238.724.975
424.693.231.108.488	283.128.820.738.992	70.782.205.184.748	601.648.744.070.358
135.707.907.494.769	337.135.996.606.501	810.833.409.559.939	213.377.213.042.089
131.954.127.807.114	231.811.305.607.092	95.102.074.095.217	118.877.592.619.021
728.391.613.494.429	72.839.161.349.443	946.909.097.542.759	291.356.645.397.772
112.603.532.082.618	43.054.291.678.648	902.484.190.956.275	124.195.072.149.946

**PRKACG-mRNA**	**EPOR-mRNA**	**HGF-mRNA**	**MMP7-mRNA**
519.623.326.223.166	698.472.915.703.794	738.065.783.336.908	519.623.326.223.166
0.562242025691289	564.970.486.694.163	423.466.736.766.278	17.321.670.271.336
503.496.044.850.989	703.496.044.850.989	616.049.133.059.375	503.496.044.850.989
226.074.611.682.749	62.875.461.761.712	562.531.854.912.334	310.874.302.338.244
386.806.008.869.917	642.131.372.969.299	737.240.412.921.204	398.835.432.241.688
336.425.414.181.251	698.718.449.273.268	66.976.778.755.377	317.982.957.067.508
37.724.749.579.347	707.732.953.946.312	541.293.257.124.756	249.236.703.874.197
563.074.163.097.449	668.136.770.404.446	49.933.117.103.592	382.338.670.891.689
135.644.052.207.821	588.000.247.813.522	620.610.624.899.378	135.644.052.207.821
157.140.490.026.609	569.242.030.122.746	606.965.576.779.392	205.683.172.743.634
603.463.931.304.914	703.463.931.304.914	632.414.593.024.413	586.471.431.160.683
0.312607930998996	614.549.794.516.374	565.838.276.784.073	20.495.735.251.652

**PRKACG-mRNA**	**EPOR-mRNA**	**HGF-mRNA**	**MMP7-mRNA**
366.625.000.346.134	126.652.272.846.846	166.647.727.430.061	366.625.000.346.134
147.656.209.211.473	502.031.111.319.009	188.261.666.744.628	332.226.470.725.815
32.784.919.526.034	131.139.678.104.136	715.307.335.113.468	32.784.919.526.034
47.923.926.535.306	781.160.002.525.488	493.616.443.313.652	862.630.677.635.508
146.016.559.675.631	857.053.719.835.225	165.697.052.501.477	158.713.651.821.338
102.977.278.424.967	126.868.007.019.559	103.801.096.652.367	906.200.050.139.708
136.655.816.145.285	135.048.100.661.223	426.044.603.276.478	562.700.419.421.764
495.475.436.293.236	102.634.197.517.885	318.519.923.331.366	141.564.410.369.496
256.052.655.650.507	588.921.107.996.166	738.285.157.125.628	256.052.655.650.507
297.193.981.547.553	517.117.527.892.742	67.165.839.829.747	416.071.574.166.575
655.552.452.144.987	131.110.490.428.997	801.230.774.843.872	582.713.290.795.544
124.195.072.149.946	707.911.911.254.693	505.059.960.076.448	413.983.573.833.154

LEPR-mRNA	VEGFA-mRNA	SRSF2-mRNA	KMT2C-mRNA
788.654.876.309.904	881.361.724.064.519	874.802.889.901.761	802.220.386.245.661
69.065.379.336.071	820.970.045.214.621	815.469.906.295.937	778.141.054.615.345
57.454.538.313.149	891.537.883.275.721	947.034.659.318.053	861.992.294.923.104
686.955.535.950.301	915.677.571.980.424	888.864.473.269.923	78.426.998.676.763
767.988.848.733.608	923.628.183.586.047	887.977.350.926.296	807.241.858.720.536
922.918.311.410.229	98.733.165.281.744	881.974.576.244.097	827.306.704.955.205
946.637.183.020.902	920.464.836.952.758	932.163.673.722.801	802.040.247.137.829
668.136.770.404.446	998.829.363.559.258	929.912.013.988.329	829.912.013.988.329
882.404.607.216.121	887.738.652.992.821	843.325.611.912.904	811.132.802.424.168
860.262.363.087.681	930.204.485.618.289	840.429.491.443.084	807.919.954.046.479
632.414.593.024.413	856.515.402.974.792	89.939.973.285.518	818.664.240.649.419
100.461.882.399.806	760.722.867.989.062	785.692.844.722.281	794.196.455.107.861

LEPR-mRNA	VEGFA-mRNA	SRSF2-mRNA	KMT2C-mRNA
236.639.772.950.687	449.948.864.061.165	429.951.136.769.557	259.970.454.790.895
119.970.669.984.322	296.050.699.469.004	284.976.483.778.143	220.007.751.725.095
536.480.501.335.101	482.832.451.201.591	709.346.440.654.189	393.419.034.312.407
116.934.380.746.147	570.773.965.035.495	473.967.633.434.176	229.555.608.104.116
205.058.038.153.169	603.111.876.921.085	471.062.118.605.731	269.178.353.488.989
600.151.578.660.707	937.917.051.894.597	451.864.297.728.754	309.343.744.388.601
70.739.481.298.736	590.031.582.650.821	639.870.762.656.749	259.646.050.676.042
102.634.197.517.885	101.572.464.440.113	629.961.626.144.257	314.980.813.072.129
453.213.200.501.397	470.283.377.544.765	345.671.085.128.185	276.536.868.102.547
388.729.727.864.199	631.240.016.807.003	338.801.138.964.211	270.446.523.208.273
801.230.774.843.872	378.763.639.017.103	5.098.741.294.461	291.356.645.397.772
105.731.404.756.987	194.986.263.275.416	231.830.801.346.566	245.906.242.856.893

CACNA1E-mRNA	XPA-mRNA	FGF16-mRNA	CDKN1A-mRNA
626.036.359.965.137	654.415.656.565.196	505.872.973.848.172	702.220.386.245.661
288.417.012.057.865	685.686.277.458.291	156.224.202.569.129	796.950.978.993.602
516.049.133.059.375	738.288.375.193.019	457.552.882.987.259	730.344.928.443.579
193.881.802.194.012	636.508.277.664.222	174.617.294.399.773	840.642.357.202.312
398.835.432.241.688	675.388.906.877.986	359.242.564.608.574	676.235.064.752.805
472.039.795.203.778	666.291.245.737.702	296.832.546.548.137	687.521.606.108.989
407.732.953.946.312	629.972.196.079.957	314.444.373.532.166	786.988.745.959.264
573.027.730.452.541	591.084.955.016.723	382.338.670.891.689	903.284.007.454.584
401.940.553.480.064	685.894.086.260.739	157.883.294.341.466	636.393.505.862.513
183.443.930.609.989	657.140.490.026.609	157.140.490.026.609	829.932.535.482.929
656.515.402.974.792	544.967.681.232.799	486.471.431.160.683	732.414.593.024.413
425.120.738.633.485	666.428.336.928.041	153.500.035.233.544	779.104.051.156.635

CACNA1E-mRNA	XPA-mRNA	FGF16-mRNA	CDKN1A-mRNA
766.579.546.178.281	933.227.273.608.341	333.295.454.860.122	129.985.227.395.448
738.281.046.057.366	115.910.124.231.006	295.312.418.422.946	250.646.415.136.476
357.653.667.556.734	166.905.044.859.809	238.435.778.371.156	157.963.703.170.891
383.391.412.282.448	824.291.536.407.263	335.467.485.747.142	339.301.399.869.966
158.713.651.821.338	10.792.528.323.851	120.622.375.384.217	108.560.137.845.795
263.621.832.767.915	101.329.641.970.167	782.627.316.029.748	117.394.097.404.462
168.810.125.826.529	787.780.587.190.469	8.842.435.162.342	233.922.602.931.047
53.086.653.888.561	601.648.744.070.358	141.564.410.369.496	523.788.318.367.135
162.166.681.911.988	116.077.203.894.896	298.728.098.258.925	823.636.042.342.464
356.632.777.857.064	95.102.074.095.217	297.193.981.547.553	315.025.620.440.406
946.909.097.542.759	437.034.968.096.658	291.356.645.397.772	160.246.154.968.774
190.432.443.963.251	101.425.975.589.123	289.788.501.683.208	221.481.212.000.737

SPRY2-mRNA	HMGA1-mRNA	EFNA3-mRNA	EFNA1-mRNA
764.369.223.920.288	75.696.916.577.591	649.168.914.575.783	598.472.915.703.794
688.417.012.057.865	539.513.203.985.603	360.663.614.504.974	561.752.446.119.248
748.241.942.548.111	693.308.083.449.067	548.241.942.548.111	627.596.854.801.368
734.820.895.807.783	547.797.683.304.815	384.570.861.754.864	706.810.103.888.509
677.076.288.734.425	512.585.784.616.682	409.938.563.480.562	639.978.056.814.335
676.479.207.139.624	424.395.990.809.479	396.832.546.548.137	623.609.779.032.182
769.623.937.210.762	511.127.687.138.646	463.920.842.707.124	561.574.945.424.725
105.782.742.110.804	80.132.112.677.969	621.570.413.169.565	646.724.289.869.161
779.661.758.363.556	529.503.997.741.407	294.140.302.279.937	516.379.544.413.581
770.068.791.721.106	522.675.672.887.865	383.443.930.609.989	604.389.267.172.884
756.515.402.974.792	656.515.402.974.792	677.160.490.721.535	644.967.681.232.799
730.129.261.777.116	421.949.852.660.751	30.495.735.251.652	458.562.642.540.541

SPRY2-mRNA	HMGA1-mRNA	EFNA3-mRNA	EFNA1-mRNA
199.977.272.916.073	189.978.409.270.269	899.897.728.122.329	633.261.364.234.232
118.124.967.369.179	420.820.196.252.699	121.816.372.599.465	490.956.895.628.149
178.826.833.778.367	122.198.336.415.217	447.067.084.445.918	774.916.279.706.257
162.941.350.220.041	445.692.516.778.346	143.771.779.605.918	134.186.994.298.857
109.194.992.453.081	349.170.034.006.944	171.410.743.967.045	844.356.627.689.518
108.744.006.016.765	189.478.192.301.939	15.652.546.320.595	753.793.678.070.757
207.395.297.444.021	345.658.829.073.369	249.195.900.029.638	490.353.222.638.966
152.889.563.199.056	25.835.504.892.433	743.213.154.439.854	88.477.756.480.935
222.339.055.989.857	392.614.071.997.444	768.157.966.951.521	35.847.371.791.071
208.035.787.083.287	374.464.416.749.917	142.653.111.142.826	659.770.639.035.568
189.381.819.508.552	946.909.097.542.759	109.258.742.024.164	874.069.936.193.316
157.727.741.630.432	186.292.608.224.919	827.967.147.666.308	240.110.472.823.229

CDC14A-mRNA	ZAK-mRNA	RAD21-mRNA	ITGA3-mRNA
605.872.973.848.172	519.623.326.223.166	773.680.164.359.436	619.623.326.223.166
626.268.174.383.238	482.902.856.638.619	847.961.410.516.813	490.209.202.857.591
582.345.634.331.618	627.596.854.801.368	761.992.294.923.104	616.049.133.059.375
650.867.363.027.107	5.167.636.712.436	837.135.992.232.838	702.628.086.319.046
695.920.797.675.736	5.227.141.182.004	792.192.696.067.791	669.322.628.687.323
629.025.356.036.873	521.225.104.836.746	843.120.438.573.713	576.479.207.139.623
600.694.021.157.172	493.293.963.012.795	831.800.731.382.732	549.236.703.874.196
652.382.642.705.798	340.834.920.963.804	803.284.007.454.584	49.933.117.103.592
743.325.611.912.904	479.384.583.438.551	855.283.773.488.171	697.604.916.560.626
641.940.180.682.104	596.372.232.304.485	888.247.200.252.169	705.037.670.529.904
790.910.843.096.528	518.664.240.649.419	744.967.681.232.799	677.160.490.721.535
586.719.678.267.663	491.746.998.915.786	875.278.499.255.635	549.582.975.505.477

CDC14A-mRNA	ZAK-mRNA	RAD21-mRNA	ITGA3-mRNA
666.590.909.720.244	366.625.000.346.134	213.309.091.110.478	733.250.000.692.268
767.812.287.899.661	284.238.202.732.086	356.958.885.768.737	299.003.823.653.233
566.284.973.631.495	774.916.279.706.257	196.709.517.156.204	715.307.335.113.468
910.554.604.170.814	359.429.449.014.795	331.154.332.358.964	130.353.080.176.032
124.431.503.027.929	374.564.218.298.358	242.514.459.983.005	103.481.300.987.512
782.627.316.029.748	370.718.202.329.881	345.179.837.280.489	543.720.030.083.825
643.086.193.624.873	305.465.941.971.814	319.131.523.586.343	450.160.335.537.411
920.168.667.401.724	106.173.307.777.122	261.894.159.183.568	318.519.923.331.366
172.835.542.564.092	277.390.376.954.716	375.543.894.954.077	125.892.555.694.833
855.918.666.856.953	624.107.361.249.862	471.944.042.697.514	132.548.515.770.209
240.369.232.453.162	364.195.806.747.215	174.813.987.238.663	109.258.742.024.164
583.716.839.104.747	302.208.008.898.202	431.370.883.934.146	451.242.095.478.137

TTK-mRNA	GATA2-mRNA	PLAT-mRNA	FGF7-mRNA
612.911.906.637.312	590.672.664.503.667	75.696.916.577.591	638.065.783.336.908
356.224.202.569.129	326.268.174.383.238	869.152.504.263.626	449.297.936.325.418
438.288.375.193.019	389.745.692.475.995	803.496.044.850.989	816.049.133.059.375
422.422.024.080.237	644.661.266.213.882	102.114.478.069.165	580.918.274.152.353
359.242.564.608.574	669.322.628.687.323	881.413.994.988.167	804.840.170.608.682
357.837.894.716.535	644.149.714.074.497	905.019.429.025.848	872.039.795.203.778
414.444.373.532.166	694.239.995.937.701	864.501.404.875.244	838.197.964.291.865
528.281.832.755.419	514.531.480.380.425	84.527.433.289.965	563.074.163.097.449
177.147.802.135.705	708.436.097.664.141	905.456.637.438.106	752.636.552.352.052
294.991.652.351.982	689.333.299.515.346	989.333.299.515.346	814.126.050.859.704
632.414.593.024.413	567.206.923.366.443	818.664.240.649.419	686.471.431.160.683
20.495.735.251.652	532.755.827.246.497	971.063.900.497.215	824.728.168.312.105

TTK-mRNA	GATA2-mRNA	PLAT-mRNA	FGF7-mRNA
699.920.455.206.256	599.931.818.748.219	189.978.409.270.269	833.238.637.150.305
118.124.967.369.179	959.765.359.874.576	413.437.385.792.125	225.175.719.047.497
208.631.306.074.761	149.022.361.481.972	262.279.356.208.272	286.122.934.045.387
186.903.313.487.693	872.215.462.942.569	118.563.794.248.347	56.070.994.046.308
120.622.375.384.217	103.481.300.987.512	450.111.916.565.315	264.734.371.237.992
119.453.642.972.962	86.912.822.990.672	530.127.029.331.729	421.794.932.428.664
17.684.870.324.684	122.990.234.530.757	400.321.155.531.484	333.600.962.942.903
389.302.128.516.114	35.391.102.592.374	350.371.915.664.503	495.475.436.293.236
341.403.540.867.343	135.707.907.494.769	531.736.014.900.886	184.357.912.068.365
772.704.352.023.638	118.877.592.619.021	951.020.740.952.171	282.334.282.470.176
801.230.774.843.872	509.874.129.446.101	291.356.645.397.772	116.542.658.159.109
413.983.573.833.154	401.564.066.618.159	837.902.753.438.304	303.863.943.193.535

**TLR4-mRNA**	**WIF1-mRNA**	**WNT2B-mRNA**	**ERBB2-mRNA**
619.623.326.223.166	794.625.500.922.331	519.623.326.223.166	664.369.223.920.288
498.850.678.039.339	600.518.552.154.002	552.802.631.035.338	671.198.914.519.597
548.241.942.548.111	657.552.882.987.259	47.454.538.313.149	738.288.375.193.019
630.514.023.618.594	108.264.194.464.488	514.827.138.756.907	761.124.336.391.162
639.978.056.814.335	832.177.805.614.207	527.523.547.020.504	748.915.637.547.404
642.775.708.411.866	10.216.252.978.925	450.175.766.556.244	725.567.332.865.858
616.074.554.765.076	100.304.173.634.022	489.446.548.231.331	736.449.221.618.981
540.834.920.963.804	718.093.871.353.497	582.338.670.891.689	482.338.670.891.689
564.184.274.094.046	10.058.035.782.965	444.390.336.332.855	845.797.854.854.027
608.236.681.954.347	830.204.485.618.289	477.303.876.143.574	79.807.958.364.038
544.967.681.232.799	818.664.240.649.419	586.471.431.160.683	711.264.182.505.042
591.746.998.915.786	106.756.475.612.555	40.495.735.251.652	705.407.491.740.014

**TLR4-mRNA**	**WIF1-mRNA**	**WNT2B-mRNA**	**ERBB2-mRNA**
733.250.000.692.268	24.663.863.659.649	366.625.000.346.134	999.886.364.580.366
317.460.849.804.667	642.304.510.069.908	461.425.653.785.854	104.835.908.540.146
447.067.084.445.918	953.743.113.484.624	26.824.025.066.755	166.905.044.859.809
790.744.787.832.549	181.583.757.642.274	354.637.056.361.264	195.529.620.264.048
844.356.627.689.518	319.966.722.071.817	387.261.310.444.065	179.663.853.861.755
860.890.047.632.723	118.959.352.036.522	226.550.012.534.927	152.818.281.182.651
715.433.390.407.671	104.581.892.238.245	297.427.364.551.504	164.790.837.116.374
424.693.231.108.488	145.103.520.628.733	566.257.641.477.984	283.128.820.738.992
499.302.678.518.489	106.603.255.635.828	217.644.757.302.931	351.645.647.093.363
677.602.277.928.421	315.620.008.403.502	273.418.463.023.749	25.261.488.431.542
437.034.968.096.658	291.356.645.397.772	582.713.290.795.544	138.394.406.563.942
604.416.017.796.405	163.564.910.021.479	165.593.429.533.262	132.888.727.200.442

DDIT3-mRNA	U2AF1-mRNA	FBXW7-mRNA	ARID2-mRNA
673.680.164.359.436	872.548.633.036.652	490.672.664.503.667	816.306.639.829.646
69.065.379.336.071	886.716.369.527.296	563.905.762.274.212	783.836.643.096.553
764.161.802.033.036	891.537.883.275.721	548.241.942.548.111	831.699.581.627.374
745.845.427.478.334	983.060.172.515.843	472.017.773.546.478	803.157.516.285.998
756.366.665.310.432	937.102.157.516.192	547.378.114.958.712	792.946.063.336.331
751.805.947.789.154	941.475.583.925.923	567.459.426.242.466	796.595.065.829.346
72.007.119.549.684	951.314.860.087.847	595.179.865.737.926	806.871.640.915.841
818.093.871.353.497	927.459.782.074.922	552.382.642.705.798	721.570.413.169.565
693.134.935.813.544	908.549.472.525.841	571.399.252.669.629	810.909.992.334.956
719.784.403.696.341	927.876.403.234.698	503.083.651.890.339	788.610.142.592.238
718.664.240.649.419	750.857.050.138.155	544.967.681.232.799	79.939.973.285.518
589.757.043.172.015	856.211.648.026.206	653.500.035.233.544	740.007.077.224.934

DDIT3-mRNA	U2AF1-mRNA	FBXW7-mRNA	ARID2-mRNA
106.654.545.555.239	423.285.227.672.355	29.996.590.937.411	286.634.091.179.705
119.970.669.984.322	466.962.761.631.284	498.339.706.088.722	228.867.124.277.784
199.689.964.385.843	482.832.451.201.591	447.067.084.445.918	318.907.853.571.421
175.880.810.384.573	910.554.604.170.815	263.581.595.944.183	261.664.638.882.771
189.186.672.971.035	662.153.355.398.622	444.398.225.099.746	243.784.169.197.575
183.299.555.596.441	68.253.340.140.068	510.767.300.987.835	250.028.832.015.819
14.710.596.679.169	730.706.687.506.262	61.897.046.136.394	268.488.485.838.384
290.207.041.257.467	619.344.295.366.545	460.084.333.700.862	148.642.630.887.971
122.051.765.860.075	543.258.384.405.159	524.907.944.083.539	276.110.113.676.463
146.813.826.884.491	621.135.421.434.387	326.913.379.702.309	236.566.409.311.852
145.678.322.698.886	182.097.903.373.607	437.034.968.096.658	25.493.706.472.305
596.136.346.319.741	37.796.700.290.967	927.323.205.386.264	168.905.298.123.927

CREBBP-mRNA	CHEK2-mRNA	CCNA1-mRNA	IL1R1-mRNA
554.415.656.565.196	598.472.915.703.794	532.176.414.431.551	626.036.359.965.137
502.167.364.432.859	360.663.614.504.974	27.321.670.271.336	905.609.747.493.211
527.596.854.801.368	60.990.907.859.296	457.552.882.987.259	60.990.907.859.296
474.617.294.399.773	449.340.687.361.776	274.617.294.399.773	802.893.044.160.441
425.138.872.825.068	5.227.141.182.004	447.378.114.958.712	707.920.775.286.799
467.459.426.242.466	504.232.604.692.514	357.837.894.716.535	694.439.962.623.589
507.732.953.946.312	485.493.711.812.667	349.236.703.874.197	711.127.687.138.646
514.531.480.380.425	49.933.117.103.592	528.281.832.755.419	775.412.404.647.977
488.000.247.813.522	394.140.302.279.937	352.636.552.352.052	66.723.448.293.378
486.418.664.949.394	433.693.964.662.907	555.325.755.355.583	690.052.849.655.766
518.664.240.649.419	603.463.931.304.914	567.206.923.366.443	586.471.431.160.683
483.616.988.705.601	242.808.514.841.893	2.312.607.930.999	750.243.248.987.901

CREBBP-mRNA	CHEK2-mRNA	CCNA1-mRNA	IL1R1-mRNA
466.613.636.804.171	633.261.364.234.232	399.954.545.832.146	766.579.546.178.281
324.843.660.265.241	121.816.372.599.465	664.452.941.451.629	532.300.634.207.361
387.458.139.853.129	685.502.862.817.074	238.435.778.371.156	685.502.862.817.074
268.373.988.597.714	225.242.454.715.938	670.934.971.494.284	261.185.399.617.418
190.456.382.185.606	374.564.218.298.358	222.199.112.549.873	13.522.403.135.178
255.383.650.493.918	329.527.290.959.894	119.453.642.972.962	12.316.082.499.626
337.620.251.653.058	289.388.787.131.193	112.540.083.884.353	138.263.531.629.348
35.391.102.592.374	318.519.923.331.366	389.302.128.516.114	215.885.725.813.481
294.460.553.998.083	153.631.593.390.304	115.223.695.042.728	101.994.307.834.119
291.250.101.916.602	202.091.907.452.336	469.566.490.845.134	119.471.980.582.116
364.195.806.747.215	655.552.452.144.987	509.874.129.446.101	582.713.290.795.544
285.648.665.944.876	5.381.786.459.831	496.780.288.599.784	181.324.805.338.921

CASP8-mRNA	SF3B1-mRNA	NBN-mRNA	FGF14-mRNA
769.099.795.398.123	86.194.446.929.562	712.911.906.637.312	543.724.136.173.545
701.345.313.752.362	863.638.348.844.379	745.705.978.899.923	114.720.452.641.244
786.093.104.873.484	941.473.261.796.953	752.972.514.025.947	489.745.692.475.995
768.028.500.834.127	954.151.688.695.809	726.074.611.682.748	466.673.847.650.332
795.182.844.639.177	986.511.681.319.844	737.240.412.921.204	455.395.149.827.111
802.189.414.702.033	976.683.990.004.509	743.807.437.510.418	280.786.079.328.812
806.439.048.375.562	977.512.453.634.865	705.133.433.093.018	285.493.711.812.667
663.074.163.097.449	884.575.452.194.534	695.266.972.586.185	607.131.422.236.047
771.692.185.773.496	930.966.695.340.947	75.163.118.588.566	60.568.802.402.193
726.628.509.306.529	937.489.027.586.205	752.093.983.328.311	370.890.842.401.603
725.703.173.438.559	818.664.240.649.419	711.264.182.505.042	544.967.681.232.799
697.557.294.372.143	824.531.481.841.165	761.028.847.963.968	242.808.514.841.893

CASP8-mRNA	SF3B1-mRNA	NBN-mRNA	FGF14-mRNA
206.643.182.013.276	393.288.636.734.944	139.984.091.041.251	433.284.091.318.159
129.199.183.060.039	39.793.348.382.492	175.710.888.961.653	22.148.431.381.721
232.474.883.911.877	682.522.415.587.434	184.787.728.237.646	298.044.722.963.945
20.511.440.557.111	745.217.057.624.009	153.356.564.912.979	253.996.810.637.122
247.593.296.841.287	932.601.418.102.183	165.697.052.501.477	23.489.620.469.558
259.914.650.744.616	871.187.775.475.218	173.413.736.867.644	700.245.493.289.774
267.684.628.096.353	876.204.938.813.888	13.263.652.743.513	723.471.967.827.982
990.950.872.586.472	460.084.333.700.862	123.868.859.073.309	672.430.949.255.106
2.103.899.320.595	634.583.831.587.173	183.077.648.790.112	665.736.904.691.318
153.946.482.441.633	663.931.354.777.234	183.665.880.596.388	130.765.351.880.923
15.296.223.883.383	291.356.645.397.772	138.394.406.563.942	437.034.968.096.658
125.851.006.445.279	303.449.959.619.701	195.400.246.849.249	5.381.786.459.831

MAPK8IP1-mRNA	WNT2-mRNA	SFN-mRNA	IRS1-mRNA
664.369.223.920.288	505.872.973.848.172	519.623.326.223.166	58.242.644.848.447
246.913.262.129.981	446.913.262.129.981	202.167.364.432.859	702.576.639.896.247
538.288.375.193.019	457.552.882.987.259	47.454.538.313.149	503.496.044.850.989
455.352.786.605.533	210.874.302.338.244	547.797.683.304.815	612.864.258.082.014
462.990.035.150.441	325.138.872.825.068	512.585.784.616.682	455.395.149.827.111
411.271.537.481.654	296.832.546.548.137	324.395.990.809.479	478.648.714.249.555
481.429.513.362.933	385.493.711.812.667	349.236.703.874.197	493.293.963.012.795
540.834.920.963.804	514.531.480.380.425	552.382.642.705.798	528.281.832.755.419
562.945.901.648.462	135.644.052.207.821	518.086.895.749.475	553.966.234.613.398
429.387.092.473.718	124.947.680.537.873	50.043.643.075.422	524.947.680.537.873
518.664.240.649.419	618.664.240.649.419	618.664.240.649.419	444.967.681.232.799
368.184.174.066.471	-127.235.456.972.216	20.495.735.251.652	569.342.971.493.993

MAPK8IP1-mRNA	WNT2-mRNA	SFN-mRNA	IRS1-mRNA
999.886.364.580.366	333.295.454.860.122	366.625.000.346.134	566.602.273.262.207
553.710.784.543.025	22.148.431.381.721	406.054.575.331.551	130.306.604.629.125
417.262.612.149.523	238.435.778.371.156	26.824.025.066.755	32.784.919.526.034
234.827.240.022.999	431.315.338.817.754	445.692.516.778.346	699.689.327.415.468
247.593.296.841.287	952.281.910.928.028	349.170.034.006.944	23.489.620.469.558
173.001.827.753.944	782.627.316.029.748	947.390.961.509.695	275.979.106.178.911
281.350.209.710.882	144.694.393.565.596	112.540.083.884.353	305.465.941.971.814
424.693.231.108.488	35.391.102.592.374	460.084.333.700.862	389.302.128.516.114
495.035.134.257.647	256.052.655.650.507	362.741.262.171.551	465.162.324.431.754
196.148.027.821.385	237.755.185.238.043	320.969.500.071.357	380.408.296.380.868
364.195.806.747.215	728.391.613.494.429	728.391.613.494.429	218.517.484.048.329
128.334.907.888.278	0.413983573833154	413.983.573.833.154	517.479.467.291.442

PAX8-mRNA	EFNA2-mRNA	FGF22-mRNA	CALML5-mRNA
554.415.656.565.196	473.680.164.359.436	573.680.164.359.436	454.415.656.565.196
256.224.202.569.129	114.720.452.641.244	17.321.670.271.336	136.959.694.774.889
416.049.133.059.375	357.552.882.987.259	457.552.882.987.259	389.745.692.475.995
352.378.052.266.128	310.874.302.338.244	496.118.583.496.858	174.617.294.399.773
415.185.305.469.976	351.442.313.408.447	420.247.912.776.973	373.681.555.542.092
26.272.885.476.463	272.039.795.203.778	317.982.957.067.508	26.272.885.476.463
443.989.961.884.783	349.236.703.874.197	529.972.196.079.957	314.444.373.532.166
614.531.480.380.425	482.338.670.891.689	573.027.730.452.541	340.834.920.963.804
135.644.052.207.821	0.771478021357053	301.940.553.480.064	135.644.052.207.821
283.443.930.609.989	183.443.930.609.989	205.683.172.743.634	183.443.930.609.989
518.664.240.649.419	518.664.240.649.419	567.206.923.366.443	386.471.431.160.683
0.312607930998996	-0.27235456972216	189.757.043.172.015	0.72764543027784

PAX8-mRNA	EFNA2-mRNA	FGF22-mRNA	CALML5-mRNA
466.613.636.804.171	266.636.363.888.098	533.272.727.776.195	233.306.818.402.085
590.624.836.845.893	22.148.431.381.721	332.226.470.725.815	258.398.366.120.078
178.826.833.778.367	119.217.889.185.578	238.435.778.371.156	149.022.361.481.972
115.017.423.684.734	862.630.677.635.508	311.505.522.479.489	335.467.485.747.142
177.759.290.039.899	114.273.829.311.363	184.107.836.112.752	133.319.467.529.924
617.863.670.549.801	659.054.581.919.788	906.200.050.139.708	617.863.670.549.801
217.041.590.348.394	112.540.083.884.353	393.890.293.595.235	8.842.435.162.342
70.782.205.184.748	283.128.820.738.992	53.086.653.888.561	106.173.307.777.122
256.052.655.650.507	170.701.770.433.671	810.833.409.559.939	256.052.655.650.507
713.265.555.714.128	356.632.777.857.064	416.071.574.166.575	356.632.777.857.064
364.195.806.747.215	364.195.806.747.215	509.874.129.446.101	145.678.322.698.886
124.195.072.149.946	0.827967147666308	372.585.216.449.838	165.593.429.533.262

MCM7-mRNA	GRIN2A-mRNA	BMP5-mRNA	CDKN1B-mRNA
892.662.620.247.438	554.415.656.565.196	664.369.223.920.288	554.415.656.565.196
831.378.608.478.039	264.970.486.694.163	264.970.486.694.163	436.959.694.774.889
891.537.883.275.721	566.299.167.112.293	757.552.882.987.259	589.745.692.475.995
853.314.262.586.497	326.074.611.682.749	426.074.611.682.749	508.856.514.144.481
896.837.962.265.561	359.242.564.608.574	634.449.813.264.216	580.392.975.127.945
879.721.354.908.861	514.666.270.673.988	681.315.509.295.763	567.459.426.242.466
890.418.063.714.652	454.299.311.181.193	563.920.842.707.124	563.920.842.707.124
848.159.819.166.868	514.531.480.380.425	528.281.832.755.419	540.834.920.963.804
810.909.992.334.956	426.333.111.768.673	617.235.745.763.924	574.875.794.485.697
811.366.295.003.301	663.318.109.785.278	68.117.192.295.998	555.325.755.355.583
703.463.931.304.914	567.206.923.366.443	695.217.715.285.717	444.967.681.232.799
801.535.780.982.729	4.312.607.930.999	673.326.997.947.172	513.703.636.641.554

MCM7-mRNA	GRIN2A-mRNA	BMP5-mRNA	CDKN1B-mRNA
486.611.364.095.778	466.613.636.804.171	999.886.364.580.366	466.613.636.804.171
318.199.130.850.725	627.538.889.148.761	627.538.889.148.761	206.718.692.896.062
482.832.451.201.591	506.676.029.038.706	190.748.622.696.925	59.608.944.592.789
370.451.952.117.916	95.847.853.070.612	191.695.706.141.224	340.259.878.400.673
500.900.285.148.143	120.622.375.384.217	812.613.897.325.251	55.867.205.441.111
444.861.842.795.857	354.241.837.781.886	112.451.188.040.064	510.767.300.987.835
479.099.214.250.531	233.118.745.189.016	498.391.800.059.276	498.391.800.059.276
357.450.136.182.977	35.391.102.592.374	389.302.128.516.114	424.693.231.108.488
276.110.113.676.463	19.203.949.173.788	721.214.980.082.261	537.710.576.866.065
276.984.790.802.319	992.627.898.368.827	112.339.325.024.975	469.566.490.845.134
131.110.490.428.997	509.874.129.446.101	123.826.574.294.053	218.517.484.048.329
258.739.733.645.721	198.712.115.439.914	106.393.778.475.121	351.886.037.758.181

CAMK2B-mRNA	KIT-mRNA	IL7R-mRNA	LAMA1-mRNA
473.680.164.359.436	698.472.915.703.794	694.625.500.922.331	505.872.973.848.172
27.321.670.271.336	544.488.507.505.313	782.433.687.106.147	214.720.452.641.244
696.784.625.265.135	589.745.692.475.995	516.049.133.059.375	416.049.133.059.375
333.113.544.471.888	663.230.497.943.945	782.146.107.130.196	226.074.611.682.749
420.247.912.776.973	661.128.467.333.706	573.681.555.542.092	366.642.622.752.952
452.775.287.409.538	672.039.795.203.778	6.406.898.479.221	311.271.537.481.654
449.236.703.874.196	764.501.404.875.244	600.694.021.157.172	300.694.021.157.172
49.933.117.103.592	663.074.163.097.449	552.382.642.705.798	382.338.670.891.689
390.076.103.830.202	63.489.068.493.928	665.412.107.071.889	177.147.802.135.705
183.443.930.609.989	611.984.152.496.214	575.727.144.557.743	305.683.172.743.634
603.463.931.304.914	544.967.681.232.799	703.463.931.304.914	544.967.681.232.799
281.510.827.152.818	558.562.642.540.541	546.911.241.667.899	-127.235.456.972.216

CAMK2B-mRNA	KIT-mRNA	IL7R-mRNA	LAMA1-mRNA
266.636.363.888.098	126.652.272.846.846	123.319.318.298.245	333.295.454.860.122
664.452.941.451.629	435.585.817.173.846	226.652.281.139.611	44.296.862.763.442
125.178.783.644.857	59.608.944.592.789	357.653.667.556.734	178.826.833.778.367
100.640.245.724.143	992.025.279.280.834	226.200.933.246.644	47.923.926.535.306
184.107.836.112.752	977.676.095.219.442	533.277.870.119.696	12.697.092.145.707
230.669.103.671.926	105.448.733.107.166	848.532.774.221.727	865.009.138.769.722
225.080.167.768.705	200.160.577.765.742	643.086.193.624.873	803.857.742.031.091
318.519.923.331.366	990.950.872.586.472	460.084.333.700.862	141.564.410.369.496
149.364.049.129.462	81.510.095.382.078	100.714.044.555.866	341.403.540.867.343
356.632.777.857.064	695.433.916.821.275	540.893.046.416.547	832.143.148.333.149
655.552.452.144.987	437.034.968.096.658	131.110.490.428.997	437.034.968.096.658
703.772.075.516.361	480.220.945.646.458	442.962.424.001.475	0.413983573833154

SPOP-mRNA	TIAM1-mRNA	SFRP1-mRNA	RFC3-mRNA
554.415.656.565.196	698.472.915.703.794	638.065.783.336.908	694.625.500.922.331
456.224.202.569.129	718.795.086.875.575	583.836.643.096.553	514.720.452.641.244
503.496.044.850.989	686.093.104.873.484	457.552.882.987.259	60.990.907.859.296
429.637.002.655.821	719.148.345.439.037	607.836.937.433.892	586.955.535.950.301
477.076.288.734.425	671.082.034.688.797	520.247.912.776.973	627.523.547.020.504
457.837.894.716.535	631.285.498.930.586	598.718.449.273.268	621.225.104.836.746
472.940.623.604.281	666.229.204.018.428	600.694.021.157.172	616.074.554.765.076
482.338.670.891.689	682.338.670.891.689	563.074.163.097.449	646.724.289.869.161
385.894.086.260.739	745.447.260.503.874	794.140.302.279.937	576.016.270.812.922
449.740.431.882.232	632.629.240.242.956	854.868.482.376.601	622.675.672.887.865
632.414.593.024.413	711.264.182.505.042	518.664.240.649.419	677.160.490.721.535
465.838.276.784.073	849.913.489.977.844	754.462.905.353.322	508.519.743.489.592

SPOP-mRNA	TIAM1-mRNA	SFRP1-mRNA	RFC3-mRNA
466.613.636.804.171	126.652.272.846.846	833.238.637.150.305	123.319.318.298.245
236.249.934.738.357	14.581.050.659.633	572.167.810.694.459	354.374.902.107.536
32.784.919.526.034	116.237.441.955.939	238.435.778.371.156	685.502.862.817.074
196.488.098.794.755	146.167.975.932.683	675.727.364.147.815	584.671.903.730.733
272.987.481.132.701	104.751.010.202.083	368.215.672.225.504	77.452.262.088.813
238.907.285.945.923	794.984.589.440.744	634.340.035.097.796	741.436.404.659.761
26.527.305.487.026	101.286.075.495.917	643.086.193.624.873	715.433.390.407.671
283.128.820.738.992	113.251.528.295.597	495.475.436.293.236	88.477.756.480.935
145.096.504.868.621	175.396.069.120.597	245.810.549.424.487	541.978.121.126.906
22.586.742.597.614	802.423.750.178.394	374.464.416.749.917	748.928.833.499.834
801.230.774.843.872	138.394.406.563.942	364.195.806.747.215	109.258.742.024.164
252.529.980.038.224	361.821.643.530.177	186.706.591.798.752	339.466.530.543.186

LEFTY1-mRNA	TNFRSF10A-mRNA	CCNE2-mRNA	CSF3R-mRNA
626.036.359.965.137	473.680.164.359.436	543.724.136.173.545	626.036.359.965.137
226.268.174.383.238	246.913.262.129.981	320.609.821.546.601	377.169.539.132.024
457.552.882.987.259	503.496.044.850.989	548.241.942.548.111	557.552.882.987.259
210.874.302.338.244	3.893.014.332.327	352.378.052.266.128	534.820.895.807.782
351.442.313.408.447	429.869.444.302.903	451.442.313.408.447	604.493.785.078.325
242.083.767.017.887	450.175.766.556.244	357.837.894.716.535	651.481.381.838.789
368.501.211.668.436	463.920.842.707.124	432.886.830.645.909	568.501.211.668.436
540.834.920.963.804	49.933.117.103.592	440.834.920.963.804	414.531.480.380.425
-0.228521978642947	347.191.773.949.814	223.090.963.999.435	531.063.683.246.508
0.249476805378731	405.683.172.743.634	205.683.172.743.634	486.418.664.949.394
518.664.240.649.419	444.967.681.232.799	677.160.490.721.535	486.471.431.160.683
0.312607930998996	20.495.735.251.652	172.764.543.027.784	461.028.847.963.968

LEFTY1-mRNA	TNFRSF10A-mRNA	CCNE2-mRNA	CSF3R-mRNA
766.579.546.178.281	266.636.363.888.098	433.284.091.318.159	766.579.546.178.281
479.882.679.937.288	553.710.784.543.025	922.851.307.571.707	136.581.993.520.613
238.435.778.371.156	32.784.919.526.034	447.067.084.445.918	476.871.556.742.312
431.315.338.817.754	148.564.172.259.449	115.017.423.684.734	407.353.375.550.101
114.273.829.311.363	196.804.928.258.459	228.547.658.622.727	660.248.791.576.766
535.481.847.809.827	226.550.012.534.927	119.453.642.972.962	914.438.232.413.706
128.617.238.724.975	249.195.900.029.638	200.964.435.507.773	514.468.954.899.898
424.693.231.108.488	318.519.923.331.366	212.346.615.554.244	17.695.551.296.187
0.853508852168356	110.956.150.781.886	469.429.868.692.596	396.881.616.258.286
118.877.592.619.021	16.642.862.966.663	416.071.574.166.575	291.250.101.916.602
364.195.806.747.215	218.517.484.048.329	109.258.742.024.164	291.356.645.397.772
124.195.072.149.946	413.983.573.833.154	331.186.859.066.523	244.250.308.561.561

IGF1-mRNA	ATRX-mRNA	PLAU-mRNA	B2M-mRNA
766.753.898.115.724	930.665.725.192.531	678.119.576.295.281	122.681.831.041.107
579.106.071.618.717	867.598.419.174.048	724.523.660.937.297	123.883.932.404.915
750.626.616.743.548	100.101.570.575.093	666.299.167.112.293	112.863.352.635.719
626.973.490.005.474	893.599.750.287.774	768.028.500.834.127	138.432.174.433.207
677.912.636.027.888	927.966.318.224.585	688.365.694.375.019	130.034.905.662.143
675.931.694.133.008	914.771.079.617.275	663.328.728.826.774	134.177.456.565.372
773.486.066.613.492	936.976.073.710.599	6.239.600.968.362	127.723.091.976.035
563.074.163.097.449	874.224.994.619.148	63.469.486.649.739	126.451.606.908.875
603.826.456.205.195	883.487.310.264.556	3.980.931.386.986	13.529.336.767.948
599.094.379.177.988	909.809.974.580.807	460.702.880.999.682	135.918.293.154.665
567.206.923.366.443	853.713.965.357.833	544.967.681.232.799	113.485.300.888.711
725.902.689.079.415	856.842.335.387.289	495.646.412.077.372	128.624.727.999.109

IGF1-mRNA	ATRX-mRNA	PLAU-mRNA	B2M-mRNA
203.310.227.464.674	633.261.364.234.231	10.998.750.010.384	493.277.273.192.981
553.710.784.543.024	409.007.699.515.781	151.716.754.964.789	536.139.695.646.859
181.807.281.008.006	103.123.474.145.525	101.335.205.807.741	249.761.477.843.786
771.575.217.218.427	489.782.529.190.827	20.511.440.557.111	146.968.305.505.823
109.829.847.060.366	62.152.266.053.236	118.082.956.955.075	821.184.434.523.603
108.332.096.903.065	567.198.849.564.717	99.270.096.401.668	109.431.894.236.644
213.022.301.638.239	661.574.921.691.588	755.626.277.509.226	699.597.392.889.659
495.475.436.293.236	428.232.341.367.725	813.995.359.624.602	640.578.956.921.969
657.201.816.169.634	456.627.235.910.071	157.899.137.651.146	118.232.313.746.622
635.995.120.511.764	548.025.701.973.689	243.699.064.868.994	123.466.267.694.116
509.874.129.446.101	371.479.722.882.159	437.034.968.096.658	260.764.197.631.006
153.173.922.318.267	379.622.937.205.002	310.487.680.374.865	744.715.050.968.461

PIK3R5-mRNA	SOS1-mRNA	CREB5-mRNA	FGF21-mRNA
654.415.656.565.196	812.911.906.637.312	669.099.795.398.123	590.672.664.503.667
41.769.518.698.065	744.488.507.505.313	414.720.452.641.244	202.167.364.432.859
489.745.692.475.995	909.122.866.815.663	616.049.133.059.375	548.241.942.548.111
477.170.803.610.486	742.263.379.920.438	565.306.353.960.625	274.617.294.399.773
525.138.872.825.068	80.893.319.701.417	5.227.141.182.004	366.642.622.752.952
516.334.144.788.651	80.104.167.989.704	570.908.263.880.995	296.832.546.548.137
546.637.183.020.902	777.776.925.760.421	563.920.842.707.124	393.293.963.012.795
668.136.770.404.446	749.581.205.088.838	691.084.955.016.723	582.338.670.891.689
467.836.861.696.557	762.945.901.648.463	494.140.302.279.937	135.644.052.207.821
53.155.659.958.365	789.693.523.183.365	50.043.643.075.422	183.443.930.609.989
695.217.715.285.717	725.703.173.438.559	544.967.681.232.799	386.471.431.160.683
563.453.602.588.636	714.127.335.930.201	518.707.704.891.514	172.764.543.027.784

PIK3R5-mRNA	SOS1-mRNA	CREB5-mRNA	FGF21-mRNA
933.227.273.608.341	279.968.182.082.502	103.321.591.006.638	599.931.818.748.219
180.878.856.284.055	174.234.326.869.538	177.187.451.053.768	406.054.575.331.551
298.044.722.963.945	545.421.843.024.019	715.307.335.113.468	447.067.084.445.918
273.166.381.251.244	171.567.656.996.395	503.201.228.620.713	670.934.971.494.284
380.912.764.371.211	272.352.626.525.416	374.564.218.298.358	12.697.092.145.707
358.360.928.918.884	257.855.105.176.117	523.124.574.398.831	782.627.316.029.748
4.421.217.581.171	219.453.163.574.488	498.391.800.059.276	152.732.970.985.907
102.634.197.517.885	180.494.623.221.107	120.329.748.814.072	566.257.641.477.984
256.052.655.650.507	198.014.053.703.059	307.263.186.780.608	256.052.655.650.507
398.239.935.273.721	238.349.573.201.138	320.969.500.071.357	356.632.777.857.064
123.826.574.294.053	15.296.223.883.383	437.034.968.096.658	145.678.322.698.886
496.780.288.599.785	141.168.398.677.106	364.305.544.973.175	331.186.859.066.523

PPP2R1A-mRNA	PIK3R3-mRNA	PBX3-mRNA	GLI3-mRNA
686.608.466.053.932	678.119.576.295.281	694.625.500.922.331	626.036.359.965.137
841.711.040.895.152	578.141.054.615.345	543.260.674.527.469	763.905.762.274.212
70.990.907.859.296	627.596.854.801.368	784.231.537.056.749	596.784.625.265.135
821.494.242.721.436	679.679.901.706.769	614.827.138.756.907	670.700.234.671.705
671.953.756.398.908	688.365.694.375.019	621.486.285.222.556	658.290.287.196.724
722.819.259.223.648	667.459.426.242.466	689.532.363.453.846	612.127.738.831.997
774.029.455.218.555	671.843.511.822.181	66.275.266.220.236	660.387.535.395.896
773.027.730.452.541	677.758.301.930.376	573.027.730.452.541	682.338.670.891.689
809.340.611.624.441	666.022.127.025.531	58.049.010.228.945	606.609.877.024.868
822.389.139.518.426	732.629.240.242.956	621.526.109.004.082	587.883.342.545.834
725.703.173.438.559	656.515.402.974.792	544.967.681.232.799	586.471.431.160.683
834.051.392.756.888	538.585.691.302.963	618.707.704.891.514	642.113.238.777.717

PPP2R1A-mRNA	PIK3R3-mRNA	PBX3-mRNA	GLI3-mRNA
116.653.409.201.043	10.998.750.010.384	123.319.318.298.245	766.579.546.178.281
34.182.412.432.456	550.019.379.312.738	431.894.411.943.559	199.335.882.435.489
137.100.572.563.415	774.916.279.706.257	229.494.436.682.238	625.893.918.224.285
297.128.344.518.897	11.118.350.956.191	709.274.112.722.529	104.474.159.846.967
105.385.864.809.368	118.082.956.955.075	742.779.890.523.862	958.630.457.000.881
149.934.917.386.752	102.153.460.197.567	119.041.733.859.262	696.126.402.152.776
21.382.615.938.027	105.305.364.206.073	988.745.022.698.242	97.266.786.785.762
212.346.615.554.244	109.712.418.036.359	53.086.653.888.561	113.251.528.295.597
273.122.832.693.874	10.114.079.898.195	559.048.298.170.273	67.000.444.895.216
298.977.145.436.838	160.484.750.035.679	742.984.953.868.883	588.444.083.464.155
15.296.223.883.383	946.909.097.542.759	437.034.968.096.658	582.713.290.795.544
32.414.913.831.136	418.123.409.571.485	728.611.089.946.351	856.945.997.834.628

PPARGC1A-mRNA	BAD-mRNA	FGF20-mRNA	PPP3CC-mRNA
590.672.664.503.667	678.119.576.295.281	405.872.973.848.172	619.623.326.223.166
156.224.202.569.129	756.224.202.569.129	202.167.364.432.859	60.858.039.817.483
503.496.044.850.989	740.841.884.403.733	557.552.882.987.259	730.344.928.443.579
508.856.514.144.481	775.259.921.315.716	226.074.611.682.749	665.991.721.064.731
51.773.881.468.069	698.835.432.241.688	373.681.555.542.092	726.336.136.991.675
487.014.507.154.246	748.858.227.681.471	311.271.537.481.654	755.644.830.709.585
597.041.433.554.661	78.649.212.066.993	285.493.711.812.667	697.041.433.554.661
440.834.920.963.804	628.281.832.755.419	382.338.670.891.689	724.965.146.361.899
470.221.535.891.994	713.780.023.560.287	194.140.302.279.937	686.423.516.227.691
63.787.598.223.237	703.737.936.477.016	205.683.172.743.634	661.579.901.962.455
603.463.931.304.914	644.967.681.232.799	544.967.681.232.799	686.471.431.160.683
372.764.543.027.784	690.257.111.277.852	1.312.607.930.999	637.150.162.005.256

PPARGC1A-mRNA	BAD-mRNA	FGF20-mRNA	PPP3CC-mRNA
599.931.818.748.219	10.998.750.010.384	166.647.727.430.061	733.250.000.692.268
295.312.418.422.946	188.999.947.790.686	406.054.575.331.551	679.218.562.372.777
32.784.919.526.034	169.885.492.089.449	476.871.556.742.312	157.963.703.170.891
340.259.878.400.673	215.657.669.408.877	47.923.926.535.306	101.119.484.989.496
361.867.126.152.651	12.697.092.145.707	133.319.467.529.924	153.634.814.963.055
292.455.470.726.906	179.592.373.573.142	865.009.138.769.722	188.242.464.960.839
627.009.038.784.251	233.118.745.189.016	723.471.967.827.982	12.540.180.775.685
212.346.615.554.244	778.604.257.032.228	141.564.410.369.496	152.181.741.147.208
260.320.199.911.349	140.828.960.607.779	38.407.898.347.576	116.503.958.320.981
832.143.148.333.149	131.359.739.844.019	416.071.574.166.575	980.740.139.106.926
655.552.452.144.987	874.069.936.193.316	437.034.968.096.658	116.542.658.159.109
132.474.743.626.609	119.641.252.837.781	248.390.144.299.892	827.967.147.666.307

HOXA9-mRNA	BMPR1B-mRNA	THBS1-mRNA	CACNA1H-mRNA
58.242.644.848.447	632.176.414.431.551	930.665.725.192.531	543.724.136.173.545
288.417.012.057.865	346.913.262.129.981	110.425.273.466.277	364.970.486.694.163
516.049.133.059.375	503.496.044.850.989	936.343.138.926.402	596.784.625.265.135
252.378.052.266.128	646.237.997.799.714	865.134.502.237.995	550.867.363.027.107
373.681.555.542.092	555.395.149.827.111	916.627.211.461.272	582.023.156.360.855
404.232.604.692.514	476.479.207.139.623	116.476.203.547.624	639.987.805.154.323
443.989.961.884.783	485.493.711.812.667	110.965.231.046.964	572.940.623.604.281
628.281.832.755.419	514.531.480.380.425	107.332.797.926.869	563.074.163.097.449
257.883.294.341.466	636.393.505.862.513	929.895.502.741.745	601.940.553.480.064
241.940.180.682.104	565.886.774.151.643	106.185.288.123.801	70.242.638.649.799
544.967.681.232.799	703.463.931.304.914	903.463.931.304.914	667.206.923.366.443
20.495.735.251.652	348.253.293.244.131	108.014.522.774.563	497.557.294.372.143

HOXA9-mRNA	BMPR1B-mRNA	THBS1-mRNA	CACNA1H-mRNA
566.602.273.262.207	799.909.091.664.293	633.261.364.234.231	433.284.091.318.159
738.281.046.057.366	110.742.156.908.605	210.926.894.858.589	125.507.777.829.752
357.653.667.556.734	32.784.919.526.034	658.678.837.750.319	625.893.918.224.285
575.087.118.423.672	881.800.248.249.631	402.081.743.631.217	455.277.302.085.407
133.319.467.529.924	469.792.409.391.161	574.543.419.593.243	565.020.600.483.963
164.763.645.479.947	271.860.015.041.912	320.836.008.660.826	844.413.683.084.728
217.041.590.348.394	289.388.787.131.193	218.970.848.929.269	53.054.610.974.052
778.604.257.032.228	35.391.102.592.374	170.231.203.469.319	495.475.436.293.236
59.745.619.651.785	823.636.042.342.464	629.889.532.900.247	648.666.727.647.951
534.949.166.785.596	50.522.976.863.084	157.215.616.238.656	130.170.963.917.828
437.034.968.096.658	131.110.490.428.997	524.441.961.715.989	10.197.482.588.922
413.983.573.833.154	111.775.564.934.951	178.468.318.679.472	314.627.516.113.197

SOCS2-mRNA	HSPA2-mRNA	ITGA8-mRNA	CACNA1C-mRNA
771.408.156.709.427	543.724.136.173.545	543.724.136.173.545	505.872.973.848.172
819.341.908.139.527	614.720.452.641.244	226.268.174.383.238	614.720.452.641.244
721.938.501.964.731	457.552.882.987.259	57.454.538.313.149	589.745.692.475.995
826.749.294.926.807	761.124.336.391.162	363.925.774.008.121	565.306.353.960.625
775.812.606.166.633	568.434.813.552.678	434.449.813.264.216	653.432.269.152.217
903.214.626.704.274	442.083.767.017.887	372.039.795.203.778	627.498.680.371.542
819.280.675.688.306	617.686.521.301.404	359.190.271.229.288	609.440.305.282.206
855.130.716.348.009	737.797.556.059.453	582.338.670.891.689	668.136.770.404.446
839.718.686.442.152	556.589.388.770.716	552.636.552.352.052	559.165.698.377.224
858.039.368.349.335	580.406.565.705.637	722.675.672.887.865	574.132.990.170.841
79.939.973.285.518	518.664.240.649.419	567.206.923.366.443	644.967.681.232.799
839.120.353.449.511	415.391.018.497.994	477.203.954.963.629	513.703.636.641.554

SOCS2-mRNA	HSPA2-mRNA	ITGA8-mRNA	CACNA1C-mRNA
209.976.136.561.877	433.284.091.318.159	433.284.091.318.159	333.295.454.860.122
292.728.434.761.746	708.749.804.215.071	479.882.679.937.288	708.749.804.215.071
149.022.361.481.972	238.435.778.371.156	536.480.501.335.101	59.608.944.592.789
308.150.847.622.018	195.529.620.264.048	124.602.208.991.796	503.201.228.620.713
216.485.421.084.305	514.232.231.901.135	203.153.474.331.313	926.887.726.636.614
523.536.483.512.532	214.192.739.123.931	131.810.916.383.958	774.389.133.755.751
292.604.218.099.317	723.471.967.827.982	120.578.661.304.664	683.279.080.726.427
375.145.687.479.164	166.338.182.184.158	566.257.641.477.984	102.634.197.517.885
337.135.996.606.501	473.697.412.953.438	460.894.780.170.912	482.232.501.475.121
382.785.848.233.248	5.587.246.853.094	149.785.766.699.967	534.949.166.785.596
25.493.706.472.305	364.195.806.747.215	509.874.129.446.101	874.069.936.193.316
335.740.678.378.688	178.012.936.748.256	273.229.158.729.881	351.886.037.758.181

**APC-mRNA**	**GATA1-mRNA**	**ERCC2-mRNA**	**NF2-mRNA**
58.242.644.848.447	490.672.664.503.667	490.672.664.503.667	790.672.664.503.667
566.027.410.865.181	114.720.452.641.244	469.152.504.263.625	68.705.810.558.307
643.350.982.500.016	489.745.692.475.995	527.596.854.801.368	826.202.935.705.581
54.306.711.182.698	210.874.302.338.244	502.628.086.319.046	841.859.828.596.922
625.138.872.825.068	292.946.063.336.331	538.889.225.200.061	794.441.097.482.928
601.501.870.092.941	311.271.537.481.654	554.057.691.445.297	806.691.168.520.342
563.920.842.707.124	338.545.183.482.545	5.239.600.968.362	832.163.673.722.801
514.531.480.380.425	440.834.920.963.804	440.834.920.963.804	752.382.642.705.798
541.533.421.113.178	135.644.052.207.821	462.945.901.648.462	763.566.416.601.133
61.074.578.005.063	0.249476805378731	467.574.156.008.083	790.052.849.655.766
586.471.431.160.683	486.471.431.160.683	386.471.431.160.683	786.471.431.160.683
589.757.043.172.015	-127.235.456.972.216	372.764.543.027.784	733.867.022.758.519

**APC-mRNA**	**GATA1-mRNA**	**ERCC2-mRNA**	**NF2-mRNA**
566.602.273.262.207	29.996.590.937.411	29.996.590.937.411	239.972.727.499.288
505.722.516.549.296	22.148.431.381.721	258.398.366.120.078	117.017.545.800.093
86.432.969.659.544	298.044.722.963.945	387.458.139.853.129	306.986.064.652.863
431.315.338.817.754	431.315.338.817.754	325.882.700.440.081	342.176.835.462.084
761.825.528.742.423	761.825.528.742.422	419.004.040.808.332	246.323.587.626.716
646.697.308.508.792	865.009.138.769.722	46.545.729.848.085	268.152.833.018.613
498.391.800.059.276	104.501.506.464.042	377.813.138.754.613	319.935.381.328.375
35.391.102.592.374	212.346.615.554.244	212.346.615.554.244	184.033.733.480.345
426.754.426.084.178	256.052.655.650.507	247.517.567.128.823	198.867.562.555.227
689.490.037.190.323	118.877.592.619.021	255.586.824.130.896	238.943.961.164.233
582.713.290.795.544	291.356.645.397.772	145.678.322.698.886	233.085.316.318.218
596.136.346.319.741	0.413983573833154	132.474.743.626.609	161.867.577.368.763

PGF-mRNA	PTPN11-mRNA	IL20RB-mRNA	RAC1-mRNA
626.036.359.965.137	722.865.473.992.403	490.672.664.503.667	649.168.914.575.783
524.874.255.287.451	776.191.437.052.765	314.720.452.641.244	823.643.429.383.697
538.288.375.193.019	713.011.768.155.023	503.496.044.850.989	616.049.133.059.375
704.734.247.871.829	765.306.353.960.625	339.824.964.057.742	784.570.861.754.864
586.806.008.869.917	721.486.285.222.556	425.138.872.825.068	653.432.269.152.217
615.502.617.967.451	706.912.610.626.886	336.425.414.181.251	678.109.388.372.534
554.299.311.181.193	751.156.060.397.528	385.493.711.812.667	654.299.311.181.193
686.778.082.827.534	852.382.642.705.798	482.338.670.891.689	731.523.980.524.656
571.399.252.669.629	715.949.530.670.219	209.340.611.624.442	772.567.433.174.393
572.521.023.634.513	764.608.158.656.059	315.636.740.098.725	83.155.659.958.365
677.160.490.721.535	677.160.490.721.535	644.967.681.232.799	603.463.931.304.914
66.163.886.791.761	752.206.129.662.795	153.500.035.233.544	862.700.235.320.095

PGF-mRNA	PTPN11-mRNA	IL20RB-mRNA	RAC1-mRNA
766.579.546.178.281	149.982.954.687.055	29.996.590.937.411	899.897.728.122.329
380.214.738.719.544	217.054.627.540.866	885.937.255.268.839	301.587.807.314.434
417.262.612.149.523	140.081.019.793.054	32.784.919.526.034	715.307.335.113.468
132.270.037.237.445	201.280.491.448.285	105.432.638.377.673	230.034.847.369.469
584.066.238.702.524	148.555.978.104.772	190.456.382.185.606	926.887.726.636.614
71.260.276.670.077	134.282.371.066.157	102.977.278.424.967	109.979.733.357.865
466.237.490.378.033	182.475.707.441.058	144.694.393.565.596	932.474.980.756.065
116.790.638.554.834	36.806.746.696.069	283.128.820.738.992	159.259.961.665.683
524.907.944.083.539	1.429.627.327.382	426.754.426.084.178	211.670.195.337.752
529.005.287.154.645	200.308.743.563.051	89.158.194.464.266	318.591.948.218.977
109.258.742.024.164	109.258.742.024.164	874.069.936.193.316	655.552.452.144.987
981.141.069.984.574	18.380.870.678.192	289.788.501.683.208	395.354.313.010.662

PLD1-mRNA	FZD3-mRNA	FGF13-mRNA	AKT3-mRNA
673.680.164.359.436	649.168.914.575.783	373.680.164.359.436	780.289.083.405.213
569.152.504.263.625	67.321.670.271.336	460.663.614.504.974	74.540.257.289.096
60.990.907.859.296	678.498.219.550.154	527.596.854.801.368	793.308.083.449.067
640.642.357.202.312	738.590.124.814.978	318.674.553.538.371	821.261.362.115.439
682.831.390.990.641	698.835.432.241.688	366.642.622.752.952	813.891.399.899.226
763.926.118.931.238	737.503.398.056.575	272.039.795.203.778	869.194.150.598.855
72.007.119.549.684	746.637.183.020.902	314.444.373.532.166	88.423.590.520.472
528.281.832.755.419	621.570.413.169.565	382.338.670.891.689	721.570.413.169.565
629.503.997.741.407	683.217.395.304.461	285.894.086.260.739	84.874.400.116.122
670.068.791.721.106	641.940.180.682.104	424.947.680.537.873	878.280.653.768.456
544.967.681.232.799	718.664.240.649.419	544.967.681.232.799	718.664.240.649.419
587.739.254.978.252	683.093.323.868.986	337.150.162.005.256	879.104.051.156.635

PLD1-mRNA	FZD3-mRNA	FGF13-mRNA	AKT3-mRNA
106.654.545.555.239	899.897.728.122.329	133.318.181.944.049	223.307.954.756.282
516.796.732.240.156	106.312.470.632.261	243.632.745.198.931	175.341.748.438.624
685.502.862.817.074	11.027.654.749.666	387.458.139.853.129	244.396.672.830.435
848.253.499.674.916	167.254.503.608.218	910.554.604.170.814	296.649.105.253.544
113.638.974.704.078	12.697.092.145.707	12.697.092.145.707	281.875.445.634.696
199.364.011.030.736	165.999.372.821.047	659.054.581.919.788	413.556.750.154.667
14.710.596.679.169	17.684.870.324.684	8.842.435.162.342	459.002.770.699.753
389.302.128.516.114	743.213.154.439.854	141.564.410.369.496	148.642.630.887.971
785.228.143.994.888	113.943.431.764.476	725.482.524.343.103	358.900.472.336.794
104.017.893.541.644	855.918.666.856.953	190.204.148.190.434	440.441.480.653.474
437.034.968.096.658	145.678.322.698.886	437.034.968.096.658	145.678.322.698.886
587.856.674.843.078	113.845.482.804.117	103.495.893.458.288	442.962.424.001.475

C19orf40-mRNA	NKD1-mRNA	CREB3L4-mRNA	BID-mRNA
598.472.915.703.794	58.242.644.848.447	598.472.915.703.794	632.176.414.431.551
326.268.174.383.238	786.830.371.511.963	384.764.424.455.354	523.466.736.766.278
589.745.692.475.995	457.552.882.987.259	538.288.375.193.019	527.596.854.801.368
406.810.103.888.509	850.487.206.011.121	358.267.421.171.485	4.893.014.332.327
486.806.008.869.917	720.247.912.776.973	473.681.555.542.092	520.247.912.776.973
442.083.767.017.887	519.613.138.300.418	411.271.537.481.654	492.985.131.766.673
443.989.961.884.783	818.485.800.376.757	468.501.211.668.436	468.501.211.668.436
552.382.642.705.798	540.834.920.963.804	582.338.670.891.689	673.027.730.452.541
381.587.214.071.551	659.165.698.377.224	426.333.111.768.673	465.412.107.071.889
370.890.842.401.603	703.737.936.477.016	415.636.740.098.725	489.333.299.515.346
656.515.402.974.792	618.664.240.649.419	632.414.593.024.413	567.206.923.366.443
3.312.607.930.999	708.079.225.577.592	445.556.588.484.104	534.235.527.439.305

C19orf40-mRNA	NKD1-mRNA	CREB3L4-mRNA	BID-mRNA
633.261.364.234.232	566.602.273.262.207	633.261.364.234.232	799.909.091.664.293
959.765.359.874.576	233.665.951.077.156	143.964.803.981.186	376.523.333.489.257
59.608.944.592.789	238.435.778.371.156	417.262.612.149.523	387.458.139.853.129
167.733.742.873.571	363.263.363.137.619	119.809.816.338.265	297.128.344.518.897
292.033.119.351.262	147.286.268.890.202	266.638.935.059.848	368.215.672.225.504
214.192.739.123.931	366.599.111.192.882	173.001.827.753.944	304.812.744.137.902
217.041.590.348.394	290.996.502.615.255	257.234.477.449.949	257.234.477.449.949
460.084.333.700.862	424.693.231.108.488	566.257.641.477.984	106.173.307.777.122
140.828.960.607.779	964.465.002.950.243	19.203.949.173.788	251.785.111.389.665
130.765.351.880.923	131.359.739.844.019	178.316.388.928.532	297.193.981.547.553
946.909.097.542.759	728.391.613.494.429	801.230.774.843.872	509.874.129.446.101
993.560.577.199.569	135.372.628.643.441	219.411.294.131.571	405.703.902.356.491

TNFRSF10D-mRNA	CDKN2B-mRNA	FGF4-mRNA	RRAS2-mRNA
619.623.326.223.166	612.911.906.637.312	505.872.973.848.172	764.369.223.920.288
524.874.255.287.451	460.663.614.504.974	202.167.364.432.859	497.163.296.182.899
57.454.538.313.149	503.496.044.850.989	516.049.133.059.375	589.745.692.475.995
618.674.553.538.371	486.955.535.950.301	210.874.302.338.244	563.925.774.008.121
545.302.258.942.033	504.493.785.078.325	334.449.813.264.216	532.177.805.614.207
511.271.537.481.654	556.588.800.298.216	311.271.537.481.654	537.860.943.478.958
628.492.495.887.149	514.444.373.532.166	359.190.271.229.288	581.429.513.362.933
765.627.672.308.163	49.933.117.103.592	482.338.670.891.689	746.724.289.869.161
483.756.721.181.483	277.147.802.135.705	0.356440522078209	62.871.778.596.411
655.325.755.355.583	341.940.180.682.104	224.947.680.537.873	642.938.589.539.367
518.664.240.649.419	567.206.923.366.443	586.471.431.160.683	767.206.923.366.443
545.556.588.484.104	468.184.174.066.471	0.312607930998996	613.703.636.641.554

TNFRSF10D-mRNA	CDKN2B-mRNA	FGF4-mRNA	RRAS2-mRNA
733.250.000.692.268	699.920.455.206.256	333.295.454.860.122	199.977.272.916.073
380.214.738.719.544	243.632.745.198.931	406.054.575.331.551	313.769.444.574.381
536.480.501.335.101	32.784.919.526.034	357.653.667.556.734	59.608.944.592.789
728.443.683.336.651	292.335.951.865.367	431.315.338.817.754	498.408.835.967.182
438.049.679.026.893	330.124.395.788.383	101.576.737.165.656	399.958.402.589.772
346.003.655.507.889	473.695.480.754.847	865.009.138.769.722	416.028.204.836.866
779.742.009.770.158	35.369.740.649.368	120.578.661.304.664	562.700.419.421.764
201.729.284.776.532	318.519.923.331.366	283.128.820.738.992	17.695.551.296.187
285.925.465.476.399	682.807.081.734.685	128.026.327.825.253	780.960.599.734.046
939.132.981.690.268	106.989.833.357.119	475.510.370.476.085	861.862.546.487.904
364.195.806.747.215	509.874.129.446.101	582.713.290.795.544	20.394.965.177.844
438.822.588.263.143	256.669.815.776.555	124.195.072.149.946	703.772.075.516.361

EGFR-mRNA	STAT4-mRNA	DAXX-mRNA	PROM1-mRNA
716.306.639.829.646	605.872.973.848.172	719.623.326.223.166	598.472.915.703.794
549.297.936.325.418	364.970.486.694.163	669.666.834.591.222	202.167.364.432.859
713.011.768.155.023	503.496.044.850.989	730.344.928.443.579	457.552.882.987.259
753.873.086.412.725	518.674.553.538.371	697.224.102.347.757	731.385.745.328.705
761.596.116.054.653	655.395.149.827.111	685.229.277.284.085	681.210.368.272.515
753.096.958.677.893	713.402.588.106.195	670.908.263.880.995	411.271.537.481.654
753.676.115.810.042	572.940.623.604.281	678.304.419.964.489	37.724.749.579.347
691.084.955.016.723	614.531.480.380.425	718.093.871.353.497	540.834.920.963.804
850.449.334.304.302	607.525.876.953.416	673.726.230.601.914	831.644.245.414.629
886.786.230.763.734	503.083.651.890.339	696.372.232.304.485	697.739.725.994.193
750.857.050.138.155	603.463.931.304.914	711.264.182.505.042	790.910.843.096.528
725.902.689.079.415	481.510.827.152.818	671.633.011.705.001	2.312.607.930.999

EGFR-mRNA	STAT4-mRNA	DAXX-mRNA	PROM1-mRNA
143.317.045.589.852	666.590.909.720.244	146.650.000.138.454	633.261.364.234.232
450.351.438.094.993	125.507.777.829.752	10.372.848.697.106	406.054.575.331.551
140.081.019.793.054	32.784.919.526.034	157.963.703.170.891	238.435.778.371.156
185.944.834.956.987	364.221.841.668.326	125.560.687.522.502	159.107.436.097.216
196.170.073.651.174	939.584.818.782.321	115.543.538.525.934	112.369.265.489.507
18.494.719.205.124	140.461.007.771.655	104.624.914.879.766	173.001.827.753.944
185.691.138.409.182	53.054.610.974.052	110.128.510.658.259	136.655.816.145.285
120.329.748.814.072	70.782.205.184.748	145.103.520.628.733	424.693.231.108.488
363.168.016.597.636	674.271.993.213.002	106.688.606.521.045	318.785.556.284.881
467.188.938.992.754	326.913.379.702.309	124.821.472.249.972	126.010.248.176.163
182.097.903.373.607	655.552.452.144.987	138.394.406.563.942	240.369.232.453.162
153.173.922.318.267	281.508.830.206.545	105.151.827.753.621	496.780.288.599.784

FGFR2-mRNA	CDK6-mRNA	MPO-mRNA	CACNG1-mRNA
804.058.239.177.146	626.036.359.965.137	598.472.915.703.794	532.176.414.431.551
732.379.325.813.577	703.391.724.008.333	202.167.364.432.859	0.884170120578651
989.294.144.363.746	589.745.692.475.995	457.552.882.987.259	548.241.942.548.111
636.508.277.664.222	682.756.127.083.838	226.074.611.682.749	239.824.964.057.742
801.692.347.461.365	690.674.055.686.323	392.946.063.336.331	334.449.813.264.216
806.247.262.003.692	639.987.805.154.323	317.982.957.067.508	347.528.545.420.125
739.237.124.876.524	738.545.183.482.545	338.545.183.482.545	385.493.711.812.667
657.827.421.108.036	652.382.642.705.798	282.338.670.891.689	440.834.920.963.804
729.895.502.741.745	601.940.553.480.064	-0.228521978642947	135.644.052.207.821
776.517.664.366.277	671.708.235.546.173	205.683.172.743.634	0.834439306099887
750.857.050.138.155	644.967.681.232.799	544.967.681.232.799	518.664.240.649.419
720.337.886.124.424	593.709.879.590.679	0.72764543027784	172.764.543.027.784

FGFR2-mRNA	CDK6-mRNA	MPO-mRNA	CACNG1-mRNA
263.303.409.339.497	766.579.546.178.281	633.261.364.234.232	399.954.545.832.146
160.206.986.994.448	131.044.885.675.183	406.054.575.331.551	184.570.261.514.342
950.762.666.254.984	59.608.944.592.789	238.435.778.371.156	447.067.084.445.918
824.291.536.407.263	113.579.705.888.675	47.923.926.535.306	527.163.191.888.366
259.020.679.772.424	119.987.520.776.932	152.365.105.748.484	101.576.737.165.656
267.329.014.791.214	844.413.683.084.728	906.200.050.139.708	111.215.460.698.964
168.006.268.084.498	167.202.410.342.467	104.501.506.464.042	144.694.393.565.596
955.559.769.994.098	920.168.667.401.724	70.782.205.184.748	212.346.615.554.244
157.472.383.225.062	648.666.727.647.951	0.853508852168356	256.052.655.650.507
217.545.994.492.809	105.206.669.467.834	416.071.574.166.575	178.316.388.928.532
182.097.903.373.607	874.069.936.193.316	437.034.968.096.658	364.195.806.747.215
147.378.152.284.603	612.695.689.273.068	165.593.429.533.262	331.186.859.066.523

MECOM-mRNA	GPC4-mRNA	HDAC10-mRNA	GRIA3-mRNA
632.176.414.431.551	654.415.656.565.196	84.782.686.299.955	690.672.664.503.667
442.022.302.081.886	737.602.321.690.833	611.683.087.736.893	405.409.512.202.096
596.784.625.265.135	700.179.358.457.469	8.445.893.549.456	81.902.386.739.878
610.874.302.338.244	706.293.933.376.931	774.294.904.312.344	493.881.802.194.012
573.681.555.542.092	659.242.564.608.574	818.998.818.358.653	609.938.563.480.562
567.459.426.242.466	736.064.288.826.013	859.230.318.969.697	573.162.520.746.104
5.239.600.968.362	663.920.842.707.124	845.979.917.628.554	611.127.687.138.646
463.074.163.097.449	73.469.486.649.739	657.827.421.108.036	482.338.670.891.689
69.662.348.757.793	696.130.258.023.707	731.063.683.246.508	423.090.963.999.435
685.680.711.912.834	723.247.038.007.304	719.199.131.071.797	524.947.680.537.873
586.471.431.160.683	632.414.593.024.413	738.827.626.766.384	586.471.431.160.683
485.692.844.722.281	77.219.988.671.367	673.326.997.947.172	468.184.174.066.471

MECOM-mRNA	GPC4-mRNA	HDAC10-mRNA	GRIA3-mRNA
799.909.091.664.293	933.227.273.608.341	35.662.613.670.033	119.986.363.749.644
214.101.503.356.636	166.113.235.362.907	693.984.183.293.924	166.113.235.362.907
625.893.918.224.285	128.159.230.874.496	348.712.325.867.816	292.083.828.504.666
690.104.542.108.406	133.707.755.033.504	214.219.951.612.818	306.713.129.825.958
533.277.870.119.696	964.979.003.073.735	292.033.119.351.262	68.564.297.586.818
510.767.300.987.835	164.351.736.366.247	385.958.839.536.775	531.362.756.672.829
377.813.138.754.613	996.783.600.118.552	352.089.691.009.618	691.317.658.146.738
247.737.718.146.618	16.279.907.192.492	955.559.769.994.098	283.128.820.738.992
125.039.046.842.664	12.461.229.241.658	158.752.646.503.314	187.771.947.477.038
115.905.652.803.546	150.380.154.663.062	146.219.438.921.396	380.408.296.380.868
582.713.290.795.544	801.230.774.843.872	167.530.071.103.719	582.713.290.795.544
289.788.501.683.208	211.131.622.654.909	106.393.778.475.121	256.669.815.776.555

SMC3-mRNA	IL23R-mRNA	PIK3CB-mRNA	ITGB6-mRNA
766.753.898.115.724	612.911.906.637.312	554.415.656.565.196	543.724.136.173.545
712.066.273.895.995	0.562242025691289	533.042.635.046.821	188.417.012.057.865
686.093.104.873.484	457.552.882.987.259	516.049.133.059.375	47.454.538.313.149
747.797.683.304.815	210.874.302.338.244	569.370.552.410.359	406.810.103.888.509
754.417.047.747.852	398.835.432.241.688	509.938.563.480.562	351.442.313.408.447
765.997.716.635.247	324.395.990.809.479	548.858.227.681.471	330.536.045.275.894
726.997.461.740.552	368.501.211.668.436	559.190.271.229.288	359.190.271.229.288
749.581.205.088.838	528.281.832.755.419	621.570.413.169.565	540.834.920.963.804
773.726.230.601.914	247.191.773.949.815	559.165.698.377.224	409.340.611.624.442
758.039.368.349.335	124.947.680.537.873	527.184.461.840.719	364.179.422.815.749
756.515.402.974.792	603.463.931.304.914	667.206.923.366.443	656.515.402.974.792
728.988.785.449.891	0.72764543027784	51.199.628.530.566	0.72764543027784

SMC3-mRNA	IL23R-mRNA	PIK3CB-mRNA	ITGB6-mRNA
203.310.227.464.674	699.920.455.206.256	466.613.636.804.171	433.284.091.318.159
139.165.977.181.813	147.656.209.211.473	402.363.170.101.264	369.140.523.028.683
116.237.441.955.939	238.435.778.371.156	357.653.667.556.734	26.824.025.066.755
178.277.006.711.338	431.315.338.817.754	517.578.406.581.305	167.733.742.873.571
186.647.254.541.893	158.713.651.821.338	34.282.148.793.409	114.273.829.311.363
202.247.374.826.635	947.390.961.509.695	448.980.933.932.855	988.581.872.879.682
154.340.686.469.969	128.617.238.724.975	482.314.645.218.655	120.578.661.304.664
180.494.623.221.107	389.302.128.516.114	743.213.154.439.854	424.693.231.108.488
213.377.213.042.089	554.780.753.909.432	482.232.501.475.121	170.701.770.433.671
191.392.924.116.624	237.755.185.238.043	386.352.176.011.819	124.821.472.249.972
189.381.819.508.552	655.552.452.144.987	10.197.482.588.922	946.909.097.542.759
156.485.790.908.932	165.593.429.533.262	347.746.202.019.849	165.593.429.533.262

KMT2D-mRNA	FOXL2-mRNA	FANCF-mRNA	CASP10-mRNA
754.415.656.565.196	543.724.136.173.545	678.119.576.295.281	619.623.326.223.166
780.541.600.916.424	477.169.539.132.024	486.602.277.386.839	246.913.262.129.981
811.468.764.098.062	770.481.184.681.756	596.784.625.265.135	516.049.133.059.375
804.734.247.871.829	226.074.611.682.749	585.768.125.921.472	369.370.552.410.359
848.660.518.994.471	404.493.785.078.325	51.773.881.468.069	392.946.063.336.331
858.613.122.288.954	487.014.507.154.246	524.395.990.809.479	392.985.131.766.673
794.710.696.205.454	454.299.311.181.193	493.293.963.012.795	414.444.373.532.166
823.277.764.505.459	628.281.832.755.419	582.338.670.891.689	49.933.117.103.592
787.476.582.976.907	285.894.086.260.739	592.122.514.086.174	285.894.086.260.739
79.807.958.364.038	333.693.964.662.907	524.947.680.537.873	324.947.680.537.873
803.463.931.304.914	486.471.431.160.683	586.471.431.160.683	544.967.681.232.799
747.247.926.777.739	456.053.544.444.258	528.223.428.195.548	30.495.735.251.652

KMT2D-mRNA	FOXL2-mRNA	FANCF-mRNA	CASP10-mRNA
186.645.454.721.668	433.284.091.318.159	10.998.750.010.384	733.250.000.692.268
223.699.156.955.382	273.163.987.041.226	29.162.101.319.266	553.710.784.543.025
277.181.592.356.469	208.631.306.074.761	625.893.918.224.285	357.653.667.556.734
264.540.074.474.889	47.923.926.535.306	579.879.511.077.203	129.394.601.645.326
358.692.853.116.224	165.062.197.894.191	361.867.126.152.651	152.365.105.748.484
384.311.203.081.977	292.455.470.726.906	378.956.384.603.878	152.406.372.068.951
246.784.326.803.545	233.118.745.189.016	305.465.941.971.814	17.684.870.324.684
300.824.372.035.179	778.604.257.032.228	566.257.641.477.984	318.519.923.331.366
234.714.934.346.298	725.482.524.343.103	605.991.285.039.533	725.482.524.343.103
25.261.488.431.542	101.045.953.726.168	380.408.296.380.868	95.102.074.095.217
262.220.980.857.995	291.356.645.397.772	582.713.290.795.544	437.034.968.096.658
177.598.953.174.423	235.970.637.084.898	389.144.559.403.165	827.967.147.666.308

PPP2R2B-mRNA	STK11-mRNA	MAP3K14-mRNA	ETS2-mRNA
473.680.164.359.436	792.662.620.247.438	746.472.209.815.756	766.753.898.115.724
188.417.012.057.865	727.648.754.335.741	524.874.255.287.451	817.511.052.298.233
416.049.133.059.375	772.527.594.937.727	834.371.315.464.952	928.977.434.753.871
210.874.302.338.244	733.542.280.312.198	636.508.277.664.222	936.508.277.664.222
325.138.872.825.068	710.604.936.508.664	792.569.871.456.711	90.239.782.321.476
26.272.885.476.463	720.421.372.930.204	807.574.904.846.259	872.461.841.835.598
349.236.703.874.197	798.422.013.507.164	767.369.680.345.653	918.086.714.357.153
282.338.670.891.689	691.084.955.016.723	607.131.422.236.047	972.425.351.689.764
194.140.302.279.937	697.115.036.619.342	668.436.735.758.702	898.822.387.955.236
157.140.490.026.609	707.602.529.266.965	684.938.964.756.586	968.410.503.301.546
586.471.431.160.683	77.226.953.067.344	738.827.626.766.384	864.607.402.513.149
1.312.607.930.999	735.335.427.334.231	601.304.764.914.009	845.045.296.144.739

PPP2R2B-mRNA	STK11-mRNA	MAP3K14-mRNA	ETS2-mRNA
266.636.363.888.098	243.305.682.047.889	176.646.591.075.865	203.310.227.464.674
369.140.523.028.683	155.039.019.672.047	380.214.738.719.544	289.037.029.531.459
178.826.833.778.367	211.611.753.304.401	3.248.687.480.307	625.893.918.224.285
431.315.338.817.754	161.503.632.423.981	824.291.536.407.263	659.433.229.125.811
952.281.910.928.028	137.763.449.780.921	24.314.931.459.029	520.580.777.973.989
617.863.670.549.801	147.463.462.704.552	269.800.469.473.413	423.030.659.769.764
112.540.083.884.353	253.215.188.739.794	204.179.866.475.897	580.385.289.746.448
70.782.205.184.748	120.329.748.814.072	672.430.949.255.106	845.847.351.957.739
38.407.898.347.576	125.465.801.268.748	102.847.816.686.287	507.837.767.040.172
297.193.981.547.553	134.926.067.622.589	115.311.264.840.451	822.632.940.923.627
582.713.290.795.544	211.233.567.913.384	167.530.071.103.719	400.615.387.421.936
248.390.144.299.892	163.523.511.664.096	64.581.437.517.972	349.816.119.889.015

DKK4-mRNA	H3F3A-mRNA	FASLG-mRNA	POLR2D-mRNA
612.911.906.637.312	101.880.127.554.267	505.872.973.848.172	598.472.915.703.794
0.884170120578651	112.375.375.247.853	114.720.452.641.244	662.293.795.737.884
489.745.692.475.995	951.216.676.887.516	389.745.692.475.995	689.745.692.475.995
318.674.553.538.371	112.579.255.977.651	193.881.802.194.012	625.170.097.722.448
351.442.313.408.447	106.970.933.637.765	351.442.313.408.447	600.270.961.539.395
317.982.957.067.508	108.390.146.413.893	376.479.207.139.623	588.026.928.881.617
407.732.953.946.312	1.041.037.837.458	326.997.461.740.552	595.179.865.737.926
573.027.730.452.541	109.240.490.479.221	463.074.163.097.449	668.136.770.404.446
157.883.294.341.466	109.854.935.127.679	335.644.052.207.821	622.268.913.318.938
370.890.842.401.603	110.176.611.301.557	241.940.180.682.104	638.902.815.777.752
544.967.681.232.799	963.289.863.638.376	518.664.240.649.419	603.463.931.304.914
10.495.735.251.652	104.119.557.414.022	153.500.035.233.544	641.414.595.746.106

DKK4-mRNA	H3F3A-mRNA	FASLG-mRNA	POLR2D-mRNA
699.920.455.206.256	116.653.409.201.043	333.295.454.860.122	633.261.364.234.232
184.570.261.514.342	241.454.816.113.062	22.148.431.381.721	985.605.196.486.584
298.044.722.963.945	730.209.571.261.665	149.022.361.481.972	119.217.889.185.578
910.554.604.170.814	244.891.264.595.413	383.391.412.282.448	761.990.431.911.366
114.273.829.311.363	16.601.447.980.512	114.273.829.311.363	641.203.153.358.206
906.200.050.139.708	183.175.982.862.331	135.930.007.520.956	58.903.003.259.081
168.810.125.826.529	136.093.115.725.864	964.629.290.437.309	61.897.046.136.394
53.086.653.888.561	194.297.153.232.133	247.737.718.146.618	102.634.197.517.885
298.728.098.258.925	202.751.027.832.593	102.421.062.260.203	746.820.245.647.312
130.765.351.880.923	207.322.521.527.573	534.949.166.785.596	8.380.870.279.641
437.034.968.096.658	793.946.858.708.928	364.195.806.747.215	655.552.452.144.987
206.991.786.916.577	136.241.994.148.491	289.788.501.683.208	852.806.162.096.297

BDNF-mRNA	CD40-mRNA	SUV39H2-mRNA	SPRY4-mRNA
490.672.664.503.667	554.415.656.565.196	573.680.164.359.436	573.680.164.359.436
411.683.087.736.893	402.167.364.432.859	526.268.174.383.238	602.984.757.577.429
489.745.692.475.995	389.745.692.475.995	548.241.942.548.111	548.241.942.548.111
369.370.552.410.359	534.820.895.807.782	514.827.138.756.907	716.281.969.613.823
44.319.609.738.925	54.319.609.738.925	495.920.797.675.736	564.827.888.081.926
372.039.795.203.778	519.613.138.300.418	484.968.096.898.275	619.613.138.300.418
538.545.183.482.545	529.972.196.079.957	5.239.600.968.362	667.369.680.345.653
540.834.920.963.804	414.531.480.380.425	552.382.642.705.798	707.131.422.236.047
285.894.086.260.739	571.399.252.669.629	501.940.553.480.064	583.756.721.181.483
283.443.930.609.989	601.766.113.015.566	497.739.725.994.193	645.893.017.100.768
586.471.431.160.683	486.471.431.160.683	618.664.240.649.419	518.664.240.649.419
153.500.035.233.544	525.120.738.633.485	45.090.051.438.025	652.854.533.019.814

BDNF-mRNA	CD40-mRNA	SUV39H2-mRNA	SPRY4-mRNA
29.996.590.937.411	466.613.636.804.171	533.272.727.776.195	533.272.727.776.195
173.496.045.823.481	162.421.830.132.621	38.390.614.394.983	653.378.725.760.769
298.044.722.963.945	149.022.361.481.972	447.067.084.445.918	447.067.084.445.918
129.394.601.645.326	407.353.375.550.101	354.637.056.361.264	143.292.540.340.565
21.585.056.647.702	431.701.132.954.039	311.078.757.569.823	501.535.139.755.428
131.810.916.383.958	366.599.111.192.882	288.336.379.589.907	733.198.222.385.764
418.006.025.856.167	393.890.293.595.235	377.813.138.754.613	102.089.933.237.949
424.693.231.108.488	17.695.551.296.187	460.084.333.700.862	134.486.189.851.021
725.482.524.343.103	524.907.944.083.539	324.333.363.823.975	571.850.930.952.799
713.265.555.714.128	647.882.879.773.666	315.025.620.440.406	879.694.185.380.758
582.713.290.795.544	291.356.645.397.772	728.391.613.494.429	364.195.806.747.215
289.788.501.683.208	380.864.887.926.502	227.690.965.608.235	923.183.369.647.933

HMGA2-mRNA	LAMC3-mRNA	NPM1-mRNA	ID4-mRNA
543.724.136.173.545	505.872.973.848.172	598.472.915.703.794	632.176.414.431.551
236.959.694.774.889	618.429.384.514.767	40.858.039.817.483	423.466.736.766.278
503.496.044.850.989	47.454.538.313.149	648.241.942.548.111	780.434.752.036.847
274.617.294.399.773	452.378.052.266.128	498.321.214.129.858	613.849.036.677.649
429.869.444.302.903	409.938.563.480.562	549.424.525.214.684	709.269.098.223.162
342.083.767.017.887	400.580.017.090.003	512.978.888.817.548	844.320.548.320.733
432.886.830.645.909	514.444.373.532.166	543.989.961.884.783	67.724.749.579.347
540.834.920.963.804	482.338.670.891.689	482.338.670.891.689	646.724.289.869.161
235.644.052.207.821	235.644.052.207.821	472.567.433.174.393	682.132.657.080.762
283.443.930.609.989	241.940.180.682.104	486.418.664.949.394	744.423.365.980.098
444.967.681.232.799	603.463.931.304.914	486.471.431.160.683	586.471.431.160.683
189.757.043.172.015	325.120.738.633.485	465.838.276.784.073	772.764.543.027.784

HMGA2-mRNA	LAMC3-mRNA	NPM1-mRNA	ID4-mRNA
433.284.091.318.159	333.295.454.860.122	633.261.364.234.232	799.909.091.664.293
516.796.732.240.156	727.206.830.366.506	169.804.640.593.194	188.261.666.744.628
32.784.919.526.034	26.824.025.066.755	894.134.168.891.835	223.533.542.222.959
670.934.971.494.284	230.034.847.369.469	31.629.791.513.302	704.481.720.068.998
196.804.928.258.459	171.410.743.967.045	4.507.467.711.726	136.493.740.566.351
107.096.369.561.965	160.644.554.342.948	350.122.746.644.887	348.063.201.076.388
200.964.435.507.773	35.369.740.649.368	434.083.180.696.789	109.324.652.916.228
424.693.231.108.488	283.128.820.738.992	283.128.820.738.992	88.477.756.480.935
512.105.311.301.014	512.105.311.301.014	26.458.774.417.219	113.089.922.912.307
713.265.555.714.128	534.949.166.785.596	291.250.101.916.602	174.155.673.186.866
218.517.484.048.329	655.552.452.144.987	291.356.645.397.772	582.713.290.795.544
372.585.216.449.838	952.162.219.816.254	252.529.980.038.224	211.959.589.802.575

IL22RA2-mRNA	PKMYT1-mRNA	EP300-mRNA	FGF23-mRNA
532.176.414.431.551	490.672.664.503.667	754.415.656.565.196	564.369.223.920.288
0.884170120578651	326.268.174.383.238	765.763.904.848.385	214.720.452.641.244
457.552.882.987.259	489.745.692.475.995	780.434.752.036.847	457.552.882.987.259
293.881.802.194.012	426.074.611.682.749	767.013.705.296.519	193.881.802.194.012
404.493.785.078.325	398.835.432.241.688	790.291.884.591.082	359.242.564.608.574
342.083.767.017.887	347.528.545.420.125	773.162.520.746.104	317.982.957.067.508
338.545.183.482.545	385.493.711.812.667	76.275.266.220.236	400.694.021.157.172
340.834.920.963.804	382.338.670.891.689	829.912.013.988.329	514.531.480.380.425
0.356440522078209	194.140.302.279.937	795.138.711.137.199	135.644.052.207.821
-0.750523194621269	157.140.490.026.609	810.434.518.863.897	-0.750523194621269
567.206.923.366.443	486.471.431.160.683	750.857.050.138.155	603.463.931.304.914
0.312607930998996	218.707.704.891.514	798.031.086.272.809	389.757.043.172.015

IL22RA2-mRNA	PKMYT1-mRNA	EP300-mRNA	FGF23-mRNA
399.954.545.832.146	29.996.590.937.411	186.645.454.721.668	499.943.182.290.183
184.570.261.514.342	959.765.359.874.576	20.191.986.609.669	44.296.862.763.442
238.435.778.371.156	298.044.722.963.945	223.533.542.222.959	238.435.778.371.156
766.782.824.564.896	191.695.706.141.224	203.676.687.775.051	383.391.412.282.448
165.062.197.894.191	158.713.651.821.338	239.340.186.946.578	120.622.375.384.217
107.096.369.561.965	111.215.460.698.964	212.545.102.669.131	906.200.050.139.708
104.501.506.464.042	144.694.393.565.596	197.749.004.539.648	160.771.548.406.218
106.173.307.777.122	141.564.410.369.496	314.980.813.072.129	35.391.102.592.374
128.026.327.825.253	38.407.898.347.576	247.517.567.128.823	256.052.655.650.507
0.594387963095106	297.193.981.547.553	275.201.626.913.034	0.594387963095106
509.874.129.446.101	291.356.645.397.772	182.097.903.373.607	655.552.452.144.987
124.195.072.149.946	455.381.931.216.469	252.529.980.038.224	149.034.086.579.935

**CUL1-mRNA**	**TNR-mRNA**	**DKK2-mRNA**	**MAPK3-mRNA**
712.911.906.637.312	564.369.223.920.288	543.724.136.173.545	698.472.915.703.794
721.687.805.421.926	226.268.174.383.238	522.045.350.844.308	726.268.174.383.238
643.350.982.500.016	516.049.133.059.375	596.784.625.265.135	70.990.907.859.296
759.702.950.469.192	318.674.553.538.371	293.881.802.194.012	751.246.520.943.344
725.138.872.825.068	434.449.813.264.216	415.185.305.469.976	773.681.555.542.092
777.296.600.284.193	336.425.414.181.251	414.666.270.673.988	8.240.034.204.881
736.449.221.618.981	407.732.953.946.312	554.299.311.181.193	697.041.433.554.661
83.469.486.649.739	573.027.730.452.541	514.531.480.380.425	59.933.117.103.592
752.302.208.044.615	194.140.302.279.937	277.147.802.135.705	764.184.274.094.046
758.932.680.826.336	305.683.172.743.634	270.890.842.401.603	760.262.363.087.681
756.515.402.974.792	603.463.931.304.914	544.967.681.232.799	667.206.923.366.443
74.589.644.613.029	2.312.607.930.999	281.510.827.152.818	765.245.793.388.362

**CUL1-mRNA**	**TNR-mRNA**	**DKK2-mRNA**	**MAPK3-mRNA**
139.984.091.041.251	499.943.182.290.183	433.284.091.318.159	126.652.272.846.846
148.763.630.780.559	479.882.679.937.288	37.283.192.825.897	153.562.457.579.932
86.432.969.659.544	357.653.667.556.734	625.893.918.224.285	137.100.572.563.415
193.612.663.202.636	910.554.604.170.814	766.782.824.564.896	182.590.160.099.516
152.365.105.748.485	203.153.474.331.313	177.759.290.039.899	213.311.148.047.878
218.723.739.374.629	102.977.278.424.967	177.120.918.890.943	302.341.289.455.703
164.790.837.116.374	168.810.125.826.529	466.237.490.378.033	12.540.180.775.685
325.598.143.849.841	53.086.653.888.561	35.391.102.592.374	637.039.846.662.732
183.931.157.642.281	38.407.898.347.576	682.807.081.734.685	199.721.071.407.395
192.581.700.042.814	832.143.148.333.149	653.826.759.404.617	1.943.648.639.321
189.381.819.508.552	655.552.452.144.987	437.034.968.096.658	10.197.482.588.922
17.594.301.887.909	496.780.288.599.784	703.772.075.516.361	201.196.016.882.913

BCOR-mRNA	IL3RA-mRNA	GTF2H3-mRNA	DLL3-mRNA
612.911.906.637.312	505.872.973.848.172	564.369.223.920.288	454.415.656.565.196
611.683.087.736.893	436.959.694.774.889	618.429.384.514.767	-0.437757974308711
596.784.625.265.135	457.552.882.987.259	538.288.375.193.019	316.049.133.059.375
583.363.578.524.807	410.874.302.338.244	592.750.270.871.229	193.881.802.194.012
601.692.347.461.365	498.835.432.241.688	555.395.149.827.111	351.442.313.408.447
567.459.426.242.466	414.666.270.673.988	588.026.928.881.617	252.775.287.409.539
520.857.407.274.137	426.997.461.740.552	511.127.687.138.646	226.997.461.740.552
710.878.892.777.914	591.084.955.016.723	482.338.670.891.689	440.834.920.963.804
576.016.270.812.922	372.567.433.174.393	583.756.721.181.483	0.771478021357053
615.636.740.098.725	43.787.598.223.237	618.021.414.294.162	157.140.490.026.609
686.471.431.160.683	518.664.240.649.419	518.664.240.649.419	518.664.240.649.419
601.304.764.914.009	325.120.738.633.485	584.658.650.300.135	0.312607930998996

BCOR-mRNA	IL3RA-mRNA	GTF2H3-mRNA	DLL3-mRNA
699.920.455.206.256	333.295.454.860.122	499.943.182.290.183	233.306.818.402.085
693.984.183.293.924	206.718.692.896.062	727.206.830.366.506	0.738281046057366
625.893.918.224.285	238.435.778.371.156	417.262.612.149.523	894.134.168.891.835
570.294.725.770.142	172.526.135.527.102	608.633.866.998.386	383.391.412.282.448
647.551.699.431.059	317.427.303.642.676	469.792.409.391.161	114.273.829.311.363
510.767.300.987.835	177.120.918.890.943	58.903.003.259.081	576.672.759.179.814
369.774.561.334.302	192.925.858.087.462	345.658.829.073.369	482.314.645.218.654
138.025.300.110.259	601.648.744.070.358	283.128.820.738.992	212.346.615.554.244
541.978.121.126.906	132.293.872.086.095	571.850.930.952.799	170.701.770.433.671
713.265.555.714.128	208.035.787.083.287	72.515.331.497.603	297.193.981.547.553
116.542.658.159.109	364.195.806.747.215	364.195.806.747.215	364.195.806.747.215
64.581.437.517.972	952.162.219.816.254	575.437.167.628.084	124.195.072.149.946

HNF1A-mRNA	CXXC4-mRNA	MED12-mRNA	RNF43-mRNA
58.242.644.848.447	505.872.973.848.172	543.724.136.173.545	332.176.414.431.551
136.959.694.774.889	114.720.452.641.244	607.003.666.588.998	17.321.670.271.336
47.454.538.313.149	47.454.538.313.149	548.241.942.548.111	257.552.882.987.259
152.378.052.266.128	369.370.552.410.359	563.925.774.008.121	333.113.544.471.888
373.681.555.542.092	386.806.008.869.917	620.247.912.776.973	373.681.555.542.092
317.982.957.067.508	289.032.295.348.009	595.880.269.136.286	317.982.957.067.508
368.501.211.668.436	338.545.183.482.545	554.299.311.181.193	432.886.830.645.909
528.281.832.755.419	49.933.117.103.592	552.382.642.705.798	340.834.920.963.804
109.340.611.624.442	372.567.433.174.393	626.333.111.768.673	390.076.103.830.202
157.140.490.026.609	315.636.740.098.725	641.940.180.682.104	294.991.652.351.982
518.664.240.649.419	444.967.681.232.799	667.206.923.366.443	286.471.431.160.683
1.312.607.930.999	3.312.607.930.999	598.503.327.297.049	1.312.607.930.999

HNF1A-mRNA	CXXC4-mRNA	MED12-mRNA	RNF43-mRNA
566.602.273.262.207	333.295.454.860.122	433.284.091.318.159	999.886.364.580.366
258.398.366.120.078	22.148.431.381.721	671.835.751.912.203	332.226.470.725.815
26.824.025.066.755	26.824.025.066.755	447.067.084.445.918	59.608.944.592.789
287.543.559.211.836	129.394.601.645.326	498.408.835.967.182	100.640.245.724.143
133.319.467.529.924	146.016.559.675.631	736.431.344.451.008	133.319.467.529.924
906.200.050.139.708	741.436.404.659.761	621.982.761.686.799	906.200.050.139.708
128.617.238.724.975	104.501.506.464.042	466.237.490.378.033	200.964.435.507.773
389.302.128.516.114	318.519.923.331.366	460.084.333.700.862	106.173.307.777.122
213.377.213.042.089	132.293.872.086.095	768.157.966.951.521	149.364.049.129.462
297.193.981.547.553	89.158.194.464.266	855.918.666.856.953	772.704.352.023.638
364.195.806.747.215	218.517.484.048.329	10.197.482.588.922	72.839.161.349.443
248.390.144.299.892	993.560.577.199.569	633.394.867.964.725	248.390.144.299.892

WNT10A-mRNA	MAP3K8-mRNA	STAT3-mRNA	ARID1B-mRNA
490.672.664.503.667	612.911.906.637.312	822.865.473.992.403	686.608.466.053.932
136.959.694.774.889	414.720.452.641.244	933.455.659.961.294	771.706.013.474.339
503.496.044.850.989	548.241.942.548.111	901.847.232.572.132	743.350.982.500.016
439.824.964.057.742	561.124.336.391.162	966.758.887.148.278	764.272.159.538.479
315.185.305.469.976	615.185.305.469.976	91.143.359.762.716	748.404.948.504.095
217.982.957.067.508	655.960.174.013.472	9.036.679.483.784	735.702.257.258.143
400.694.021.157.172	684.488.345.346.275	936.800.670.036.605	743.320.496.627.382
382.338.670.891.689	668.136.770.404.446	816.323.671.180.151	755.130.716.348.009
257.883.294.341.466	701.465.200.483	880.765.163.391.054	785.362.706.271.092
183.443.930.609.989	724.947.680.537.873	932.762.761.311.338	789.693.523.183.365
567.206.923.366.443	695.217.715.285.717	711.264.182.505.042	738.827.626.766.384
172.764.543.027.784	538.585.691.302.963	921.749.339.071.714	761.334.180.361.723

WNT10A-mRNA	MAP3K8-mRNA	STAT3-mRNA	ARID1B-mRNA
29.996.590.937.411	699.920.455.206.256	29.996.590.937.411	116.653.409.201.043
258.398.366.120.078	177.187.451.053.768	645.626.774.777.167	210.410.098.126.349
32.784.919.526.034	447.067.084.445.918	518.597.817.957.265	172.865.939.319.088
210.865.276.755.346	488.824.050.660.121	813.269.033.304.142	199.842.773.652.226
888.796.450.199.493	711.037.160.159.594	554.228.072.160.113	179.028.999.254.469
453.100.025.069.854	943.271.870.372.696	525.184.119.967.331	163.939.827.252.547
160.771.548.406.218	114.951.657.110.446	660.771.063.949.557	172.829.414.536.685
141.564.410.369.496	102.634.197.517.885	286.667.930.998.229	187.572.843.739.582
59.745.619.651.785	129.306.591.103.506	448.092.147.388.387	231.300.898.937.624
356.632.777.857.064	152.163.318.552.347	64.253.338.810.581	238.349.573.201.138
509.874.129.446.101	123.826.574.294.053	138.394.406.563.942	167.530.071.103.719
331.186.859.066.523	418.123.409.571.485	595.308.379.172.075	195.814.230.423.082

POLR2J-mRNA	HES5-mRNA	IL1R2-mRNA	COL27A1-mRNA
590.672.664.503.667	598.472.915.703.794	473.680.164.359.436	919.623.326.223.165
462.833.121.614.906	346.913.262.129.981	256.224.202.569.129	794.378.497.687.587
638.288.375.193.019	516.049.133.059.375	416.049.133.059.375	119.374.726.036.078
533.113.544.471.888	402.628.086.319.046	293.881.802.194.012	824.031.421.692.267
575.388.906.877.986	520.247.912.776.973	334.449.813.264.216	946.214.123.403.125
583.933.902.476.129	36.272.885.476.463	421.225.104.836.746	942.430.152.548.244
538.545.183.482.545	349.236.703.874.197	546.637.183.020.902	100.469.558.904.196
686.778.082.827.534	63.469.486.649.739	540.834.920.963.804	728.281.832.755.419
492.122.514.086.174	372.567.433.174.393	277.147.802.135.705	712.903.002.597.514
492.190.214.735.023	341.940.180.682.104	370.890.842.401.603	769.656.003.158.838
586.471.431.160.683	544.967.681.232.799	586.471.431.160.683	822.226.631.622.491
503.142.617.845.494	281.510.827.152.818	3.312.607.930.999	777.203.954.963.629

POLR2J-mRNA	HES5-mRNA	IL1R2-mRNA	COL27A1-mRNA
599.931.818.748.219	633.261.364.234.232	266.636.363.888.098	586.600.000.553.814
247.324.150.429.218	110.742.156.908.605	590.624.836.845.893	246.216.728.860.132
834.525.224.299.046	357.653.667.556.734	178.826.833.778.367	392.226.855.420.552
402.560.982.896.571	16.294.135.022.004	766.782.824.564.896	302.399.976.437.781
539.626.416.192.549	368.215.672.225.504	101.576.737.165.656	705.323.468.694.026
572.553.668.042.816	12.357.273.410.996	18.535.910.116.494	687.064.401.651.379
418.006.025.856.167	112.540.083.884.353	4.421.217.581.171	105.787.678.851.292
116.790.638.554.834	813.995.359.624.602	424.693.231.108.488	155.720.851.406.446
302.995.642.519.767	132.293.872.086.095	682.807.081.734.685	13.997.545.175.561
303.137.861.178.504	106.989.833.357.119	130.765.351.880.923	207.441.399.120.192
582.713.290.795.544	437.034.968.096.658	582.713.290.795.544	298.640.561.532.716
327.047.023.328.192	703.772.075.516.361	993.560.577.199.569	218.583.326.983.905

H3F3C-mRNA	CNTFR-mRNA	ACVR2A-mRNA	HIST1H3G-mRNA
75.696.916.577.591	490.672.664.503.667	68.242.644.848.447	58.242.644.848.447
883.370.505.359.566	320.609.821.546.601	545.705.978.899.923	583.836.643.096.553
759.789.664.290.104	82.048.854.499.522	566.299.167.112.293	47.454.538.313.149
995.004.527.736.338	263.925.774.008.122	529.637.002.655.821	580.918.274.152.353
836.963.769.492.066	477.076.288.734.425	547.378.114.958.712	415.185.305.469.976
831.098.500.195.282	59.102.225.109.178	554.057.691.445.297	455.328.796.620.252
827.746.915.395.244	616.074.554.765.076	538.545.183.482.545	438.545.183.482.545
953.763.222.658.301	49.933.117.103.592	463.074.163.097.449	59.933.117.103.592
986.159.044.102.134	612.903.002.597.514	556.589.388.770.716	223.090.963.999.435
991.658.834.745.376	784.193.384.264.681	565.886.774.151.643	333.693.964.662.907
847.942.415.572.204	486.471.431.160.683	618.664.240.649.419	618.664.240.649.419
969.270.818.702.247	650.243.248.987.901	554.782.439.269.303	393.709.879.590.679

H3F3C-mRNA	CNTFR-mRNA	ACVR2A-mRNA	HIST1H3G-mRNA
189.978.409.270.269	29.996.590.937.411	113.320.454.652.441	566.602.273.262.207
456.257.686.463.452	922.851.307.571.707	439.277.222.404.133	572.167.810.694.459
193.729.069.926.564	295.064.275.734.306	506.676.029.038.706	26.824.025.066.755
989.149.843.688.715	623.011.044.958.978	392.976.197.589.509	56.070.994.046.308
330.759.250.395.668	272.987.481.132.701	444.398.225.099.746	177.759.290.039.899
317.581.926.662.598	601.387.306.001.806	46.545.729.848.085	234.788.194.808.924
310.289.088.424.001	715.433.390.407.671	418.006.025.856.167	209.003.012.928.084
743.213.154.439.854	318.519.923.331.366	247.737.718.146.618	637.039.846.662.732
930.324.648.863.509	699.877.258.778.052	473.697.412.953.438	469.429.868.692.596
966.474.827.992.642	229.433.753.754.711	50.522.976.863.084	101.045.953.726.168
35.691.189.061.227	291.356.645.397.772	728.391.613.494.429	728.391.613.494.429
827.553.164.092.475	906.624.026.694.607	467.801.438.431.464	153.173.922.318.267

RASGRP2-mRNA	TSPAN7-mRNA	LFNG-mRNA	BRIP1-mRNA
532.176.414.431.551	632.176.414.431.551	678.119.576.295.281	598.472.915.703.794
391.979.403.030.937	429.016.248.025.449	527.648.754.335.741	414.720.452.641.244
538.288.375.193.019	780.434.752.036.847	724.795.417.184.409	489.745.692.475.995
463.925.774.008.121	625.170.097.722.448	626.074.611.682.749	379.679.901.706.769
580.392.975.127.945	637.792.113.417.961	672.820.242.511.621	473.681.555.542.092
669.194.150.598.855	7.762.057.103.675	685.994.930.443.657	404.232.604.692.514
563.920.842.707.124	554.299.311.181.193	616.074.554.765.076	432.886.830.645.909
49.933.117.103.592	59.933.117.103.592	514.531.480.380.425	582.338.670.891.689
652.636.552.352.052	657.237.792.127.736	673.147.995.342.513	316.379.544.413.581
641.940.180.682.104	764.179.422.815.749	620.367.311.576.561	305.683.172.743.634
677.160.490.721.535	286.471.431.160.683	603.463.931.304.914	567.206.923.366.443
680.979.447.163.171	754.462.905.353.322	477.203.954.963.629	337.150.162.005.256

RASGRP2-mRNA	TSPAN7-mRNA	LFNG-mRNA	BRIP1-mRNA
399.954.545.832.146	799.909.091.664.293	10.998.750.010.384	633.261.364.234.232
15.134.761.444.176	195.644.477.205.202	387.597.549.180.117	177.187.451.053.768
417.262.612.149.523	223.533.542.222.959	152.002.808.711.612	298.044.722.963.945
249.204.417.983.591	761.990.431.911.366	766.782.824.564.896	138.979.386.952.387
55.867.205.441.111	831.659.535.543.811	106.020.719.416.654	266.638.935.059.848
103.389.187.538.667	21.707.610.291.983	116.158.370.063.363	164.763.645.479.947
498.391.800.059.276	466.237.490.378.033	715.433.390.407.671	200.964.435.507.773
318.519.923.331.366	637.039.846.662.732	35.391.102.592.374	566.257.641.477.984
921.789.560.341.825	951.662.370.167.717	10.626.185.209.496	896.184.294.776.775
855.918.666.856.953	199.714.355.599.956	737.041.074.237.932	832.143.148.333.149
109.258.742.024.164	72.839.161.349.443	655.552.452.144.987	509.874.129.446.101
112.189.548.508.785	186.706.591.798.752	273.229.158.729.881	103.495.893.458.288

CLCF1-mRNA	HHIP-mRNA	POLR2H-mRNA	MAPK8IP2-mRNA
173.680.164.359.436	273.680.164.359.436	702.220.386.245.661	519.623.326.223.166
114.720.452.641.244	226.268.174.383.238	695.455.944.847.005	214.720.452.641.244
257.552.882.987.259	316.049.133.059.375	71.902.386.739.878	516.049.133.059.375
174.617.294.399.773	263.925.774.008.122	756.452.686.500.459	393.881.802.194.012
215.185.305.469.976	292.946.063.336.331	738.341.712.193.446	425.138.872.825.068
152.775.287.409.539	172.039.795.203.778	687.521.606.108.989	330.536.045.275.894
0.685012116684361	226.997.461.740.552	671.843.511.822.181	407.732.953.946.312
182.338.670.891.689	182.338.670.891.689	582.338.670.891.689	463.074.163.097.449
0.771478021357053	247.191.773.949.815	693.638.494.803.274	285.894.086.260.739
205.683.172.743.634	441.940.180.682.104	686.418.664.949.394	283.443.930.609.989
386.471.431.160.683	386.471.431.160.683	725.703.173.438.559	486.471.431.160.683
0.312607930998996	2.312.607.930.999	653.500.035.233.544	297.557.294.372.143

CLCF1-mRNA	HHIP-mRNA	POLR2H-mRNA	MAPK8IP2-mRNA
333.295.454.860.122	666.590.909.720.244	129.985.227.395.448	366.625.000.346.134
22.148.431.381.721	479.882.679.937.288	124.031.215.737.637	44.296.862.763.442
59.608.944.592.789	894.134.168.891.835	146.041.914.252.333	357.653.667.556.734
335.467.485.747.142	623.011.044.958.978	189.299.509.814.459	153.356.564.912.979
444.398.225.099.746	761.825.528.742.422	166.966.761.716.048	190.456.382.185.606
288.336.379.589.907	329.527.290.959.894	117.394.097.404.462	988.581.872.879.682
160.771.548.406.218	482.314.645.218.654	105.305.364.206.073	168.810.125.826.529
35.391.102.592.374	35.391.102.592.374	566.257.641.477.984	247.737.718.146.618
170.701.770.433.671	554.780.753.909.432	122.478.520.286.159	725.482.524.343.103
416.071.574.166.575	213.979.666.714.238	116.500.040.766.641	713.265.555.714.128
145.678.322.698.886	145.678.322.698.886	15.296.223.883.383	291.356.645.397.772
124.195.072.149.946	496.780.288.599.784	927.323.205.386.264	786.568.790.282.992

STMN1-mRNA	RASGRP1-mRNA	PTCH1-mRNA	SMAD4-mRNA
659.478.263.872.193	373.680.164.359.436	564.369.223.920.288	764.369.223.920.288
644.488.507.505.313	246.913.262.129.981	683.370.505.359.566	815.282.907.560.632
516.049.133.059.375	157.552.882.987.259	703.496.044.850.989	793.308.083.449.067
626.074.611.682.749	458.267.421.171.485	686.955.535.950.301	81.137.437.044.408
621.486.285.222.556	404.493.785.078.325	634.449.813.264.216	790.674.055.686.323
631.285.498.930.586	46.976.778.755.377	560.304.100.139.962	817.777.883.111.032
55.179.021.308.491	393.293.963.012.795	614.444.373.532.166	818.883.785.468.011
382.338.670.891.689	182.338.670.891.689	563.074.163.097.449	728.281.832.755.419
5.980.931.386.986	362.945.901.648.462	653.302.925.380.153	80.591.904.009.065
63.580.012.621.569	224.947.680.537.873	593.597.733.256.195	821.814.359.857.394
603.463.931.304.914	518.664.240.649.419	444.967.681.232.799	603.463.931.304.914
549.582.975.505.477	253.500.035.233.544	649.582.975.505.477	795.886.661.098.903

STMN1-mRNA	RASGRP1-mRNA	PTCH1-mRNA	SMAD4-mRNA
966.556.819.094.353	133.318.181.944.049	499.943.182.290.183	199.977.272.916.073
871.171.634.347.692	553.710.784.543.025	114.064.421.615.863	284.607.343.255.115
357.653.667.556.734	298.044.722.963.945	131.139.678.104.136	244.396.672.830.435
766.782.824.564.896	23.961.963.267.653	116.934.380.746.147	277.000.295.374.068
742.779.890.523.862	165.062.197.894.191	812.613.897.325.251	239.975.041.553.863
794.984.589.440.744	259.502.741.630.916	486.052.754.165.844	289.572.106.931.007
458.198.912.957.722	152.732.970.985.907	70.739.481.298.736	291.800.360.357.286
141.564.410.369.496	35.391.102.592.374	495.475.436.293.236	155.720.851.406.446
631.596.550.604.584	123.758.783.564.412	926.057.104.602.667	266.721.516.302.611
820.255.389.071.247	475.510.370.476.085	61.221.960.198.796	297.788.369.510.648
655.552.452.144.987	364.195.806.747.215	218.517.484.048.329	655.552.452.144.987
451.242.095.478.137	579.577.003.366.415	902.484.190.956.275	248.804.127.873.726

DUSP5-mRNA	GADD45B-mRNA	PDGFD-mRNA	RPS6KA6-mRNA
775.916.945.662.281	104.916.891.457.578	7.291.390.495.272	564.369.223.920.288
581.962.986.838.394	836.959.694.774.889	988.754.748.078.097	369.152.504.263.625
743.350.982.500.016	977.520.117.470.895	842.101.888.081.697	438.288.375.193.019
796.672.401.851.001	899.002.696.285.489	853.873.086.412.725	402.628.086.319.046
757.811.780.940.186	872.604.108.382.674	826.931.063.624.794	373.681.555.542.092
713.825.046.692.368	104.698.484.176.077	943.635.994.229.292	389.032.295.348.009
818.883.785.468.011	104.390.644.842.133	878.829.992.509.638	37.724.749.579.347
105.782.742.110.804	127.174.465.551.909	755.130.716.348.009	552.382.642.705.798
674.875.794.485.697	866.781.042.526.699	813.998.448.286.475	441.533.421.113.178
774.132.990.170.841	923.389.526.417.987	826.070.406.080.198	424.947.680.537.873
781.891.062.199.371	102.134.424.658.379	632.414.593.024.413	544.967.681.232.799
632.010.246.754.592	904.052.838.556.219	903.370.711.970.618	325.120.738.633.485

DUSP5-mRNA	GADD45B-mRNA	PDGFD-mRNA	RPS6KA6-mRNA
216.642.045.659.079	143.983.636.499.573	156.648.863.784.257	499.943.182.290.183
564.785.000.233.885	3.307.499.086.337	9.472.145.820.916	129.199.183.060.039
172.865.939.319.088	876.251.485.513.998	342.751.431.408.537	208.631.306.074.761
250.162.896.514.297	508.472.860.539.597	371.889.669.913.974	16.294.135.022.004
191.091.236.792.891	42.344.802.305.933	308.539.339.140.681	133.319.467.529.924
140.872.916.885.355	141.820.307.846.864	692.831.129.243.176	148.287.280.931.952
291.800.360.357.286	138.826.232.048.769	4.421.217.581.171	136.655.816.145.285
152.889.563.199.056	673.492.682.332.878	187.572.843.739.582	460.084.333.700.862
107.542.115.373.213	406.696.968.058.222	282.084.675.641.642	213.377.213.042.089
213.979.666.714.238	602.115.006.615.342	306.704.188.957.075	190.204.148.190.434
225.801.400.183.273	118.727.832.999.592	801.230.774.843.872	437.034.968.096.658
798.988.297.497.987	526.587.105.915.771	524.103.204.472.773	952.162.219.816.254

MDM2-mRNA	ITGB4-mRNA	IL24-mRNA	CDC25C-mRNA
678.119.576.295.281	705.872.973.848.172	505.872.973.848.172	473.680.164.359.436
799.687.025.332.801	535.665.789.204.139	226.268.174.383.238	202.167.364.432.859
759.789.664.290.104	682.345.634.331.618	257.552.882.987.259	357.552.882.987.259
847.603.642.247.872	791.895.759.957.928	263.925.774.008.122	263.925.774.008.122
833.601.997.871.785	802.749.271.632.384	166.642.622.752.952	215.185.305.469.976
868.906.474.523.299	709.977.631.910.904	104.232.604.692.514	189.032.295.348.009
825.486.772.501.531	725.486.772.501.531	249.236.703.874.197	226.997.461.740.552
749.581.205.088.838	663.074.163.097.449	514.531.480.380.425	514.531.480.380.425
82.431.532.357.491	85.213.474.487.539	0.356440522078209	0.771478021357053
80.043.643.075.422	888.247.200.252.169	124.947.680.537.873	224.947.680.537.873
811.264.182.505.042	767.206.923.366.443	486.471.431.160.683	444.967.681.232.799
810.051.049.039.043	777.203.954.963.629	10.495.735.251.652	10.495.735.251.652

MDM2-mRNA	ITGB4-mRNA	IL24-mRNA	CDC25C-mRNA
10.998.750.010.384	133.318.181.944.049	333.295.454.860.122	266.636.363.888.098
255.445.241.935.849	409.745.980.561.838	479.882.679.937.288	406.054.575.331.551
193.729.069.926.564	113.256.994.726.299	59.608.944.592.789	119.217.889.185.578
356.074.774.157.323	242.015.829.003.295	623.011.044.958.978	623.011.044.958.978
323.140.995.108.244	26.092.524.359.428	317.427.303.642.676	444.398.225.099.746
412.732.931.927.267	137.165.734.862.056	205.954.556.849.934	370.718.202.329.881
305.465.941.971.815	152.732.970.985.907	562.700.419.421.764	482.314.645.218.654
180.494.623.221.107	990.950.872.586.472	35.391.102.592.374	35.391.102.592.374
302.995.642.519.767	367.435.560.858.477	128.026.327.825.253	170.701.770.433.671
256.775.600.057.086	471.944.042.697.514	237.755.185.238.043	475.510.370.476.085
276.788.813.127.883	20.394.965.177.844	291.356.645.397.772	218.517.484.048.329
274.471.109.451.381	218.583.326.983.905	206.991.786.916.577	206.991.786.916.577

FGFR1-mRNA	PTTG2-mRNA	PLA2G4F-mRNA	KLF4-mRNA
889.667.298.037.275	58.242.644.848.447	573.680.164.359.436	76.194.446.929.562
109.919.102.908.244	423.466.736.766.278	398.850.678.039.339	765.235.444.535.558
1.008.728.148.364	548.241.942.548.111	652.972.514.025.947	661.992.294.923.104
10.690.780.435.401	582.146.107.130.196	302.628.086.319.046	763.578.554.817.441
97.064.419.063.774	555.395.149.827.111	568.434.813.552.678	731.604.168.659.293
10.537.781.379.935	634.244.977.149.416	421.225.104.836.746	659.076.267.162.119
106.622.920.401.843	604.256.412.130.245	407.732.953.946.312	705.133.433.093.018
10.172.114.863.148	628.281.832.755.419	628.281.832.755.419	977.758.301.930.376
994.640.370.385.773	582.676.045.685.824	488.000.247.813.522	779.661.758.363.556
957.027.735.426.037	570.890.842.401.603	497.739.725.994.193	833.693.964.662.907
97.226.953.067.344	586.471.431.160.683	386.471.431.160.683	744.967.681.232.799
109.844.438.163.572	5.312.607.930.999	2.312.607.930.999	808.958.920.401.308

FGFR1-mRNA	PTTG2-mRNA	PLA2G4F-mRNA	KLF4-mRNA
476.612.500.449.974	566.602.273.262.207	533.272.727.776.195	196.644.318.367.472
203.654.826.554.924	188.261.666.744.628	158.730.424.902.334	201.181.585.050.632
10.878.632.388.184	447.067.084.445.918	923.938.641.188.229	983.547.585.781.019
165.289.622.620.271	565.502.333.116.611	814.706.751.100.202	19.888.429.512.152
835.468.663.187.523	469.792.409.391.161	514.232.231.901.135	159.348.506.428.623
148.657.999.134.282	811.460.953.988.739	18.535.910.116.494	96.386.732.605.769
162.057.720.793.468	659.163.348.465.495	168.810.125.826.529	13.263.652.743.513
115.374.994.451.139	778.604.257.032.228	778.604.257.032.228	877.699.344.290.874
98.665.623.310.662	567.583.386.691.957	294.460.553.998.083	222.339.055.989.857
760.222.204.798.642	523.061.407.523.694	315.025.620.440.406	323.347.051.923.738
844.934.271.653.538	582.713.290.795.544	145.678.322.698.886	174.813.987.238.663
202.603.561.033.946	397.424.230.879.828	496.780.288.599.784	272.401.191.582.215

IL1RAP-mRNA	CACNG4-mRNA	CDKN2C-mRNA	NUMBL-mRNA
543.724.136.173.545	519.623.326.223.166	590.672.664.503.667	705.872.973.848.172
462.833.121.614.906	426.268.174.383.238	591.097.017.992.237	722.402.012.346.328
596.784.625.265.135	457.552.882.987.259	503.496.044.850.989	826.202.935.705.581
603.685.010.490.065	326.074.611.682.749	620.560.456.263.502	727.420.837.663.405
492.946.063.336.331	251.442.313.408.447	562.990.035.150.441	728.113.607.164.473
519.613.138.300.418	272.039.795.203.778	609.543.738.338.471	768.618.223.669.987
585.493.711.812.667	349.236.703.874.197	638.545.183.482.545	737.151.264.386.758
614.531.480.380.425	552.382.642.705.798	440.834.920.963.804	707.131.422.236.047
565.412.107.071.889	209.340.611.624.442	6.341.333.629.688	733.753.205.952.815
564.179.422.815.749	270.890.842.401.603	654.409.755.427.036	677.303.876.143.574
618.664.240.649.419	444.967.681.232.799	486.471.431.160.683	725.703.173.438.559
463.453.602.588.636	153.500.035.233.544	595.646.412.077.372	713.278.689.341.418

IL1RAP-mRNA	CACNG4-mRNA	CDKN2C-mRNA	NUMBL-mRNA
433.284.091.318.159	366.625.000.346.134	599.931.818.748.219	133.318.181.944.049
247.324.150.429.218	191.953.071.974.915	601.699.052.536.753	149.501.911.826.617
625.893.918.224.285	238.435.778.371.156	32.784.919.526.034	306.986.064.652.863
656.557.793.533.692	95.847.853.070.612	738.028.468.643.712	154.794.282.709.038
304.730.211.496.969	571.369.146.556.817	495.186.593.682.575	155.539.378.784.911
366.599.111.192.882	659.054.581.919.788	68.376.912.874.178	205.954.556.849.934
578.777.574.262.385	112.540.083.884.353	836.012.051.712.334	165.594.694.858.405
70.782.205.184.748	460.084.333.700.862	212.346.615.554.244	134.486.189.851.021
50.357.022.277.933	426.754.426.084.178	810.833.409.559.939	161.739.927.485.904
49.928.588.899.989	653.826.759.404.617	933.189.102.059.317	1.093.673.852.095
728.391.613.494.429	218.517.484.048.329	291.356.645.397.772	15.296.223.883.383
248.390.144.299.892	289.788.501.683.208	62.097.536.074.973	140.340.431.529.439

TPO-mRNA	NOS3-mRNA	COL11A2-mRNA	TNFAIP3-mRNA
554.415.656.565.196	664.369.223.920.288	941.628.174.309.981	766.753.898.115.724
320.609.821.546.601	502.167.364.432.859	980.303.335.785.324	596.312.146.197.347
316.049.133.059.375	603.496.044.850.989	791.537.883.275.721	703.496.044.850.989
252.378.052.266.128	639.824.964.057.742	105.415.168.869.581	732.252.231.441.417
34.319.609.738.925	666.642.622.752.952	86.505.598.220.705	812.257.526.217.751
280.786.079.328.812	673.720.623.972.434	775.931.694.133.008	848.858.227.681.471
268.501.211.668.436	611.127.687.138.646	973.213.602.879.839	865.079.640.134.645
552.382.642.705.798	482.338.670.891.689	773.027.730.452.541	112.070.910.013.909
267.836.861.696.557	512.903.002.597.514	667.836.861.696.557	867.836.861.696.557
377.303.876.143.574	636.841.787.810.224	562.451.623.672.566	828.839.579.467.103
518.664.240.649.419	618.664.240.649.419	76.196.018.137.703	886.471.431.160.683
2.312.607.930.999	557.313.548.122.221	726.292.080.689.864	713.703.636.641.554

TPO-mRNA	NOS3-mRNA	COL11A2-mRNA	TNFAIP3-mRNA
466.613.636.804.171	999.886.364.580.366	683.255.682.463.251	203.310.227.464.674
922.851.307.571.707	324.843.660.265.241	893.320.065.729.413	623.847.483.918.474
894.134.168.891.835	655.698.390.520.679	241.416.225.600.795	131.139.678.104.136
575.087.118.423.672	843.461.107.021.386	149.043.411.524.802	160.065.914.627.922
10.792.528.323.851	101.576.737.165.656	401.862.966.411.628	27.870.117.259.827
700.245.493.289.774	106.684.460.448.266	21.666.419.380.613	359.184.747.146.284
643.086.193.624.873	691.317.658.146.738	850.481.491.068.894	401.928.871.015.545
460.084.333.700.862	283.128.820.738.992	212.346.615.554.244	236.412.565.317.058
640.131.639.126.267	349.938.629.389.026	102.421.062.260.203	409.684.249.040.811
136.709.231.511.874	826.199.268.702.198	493.342.009.368.938	312.648.068.588.026
364.195.806.747.215	728.391.613.494.429	196.665.735.643.496	466.170.632.636.435
496.780.288.599.784	476.081.109.908.127	1.535.879.058.921	140.754.415.103.272

JAK3-mRNA	MAP3K13-mRNA	BIRC7-mRNA	FGF9-mRNA
58.242.644.848.447	619.623.326.223.166	473.680.164.359.436	643.724.136.173.545
630.370.901.209.244	51.769.518.698.065	331.712.952.785.476	444.488.507.505.313
596.784.625.265.135	603.496.044.850.989	389.745.692.475.995	489.745.692.475.995
668.701.087.152.958	650.867.363.027.107	318.674.553.538.371	398.321.214.129.858
690.674.055.686.323	594.441.097.482.928	304.493.785.078.325	392.946.063.336.331
741.736.547.827.207	601.501.870.092.941	217.982.957.067.508	357.837.894.716.535
702.486.211.956.899	625.486.772.501.531	300.694.021.157.172	438.545.183.482.545
463.074.163.097.449	482.338.670.891.689	528.281.832.755.419	552.382.642.705.798
578.270.527.678.031	623.908.357.144.005	177.147.802.135.705	542.968.950.410.885
557.140.490.026.609	522.675.672.887.865	183.443.930.609.989	474.132.990.170.841
667.206.923.366.443	544.967.681.232.799	286.471.431.160.683	567.206.923.366.443
520.337.886.124.424	518.707.704.891.514	20.495.735.251.652	489.757.043.172.015

JAK3-mRNA	MAP3K13-mRNA	BIRC7-mRNA	FGF9-mRNA
566.602.273.262.207	733.250.000.692.268	266.636.363.888.098	866.568.182.636.317
789.960.719.281.382	361.757.712.568.109	996.679.412.177.444	217.792.908.586.923
625.893.918.224.285	655.698.390.520.679	149.022.361.481.972	298.044.722.963.945
103.036.442.050.908	910.554.604.170.814	910.554.604.170.814	15.814.895.756.651
119.987.520.776.932	615.808.969.066.792	825.310.989.470.958	152.365.105.748.484
170.942.282.185.445	646.697.308.508.792	453.100.025.069.854	119.453.642.972.962
130.224.954.209.037	763.664.854.929.536	803.857.742.031.091	209.003.012.928.084
247.737.718.146.618	283.128.820.738.992	389.302.128.516.114	460.084.333.700.862
55.051.320.964.859	755.355.334.168.996	341.403.540.867.343	43.102.197.034.502
475.510.370.476.085	374.464.416.749.917	356.632.777.857.064	267.474.583.392.798
10.197.482.588.922	437.034.968.096.658	72.839.161.349.443	509.874.129.446.101
368.445.380.711.507	364.305.544.973.175	413.983.573.833.154	298.068.173.159.871

SGK2-mRNA	GADD45A-mRNA	SMAD9-mRNA	AXIN2-mRNA
490.672.664.503.667	612.911.906.637.312	58.242.644.848.447	649.168.914.575.783
364.970.486.694.163	597.163.296.182.899	735.014.458.508.272	673.716.770.819.197
316.049.133.059.375	721.938.501.964.731	616.049.133.059.375	752.972.514.025.947
406.810.103.888.509	646.237.997.799.714	698.321.214.129.858	777.170.803.610.486
434.449.813.264.216	689.139.259.252.979	636.686.594.567.061	731.604.168.659.293
352.775.287.409.538	719.613.138.300.418	587.014.507.154.246	670.339.152.673.209
407.732.953.946.312	716.882.789.394.862	641.293.257.124.756	811.964.034.432.109
582.338.670.891.689	865.627.672.308.163	482.338.670.891.689	668.136.770.404.446
294.140.302.279.937	731.450.984.161.229	620.610.624.899.378	76.723.448.293.378
357.140.490.026.609	727.738.280.194.862	732.093.916.793.535	765.461.826.851.508
486.471.431.160.683	586.471.431.160.683	667.206.923.366.443	632.414.593.024.413
297.557.294.372.143	84.315.490.037.225	6.312.607.930.999	679.373.462.073.561

SGK2-mRNA	GADD45A-mRNA	SMAD9-mRNA	AXIN2-mRNA
29.996.590.937.411	699.920.455.206.256	566.602.273.262.207	899.897.728.122.329
125.507.777.829.752	627.538.889.148.761	163.160.111.178.678	106.681.611.155.289
894.134.168.891.835	149.022.361.481.972	715.307.335.113.468	184.787.728.237.646
167.733.742.873.571	881.800.248.249.631	126.519.166.053.208	218.533.105.000.995
203.153.474.331.313	118.717.811.562.361	825.310.989.470.958	159.348.506.428.623
115.334.551.835.963	146.639.644.477.153	584.910.941.453.812	104.213.005.766.066
168.810.125.826.529	143.890.535.823.565	852.089.206.552.956	278.134.778.742.758
566.257.641.477.984	403.458.569.553.063	283.128.820.738.992	102.634.197.517.885
768.157.966.951.521	159.179.400.929.398	738.285.157.125.628	203.988.615.668.237
118.877.592.619.021	155.135.258.367.823	159.890.362.072.584	201.497.519.489.241
291.356.645.397.772	582.713.290.795.544	10.197.482.588.922	801.230.774.843.872
786.568.790.282.992	34.526.230.057.685	794.848.461.759.655	110.947.597.787.285

CEBPA-mRNA	PRKCA-mRNA	COL1A1-mRNA	DNMT3A-mRNA
564.369.223.920.288	590.672.664.503.667	165.154.948.268.182	664.369.223.920.288
467.076.648.246.946	714.344.260.761.625	172.554.464.985.422	713.209.763.402.224
457.552.882.987.259	633.041.633.203.606	169.066.907.101.175	735.688.854.339.725
506.810.103.888.509	703.685.010.490.065	169.888.313.745.742	702.096.706.329.399
486.806.008.869.917	512.585.784.616.682	15.205.391.114.981	714.539.803.256.246
372.039.795.203.778	595.880.269.136.286	152.670.814.409.256	690.527.329.494.606
393.293.963.012.795	666.229.204.018.428	159.337.921.225.016	654.299.311.181.193
582.338.670.891.689	591.084.955.016.723	137.459.711.116.306	563.074.163.097.449
409.340.611.624.442	570.221.535.891.994	121.301.292.222.499	653.302.925.380.153
470.890.842.401.603	652.560.121.065.297	133.910.251.889.661	674.932.269.246.194
486.471.431.160.683	618.664.240.649.419	118.870.821.246.353	644.967.681.232.799
453.500.035.233.544	697.081.941.375.079	145.516.594.291.511	611.134.972.275.189

CEBPA-mRNA	PRKCA-mRNA	COL1A1-mRNA	DNMT3A-mRNA
499.943.182.290.183	599.931.818.748.219	93.682.686.452.083	999.886.364.580.366
254.706.960.889.791	141.380.820.319.986	156.461.318.107.276	1.402.733.987.509
238.435.778.371.156	804.720.752.002.651	122.862.976.147.427	16.392.459.763.017
335.467.485.747.142	131.311.558.706.739	130.061.223.463.432	129.873.840.910.679
292.033.119.351.262	349.170.034.006.944	377.814.673.887.659	141.572.577.424.634
131.810.916.383.958	621.982.761.686.799	394.320.594.544.883	119.865.552.086.661
152.732.970.985.907	101.286.075.495.917	625.964.023.719.612	932.474.980.756.065
566.257.641.477.984	601.648.744.070.358	137.388.260.263.596	495.475.436.293.236
170.701.770.433.671	520.640.399.822.698	448.262.849.158.821	926.057.104.602.667
261.530.703.761.847	921.301.342.797.415	107.423.736.570.179	107.584.221.320.214
291.356.645.397.772	728.391.613.494.429	378.763.639.017.103	874.069.936.193.316
231.830.801.346.566	125.437.022.871.446	240.151.871.180.612	691.352.568.301.367

GAS1-mRNA	LAMC2-mRNA	PDGFB-mRNA	MPL-mRNA
626.036.359.965.137	454.415.656.565.196	564.369.223.920.288	405.872.973.848.172
799.478.392.607.955	388.417.012.057.865	439.513.203.985.603	246.913.262.129.981
670.481.184.681.756	316.049.133.059.375	686.093.104.873.484	257.552.882.987.259
664.617.715.402.101	541.455.145.290.652	518.674.553.538.371	210.874.302.338.244
758.767.211.611.511	501.692.347.461.365	603.099.865.982.538	234.449.813.264.216
837.682.281.531.556	317.982.957.067.508	474.276.576.506.624	26.272.885.476.463
93.556.683.658.028	368.501.211.668.436	414.444.373.532.166	168.501.211.668.436
942.329.955.110.402	382.338.670.891.689	652.382.642.705.798	440.834.920.963.804
74.117.229.575.794	623.090.963.999.435	444.390.336.332.855	0.771478021357053
794.991.652.351.982	474.132.990.170.841	53.787.598.223.237	157.140.490.026.609
656.515.402.974.792	567.206.923.366.443	486.471.431.160.683	544.967.681.232.799
866.281.047.988.154	20.495.735.251.652	425.120.738.633.485	0.72764543027784

GAS1-mRNA	LAMC2-mRNA	PDGFB-mRNA	MPL-mRNA
766.579.546.178.281	233.306.818.402.085	499.943.182.290.183	166.647.727.430.061
25.507.610.141.282	147.656.209.211.473	210.410.098.126.349	553.710.784.543.025
104.315.653.037.381	894.134.168.891.835	116.237.441.955.939	59.608.944.592.789
10.016.100.645.879	426.522.946.164.223	364.221.841.668.326	431.315.338.817.754
192.360.946.007.462	323.775.849.715.529	653.900.245.503.913	507.883.685.828.282
332.410.654.755.793	906.200.050.139.708	267.740.923.904.914	617.863.670.549.801
65.514.405.975.534	128.617.238.724.975	17.684.870.324.684	321.543.096.812.436
686.587.390.292.056	141.564.410.369.496	920.168.667.401.724	212.346.615.554.244
170.275.016.007.587	751.087.789.908.154	217.644.757.302.931	170.701.770.433.671
247.265.392.647.564	267.474.583.392.798	416.071.574.166.574	297.193.981.547.553
946.909.097.542.759	509.874.129.446.101	291.356.645.397.772	437.034.968.096.658
405.289.918.782.658	413.983.573.833.154	190.432.443.963.251	165.593.429.533.262

PRKACB-mRNA	NF1-mRNA	GATA3-mRNA	MTOR-mRNA
373.680.164.359.436	716.306.639.829.646	490.672.664.503.667	649.168.914.575.783
566.027.410.865.181	75.076.858.620.692	114.720.452.641.244	632.379.325.813.577
47.454.538.313.149	733.041.633.203.606	357.552.882.987.259	657.552.882.987.259
496.118.583.496.858	832.468.042.258.158	193.881.802.194.012	683.363.578.524.807
486.806.008.869.917	829.286.536.422.683	166.642.622.752.952	684.434.401.972.536
511.271.537.481.654	838.039.384.446.776	130.536.045.275.894	702.417.870.021.488
37.724.749.579.347	802.486.211.956.899	200.694.021.157.172	691.383.080.718.024
282.338.670.891.689	710.878.892.777.914	49.933.117.103.592	646.724.289.869.161
511.132.802.424.168	823.704.442.616.645	157.883.294.341.466	676.016.270.812.922
529.387.092.473.718	811.056.371.137.413	0.249476805378731	665.035.624.166.092
486.471.431.160.683	732.414.593.024.413	444.967.681.232.799	686.471.431.160.683
541.414.595.746.106	756.999.577.369.165	153.500.035.233.544	615.391.018.497.994

PRKACB-mRNA	NF1-mRNA	GATA3-mRNA	MTOR-mRNA
133.318.181.944.049	143.317.045.589.852	29.996.590.937.411	899.897.728.122.329
505.722.516.549.296	181.986.277.853.141	22.148.431.381.721	801.034.934.972.242
26.824.025.066.755	16.094.415.040.053	119.217.889.185.578	953.743.113.484.624
311.505.522.479.489	320.611.068.521.197	383.391.412.282.448	114.058.945.154.028
292.033.119.351.262	313.618.175.998.964	317.427.303.642.676	114.908.683.918.649
346.003.655.507.889	333.234.472.983.192	24.714.546.821.992	130.163.279.929.158
136.655.816.145.285	260.449.908.418.074	401.928.871.015.545	120.578.661.304.664
70.782.205.184.748	138.025.300.110.259	318.519.923.331.366	88.477.756.480.935
345.671.085.128.184	301.715.379.241.514	298.728.098.258.925	108.395.624.225.381
39.229.605.564.277	276.390.402.839.225	118.877.592.619.021	100.451.565.763.073
291.356.645.397.772	160.246.154.968.774	218.517.484.048.329	116.542.658.159.109
426.403.081.048.148	190.018.460.389.417	289.788.501.683.208	712.051.746.993.024

RHOA-mRNA	FUBP1-mRNA	WHSC1-mRNA	TNFRSF10B-mRNA
769.099.795.398.123	664.369.223.920.288	649.168.914.575.783	564.369.223.920.288
955.376.387.176.698	739.829.238.074.936	600.518.552.154.002	701.756.924.599.585
764.161.802.033.036	793.308.083.449.067	582.345.634.331.618	689.745.692.475.995
93.279.115.438.446	743.467.304.882.729	647.019.948.245.643	696.118.583.496.858
800.270.961.539.395	77.496.395.957.785	62.393.158.959.501	74.319.609.738.925
795.401.762.879.748	760.304.100.139.962	609.543.738.338.471	724.787.495.809.818
917.285.215.050.741	74.987.933.079.014	625.486.772.501.531	735.743.745.865.586
810.878.892.777.914	695.266.972.586.185	49.933.117.103.592	74.380.965.530.321
863.256.492.735.245	742.611.404.988.502	597.115.036.619.342	626.333.111.768.673
915.636.740.098.725	734.223.394.629.858	578.863.561.648.676	649.740.431.882.232
711.264.182.505.042	567.206.923.366.443	632.414.593.024.413	603.463.931.304.914
956.053.544.444.258	698.974.027.564.802	568.184.174.066.471	634.969.724.973.422

RHOA-mRNA	FUBP1-mRNA	WHSC1-mRNA	TNFRSF10B-mRNA
206.643.182.013.276	999.886.364.580.366	899.897.728.122.329	499.943.182.290.183
751.570.104.886.399	168.697.219.024.108	642.304.510.069.908	129.568.323.583.068
199.689.964.385.843	244.396.672.830.435	566.284.973.631.495	119.217.889.185.578
642.659.854.838.453	173.005.374.792.455	886.592.640.903.161	124.602.208.991.796
256.481.261.343.282	215.215.711.869.734	755.476.982.669.569	172.680.453.181.616
24.796.928.644.732	194.421.101.666.337	68.376.912.874.178	151.994.462.955.251
577.169.858.778.324	180.867.991.956.995	763.664.854.929.536	163.986.979.374.342
276.050.600.220.517	123.868.859.073.309	318.519.923.331.366	173.416.402.702.633
396.881.616.258.286	171.982.033.711.924	627.329.006.343.742	768.157.966.951.521
570.612.444.571.302	162.267.913.924.964	552.780.805.678.449	903.469.703.904.561
138.394.406.563.942	509.874.129.446.101	801.230.774.843.872	655.552.452.144.987
755.106.038.671.673	127.092.957.166.778	513.339.631.553.111	815.547.640.451.313

**RPA3-mRNA**	**JUN-mRNA**	**NFKBIA-mRNA**	**BAX-mRNA**
454.415.656.565.196	764.369.223.920.288	775.916.945.662.281	694.625.500.922.331
27.321.670.271.336	653.378.557.964.206	921.598.280.439.786	807.201.703.001.823
357.552.882.987.259	638.288.375.193.019	80.990.907.859.296	759.789.664.290.104
274.617.294.399.773	807.580.913.402.035	85.608.698.413.965	841.859.828.596.922
315.185.305.469.976	768.879.404.055.797	823.324.138.154.041	836.130.642.032.871
104.232.604.692.514	747.528.545.420.125	973.232.401.834.459	850.830.051.142.921
249.236.703.874.197	745.319.644.146.129	95.003.954.124.979	826.621.269.860.932
463.074.163.097.449	986.778.082.827.534	124.111.642.252.451	69.933.117.103.592
277.147.802.135.705	774.014.481.455.226	10.059.767.363.538	700.988.276.068.213
157.140.490.026.609	888.610.142.592.238	101.121.141.629.375	755.325.755.355.583
386.471.431.160.683	76.196.018.137.703	790.910.843.096.528	718.664.240.649.419
172.764.543.027.784	730.129.261.777.116	100.234.143.646.983	728.988.785.449.891

**RPA3-mRNA**	**JUN-mRNA**	**NFKBIA-mRNA**	**BAX-mRNA**
233.306.818.402.085	199.977.272.916.073	216.642.045.659.079	123.319.318.298.245
664.452.941.451.629	926.542.712.801.995	594.685.382.599.208	26.910.344.128.791
119.217.889.185.578	834.525.224.299.046	27.420.114.512.683	193.729.069.926.564
670.934.971.494.284	269.811.706.393.773	377.640.541.098.211	342.176.835.462.084
888.796.450.199.493	206.327.747.367.739	300.921.083.853.257	328.854.686.573.813
205.954.556.849.934	177.944.737.118.343	850.592.319.790.226	364.127.656.510.683
562.700.419.421.764	175.240.987.762.778	724.275.825.570.012	307.877.515.197.908
247.737.718.146.618	934.325.108.438.675	544.669.068.896.636	127.407.969.332.546
682.807.081.734.685	213.803.967.468.173	106.731.281.963.653	128.879.836.677.422
297.193.981.547.553	473.132.818.623.705	110.675.038.728.309	187.826.596.338.054
145.678.322.698.886	196.665.735.643.496	240.369.232.453.162	145.678.322.698.886
331.186.859.066.523	157.727.741.630.432	104.075.470.461.655	156.485.790.908.932

CDH1-mRNA	FGFR4-mRNA	WNT11-mRNA	MCM5-mRNA
373.680.164.359.436	454.415.656.565.196	405.872.973.848.172	432.176.414.431.551
214.720.452.641.244	246.913.262.129.981	331.712.952.785.476	429.016.248.025.449
316.049.133.059.375	489.745.692.475.995	357.552.882.987.259	503.496.044.850.989
655.352.786.605.533	252.378.052.266.128	512.864.258.082.014	4.893.014.332.327
534.449.813.264.216	280.392.975.127.945	451.442.313.408.447	483.635.122.897.183
230.536.045.275.894	172.039.795.203.778	172.039.795.203.778	512.978.888.817.548
314.444.373.532.166	314.444.373.532.166	432.886.830.645.909	468.501.211.668.436
340.834.920.963.804	607.131.422.236.047	182.338.670.891.689	514.531.480.380.425
760.436.803.552.179	377.147.802.135.705	223.090.963.999.435	479.384.583.438.551
670.068.791.721.106	324.947.680.537.873	529.387.092.473.718	483.443.930.609.989
567.206.923.366.443	286.471.431.160.683	286.471.431.160.683	486.471.431.160.683
401.304.764.914.009	20.495.735.251.652	242.808.514.841.893	468.184.174.066.471

CDH1-mRNA	FGFR4-mRNA	WNT11-mRNA	MCM5-mRNA
133.318.181.944.049	233.306.818.402.085	166.647.727.430.061	199.977.272.916.073
44.296.862.763.442	553.710.784.543.025	996.679.412.177.444	195.644.477.205.202
894.134.168.891.835	298.044.722.963.945	119.217.889.185.578	32.784.919.526.034
939.308.960.091.998	575.087.118.423.672	349.844.663.707.734	297.128.344.518.897
406.306.948.662.625	698.340.068.013.887	228.547.658.622.727	285.684.573.278.408
494.290.936.439.841	329.527.290.959.894	329.527.290.959.894	350.122.746.644.887
8.842.435.162.342	8.842.435.162.342	200.964.435.507.773	257.234.477.449.949
106.173.307.777.122	672.430.949.255.106	35.391.102.592.374	35.391.102.592.374
194.600.018.294.385	136.561.416.346.937	469.429.868.692.596	277.390.376.954.716
104.017.893.541.644	95.102.074.095.217	39.229.605.564.277	285.306.222.285.651
509.874.129.446.101	72.839.161.349.443	72.839.161.349.443	291.356.645.397.772
16.145.359.379.493	413.983.573.833.154	5.381.786.459.831	256.669.815.776.555

DTX3-mRNA	PLA2G4E-mRNA	IL13RA2-mRNA	SKP1-mRNA
605.872.973.848.172	432.176.414.431.551	554.415.656.565.196	794.625.500.922.331
646.913.262.129.981	188.417.012.057.865	236.959.694.774.889	950.475.653.103.053
733.041.633.203.606	257.552.882.987.259	47.454.538.313.149	755.280.875.337.251
657.544.264.248.377	126.074.611.682.749	274.617.294.399.773	975.740.019.942.098
662.062.253.791.639	215.185.305.469.976	0.344498132642157	889.908.698.431.979
665.703.589.104.035	172.039.795.203.778	152.775.287.409.539	878.109.388.372.534
737.849.907.418.369	0.685012116684361	200.694.021.157.172	848.591.201.660.467
528.281.832.755.419	552.382.642.705.798	282.338.670.891.689	924.123.922.380.279
6.610.681.809.454	223.090.963.999.435	0.356440522078209	95.053.407.410.356
655.325.755.355.583	224.947.680.537.873	157.140.490.026.609	98.521.756.703.967
518.664.240.649.419	444.967.681.232.799	518.664.240.649.419	750.857.050.138.155
640.712.552.978.329	10.495.735.251.652	189.757.043.172.015	930.034.565.676.513

DTX3-mRNA	PLA2G4E-mRNA	IL13RA2-mRNA	SKP1-mRNA
666.590.909.720.244	199.977.272.916.073	466.613.636.804.171	24.663.863.659.649
885.937.255.268.839	369.140.523.028.683	516.796.732.240.156	726.468.549.320.448
16.094.415.040.053	59.608.944.592.789	26.824.025.066.755	187.768.175.467.285
953.686.138.052.589	23.961.963.267.653	670.934.971.494.284	865.506.113.227.627
984.024.641.292.296	444.398.225.099.746	12.697.092.145.707	477.410.664.678.585
100.917.732.856.468	329.527.290.959.894	288.336.379.589.907	439.918.933.431.459
166.398.552.600.436	160.771.548.406.218	401.928.871.015.545	358.520.552.945.866
389.302.128.516.114	460.084.333.700.862	70.782.205.184.748	605.187.854.329.596
977.267.635.732.768	469.429.868.692.596	128.026.327.825.253	726.762.787.621.356
939.132.981.690.268	475.510.370.476.085	297.193.981.547.553	924.273.282.612.891
364.195.806.747.215	218.517.484.048.329	364.195.806.747.215	182.097.903.373.607
848.666.326.357.965	206.991.786.916.577	372.585.216.449.838	630.496.982.947.893

FZD10-mRNA	DDB2-mRNA	PLA1A-mRNA	RAC2-mRNA
373.680.164.359.436	598.472.915.703.794	373.680.164.359.436	673.680.164.359.436
246.913.262.129.981	630.370.901.209.244	188.417.012.057.865	667.076.648.246.946
257.552.882.987.259	743.350.982.500.016	316.049.133.059.375	416.049.133.059.375
326.074.611.682.749	65.312.750.592.082	584.570.861.754.864	610.874.302.338.244
351.442.313.408.447	745.824.029.869.135	538.889.225.200.061	512.585.784.616.682
352.775.287.409.538	743.120.438.573.713	436.425.414.181.251	427.498.680.371.542
285.493.711.812.667	705.133.433.093.018	568.501.211.668.436	507.732.953.946.312
463.074.163.097.449	382.338.670.891.689	440.834.920.963.804	621.570.413.169.565
235.644.052.207.821	64.509.581.208.625	592.122.514.086.174	483.756.721.181.483
389.333.299.515.346	659.820.495.960.981	60.043.643.075.422	483.443.930.609.989
386.471.431.160.683	586.471.431.160.683	586.471.431.160.683	644.967.681.232.799
253.500.035.233.544	558.562.642.540.541	440.007.077.224.934	632.755.827.246.497

FZD10-mRNA	DDB2-mRNA	PLA1A-mRNA	RAC2-mRNA
133.318.181.944.049	633.261.364.234.232	133.318.181.944.049	106.654.545.555.239
553.710.784.543.025	789.960.719.281.382	369.140.523.028.683	101.882.784.355.916
59.608.944.592.789	172.865.939.319.088	894.134.168.891.835	178.826.833.778.367
95.847.853.070.612	924.931.782.131.406	575.087.118.423.672	690.104.542.108.406
114.273.829.311.363	175.854.726.218.043	419.004.040.808.332	349.170.034.006.944
115.334.551.835.963	172.589.918.640.244	205.954.556.849.934	193.597.283.438.938
723.471.967.827.982	13.263.652.743.513	514.468.954.899.898	337.620.251.653.058
247.737.718.146.618	141.564.410.369.496	212.346.615.554.244	743.213.154.439.854
512.105.311.301.014	874.846.573.472.566	605.991.285.039.533	285.925.465.476.399
148.596.990.773.777	968.852.379.845.023	641.939.000.142.715	285.306.222.285.651
145.678.322.698.886	582.713.290.795.544	582.713.290.795.544	874.069.936.193.316
579.577.003.366.415	480.220.945.646.458	211.131.622.654.908	803.128.133.236.318

EGF-mRNA	VHL-mRNA	FGF18-mRNA	POLB-mRNA
454.415.656.565.196	698.472.915.703.794	405.872.973.848.172	473.680.164.359.436
246.913.262.129.981	760.116.101.498.359	288.417.012.057.865	531.712.952.785.476
316.049.133.059.375	745.817.187.923.443	416.049.133.059.375	438.288.375.193.019
274.617.294.399.773	749.723.873.520.879	333.113.544.471.888	539.824.964.057.742
251.442.313.408.447	703.099.865.982.538	280.392.975.127.945	551.442.313.408.447
26.272.885.476.463	714.246.271.821.059	36.272.885.476.463	581.843.003.499.831
249.236.703.874.197	725.486.772.501.531	314.444.373.532.166	504.256.412.130.245
573.027.730.452.541	721.570.413.169.565	514.531.480.380.425	340.834.920.963.804
194.140.302.279.937	713.780.023.560.287	309.340.611.624.442	548.572.353.902.318
294.991.652.351.982	713.211.985.474.057	283.443.930.609.989	569.242.030.122.746
386.471.431.160.683	703.463.931.304.914	286.471.431.160.683	603.463.931.304.914
0.312607930998996	775.278.499.255.635	218.707.704.891.514	487.739.254.978.252

EGF-mRNA	VHL-mRNA	FGF18-mRNA	POLB-mRNA
233.306.818.402.085	126.652.272.846.846	166.647.727.430.061	266.636.363.888.098
553.710.784.543.025	194.167.915.113.087	738.281.046.057.366	398.671.764.870.978
894.134.168.891.835	175.846.386.548.728	178.826.833.778.367	208.631.306.074.761
670.934.971.494.284	180.673.203.038.104	100.640.245.724.143	421.730.553.510.693
571.369.146.556.817	130.780.049.100.783	698.340.068.013.887	457.095.317.245.453
617.863.670.549.801	141.284.825.999.055	12.357.273.410.996	564.315.485.768.818
562.700.419.421.764	152.732.970.985.907	8.842.435.162.342	329.581.674.232.747
53.086.653.888.561	148.642.630.887.971	35.391.102.592.374	106.173.307.777.122
38.407.898.347.576	140.828.960.607.779	853.508.852.168.357	448.092.147.388.387
772.704.352.023.638	140.275.559.290.445	713.265.555.714.128	517.117.527.892.742
145.678.322.698.886	131.110.490.428.997	72.839.161.349.443	655.552.452.144.987
124.195.072.149.946	215.685.441.967.073	455.381.931.216.469	293.928.337.421.539

FUT8-mRNA	SHC4-mRNA	FZD8-mRNA	BAP1-mRNA
605.872.973.848.172	543.724.136.173.545	605.872.973.848.172	816.306.639.829.646
710.527.384.594.653	320.609.821.546.601	721.687.805.421.926	797.586.995.471.546
706.738.192.620.227	489.745.692.475.995	772.527.594.937.727	890.644.570.798.721
654.614.833.568.973	446.237.997.799.714	712.369.336.484.841	893.033.986.801.582
600.270.961.539.395	34.319.609.738.925	6.227.141.182.004	874.537.756.892.434
647.528.545.420.125	436.425.414.181.251	664.521.045.564.356	857.214.699.345.384
647.942.798.303.447	438.545.183.482.545	616.074.554.765.076	884.488.345.346.275
382.338.670.891.689	440.834.920.963.804	668.136.770.404.446	898.325.804.569.528
629.503.997.741.407	519.774.277.605.915	674.875.794.485.697	825.327.745.302.281
672.521.023.634.513	41.074.578.005.063	605.683.172.743.634	819.492.064.175.664
444.967.681.232.799	586.471.431.160.683	518.664.240.649.419	807.416.767.723.578
648.919.666.272.232	472.764.543.027.784	75.573.681.653.639	762.246.319.358.578

FUT8-mRNA	SHC4-mRNA	FZD8-mRNA	BAP1-mRNA
666.590.909.720.244	433.284.091.318.159	666.590.909.720.244	286.634.091.179.705
137.689.415.089.699	922.851.307.571.707	148.763.630.780.559	251.753.836.705.562
134.120.125.333.775	298.044.722.963.945	211.611.753.304.401	479.852.003.971.951
934.516.567.438.467	220.450.062.062.408	13.945.862.621.774	487.865.572.129.415
641.203.153.358.206	10.792.528.323.851	749.128.436.596.716	429.161.714.524.898
889.723.685.591.713	205.954.556.849.934	100.093.914.629.068	380.604.021.058.677
892.282.093.654.511	209.003.012.928.084	715.433.390.407.671	459.806.628.441.784
141.564.410.369.496	212.346.615.554.244	102.634.197.517.885	506.092.767.070.948
785.228.143.994.888	367.008.806.432.393	107.542.115.373.213	305.129.414.650.188
105.801.057.430.929	172.372.509.297.581	665.714.518.666.519	293.033.265.805.887
218.517.484.048.329	582.713.290.795.544	364.195.806.747.215	269.504.896.992.939
898.344.355.217.944	264.949.487.253.218	188.362.526.094.085	197.056.181.144.581

RFC4-mRNA	ZBTB32-mRNA	SIRT4-mRNA	MAP3K5-mRNA
490.672.664.503.667	405.872.973.848.172	590.672.664.503.667	532.176.414.431.551
513.209.763.402.224	202.167.364.432.859	391.979.403.030.937	495.455.944.847.005
548.241.942.548.111	357.552.882.987.259	489.745.692.475.995	566.299.167.112.293
418.674.553.538.371	193.881.802.194.012	410.874.302.338.244	575.899.698.435.531
483.635.122.897.183	215.185.305.469.976	425.138.872.825.068	541.058.732.309.993
509.543.738.338.471	204.232.604.692.514	367.459.426.242.466	589.032.295.348.009
426.997.461.740.552	226.997.461.740.552	400.694.021.157.172	566.229.204.018.428
414.531.480.380.425	382.338.670.891.689	591.084.955.016.723	440.834.920.963.804
432.606.687.303.469	109.340.611.624.442	316.379.544.413.581	592.122.514.086.174
453.487.902.424.098	0.834439306099887	294.991.652.351.982	574.132.990.170.841
518.664.240.649.419	444.967.681.232.799	586.471.431.160.683	518.664.240.649.419
397.557.294.372.143	-0.27235456972216	318.707.704.891.514	51.199.628.530.566

RFC4-mRNA	ZBTB32-mRNA	SIRT4-mRNA	MAP3K5-mRNA
29.996.590.937.411	166.647.727.430.061	599.931.818.748.219	399.954.545.832.146
350.683.496.877.249	406.054.575.331.551	15.134.761.444.176	310.078.039.344.094
447.067.084.445.918	119.217.889.185.578	298.044.722.963.945	506.676.029.038.706
182.110.920.834.163	383.391.412.282.448	172.526.135.527.102	541.540.369.848.958
285.684.573.278.408	444.398.225.099.746	190.456.382.185.606	425.352.586.881.186
34.188.456.437.089	411.909.113.699.867	127.691.825.246.959	593.149.123.727.809
192.925.858.087.462	482.314.645.218.654	160.771.548.406.218	506.430.377.479.587
17.695.551.296.187	141.564.410.369.496	601.648.744.070.358	212.346.615.554.244
200.574.580.259.564	213.377.213.042.089	896.184.294.776.775	605.991.285.039.533
231.811.305.607.092	178.316.388.928.532	772.704.352.023.638	534.949.166.785.596
364.195.806.747.215	218.517.484.048.329	582.713.290.795.544	364.195.806.747.215
157.313.758.056.598	0.827967147666308	910.763.862.432.938	347.746.202.019.849

PRKAR1B-mRNA	CEBPE-mRNA	CBL-mRNA	PDGFRB-mRNA
532.176.414.431.551	543.724.136.173.545	709.435.364.821.244	780.289.083.405.213
47.321.670.271.336	246.913.262.129.981	656.224.202.569.129	827.476.902.613.111
438.288.375.193.019	389.745.692.475.995	666.299.167.112.293	905.126.226.083.899
461.124.336.391.162	252.378.052.266.128	762.531.854.912.334	904.079.369.288.936
425.138.872.825.068	280.392.975.127.945	698.112.275.318.581	945.954.178.280.387
460.304.100.139.962	189.032.295.348.009	722.422.369.003.353	103.714.496.432.167
472.940.623.604.281	326.997.461.740.552	806.871.640.915.841	849.236.703.874.196
514.531.480.380.425	414.531.480.380.425	573.027.730.452.541	810.878.892.777.914
394.140.302.279.937	0.771478021357053	623.090.963.999.435	896.253.723.588.871
389.333.299.515.346	183.443.930.609.989	646.864.532.584.089	946.864.532.584.089
444.967.681.232.799	386.471.431.160.683	486.471.431.160.683	822.226.631.622.491
440.007.077.224.934	189.757.043.172.015	60.495.735.251.652	890.631.028.128.431

PRKAR1B-mRNA	CEBPE-mRNA	CBL-mRNA	PDGFRB-mRNA
399.954.545.832.146	433.284.091.318.159	13.665.113.649.265	223.307.954.756.282
265.781.176.580.652	553.710.784.543.025	944.999.738.953.429	309.708.898.821.065
208.631.306.074.761	149.022.361.481.972	101.335.205.807.741	530.519.606.875.822
244.412.025.330.061	575.087.118.423.672	197.446.577.325.461	526.683.952.623.014
190.456.382.185.606	698.340.068.013.887	126.336.066.849.785	704.053.759.479.455
243.026.377.082.922	370.718.202.329.881	149.523.008.273.052	132.469.970.965.877
26.527.305.487.026	964.629.290.437.309	268.488.485.838.384	360.128.268.429.928
35.391.102.592.374	17.695.551.296.187	53.086.653.888.561	276.050.600.220.517
153.631.593.390.304	170.701.770.433.671	751.087.789.908.154	498.875.924.092.404
148.596.990.773.777	356.632.777.857.064	885.638.065.011.708	708.510.452.009.367
218.517.484.048.329	145.678.322.698.886	291.356.645.397.772	298.640.561.532.716
211.131.622.654.908	372.585.216.449.838	662.373.718.133.046	479.806.962.072.626

TGFBR2-mRNA	DUSP4-mRNA	BMP4-mRNA	FANCC-mRNA
832.176.414.431.551	505.872.973.848.172	659.478.263.872.193	638.065.783.336.908
848.408.296.276.578	505.409.512.202.096	811.106.393.415.004	606.208.791.277.449
806.738.192.620.227	457.552.882.987.259	772.527.594.937.727	738.288.375.193.019
912.987.723.647.178	527.866.802.482.475	788.133.252.727.936	650.867.363.027.107
859.005.083.889.784	373.681.555.542.092	709.269.098.223.162	673.681.555.542.092
919.207.316.642.983	317.982.957.067.508	699.186.097.994.216	684.451.926.386.697
926.244.094.472.011	349.236.703.874.197	729.972.196.079.957	681.429.513.362.933
849.581.205.088.838	563.074.163.097.449	640.834.920.963.804	621.570.413.169.565
918.086.895.749.476	465.412.107.071.889	729.503.997.741.407	582.676.045.685.824
956.914.892.632.573	480.406.565.705.637	658.932.680.826.336	601.766.113.015.566
815.011.653.046.908	544.967.681.232.799	667.206.923.366.443	703.463.931.304.914
966.869.303.661.842	397.557.294.372.143	708.519.743.489.592	572.764.543.027.784

TGFBR2-mRNA	DUSP4-mRNA	BMP4-mRNA	FANCC-mRNA
319.963.636.665.717	333.295.454.860.122	966.556.819.094.353	833.238.637.150.305
358.066.307.337.823	332.226.470.725.815	276.486.251.748.483	668.144.346.681.917
268.240.250.667.551	238.435.778.371.156	211.611.753.304.401	166.905.044.859.809
560.230.701.197.727	388.183.804.935.979	235.785.718.553.706	910.554.604.170.814
385.356.746.622.209	133.319.467.529.924	136.493.740.566.351	106.655.574.023.939
584.910.941.453.811	906.200.050.139.708	127.279.916.133.259	114.922.642.722.263
614.147.314.911.754	112.540.083.884.353	157.556.117.438.094	112.540.083.884.353
360.989.246.442.214	495.475.436.293.236	849.386.462.216.976	743.213.154.439.854
580.386.019.474.483	251.785.111.389.665	157.045.628.798.978	567.583.386.691.957
759.627.816.835.547	2.793.623.426.547	962.908.500.214.072	647.882.879.773.666
284.072.729.262.828	437.034.968.096.658	10.197.482.588.922	131.110.490.428.997
813.891.706.155.981	157.313.758.056.598	135.786.612.217.274	529.898.974.506.437

ITGB3-mRNA	ITGA9-mRNA	COL4A4-mRNA	NUPR1-mRNA
590.672.664.503.667	68.242.644.848.447	505.872.973.848.172	519.623.326.223.166
863.370.438.824.791	51.915.986.457.709	295.455.944.847.005	729.016.248.025.449
627.596.854.801.368	652.972.514.025.947	489.745.692.475.995	596.784.625.265.135
821.958.879.207.073	577.170.803.610.486	374.617.294.399.773	676.536.650.923.104
651.442.313.408.447	61.773.881.468.069	304.493.785.078.325	626.336.136.991.675
625.177.941.255.409	729.025.356.036.873	330.536.045.275.894	533.510.779.615.299
672.940.623.604.281	732.163.673.722.801	359.190.271.229.288	585.493.711.812.667
591.084.955.016.723	621.570.413.169.565	382.338.670.891.689	607.131.422.236.047
62.871.778.596.411	613.780.023.560.287	235.644.052.207.821	574.875.794.485.697
574.132.990.170.841	732.629.240.242.956	341.940.180.682.104	614.429.456.868.668
544.967.681.232.799	644.967.681.232.799	486.471.431.160.683	444.967.681.232.799
782.040.257.119.769	703.142.617.845.494	3.312.607.930.999	610.268.486.162.476

ITGB3-mRNA	ITGA9-mRNA	COL4A4-mRNA	NUPR1-mRNA
599.931.818.748.219	113.320.454.652.441	333.295.454.860.122	366.625.000.346.134
397.195.202.778.863	365.449.117.798.396	775.195.098.360.235	156.515.581.764.162
774.916.279.706.257	923.938.641.188.229	298.044.722.963.945	625.893.918.224.285
298.086.823.049.604	546.332.762.502.488	134.186.994.298.857	108.787.313.235.145
914.190.634.490.907	723.734.252.305.301	825.310.989.470.958	768.174.074.815.276
762.031.860.344.754	15.652.546.320.595	988.581.872.879.682	40.367.093.142.587
106.109.221.948.104	159.967.690.664.187	120.578.661.304.664	578.777.574.262.385
601.648.744.070.358	743.213.154.439.854	141.564.410.369.496	672.430.949.255.106
780.960.599.734.046	704.144.803.038.894	512.105.311.301.014	537.710.576.866.065
534.949.166.785.596	160.484.750.035.679	106.989.833.357.119	707.321.676.083.177
437.034.968.096.658	874.069.936.193.316	291.356.645.397.772	218.517.484.048.329
226.035.031.312.902	130.818.809.331.277	993.560.577.199.569	687.212.732.563.035

COL3A1-mRNA	PRKCB-mRNA	CDC25B-mRNA	PTEN-mRNA
121.461.925.797.321	612.911.906.637.312	564.369.223.920.288	811.184.107.494.128
140.835.357.374.366	391.979.403.030.937	623.466.736.766.278	911.106.393.415.004
1.409.541.075.237	389.745.692.475.995	648.241.942.548.111	786.093.104.873.484
144.028.212.557.451	466.673.847.650.332	623.343.877.083.175	845.451.786.022.416
137.798.428.390.155	489.908.698.431.979	6.227.141.182.004	856.849.980.684.026
144.721.512.034.771	560.304.100.139.962	567.459.426.242.466	853.578.124.785.132
144.151.637.898.577	489.446.548.231.331	614.444.373.532.166	863.920.842.707.124
937.797.556.059.452	49.933.117.103.592	614.531.480.380.425	786.778.082.827.534
123.451.252.088.504	494.140.302.279.937	5.980.931.386.986	860.120.075.644.311
131.989.387.847.389	433.693.964.662.907	570.890.842.401.603	926.628.509.306.529
944.967.681.232.799	518.664.240.649.419	567.206.923.366.443	686.471.431.160.683
135.709.776.664.954	491.746.998.915.786	653.500.035.233.544	918.399.984.538.613

COL3A1-mRNA	PRKCB-mRNA	CDC25B-mRNA	PTEN-mRNA
453.281.818.609.766	699.920.455.206.256	499.943.182.290.183	276.635.227.533.901
17.360.678.798.039	15.134.761.444.176	753.046.666.978.513	552.972.503.496.967
175.041.665.796.725	149.022.361.481.972	894.134.168.891.835	232.474.883.911.877
21.661.135.554.693	253.996.810.637.122	752.405.646.604.304	35.080.314.223.844
14.065.203.824.407	298.381.665.424.116	749.128.436.596.716	379.643.055.156.641
227.274.972.575.039	486.052.754.165.844	510.767.300.987.835	37.113.011.144.358
21.847.245.712.921	297.427.364.551.504	70.739.481.298.736	398.713.440.047.421
665.352.728.736.631	318.519.923.331.366	70.782.205.184.748	233.581.277.109.668
52.029.899.628.183	307.263.186.780.608	631.596.550.604.584	388.346.527.736.602
940.321.757.616.458	202.091.907.452.336	523.061.407.523.694	61.578.592.976.653
699.255.948.954.653	364.195.806.747.215	509.874.129.446.101	116.542.658.159.109
121.694.611.363.994	302.208.008.898.202	927.323.205.386.264	581.646.921.235.581

**MAD2L2-mRNA**	**POLE2-mRNA**	**MAPK1-mRNA**	**ZIC2-mRNA**
605.872.973.848.172	454.415.656.565.196	626.036.359.965.137	664.369.223.920.288
687.512.498.097.564	381.016.953.913.487	657.905.031.337.784	364.970.486.694.163
678.498.219.550.154	357.552.882.987.259	603.496.044.850.989	60.990.907.859.296
687.545.596.094.269	358.267.421.171.485	68.871.852.535.248	463.925.774.008.121
656.366.665.310.432	366.642.622.752.952	700.270.961.539.395	547.378.114.958.712
636.425.414.181.251	324.395.990.809.479	706.912.610.626.886	492.985.131.766.673
649.236.703.874.196	349.236.703.874.197	600.694.021.157.172	349.236.703.874.197
682.338.670.891.689	463.074.163.097.449	718.093.871.353.497	73.469.486.649.739
538.618.786.547.226	294.140.302.279.937	700.988.276.068.213	449.939.847.592.025
572.521.023.634.513	257.140.490.026.609	70.043.643.075.422	53.787.598.223.237
632.414.593.024.413	518.664.240.649.419	703.463.931.304.914	567.206.923.366.443
567.015.993.561.708	281.510.827.152.818	646.235.505.050.368	153.500.035.233.544

**MAD2L2-mRNA**	**POLE2-mRNA**	**MAPK1-mRNA**	**ZIC2-mRNA**
666.590.909.720.244	233.306.818.402.085	766.579.546.178.281	999.886.364.580.366
117.386.686.323.121	1.402.733.987.509	95.607.395.464.429	125.507.777.829.752
11.027.654.749.666	119.217.889.185.578	655.698.390.520.679	685.502.862.817.074
1.174.136.200.115	119.809.816.338.265	118.372.098.542.206	249.204.417.983.591
945.933.364.855.174	12.697.092.145.707	128.240.630.671.641	444.398.225.099.746
823.818.227.399.734	947.390.961.509.695	134.282.371.066.157	304.812.744.137.902
900.320.671.074.821	112.540.083.884.353	643.086.193.624.873	112.540.083.884.353
113.251.528.295.597	247.737.718.146.618	145.103.520.628.733	16.279.907.192.492
418.219.337.562.495	768.157.966.951.521	128.879.836.677.422	226.179.845.824.614
529.005.287.154.645	594.387.963.095.106	128.387.800.028.543	416.071.574.166.574
801.230.774.843.872	364.195.806.747.215	131.110.490.428.997	509.874.129.446.101
509.199.795.814.779	703.772.075.516.361	881.785.012.264.618	289.788.501.683.208

MLLT4-mRNA	NTF3-mRNA	CBLC-mRNA	NFATC1-mRNA
794.625.500.922.331	373.680.164.359.436	332.176.414.431.551	605.872.973.848.172
681.016.953.913.487	295.455.944.847.005	188.417.012.057.865	613.967.085.372.704
800.179.358.457.469	357.552.882.987.259	316.049.133.059.375	643.350.982.500.016
88.591.708.773.552	252.378.052.266.128	369.370.552.410.359	61.772.227.612.652
851.442.313.408.447	334.449.813.264.216	351.442.313.408.447	618.998.818.358.653
824.787.495.809.818	242.083.767.017.887	172.039.795.203.778	634.244.977.149.416
847.617.500.523.938	368.501.211.668.436	168.501.211.668.436	568.501.211.668.436
791.084.955.016.723	463.074.163.097.449	282.338.670.891.689	663.074.163.097.449
883.487.310.264.556	329.503.997.741.407	357.883.294.341.466	552.636.552.352.052
80.855.271.604.368	429.387.092.473.718	241.940.180.682.104	515.636.740.098.725
795.217.715.285.717	386.471.431.160.683	386.471.431.160.683	567.206.923.366.443
734.235.527.439.305	297.557.294.372.143	1.312.607.930.999	520.337.886.124.424

MLLT4-mRNA	NTF3-mRNA	CBLC-mRNA	NFATC1-mRNA
24.663.863.659.649	133.318.181.944.049	999.886.364.580.366	666.590.909.720.244
11.221.871.900.072	775.195.098.360.235	369.140.523.028.683	705.058.398.984.785
256.318.461.748.993	119.217.889.185.578	894.134.168.891.835	86.432.969.659.544
464.382.848.127.115	575.087.118.423.672	129.394.601.645.326	72.365.129.068.312
365.676.253.796.363	101.576.737.165.656	114.273.829.311.363	730.082.798.378.155
303.988.925.910.502	535.481.847.809.827	329.527.290.959.894	811.460.953.988.739
356.108.979.719.773	128.617.238.724.975	321.543.096.812.436	514.468.954.899.898
240.659.497.628.143	247.737.718.146.618	70.782.205.184.748	990.950.872.586.472
456.627.235.910.071	98.153.517.999.361	11.949.123.930.357	460.894.780.170.912
271.635.299.134.464	196.148.027.821.385	534.949.166.785.596	356.632.777.857.064
247.653.148.588.106	145.678.322.698.886	145.678.322.698.886	509.874.129.446.101
162.281.560.942.596	786.568.790.282.992	248.390.144.299.892	368.445.380.711.507

NFE2L2-mRNA	BNIP3-mRNA	COL5A2-mRNA	ASXL1-mRNA
654.415.656.565.196	605.872.973.848.172	916.306.639.829.646	712.911.906.637.312
760.663.614.504.974	682.902.856.638.619	112.792.757.751.822	73.887.905.129.822
596.784.625.265.135	589.745.692.475.995	105.895.493.001.875	750.626.616.743.548
799.951.395.362.768	601.563.361.899.095	107.550.017.033.725	744.264.375.993.587
742.131.372.969.299	6.227.141.182.004	931.028.241.730.424	811.268.245.741.908
747.862.116.676.451	652.775.287.409.538	927.210.621.365.847	788.278.928.079.469
739.925.763.435.048	504.256.412.130.245	961.130.811.146.547	713.622.322.851.669
755.130.716.348.009	714.531.480.380.425	837.797.556.059.452	814.531.480.380.425
809.115.014.230.405	654.626.508.095.823	7.666.295.784.665	718.086.895.749.476
826.070.406.080.198	62.381.614.921.509	821.814.359.857.394	747.347.847.957.684
677.160.490.721.535	667.206.923.366.443	786.471.431.160.683	744.967.681.232.799
798.503.327.297.049	587.739.254.978.252	902.111.707.911.597	690.755.452.029.277

NFE2L2-mRNA	BNIP3-mRNA	COL5A2-mRNA	ASXL1-mRNA
933.227.273.608.341	666.590.909.720.244	57.326.818.235.941	139.984.091.041.251
194.906.196.159.145	113.695.281.092.834	248.542.314.155.212	167.589.797.455.022
625.893.918.224.285	59.608.944.592.789	154.089.121.772.359	181.807.281.008.006
255.913.767.698.534	646.973.008.226.631	172.813.679.086.313	173.963.853.323.161
171.410.743.967.045	749.128.436.596.716	634.854.607.285.352	276.796.608.776.414
178.356.646.232.042	922.676.414.687.703	618.275.579.663.502	236.023.922.150.024
168.810.125.826.529	329.581.674.232.747	782.153.582.996.251	140.675.104.855.441
187.572.843.739.582	141.564.410.369.496	332.676.364.368.315	283.128.820.738.992
27.269.607.826.779	934.592.193.124.351	203.135.106.816.069	145.096.504.868.621
306.704.188.957.075	754.872.713.130.785	297.788.369.510.648	177.722.000.965.437
109.258.742.024.164	10.197.482.588.922	233.085.316.318.218	174.813.987.238.663
25.335.794.718.589	587.856.674.843.078	519.549.385.160.608	120.055.236.411.615

MUTYH-mRNA	DUSP2-mRNA	SPRY1-mRNA	INHBB-mRNA
573.680.164.359.436	473.680.164.359.436	612.911.906.637.312	543.724.136.173.545
464.970.486.694.163	302.167.364.432.859	600.518.552.154.002	552.802.631.035.338
582.345.634.331.618	357.552.882.987.259	582.345.634.331.618	603.496.044.850.989
491.609.794.544.004	512.864.258.082.014	686.955.535.950.301	655.352.786.605.533
492.946.063.336.331	359.242.564.608.574	717.104.661.993.307	589.908.698.431.979
506.024.795.492.241	342.083.767.017.887	703.781.056.580.265	489.032.295.348.009
497.041.433.554.661	543.989.961.884.783	837.849.907.418.369	628.492.495.887.149
552.382.642.705.798	657.827.421.108.036	646.724.289.869.161	805.220.539.941.277
503.826.456.205.195	441.533.421.113.178	782.404.607.216.121	611.132.802.424.168
470.890.842.401.603	424.947.680.537.873	859.377.271.329.455	486.418.664.949.394
518.664.240.649.419	486.471.431.160.683	718.664.240.649.419	695.217.715.285.717
472.764.543.027.784	3.312.607.930.999	750.243.248.987.901	385.692.844.722.281

MUTYH-mRNA	DUSP2-mRNA	SPRY1-mRNA	INHBB-mRNA
533.272.727.776.195	266.636.363.888.098	699.920.455.206.256	433.284.091.318.159
251.015.555.659.504	812.109.150.663.103	642.304.510.069.908	461.425.653.785.854
566.284.973.631.495	119.217.889.185.578	566.284.973.631.495	655.698.390.520.679
301.920.737.172.428	349.844.663.707.734	116.934.380.746.147	939.308.960.091.998
304.730.211.496.969	120.622.375.384.217	144.111.995.853.775	596.763.330.848.231
333.646.382.096.892	107.096.369.561.965	131.399.007.270.258	296.574.561.863.904
313.504.519.392.126	434.083.180.696.789	332.797.105.200.872	779.742.009.770.158
460.084.333.700.862	955.559.769.994.098	88.477.756.480.935	265.433.269.442.805
328.600.908.084.817	213.377.213.042.089	226.606.600.250.699	691.342.170.256.369
261.530.703.761.847	190.204.148.190.434	386.352.176.011.819	291.250.101.916.602
364.195.806.747.215	291.356.645.397.772	145.678.322.698.886	123.826.574.294.053
264.949.487.253.218	993.560.577.199.569	181.324.805.338.921	144.894.250.841.604

COL11A1-mRNA	AXIN1-mRNA	MMP3-mRNA	FGF19-mRNA
100.987.454.173.296	659.478.263.872.193	454.415.656.565.196	505.872.973.848.172
107.384.151.747.988	598.009.454.057.719	523.466.736.766.278	214.720.452.641.244
108.399.714.300.992	706.738.192.620.227	489.745.692.475.995	257.552.882.987.259
861.829.812.144.557	655.352.786.605.533	239.824.964.057.742	210.874.302.338.244
106.647.345.778.838	683.635.122.897.183	0.929460633363313	-0.655501867357843
117.768.663.268.091	655.328.796.620.252	289.032.295.348.009	189.032.295.348.009
111.346.766.551.606	676.182.771.373.519	300.694.021.157.172	249.236.703.874.197
873.027.730.452.541	818.093.871.353.497	106.435.656.713.321	607.131.422.236.047
829.307.846.108.078	669.034.125.863.165	109.340.611.624.442	109.340.611.624.442
915.185.191.986.476	665.886.774.151.643	157.140.490.026.609	0.249476805378731
859.263.476.617.003	686.471.431.160.683	603.463.931.304.914	518.664.240.649.419
955.816.063.725.468	626.680.424.138.587	1.312.607.930.999	-0.27235456972216

COL11A1-mRNA	AXIN1-mRNA	MMP3-mRNA	FGF19-mRNA
10.965.420.464.898	966.556.819.094.353	233.306.818.402.085	333.295.454.860.122
170.838.234.057.674	631.230.294.379.048	376.523.333.489.257	44.296.862.763.442
183.297.504.622.826	134.120.125.333.775	298.044.722.963.945	59.608.944.592.789
392.976.197.589.509	939.308.960.091.998	527.163.191.888.366	431.315.338.817.754
162.332.323.082.865	114.273.829.311.363	190.456.382.185.606	0.634854607285352
350.905.373.960.917	939.152.779.235.697	741.436.404.659.761	370.718.202.329.881
224.839.010.446.096	108.520.795.174.197	803.857.742.031.091	562.700.419.421.764
424.693.231.108.488	290.207.041.257.467	159.967.783.717.531	672.430.949.255.106
313.664.503.171.871	103.274.571.112.371	213.377.213.042.089	213.377.213.042.089
568.829.280.682.017	101.045.953.726.168	297.193.981.547.553	118.877.592.619.021
386.047.555.152.048	116.542.658.159.109	655.552.452.144.987	364.195.806.747.215
753.864.087.950.172	770.009.447.329.666	248.390.144.299.892	0.827967147666308

MCM4-mRNA	FANCB-mRNA	EPHA2-mRNA	E2F5-mRNA
454.415.656.565.196	432.176.414.431.551	705.872.973.848.172	598.472.915.703.794
577.169.539.132.024	384.764.424.455.354	910.624.047.582.583	497.163.296.182.899
57.454.538.313.149	47.454.538.313.149	727.596.854.801.368	47.454.538.313.149
588.133.252.727.936	379.679.901.706.769	834.183.004.551.512	496.118.583.496.858
498.835.432.241.688	480.392.975.127.945	782.831.390.990.641	525.138.872.825.068
517.982.957.067.508	384.968.096.898.275	87.441.523.053.372	532.031.079.422.491
511.127.687.138.646	314.444.373.532.166	799.789.507.196.872	426.997.461.740.552
49.933.117.103.592	463.074.163.097.449	793.191.116.569.506	591.084.955.016.723
526.333.111.768.673	385.894.086.260.739	774.302.157.530.783	526.333.111.768.673
545.893.017.100.768	364.179.422.815.749	749.740.431.882.232	460.702.880.999.682
486.471.431.160.683	444.967.681.232.799	644.967.681.232.799	486.471.431.160.683
518.707.704.891.514	242.808.514.841.893	792.486.212.338.789	289.757.043.172.015

MCM4-mRNA	FANCB-mRNA	EPHA2-mRNA	E2F5-mRNA
233.306.818.402.085	199.977.272.916.073	133.318.181.944.049	633.261.364.234.232
546.327.974.082.451	143.964.803.981.186	551.126.800.881.824	313.769.444.574.381
536.480.501.335.101	26.824.025.066.755	154.983.255.941.251	26.824.025.066.755
589.464.296.384.264	138.979.386.952.387	324.444.982.644.022	311.505.522.479.489
317.427.303.642.676	279.336.027.205.555	227.277.949.408.156	380.912.764.371.211
362.480.020.055.883	144.168.189.794.954	428.797.387.361.562	399.551.840.288.871
345.658.829.073.369	8.842.435.162.342	255.626.761.965.887	192.925.858.087.462
318.519.923.331.366	247.737.718.146.618	244.198.607.887.381	601.648.744.070.358
38.407.898.347.576	145.096.504.868.621	214.230.721.894.257	38.407.898.347.576
439.847.092.690.379	124.821.472.249.972	180.693.940.780.912	243.699.064.868.994
291.356.645.397.772	218.517.484.048.329	874.069.936.193.316	291.356.645.397.772
364.305.544.973.175	5.381.786.459.831	243.008.357.840.061	745.170.432.899.677

PCK1-mRNA	COL1A2-mRNA	MAP2K2-mRNA	RPS6KA5-mRNA
405.872.973.848.172	139.120.392.938.854	775.916.945.662.281	58.242.644.848.447
429.016.248.025.449	15.464.793.791.666	857.346.928.111.454	484.764.424.455.354
457.552.882.987.259	144.809.158.348.907	852.972.514.025.947	616.049.133.059.375
429.637.002.655.821	149.391.481.908.464	861.829.812.144.557	604.734.247.871.829
280.392.975.127.945	138.845.656.135.799	839.162.204.475.618	620.247.912.776.973
296.832.546.548.137	132.836.538.188.201	843.292.495.247.761	628.264.037.625.885
400.694.021.157.172	14.082.085.055.638	84.197.217.369.102	611.127.687.138.646
414.531.480.380.425	119.603.778.209.971	74.380.965.530.321	573.027.730.452.541
0.771478021357053	107.820.061.272.435	813.342.179.509.229	703.357.286.672.723
205.683.172.743.634	117.666.540.580.683	82.007.615.203.457	653.487.902.424.098
386.471.431.160.683	890.910.843.096.528	644.967.681.232.799	603.463.931.304.914
242.808.514.841.893	127.125.978.580.102	786.719.678.267.663	579.373.462.073.561

PCK1-mRNA	COL1A2-mRNA	MAP2K2-mRNA	RPS6KA5-mRNA
166.647.727.430.061	154.149.147.872.806	216.642.045.659.079	566.602.273.262.207
195.644.477.205.202	452.237.746.167.669	380.953.019.765.601	287.929.607.962.373
238.435.778.371.156	228.659.911.457.939	369.575.456.475.292	715.307.335.113.468
196.488.098.794.755	314.146.130.831.585	392.976.197.589.509	661.350.186.187.223
698.340.068.013.887	151.241.413.093.589	335.838.087.253.951	736.431.344.451.008
782.627.316.029.748	997.190.773.356.009	345.591.746.394.189	778.508.224.892.749
160.771.548.406.218	173.432.307.843.208	342.443.398.105.244	691.317.658.146.738
17.695.551.296.187	398.503.815.190.131	173.416.402.702.633	53.086.653.888.561
170.701.770.433.671	176.078.876.202.332	280.804.412.363.389	131.013.608.807.843
416.071.574.166.575	348.430.223.966.351	294.222.041.732.078	927.245.222.428.366
145.678.322.698.886	480.738.464.906.323	874.069.936.193.316	655.552.452.144.987
5.381.786.459.831	671.232.966.613.075	233.486.735.641.899	554.737.988.936.426

XRCC4-mRNA	NTRK2-mRNA	NOG-mRNA	HES1-mRNA
490.672.664.503.667	554.415.656.565.196	473.680.164.359.436	564.369.223.920.288
484.764.424.455.354	451.643.833.607.816	331.712.952.785.476	611.683.087.736.893
489.745.692.475.995	727.596.854.801.368	257.552.882.987.259	87.454.538.313.149
527.866.802.482.475	652.378.052.266.128	326.074.611.682.749	788.133.252.727.936
547.378.114.958.712	62.393.158.959.501	292.946.063.336.331	735.572.538.806.541
572.039.795.203.778	778.917.623.002.319	347.528.545.420.125	767.168.266.700.475
514.444.373.532.166	606.005.154.803.129	268.501.211.668.436	629.972.196.079.957
552.382.642.705.798	540.834.920.963.804	552.382.642.705.798	755.130.716.348.009
465.412.107.071.889	701.465.200.483	301.940.553.480.064	722.680.524.166.161
503.083.651.890.339	826.628.509.306.529	224.947.680.537.873	776.911.305.822.194
486.471.431.160.683	518.664.240.649.419	444.967.681.232.799	695.217.715.285.717
401.304.764.914.009	679.910.779.283.446	456.053.544.444.258	601.304.764.914.009

XRCC4-mRNA	NTRK2-mRNA	NOG-mRNA	HES1-mRNA
29.996.590.937.411	466.613.636.804.171	266.636.363.888.098	499.943.182.290.183
287.929.607.962.373	228.867.124.277.783	996.679.412.177.444	693.984.183.293.924
298.044.722.963.945	154.983.255.941.251	59.608.944.592.789	429.184.401.068.081
388.183.804.935.979	920.139.389.477.875	95.847.853.070.612	235.785.718.553.706
444.398.225.099.746	755.476.982.669.569	761.825.528.742.422	163.792.488.679.621
52.724.366.553.583	221.195.194.056.829	111.215.460.698.964	203.895.011.281.434
35.369.740.649.368	667.201.925.885.805	643.086.193.624.873	787.780.587.190.469
460.084.333.700.862	424.693.231.108.488	460.084.333.700.862	187.572.843.739.582
251.785.111.389.665	129.306.591.103.506	810.833.409.559.939	149.790.803.555.547
326.913.379.702.309	307.892.964.883.265	475.510.370.476.085	218.140.382.455.904
291.356.645.397.772	364.195.806.747.215	218.517.484.048.329	123.826.574.294.053
16.145.359.379.493	111.361.581.361.118	235.970.637.084.898	64.581.437.517.972

CCND1-mRNA	WT1-mRNA	HDAC5-mRNA	FANCG-mRNA
807.665.164.647.898	519.623.326.223.166	846.472.209.815.756	532.176.414.431.551
916.494.089.070.925	0.147204526412445	834.031.915.522.665	526.268.174.383.238
864.161.802.033.036	438.288.375.193.019	886.093.104.873.484	643.350.982.500.016
917.243.769.869.982	152.378.052.266.128	879.368.640.520.036	547.797.683.304.815
868.879.404.055.797	215.185.305.469.976	81.773.881.468.069	60.859.651.190.433
890.775.002.523.828	130.536.045.275.894	806.024.795.492.241	606.024.795.492.241
820.464.836.952.758	268.501.211.668.436	840.611.130.539.155	634.322.359.943.616
902.305.905.375.325	440.834.920.963.804	777.758.301.930.376	528.281.832.755.419
875.161.759.899.621	-0.228521978642947	787.476.582.976.907	501.940.553.480.064
875.131.399.028.103	-0.750523194621269	770.480.402.568.329	508.236.681.954.347
767.206.923.366.443	444.967.681.232.799	718.664.240.649.419	603.463.931.304.914
788.751.676.705.623	10.495.735.251.652	79.273.177.751.142	477.203.954.963.629

CCND1-mRNA	WT1-mRNA	HDAC5-mRNA	FANCG-mRNA
269.969.318.436.699	366.625.000.346.134	353.293.182.151.729	399.954.545.832.146
574.013.513.309.602	110.742.156.908.605	324.105.379.219.184	38.390.614.394.983
399.379.928.771.686	208.631.306.074.761	464.949.767.823.754	86.432.969.659.544
577.004.075.485.084	287.543.559.211.836	443.775.559.716.933	445.692.516.778.346
412.655.494.735.479	444.398.225.099.746	28.949.370.092.212	679.294.429.795.327
480.286.026.574.046	24.714.546.821.992	266.917.105.677.514	667.292.764.193.785
295.015.791.325.411	643.086.193.624.873	339.227.967.137.121	811.896.319.451.402
520.249.208.107.898	212.346.615.554.244	219.424.836.072.719	389.302.128.516.114
43.102.197.034.502	0.853508852168356	234.714.934.346.298	324.333.363.823.975
430.931.273.243.952	0.594387963095106	208.630.175.046.382	338.801.138.964.211
20.394.965.177.844	218.517.484.048.329	145.678.322.698.886	655.552.452.144.987
236.798.604.232.564	206.991.786.916.577	243.422.341.413.894	273.229.158.729.881

PRKAR2B-mRNA	IDH2-mRNA	CASP7-mRNA	LAMB3-mRNA
454.415.656.565.196	764.369.223.920.288	543.724.136.173.545	273.680.164.359.436
47.321.670.271.336	785.456.365.849.333	564.970.486.694.163	429.016.248.025.449
438.288.375.193.019	740.841.884.403.733	47.454.538.313.149	157.552.882.987.259
398.321.214.129.858	876.695.450.613.423	639.824.964.057.742	439.824.964.057.742
466.642.622.752.952	747.892.445.286.308	580.392.975.127.945	280.392.975.127.945
436.425.414.181.251	733.142.274.934.513	624.395.990.809.479	189.032.295.348.009
326.997.461.740.552	775.110.130.714.213	55.676.551.660.462	168.501.211.668.436
591.084.955.016.723	724.965.146.361.899	582.338.670.891.689	282.338.670.891.689
523.090.963.999.435	747.883.715.343.794	650.618.764.158.289	474.875.794.485.697
586.418.664.949.394	743.930.136.425.875	613.211.985.474.057	420.367.311.576.561
386.471.431.160.683	586.471.431.160.683	667.206.923.366.443	286.471.431.160.683
493.709.879.590.679	711.566.271.562.298	523.544.007.047.654	30.495.735.251.652

PRKAR2B-mRNA	IDH2-mRNA	CASP7-mRNA	LAMB3-mRNA
233.306.818.402.085	199.977.272.916.073	433.284.091.318.159	666.590.909.720.244
265.781.176.580.652	231.451.107.938.984	502.031.111.319.009	195.644.477.205.202
208.631.306.074.761	169.885.492.089.449	26.824.025.066.755	298.044.722.963.945
15.814.895.756.651	435.628.492.205.931	843.461.107.021.386	210.865.276.755.346
253.941.842.914.141	178.394.144.647.184	55.867.205.441.111	698.340.068.013.887
205.954.556.849.934	161.056.463.456.648	757.912.769.207.756	370.718.202.329.881
964.629.290.437.309	215.433.874.864.332	474.276.067.798.344	321.543.096.812.436
601.648.744.070.358	152.181.741.147.208	566.257.641.477.984	70.782.205.184.748
375.543.894.954.077	178.383.350.103.187	9.089.869.275.593	268.855.288.433.032
582.500.203.833.204	173.561.285.223.771	701.377.796.452.226	184.260.268.559.483
145.678.322.698.886	582.713.290.795.544	10.197.482.588.922	72.839.161.349.443
306.347.844.636.534	138.684.497.234.107	37.672.505.218.817	827.967.147.666.308

FGF3-mRNA	BCL2L1-mRNA	PTCRA-mRNA	IL6-mRNA
405.872.973.848.172	719.623.326.223.166	573.680.164.359.436	332.176.414.431.551
202.167.364.432.859	830.370.901.209.244	202.167.364.432.859	214.720.452.641.244
457.552.882.987.259	782.345.634.331.618	316.049.133.059.375	389.745.692.475.995
174.617.294.399.773	871.853.737.708.353	226.074.611.682.749	239.824.964.057.742
280.392.975.127.945	797.749.332.978.511	134.449.813.264.216	280.392.975.127.945
204.232.604.692.514	756.902.089.246.712	104.232.604.692.514	26.272.885.476.463
497.041.433.554.661	816.882.789.394.862	226.997.461.740.552	326.997.461.740.552
552.382.642.705.798	812.716.745.709.399	573.027.730.452.541	930.720.248.618.114
0.356440522078209	843.858.956.343.208	-122.852.197.864.295	0.356440522078209
205.683.172.743.634	829.387.092.473.718	157.140.490.026.609	445.893.017.100.768
444.967.681.232.799	686.471.431.160.683	486.471.431.160.683	444.967.681.232.799
153.500.035.233.544	797.081.941.375.079	-0.27235456972216	172.764.543.027.784

FGF3-mRNA	BCL2L1-mRNA	PTCRA-mRNA	IL6-mRNA
166.647.727.430.061	146.650.000.138.454	533.272.727.776.195	999.886.364.580.366
406.054.575.331.551	315.984.287.712.553	406.054.575.331.551	44.296.862.763.442
238.435.778.371.156	226.513.989.452.598	894.134.168.891.835	149.022.361.481.972
335.467.485.747.142	42.125.131.424.534	47.923.926.535.306	527.163.191.888.366
698.340.068.013.887	252.037.279.092.285	253.941.842.914.141	698.340.068.013.887
411.909.113.699.867	189.890.101.415.639	205.954.556.849.934	617.863.670.549.801
313.504.519.392.126	28.778.107.164.713	482.314.645.218.654	964.629.290.437.309
460.084.333.700.862	279.589.710.479.755	53.086.653.888.561	633.500.736.403.494
128.026.327.825.253	346.951.348.406.437	0.426754426084178	128.026.327.825.253
416.071.574.166.575	313.836.844.514.216	297.193.981.547.553	219.923.546.345.189
218.517.484.048.329	116.542.658.159.109	291.356.645.397.772	218.517.484.048.329
289.788.501.683.208	250.874.045.742.891	0.827967147666308	331.186.859.066.523

COL4A3-mRNA	LEF1-mRNA	IL22RA1-mRNA	MAPK8-mRNA
405.872.973.848.172	543.724.136.173.545	564.369.223.920.288	543.724.136.173.545
114.720.452.641.244	762.023.374.845.046	0.884170120578651	631.043.487.528.075
157.552.882.987.259	457.552.882.987.259	503.496.044.850.989	621.938.501.964.731
310.874.302.338.244	691.036.157.589.089	339.824.964.057.742	648.571.248.182.776
280.392.975.127.945	672.820.242.511.621	315.185.305.469.976	613.891.399.899.226
272.039.795.203.778	622.819.259.223.648	230.536.045.275.894	592.007.029.687.415
268.501.211.668.436	595.179.865.737.926	326.997.461.740.552	566.229.204.018.428
540.834.920.963.804	552.382.642.705.798	514.531.480.380.425	49.933.117.103.592
323.090.963.999.435	529.503.997.741.407	419.774.277.605.915	599.064.654.181.921
370.890.842.401.603	494.991.652.351.982	224.947.680.537.873	599.094.379.177.988
444.967.681.232.799	586.471.431.160.683	567.206.923.366.443	667.206.923.366.443
189.757.043.172.015	599.443.197.097.274	0.72764543027784	585.692.844.722.281

COL4A3-mRNA	LEF1-mRNA	IL22RA1-mRNA	MAPK8-mRNA
166.647.727.430.061	433.284.091.318.159	499.943.182.290.183	433.284.091.318.159
22.148.431.381.721	196.751.898.774.288	184.570.261.514.342	793.652.124.511.669
298.044.722.963.945	238.435.778.371.156	32.784.919.526.034	745.111.807.409.862
862.630.677.635.508	120.289.055.603.618	105.432.638.377.673	896.177.426.210.222
698.340.068.013.887	106.020.719.416.654	888.796.450.199.493	704.688.614.086.741
659.054.581.919.788	749.674.586.933.758	494.290.936.439.841	605.506.397.138.805
643.086.193.624.873	61.897.046.136.394	964.629.290.437.309	506.430.377.479.587
424.693.231.108.488	460.084.333.700.862	35.391.102.592.374	318.519.923.331.366
938.859.737.385.192	392.614.071.997.444	183.504.403.216.197	635.864.094.865.425
130.765.351.880.923	309.081.740.809.455	475.510.370.476.085	635.995.120.511.764
218.517.484.048.329	582.713.290.795.544	509.874.129.446.101	10.197.482.588.922
372.585.216.449.838	637.534.703.703.057	165.593.429.533.262	579.577.003.366.415

**FLT1-mRNA**	**CD19-mRNA**	**CACNA2D3-mRNA**	**MSH2-mRNA**
590.672.664.503.667	532.176.414.431.551	543.724.136.173.545	619.623.326.223.166
596.312.146.197.347	136.959.694.774.889	641.399.106.710.735	60.936.234.862.076
503.496.044.850.989	389.745.692.475.995	527.596.854.801.368	60.990.907.859.296
703.157.516.285.998	252.378.052.266.128	433.113.544.471.888	625.170.097.722.448
655.395.149.827.111	325.138.872.825.068	692.192.696.067.791	654.417.047.747.852
620.421.372.930.204	421.225.104.836.746	64.346.434.697.039	649.518.501.163.895
543.989.961.884.783	314.444.373.532.166	779.875.428.273.355	650.519.107.909.955
59.933.117.103.592	552.382.642.705.798	382.338.670.891.689	573.027.730.452.541
588.000.247.813.522	323.090.963.999.435	571.399.252.669.629	682.132.657.080.762
728.839.579.467.103	270.890.842.401.603	657.140.490.026.609	638.902.815.777.752
632.414.593.024.413	518.664.240.649.419	518.664.240.649.419	711.264.182.505.042
575.001.324.330.629	342.808.514.841.893	503.142.617.845.494	653.500.035.233.544

**FLT1-mRNA**	**CD19-mRNA**	**CACNA2D3-mRNA**	**MSH2-mRNA**
599.931.818.748.219	399.954.545.832.146	433.284.091.318.159	733.250.000.692.268
623.847.483.918.474	258.398.366.120.078	852.714.608.196.258	682.909.967.603.064
32.784.919.526.034	149.022.361.481.972	387.458.139.853.129	685.502.862.817.074
130.832.319.441.385	575.087.118.423.672	201.280.491.448.285	761.990.431.911.366
939.584.818.782.321	952.281.910.928.028	121.257.229.991.502	933.236.272.709.468
737.317.313.522.762	18.535.910.116.494	865.009.138.769.721	902.080.959.002.709
434.083.180.696.789	8.842.435.162.342	222.668.594.542.612	908.359.248.495.133
637.039.846.662.732	460.084.333.700.862	141.564.410.369.496	53.086.653.888.561
588.921.107.996.166	938.859.737.385.192	524.907.944.083.539	113.089.922.912.307
156.324.034.294.013	653.826.759.404.617	95.102.074.095.217	8.380.870.279.641
801.230.774.843.872	364.195.806.747.215	364.195.806.747.215	138.394.406.563.942
5.381.786.459.831	10.763.572.919.662	327.047.023.328.192	927.323.205.386.264

PPP3R2-mRNA	CAPN2-mRNA	STAG2-mRNA	PAK7-mRNA
373.680.164.359.436	764.369.223.920.288	564.369.223.920.288	505.872.973.848.172
214.720.452.641.244	866.421.769.664.052	724.172.212.519.673	0.562242025691289
257.552.882.987.259	861.992.294.923.104	638.288.375.193.019	357.552.882.987.259
-106.118.197.805.988	923.343.877.083.175	681.533.496.850.512	239.824.964.057.742
166.642.622.752.952	950.563.001.562.646	678.744.162.849.088	251.442.313.408.447
104.232.604.692.514	961.899.933.944.172	66.272.885.476.463	230.536.045.275.894
268.501.211.668.436	916.074.554.765.076	593.293.963.012.795	285.493.711.812.667
382.338.670.891.689	846.724.289.869.161	707.131.422.236.047	340.834.920.963.804
135.644.052.207.821	944.301.908.806.894	717.235.745.763.924	135.644.052.207.821
0.249476805378731	934.355.449.105.064	719.199.131.071.797	157.140.490.026.609
444.967.681.232.799	869.760.432.577.157	486.471.431.160.683	486.471.431.160.683
153.500.035.233.544	885.692.844.722.281	682.567.751.323.837	218.707.704.891.514

PPP3R2-mRNA	CAPN2-mRNA	STAG2-mRNA	PAK7-mRNA
133.318.181.944.049	199.977.272.916.073	499.943.182.290.183	333.295.454.860.122
44.296.862.763.442	405.685.434.808.523	15.134.761.444.176	147.656.209.211.473
59.608.944.592.789	393.419.034.312.407	834.525.224.299.046	119.217.889.185.578
0.47923926535306	601.924.517.283.443	112.621.227.357.969	527.163.191.888.366
317.427.303.642.676	726.908.525.341.729	110.464.701.667.651	571.369.146.556.817
205.954.556.849.934	786.334.498.053.046	988.581.872.879.682	494.290.936.439.841
643.086.193.624.873	572.346.712.326.137	610.931.883.943.629	723.471.967.827.982
141.564.410.369.496	35.391.102.592.374	134.486.189.851.021	106.173.307.777.122
256.052.655.650.507	696.036.468.943.294	144.242.996.016.452	256.052.655.650.507
118.877.592.619.021	649.666.043.662.951	146.219.438.921.396	297.193.981.547.553
218.517.484.048.329	415.183.219.691.825	291.356.645.397.772	291.356.645.397.772
289.788.501.683.208	463.661.602.693.132	113.431.499.230.284	455.381.931.216.469

PHF6-mRNA	KDM5C-mRNA	NFKB1-mRNA	LTBP1-mRNA
590.672.664.503.667	75.696.916.577.591	432.176.414.431.551	690.672.664.503.667
497.163.296.182.899	798.850.678.039.339	431.712.952.785.476	917.879.086.947.028
557.552.882.987.259	796.784.625.265.135	389.745.692.475.995	748.241.942.548.111
606.810.103.888.509	807.324.434.216.105	426.074.611.682.749	909.363.613.099.223
600.270.961.539.395	865.511.091.430.169	392.946.063.336.331	805.185.726.472.304
590.030.704.205.272	870.908.263.880.995	384.968.096.898.275	900.232.797.899.323
549.236.703.874.196	75.179.021.308.491	359.190.271.229.288	871.291.811.325.425
482.338.670.891.689	848.159.819.166.868	540.834.920.963.804	795.266.972.586.185
5.980.931.386.986	84.117.229.575.794	435.644.052.207.821	94.465.529.417.425
592.190.214.735.023	816.535.618.421.451	415.636.740.098.725	898.587.873.669.702
518.664.240.649.419	803.463.931.304.914	286.471.431.160.683	786.471.431.160.683
495.646.412.077.372	744.874.461.898.503	397.557.294.372.143	990.879.768.714.341

PHF6-mRNA	KDM5C-mRNA	NFKB1-mRNA	LTBP1-mRNA
599.931.818.748.219	189.978.409.270.269	199.977.272.916.073	119.986.363.749.644
313.769.444.574.381	253.968.679.843.734	199.335.882.435.489	579.550.621.155.033
476.871.556.742.312	250.357.567.289.714	149.022.361.481.972	178.826.833.778.367
670.934.971.494.284	26.933.246.712.842	191.695.706.141.224	546.332.762.502.488
641.203.153.358.206	403.132.675.626.199	152.365.105.748.484	265.369.225.845.277
597.268.214.864.808	418.499.659.519.065	144.168.189.794.954	512.826.846.556.335
450.160.335.537.411	183.279.565.183.089	120.578.661.304.664	41.961.374.134.023
283.128.820.738.992	357.450.136.182.977	424.693.231.108.488	247.737.718.146.618
631.596.550.604.584	340.550.032.015.174	204.842.124.520.406	697.743.486.647.631
606.275.722.357.008	287.089.386.174.937	178.316.388.928.532	507.012.932.520.126
364.195.806.747.215	262.220.980.857.995	72.839.161.349.443	233.085.316.318.218
310.487.680.374.865	174.701.068.157.591	157.313.758.056.598	961.269.858.440.582

GRB2-mRNA	COL6A6-mRNA	SMC1B-mRNA	RASGRF2-mRNA
726.036.359.965.137	432.176.414.431.551	173.680.164.359.436	612.911.906.637.312
75.395.219.491.912	0.884170120578651	214.720.452.641.244	556.224.202.569.129
703.496.044.850.989	316.049.133.059.375	357.552.882.987.259	489.745.692.475.995
804.734.247.871.829	210.874.302.338.244	126.074.611.682.749	579.679.901.706.769
784.035.315.952.933	192.946.063.336.331	166.642.622.752.952	699.554.982.382.109
765.113.528.960.067	152.775.287.409.539	104.232.604.692.514	635.702.257.258.143
707.732.953.946.312	268.501.211.668.436	226.997.461.740.552	583.475.923.618.904
728.281.832.755.419	382.338.670.891.689	282.338.670.891.689	463.074.163.097.449
729.111.427.420.027	223.090.963.999.435	-122.852.197.864.295	507.525.876.953.416
758.932.680.826.336	157.140.490.026.609	124.947.680.537.873	486.418.664.949.394
618.664.240.649.419	486.471.431.160.683	486.471.431.160.683	618.664.240.649.419
676.106.843.181.529	10.495.735.251.652	0.72764543027784	594.681.395.074

GRB2-mRNA	COL6A6-mRNA	SMC1B-mRNA	RASGRF2-mRNA
153.315.909.235.656	199.977.272.916.073	333.295.454.860.122	699.920.455.206.256
186.046.823.606.456	184.570.261.514.342	44.296.862.763.442	472.499.869.476.714
131.139.678.104.136	894.134.168.891.835	119.217.889.185.578	298.044.722.963.945
264.540.074.474.889	431.315.338.817.754	23.961.963.267.653	55.591.754.780.955
229.182.513.230.012	380.912.764.371.211	317.427.303.642.676	127.605.776.064.356
201.011.647.485.535	288.336.379.589.907	205.954.556.849.934	819.699.136.262.736
135.048.100.661.223	643.086.193.624.873	482.314.645.218.654	570.738.996.842.075
155.720.851.406.446	141.564.410.369.496	70.782.205.184.748	247.737.718.146.618
156.618.874.372.893	469.429.868.692.596	0.426754426084178	337.135.996.606.501
192.581.700.042.814	297.193.981.547.553	237.755.185.238.043	291.250.101.916.602
728.391.613.494.429	291.356.645.397.772	291.356.645.397.772	728.391.613.494.429
108.463.696.344.286	206.991.786.916.577	165.593.429.533.262	616.835.525.011.399

IL12A-mRNA	CCNB1-mRNA	SMAD3-mRNA	RUNX1-mRNA
373.680.164.359.436	532.176.414.431.551	766.753.898.115.724	705.872.973.848.172
236.959.694.774.889	497.163.296.182.899	101.004.309.577.437	623.466.736.766.278
257.552.882.987.259	489.745.692.475.995	875.543.791.988.752	855.280.875.337.251
339.824.964.057.742	526.074.611.682.749	841.455.145.290.652	829.416.911.836.493
292.946.063.336.331	466.642.622.752.952	820.247.912.776.973	842.930.652.044.652
280.786.079.328.812	367.459.426.242.466	826.536.238.482.702	810.626.035.267.924
200.694.021.157.172	338.545.183.482.545	866.229.204.018.428	845.319.644.146.129
282.338.670.891.689	514.531.480.380.425	728.281.832.755.419	810.878.892.777.914
294.140.302.279.937	435.644.052.207.821	86.798.706.421.308	811.577.392.927.287
0.834439306099887	364.179.422.815.749	799.766.965.496.819	711.984.152.496.214
386.471.431.160.683	386.471.431.160.683	686.471.431.160.683	832.414.593.024.413
20.495.735.251.652	358.562.642.540.541	936.245.648.044.956	719.116.980.354.902

IL12A-mRNA	CCNB1-mRNA	SMAD3-mRNA	RUNX1-mRNA
133.318.181.944.049	399.954.545.832.146	203.310.227.464.674	133.318.181.944.049
516.796.732.240.156	313.769.444.574.381	10.978.239.154.873	753.046.666.978.513
59.608.944.592.789	298.044.722.963.945	43.216.484.829.772	375.536.350.934.571
105.432.638.377.673	383.391.412.282.448	341.218.356.931.379	313.901.718.806.254
761.825.528.742.422	253.941.842.914.141	294.572.537.780.403	344.726.051.755.946
700.245.493.289.774	127.691.825.246.959	307.696.107.933.801	275.567.197.065.211
401.928.871.015.545	104.501.506.464.042	405.144.301.983.669	350.481.975.525.556
70.782.205.184.748	35.391.102.592.374	155.720.851.406.446	276.050.600.220.517
768.157.966.951.521	204.842.124.520.406	410.111.003.466.895	277.390.376.954.716
178.316.388.928.532	124.821.472.249.972	255.586.824.130.896	139.086.783.364.255
145.678.322.698.886	145.678.322.698.886	116.542.658.159.109	320.492.309.937.549
413.983.573.833.154	120.055.236.411.615	658.233.882.394.715	146.136.201.563.103

PRMT8-mRNA	CALML6-mRNA	MDC1-mRNA	LAMA5-mRNA
432.176.414.431.551	432.176.414.431.551	68.242.644.848.447	802.220.386.245.661
226.268.174.383.238	188.417.012.057.865	646.913.262.129.981	589.315.890.380.591
416.049.133.059.375	157.552.882.987.259	682.345.634.331.618	770.481.184.681.756
318.674.553.538.371	293.881.802.194.012	661.829.812.144.557	703.157.516.285.998
304.493.785.078.325	292.946.063.336.331	674.537.756.892.434	781.210.368.272.515
152.775.287.409.539	304.232.604.692.514	648.858.227.681.471	793.714.381.023.309
126.997.461.740.552	268.501.211.668.436	689.446.548.231.331	772.393.110.597.666
382.338.670.891.689	463.074.163.097.449	646.724.289.869.161	791.084.955.016.723
329.503.997.741.407	294.140.302.279.937	653.966.234.613.398	779.106.874.971.493
0.249476805378731	283.443.930.609.989	677.303.876.143.574	802.755.393.491.409
444.967.681.232.799	544.967.681.232.799	567.206.923.366.443	864.607.402.513.149
0.312607930998996	153.500.035.233.544	669.918.898.422.861	679.910.779.283.446

PRMT8-mRNA	CALML6-mRNA	MDC1-mRNA	LAMA5-mRNA
199.977.272.916.073	199.977.272.916.073	113.320.454.652.441	259.970.454.790.895
479.882.679.937.288	369.140.523.028.683	885.937.255.268.839	59.431.624.207.618
178.826.833.778.367	298.044.722.963.945	113.256.994.726.299	208.631.306.074.761
910.554.604.170.814	766.782.824.564.896	982.440.493.973.773	130.832.319.441.385
825.310.989.470.958	761.825.528.742.422	107.290.428.631.225	224.738.530.979.015
288.336.379.589.907	823.818.227.399.735	89.796.186.786.571	245.085.922.651.421
241.157.322.609.327	643.086.193.624.873	118.970.945.820.601	211.414.586.154.177
141.564.410.369.496	247.737.718.146.618	88.477.756.480.935	240.659.497.628.143
98.153.517.999.361	768.157.966.951.521	930.324.648.863.508	221.485.547.137.689
118.877.592.619.021	713.265.555.714.128	1.093.673.852.095	260.936.315.798.752
218.517.484.048.329	437.034.968.096.658	509.874.129.446.101	400.615.387.421.936
124.195.072.149.946	289.788.501.683.208	103.909.877.032.122	111.361.581.361.118

TSC1-mRNA	NTRK1-mRNA	PIM1-mRNA	CSF2-mRNA
626.036.359.965.137	473.680.164.359.436	686.608.466.053.932	373.680.164.359.436
635.014.458.508.272	326.268.174.383.238	733.373.149.519.189	226.268.174.383.238
700.179.358.457.469	389.745.692.475.995	77.454.538.313.149	257.552.882.987.259
665.306.353.960.625	439.824.964.057.742	742.665.805.576.317	126.074.611.682.749
710.604.936.508.664	557.331.682.313.804	720.868.427.729.644	215.185.305.469.976
690.527.329.494.606	59.102.225.109.178	681.843.003.499.831	104.232.604.692.514
704.256.412.130.245	625.486.772.501.531	68.649.212.066.993	249.236.703.874.197
563.074.163.097.449	563.074.163.097.449	932.323.259.600.009	463.074.163.097.449
698.579.714.215.782	335.644.052.207.821	635.644.052.207.821	-122.852.197.864.295
684.938.964.756.586	51.074.578.005.063	663.318.109.785.278	-0.750523194621269
603.463.931.304.914	567.206.923.366.443	618.664.240.649.419	286.471.431.160.683
637.150.162.005.256	289.757.043.172.015	616.227.365.791.456	-127.235.456.972.216

TSC1-mRNA	NTRK1-mRNA	PIM1-mRNA	CSF2-mRNA
766.579.546.178.281	266.636.363.888.098	116.653.409.201.043	133.318.181.944.049
81.580.055.589.339	959.765.359.874.576	161.314.408.563.535	479.882.679.937.288
128.159.230.874.496	149.022.361.481.972	21.459.220.053.404	59.608.944.592.789
100.640.245.724.143	210.865.276.755.346	172.046.896.261.749	23.961.963.267.653
137.763.449.780.921	476.140.955.464.014	147.921.123.497.487	444.398.225.099.746
119.865.552.086.661	601.387.306.001.806	112.863.097.153.764	205.954.556.849.934
131.832.669.693.099	763.664.854.929.536	116.559.372.594.508	562.700.419.421.764
495.475.436.293.236	495.475.436.293.236	640.578.956.921.969	247.737.718.146.618
126.746.064.547.001	102.421.062.260.203	819.368.498.081.622	0.426754426084178
115.311.264.840.451	344.745.018.595.162	992.627.898.368.827	0.594387963095106
655.552.452.144.987	509.874.129.446.101	728.391.613.494.429	72.839.161.349.443
827.967.147.666.307	745.170.432.899.677	716.191.582.731.356	0.413983573833154

CCNB3-mRNA	FAS-mRNA	PCNA-mRNA	IL15-mRNA
332.176.414.431.551	590.672.664.503.667	554.415.656.565.196	573.680.164.359.436
202.167.364.432.859	631.712.952.785.476	634.360.173.921.595	351.643.833.607.816
157.552.882.987.259	457.552.882.987.259	582.345.634.331.618	389.745.692.475.995
226.074.611.682.749	563.925.774.008.121	562.531.854.912.334	44.306.711.182.698
251.442.313.408.447	575.388.906.877.986	504.493.785.078.325	534.449.813.264.216
189.032.295.348.009	570.908.263.880.995	567.459.426.242.466	565.113.528.960.067
268.501.211.668.436	706.005.154.803.129	554.299.311.181.193	609.440.305.282.206
340.834.920.963.804	573.027.730.452.541	582.338.670.891.689	463.074.163.097.449
294.140.302.279.937	612.020.617.558.813	524.721.145.232.345	488.000.247.813.522
0.249476805378731	608.236.681.954.347	557.140.490.026.609	594.991.652.351.982
386.471.431.160.683	656.515.402.974.792	544.967.681.232.799	386.471.431.160.683
0.72764543027784	639.298.134.746.302	553.500.035.233.544	59.273.177.751.142

CCNB3-mRNA	FAS-mRNA	PCNA-mRNA	IL15-mRNA
999.886.364.580.366	599.931.818.748.219	466.613.636.804.171	533.272.727.776.195
406.054.575.331.551	797.343.529.741.955	812.109.150.663.103	114.433.562.138.892
298.044.722.963.945	238.435.778.371.156	566.284.973.631.495	149.022.361.481.972
47.923.926.535.306	498.408.835.967.182	493.616.443.313.652	215.657.669.408.877
571.369.146.556.817	539.626.416.192.549	330.124.395.788.383	406.306.948.662.625
370.718.202.329.881	523.124.574.398.831	510.767.300.987.835	502.529.118.713.838
643.086.193.624.873	133.440.385.177.161	466.237.490.378.033	683.279.080.726.427
106.173.307.777.122	53.086.653.888.561	566.257.641.477.984	247.737.718.146.618
768.157.966.951.521	69.560.971.451.721	379.811.439.214.919	294.460.553.998.083
118.877.592.619.021	677.602.277.928.421	475.510.370.476.085	61.816.348.161.891
145.678.322.698.886	946.909.097.542.759	437.034.968.096.658	145.678.322.698.886
165.593.429.533.262	840.386.654.881.302	463.661.602.693.132	608.555.853.534.736

DUSP8-mRNA	PPP3CB-mRNA	FGF5-mRNA	IL12RB2-mRNA
664.369.223.920.288	800.358.818.428.926	373.680.164.359.436	432.176.414.431.551
469.152.504.263.625	724.523.660.937.297	114.720.452.641.244	188.417.012.057.865
457.552.882.987.259	851.804.333.521.183	157.552.882.987.259	157.552.882.987.259
477.170.803.610.486	742.665.805.576.317	174.617.294.399.773	239.824.964.057.742
380.392.975.127.945	762.526.890.277.276	0.929460633363313	280.392.975.127.945
414.666.270.673.988	800.348.630.506.178	172.039.795.203.778	26.272.885.476.463
420.857.407.274.137	822.417.092.779.239	0.685012116684361	349.236.703.874.197
673.027.730.452.541	591.084.955.016.723	340.834.920.963.804	482.338.670.891.689
477.147.802.135.705	723.090.963.999.435	-122.852.197.864.295	194.140.302.279.937
492.190.214.735.023	727.738.280.194.862	-0.750523194621269	257.140.490.026.609
667.206.923.366.443	695.217.715.285.717	444.967.681.232.799	544.967.681.232.799
393.709.879.590.679	721.949.852.660.751	-127.235.456.972.216	281.510.827.152.818

DUSP8-mRNA	PPP3CB-mRNA	FGF5-mRNA	IL12RB2-mRNA
999.886.364.580.366	256.637.500.242.294	133.318.181.944.049	199.977.272.916.073
258.398.366.120.078	151.716.754.964.789	22.148.431.381.721	369.140.523.028.683
238.435.778.371.156	366.595.009.245.652	298.044.722.963.945	298.044.722.963.945
273.166.381.251.244	172.046.896.261.749	335.467.485.747.142	527.163.191.888.366
139.668.013.602.777	197.439.782.865.745	190.456.382.185.606	698.340.068.013.887
177.120.918.890.943	256.619.377.835.018	329.527.290.959.894	617.863.670.549.801
184.887.280.667.151	299.035.080.035.566	160.771.548.406.218	112.540.083.884.353
106.173.307.777.122	601.648.744.070.358	106.173.307.777.122	283.128.820.738.992
273.122.832.693.874	150.217.557.981.631	0.426754426084178	38.407.898.347.576
303.137.861.178.504	155.135.258.367.823	0.594387963095106	594.387.963.095.106
10.197.482.588.922	123.826.574.294.053	218.517.484.048.329	437.034.968.096.658
153.173.922.318.267	149.034.086.579.935	0.413983573833154	703.772.075.516.361

**GHR-mRNA**	**PLCG2-mRNA**	**ID1-mRNA**	**CASP9-mRNA**
519.623.326.223.166	58.242.644.848.447	798.472.915.703.794	373.680.164.359.436
405.409.512.202.096	522.045.350.844.308	98.066.058.608.118	431.712.952.785.476
621.938.501.964.731	527.596.854.801.368	975.045.451.237.327	416.049.133.059.375
426.074.611.682.749	613.849.036.677.649	104.977.170.030.868	466.673.847.650.332
398.835.432.241.688	658.290.287.196.724	96.675.528.929.838	473.681.555.542.092
444.831.840.660.098	703.328.090.732.214	943.807.437.510.418	482.892.240.881.595
385.493.711.812.667	619.280.675.688.306	806.005.154.803.129	504.256.412.130.245
49.933.117.103.592	563.074.163.097.449	855.130.716.348.009	282.338.670.891.689
460.436.803.552.179	650.618.764.158.289	893.764.110.400.317	599.064.654.181.921
445.893.017.100.768	643.930.136.425.875	984.193.384.264.681	53.787.598.223.237
386.471.431.160.683	711.264.182.505.042	87.226.953.067.344	444.967.681.232.799
540.007.077.224.934	611.134.972.275.189	857.783.226.792.361	55.090.051.438.025

**GHR-mRNA**	**PLCG2-mRNA**	**ID1-mRNA**	**CASP9-mRNA**
366.625.000.346.134	566.602.273.262.207	253.304.545.693.693	133.318.181.944.049
166.113.235.362.907	37.283.192.825.897	895.534.908.867.585	199.335.882.435.489
745.111.807.409.862	387.458.139.853.129	861.349.249.365.801	178.826.833.778.367
191.695.706.141.224	704.481.720.068.998	144.586.486.357.018	253.996.810.637.122
158.713.651.821.338	958.630.457.000.881	813.248.751.932.536	266.638.935.059.848
21.831.183.026.093	130.987.098.156.558	693.654.947.470.576	284.217.288.452.908
144.694.393.565.596	731.510.545.248.293	266.880.770.354.322	329.581.674.232.747
318.519.923.331.366	495.475.436.293.236	375.145.687.479.164	70.782.205.184.748
243.250.022.867.982	9.089.869.275.593	490.340.835.570.721	635.864.094.865.425
219.923.546.345.189	867.806.426.118.856	917.735.015.018.843	416.071.574.166.574
145.678.322.698.886	138.394.406.563.942	422.467.135.826.769	218.517.484.048.329
422.263.245.309.817	691.352.568.301.367	382.106.838.648.001	455.381.931.216.469

Gene	8910-Osteome	9185-Osteome	8914-Osteome
RAD51	2.46913262129981	0.72764543027784	0.771478021357053
GRIN2B	-0.437757974308711	0.312607930998996	0.356440522078209
IL19	-1.43775797430871	-0.27235456972216	1.57883294341466
IFNG	-1.43775797430871	0.312607930998996	-0.228521978642947
RASAL1	-1.43775797430871	0.72764543027784	2.09340611624442
RASGRF1	1.14720452641244	1.312607930999	2.77147802135705
CLCF1	1.14720452641244	0.312607930998996	0.771478021357053
PRKCG	0.147204526412445	0.72764543027784	-1.22852197864295
TSHR	-0.437757974308711	0.312607930998996	-0.228521978642947
SMC1B	2.14720452641244	0.72764543027784	-1.22852197864295
WNT16	0.562242025691289	0.312607930998996	0.356440522078209
ALK	1.14720452641244	1.72764543027784	2.67836861696557
IL13RA2	2.36959694774889	1.89757043172015	0.356440522078209
SSX1	1.36959694774889	-1.27235456972216	0.356440522078209
FGF6	2.14720452641244	0.312607930998996	1.09340611624442
GNGT1	0.884170120578651	0.72764543027784	2.23090963999435
GATA3	1.14720452641244	1.53500035233544	1.57883294341466
PRL	4.69152504263625	-1.27235456972216	-0.228521978642947
WNT2	4.46913262129981	-1.27235456972216	1.35644052207821
LAMA1	2.14720452641244	-1.27235456972216	1.77147802135705
MPO	2.02167364432859	0.72764543027784	-0.228521978642947
PPP2R2B	1.88417012057865	1.312607930999	1.94140302279937
FGF16	1.56224202569129	1.53500035233544	1.57883294341466
CALML5	1.36959694774889	0.72764543027784	1.35644052207821
PRKACG	0.562242025691289	0.312607930998996	1.35644052207821
IL23R	0.562242025691289	0.72764543027784	2.47191773949815
CACNG1	0.884170120578651	1.72764543027784	1.35644052207821
HNF1A	1.36959694774889	1.312607930999	1.09340611624442
NODAL	1.7321670271336	0.312607930998996	1.09340611624442
LEFTY1	2.26268174383238	0.312607930998996	-0.228521978642947
GATA1	1.14720452641244	-1.27235456972216	1.35644052207821
IL22RA2	0.884170120578651	0.312607930998996	0.356440522078209
IL1A	1.36959694774889	1.312607930999	-1.22852197864295
PTCRA	2.02167364432859	-0.27235456972216	-1.22852197864295
HOXA11	-0.437757974308711	0.312607930998996	-0.228521978642947
WT1	0.147204526412445	1.0495735251652	-0.228521978642947
IFNA2	1.14720452641244	1.0495735251652	0.356440522078209
CACNA2D4	0.884170120578651	1.0495735251652	-1.22852197864295
UTY	1.36959694774889	0.72764543027784	-0.228521978642947
LEP	0.884170120578651	1.0495735251652	1.57883294341466
FGF19	2.14720452641244	-0.27235456972216	1.09340611624442
TLX1	1.7321670271336	0.312607930998996	2.09340611624442
PLA2G3	0.562242025691289	0.72764543027784	2.23090963999435
HPGD	0.147204526412445	-1.27235456972216	2.35644052207821
PPP2R2C	0.147204526412445	-1.27235456972216	0.771478021357053
IL13	-0.437757974308711	0.312607930998996	-0.228521978642947
IFNA7	-1.43775797430871	0.72764543027784	0.356440522078209
EPO	-0.437757974308711	0.72764543027784	0.356440522078209
PAX5	1.14720452641244	1.312607930999	0.771478021357053
PPP3R2	2.14720452641244	1.53500035233544	1.35644052207821
LAMB4	1.88417012057865	-0.27235456972216	0.356440522078209
CSF2	2.26268174383238	-1.27235456972216	-1.22852197864295
FGF5	1.14720452641244	-1.27235456972216	-1.22852197864295
CSF3	0.147204526412445	-1.27235456972216	-1.22852197864295
CREB3L3	-0.437757974308711	-1.27235456972216	0.356440522078209
GRIN1	-0.437757974308711	-0.27235456972216	1.09340611624442
IL12B	-0.437757974308711	-0.27235456972216	1.35644052207821
TCL1B	-0.437757974308711	-1.27235456972216	-0.228521978642947
FGF8	1.14720452641244	-1.27235456972216	-0.228521978642947

**CDH1**	2.14720452641244	4.01304764914009	7.60436803552179
**COL4A5**	4.26268174383238	2.81510827152818	6.86423516227691
**LAMC2**	3.88417012057865	2.0495735251652	6.23090963999435
**BAIAP3**	1.14720452641244	2.89757043172015	7.610681809454
**PROM1**	2.02167364432859	2.312607930999	8.31644245414629
**WNT4**	2.14720452641244	3.53500035233544	5.45797854854027
**MYB**	0.562242025691289	4.08519743489592	6.56589388770716
**PLA1A**	1.88417012057865	4.40007077224934	5.92122514086174
**TMPRSS2**	0.147204526412445	1.89757043172015	7.18510595038123
**IL5RA**	0.884170120578651	1.0495735251652	6.49939847592025
**FGF14**	1.14720452641244	2.42808514841893	6.0568802402193
**CACNG6**	1.7321670271336	3.58562642540541	5.96130258023707
**SFN**	2.02167364432859	2.0495735251652	5.18086895749475
**CACNA2D3**	6.41399106710735	5.03142617845494	5.71399252669629
**GRIA3**	4.05409512202096	4.68184174066471	4.23090963999435
**CSF3R**	3.77169539132024	4.61028847963968	5.31063683246508
**CDK2**	5.40773207663566	3.97557294372143	4.72567433174393
**NPM1**	4.0858039817483	4.65838276784073	4.72567433174393
**FANCF**	4.86602277386839	5.28223428195548	5.92122514086174
**ACVR2A**	5.45705978899923	5.54782439269303	5.56589388770716
**AMER1**	5.6811830984148	5.56053544444258	5.49939847592025
**ENDOG**	5.78141054615345	5.48253293244131	5.29503997741407
**PCNA**	6.34360173921595	5.53500035233544	5.24721145232345
**PRKX**	5.61752446119248	5.65838276784073	5.48572353902318
**CDKN1B**	4.36959694774889	5.13703636641554	5.74875794485697
**MAP3K5**	4.95455944847005	5.1199628530566	5.92122514086174
**PHF6**	4.97163296182899	4.95646412077372	5.980931386986
**LIG4**	5.34360173921595	5.26680424138587	6.00988276068213
**RFC3**	5.14720452641244	5.08519743489592	5.76016270812922
**EFNA1**	5.61752446119248	4.58562642540541	5.16379544413581
**CACNA1C**	6.14720452641244	5.13703636641554	5.59165698377224
**NFATC1**	6.13967085372704	5.20337886124424	5.52636552352052
**CACNB3**	5.57346928111454	4.75001324330629	5.55283773488171
**FANCG**	5.26268174383238	4.77203954963629	5.01940553480064
**PTTG2**	4.23466736766278	5.312607930999	5.82676045685824
**ERCC6**	5.0858039817483	5.08519743489592	5.88000247813522
**PIK3CB**	5.33042635046821	5.1199628530566	5.59165698377224
**HDAC11**	5.00518552154002	5.96605016960292	6.04760242663129
**IL1RAP**	4.62833121614906	4.63453602588636	5.65412107071889
**IL2RB**	3.56224202569129	5.46911241667899	5.42968950410885
**PRKCB**	3.91979403030937	4.91746998915786	4.94140302279937
**IL15**	3.51643833607816	5.9273177751142	4.88000247813522
**MFNG**	4.42022302081886	5.72764543027784	5.09340611624442
**CD40**	4.02167364432859	5.25120738633485	5.71399252669629
**SOCS1**	5.1915986457709	5.08519743489592	5.23090963999435
**FGF1**	5.62833121614906	6.79910779283446	5.57883294341466
**FBXW7**	5.63905762274212	6.53500035233544	5.71399252669629
**SYK**	5.72211336246968	5.42808514841893	5.07525876953416
**IRS1**	7.02576639896247	5.69342971493993	5.53966234613398
**E2F5**	4.97163296182899	2.89757043172015	5.26333111768673
**RFC4**	5.13209763402224	3.97557294372143	4.32606687303469
**PRKAR1B**	4.7321670271336	4.40007077224934	3.94140302279937
**XRCC4**	4.84764424455354	4.01304764914009	4.65412107071889
**HRAS**	4.79106071618717	4.45556588484104	4.65412107071889
**MCM5**	4.29016248025449	4.68184174066471	4.79384583438551
**MAPK12**	3.91979403030937	4.0495735251652	4.0568802402193
**ERCC2**	4.69152504263625	3.72764543027784	4.62945901648462
**ALKBH2**	4.82902856638619	5.18707704891514	4.35644052207821
**GHR**	4.05409512202096	5.40007077224934	4.60436803552179
**LAMA3**	5.1915986457709	4.21949852660751	5.60436803552179

**PRKAR2B**	4.7321670271336	4.93709879590679	5.23090963999435
**BMPR1B**	3.46913262129981	3.48253293244131	6.36393505862513
**MLF1**	5.52802631035338	5.28223428195548	7.15949530670219
**ETV1**	3.26268174383238	5.10268486162476	5.86951010431758
**HELLS**	4.60663614504974	4.312607930999	6.4509581208625
**PRKACB**	5.66027410865181	5.41414595746106	5.11132802424168
**CASP9**	4.31712952785476	5.5090051438025	5.99064654181921
**MLLT3**	5.67076648246946	3.58562642540541	5.18086895749475
**MAPK9**	5.76191437052765	5.21949852660751	5.60436803552179
**MCM4**	5.77169539132024	5.18707704891514	5.26333111768673
**CACNA1D**	5.05409512202096	3.42808514841893	6.37880833510666
**ZAK**	4.82902856638619	4.91746998915786	4.79384583438551
**IDH1**	6.28334121439847	5.59801014986124	5.74875794485697
**POLB**	5.31712952785476	4.87739254978252	5.48572353902318
**GNG4**	8.04404145735704	4.70492535377776	5.29503997741407
**LIF**	4.86602277386839	3.97557294372143	4.980931386986
**FOSL1**	4.1769518698065	2.97557294372143	4.01940553480064
**HMGA1**	5.39513203985603	4.21949852660751	5.29503997741407
**FANCE**	4.75206658457131	4.34235527439305	5.5129450077582
**PDGFB**	4.39513203985603	4.25120738633485	4.44390336332855
**FLNC**	5.7321670271336	5.25120738633485	5.37139086354418
**TNFRSF10D**	5.24874255287451	5.45556588484104	4.83756721181483
**SOX9**	5.76191437052765	5.57313548122221	5.67836861696557
**PPARG**	5.22045350844308	6.14549794516374	5.57883294341466
**CREB5**	4.14720452641244	5.18707704891514	4.94140302279937
**POLR2J**	4.62833121614906	5.03142617845494	4.92122514086174
**HSPA2**	6.14720452641244	4.15391018497994	5.56589388770716
**INHBB**	5.52802631035338	3.85692844722281	6.11132802424168
**HIST1H3B**	7.34360173921595	5.57313548122221	4.32606687303469
**RAC2**	6.67076648246946	6.32755827246497	4.83756721181483
**APH1B**	5.67076648246946	6.01304764914009	5.24721145232345
**NRAS**	5.49297936325418	5.34235527439305	4.79384583438551
**BID**	5.23466736766278	5.34235527439305	4.65412107071889
**PLA2G4C**	5.53952194919121	4.87739254978252	4.70221535891994
**CREBBP**	5.02167364432859	4.83616988705601	4.88000247813522
**SUV39H2**	5.26268174383238	4.5090051438025	5.01940553480064
**BRCA1**	5.20609821546601	4.83616988705601	5.31063683246508
**MUTYH**	4.64970486694163	4.72764543027784	5.03826456205195
**FGF11**	6.13209763402224	4.40007077224934	4.0568802402193
**CDC7**	4.77169539132024	3.93709879590679	4.29503997741407
**WNT2B**	5.52802631035338	4.0495735251652	4.44390336332855
**EZH2**	5.0858039817483	3.93709879590679	4.92122514086174
**CDKN2D**	5.0858039817483	4.63453602588636	4.29503997741407
**H2AFX**	5.53952194919121	4.83616988705601	4.77147802135705
**HHEX**	4.67076648246946	5.38585691302963	5.32606687303469
**PIK3CG**	4.49297936325418	4.79373462073561	5.0568802402193
**PIK3R5**	4.1769518698065	5.63453602588636	4.67836861696557
**GZMB**	1.88417012057865	3.0495735251652	4.0568802402193
**TNF**	1.88417012057865	3.42808514841893	3.01940553480064
**FASLG**	1.14720452641244	1.53500035233544	3.35644052207821
**CD19**	1.36959694774889	3.42808514841893	3.23090963999435
**CCR7**	0.884170120578651	2.72764543027784	2.23090963999435
**CASP12**	1.14720452641244	2.53500035233544	4.12903002597514
**CXXC4**	1.14720452641244	3.312607930999	3.72567433174393
**FLT3**	0.562242025691289	3.48253293244131	2.85894086260739
**PAK3**	0.562242025691289	1.89757043172015	2.77147802135705
**RNF43**	1.7321670271336	1.312607930999	3.90076103830202
**RASGRP1**	2.46913262129981	2.53500035233544	3.62945901648462
**CACNA2D2**	3.0858039817483	2.0495735251652	3.47191773949814
**NPM2**	2.02167364432859	1.53500035233544	2.85894086260739

**LAMB3**	4.29016248025449	3.0495735251652	4.74875794485697
**COL4A6**	1.36959694774889	1.0495735251652	4.65412107071889
**HHIP**	2.26268174383238	2.312607930999	2.47191773949815
**IL20RA**	4.11683087736893	1.72764543027784	4.41533421113178
**PLA2G10**	1.14720452641244	-0.27235456972216	5.67836861696557
**PRKAA2**	1.7321670271336	1.89757043172015	4.980931386986
**PRLR**	2.95455944847005	1.53500035233544	5.27927266155575
**WNT7B**	6.52224395775937	3.312607930999	5.48572353902318
**COL2A1**	6.00518552154002	4.53500035233544	2.77147802135705
**RELN**	4.95455944847005	3.0495735251652	3.72567433174393
**WNT11**	3.31712952785476	2.42808514841893	2.23090963999435
**CSF1R**	5.42022302081886	5.06749543316246	2.94140302279937
**NGF**	4.0858039817483	3.97557294372143	3.67836861696557
**PLA2G4A**	4.34360173921595	2.53500035233544	4.12903002597514
**CALML3**	2.95455944847005	2.81510827152818	4.29503997741407
**PLA2G4F**	3.98850678039339	2.312607930999	4.88000247813522
**ZIC2**	3.64970486694163	1.53500035233544	4.49939847592025
**LEFTY2**	2.64970486694163	1.0495735251652	4.01940553480064
**PLA2G2A**	3.0858039817483	1.53500035233544	3.47191773949814
**TNN**	5.14720452641244	3.63453602588636	3.01940553480064
**FGF13**	4.60663614504974	3.37150162005256	2.85894086260739
**BDNF**	4.11683087736893	1.53500035233544	2.85894086260739
**DKK2**	5.22045350844308	2.81510827152818	2.77147802135705
**PITX2**	3.20609821546601	2.312607930999	2.35644052207821
**CAMK2B**	2.7321670271336	2.81510827152818	3.90076103830202
**WNT10B**	4.20609821546601	3.312607930999	3.81587214071551
**HES5**	3.46913262129981	2.81510827152818	3.72567433174393
**BRIP1**	4.14720452641244	3.37150162005256	3.16379544413581
**SIRT4**	3.91979403030937	3.18707704891514	3.16379544413581
**DTX1**	4.20609821546601	2.53500035233544	2.57883294341466
**CHEK1**	4.23466736766278	2.18707704891514	2.47191773949815
**TTK**	3.56224202569129	2.0495735251652	1.77147802135705
**CCNE1**	4.36959694774889	2.81510827152818	2.35644052207821
**UBE2T**	4.26268174383238	2.63453602588636	2.67836861696557
**FZD2**	3.36959694774889	2.312607930999	3.57883294341466
**CCNA1**	2.7321670271336	2.312607930999	3.52636552352052
**CACNB4**	3.46913262129981	2.42808514841893	3.62945901648462
**FGF12**	3.0858039817483	2.97557294372143	3.29503997741407
**FANCB**	3.84764424455354	2.42808514841893	3.85894086260739
**CDKN2A**	3.26268174383238	3.0495735251652	2.35644052207821
**COL4A4**	2.95455944847005	3.312607930999	2.35644052207821
**CASP10**	2.46913262129981	3.0495735251652	2.85894086260739
**MAPK8IP2**	2.14720452641244	2.97557294372143	2.85894086260739
**IL1R2**	2.56224202569129	3.312607930999	2.77147802135705
**IL2RA**	3.42022302081886	4.01304764914009	2.57883294341466
**SGK2**	3.64970486694163	2.97557294372143	2.94140302279937
**ETV7**	3.02167364432859	3.58562642540541	3.90076103830202
**WNT3**	4.20609821546601	4.5090051438025	3.01940553480064
**CACNA1E**	2.88417012057865	4.25120738633485	4.01940553480064
**FOXL2**	4.77169539132024	4.56053544444258	2.85894086260739
**LAMC3**	6.18429384514767	3.25120738633485	2.35644052207821
**HIST1H3G**	5.83836643096553	3.93709879590679	2.23090963999435
**GLI1**	4.44488507505313	5.0495735251652	4.0568802402193
**CDKN2B**	4.60663614504974	4.68184174066471	2.77147802135705
**CCNA2**	4.56224202569129	4.63453602588636	4.52636552352052
**SPOP**	4.56224202569129	4.65838276784073	3.85894086260739
**BIRC3**	2.26268174383238	3.97557294372143	4.88000247813522
**PPARGC1A**	1.56224202569129	3.72764543027784	4.70221535891994
**FGF10**	0.884170120578651	3.97557294372143	5.19774277605915
**GRIN2A**	2.64970486694163	4.312607930999	4.26333111768673

**ITGA8**	2.26268174383238	4.77203954963629	5.52636552352052
**CHEK2**	3.60663614504974	2.42808514841893	3.94140302279937
**C19orf40**	3.26268174383238	3.312607930999	3.81587214071551
**CDC6**	3.7321670271336	2.53500035233544	2.77147802135705
**EFNA3**	3.60663614504974	3.0495735251652	2.94140302279937
**CREB3L4**	3.84764424455354	4.45556588484104	4.26333111768673
**NASP**	4.29016248025449	4.81510827152818	4.81587214071551
**GNG7**	4.44488507505313	4.81510827152818	4.94140302279937
**MAPK8IP1**	2.46913262129981	3.68184174066471	5.62945901648462
**SHC4**	3.20609821546601	4.72764543027784	5.19774277605915
**ARNT2**	2.95455944847005	3.72764543027784	3.90076103830202
**RPS6KA6**	3.69152504263625	3.25120738633485	4.41533421113178
**DUSP2**	3.02167364432859	3.312607930999	4.41533421113178
**IRAK2**	3.77169539132024	4.56053544444258	4.12903002597514
**DUSP10**	3.42022302081886	3.25120738633485	4.62945901648462
**MAPK10**	4.71198914519597	4.18707704891514	5.71399252669629
**DUSP4**	5.05409512202096	3.97557294372143	4.65412107071889
**FGF9**	4.44488507505313	4.89757043172015	5.42968950410885
**DUSP8**	4.69152504263625	3.93709879590679	4.77147802135705
**SOX17**	3.69152504263625	4.68184174066471	3.81587214071551
**IL6R**	3.60663614504974	3.312607930999	4.92122514086174
**IL3RA**	4.36959694774889	3.25120738633485	3.72567433174393
**MCM2**	5.0858039817483	4.75001324330629	4.35644052207821
**CEBPA**	4.67076648246946	4.53500035233544	4.09340611624442
**CACNB2**	3.64970486694163	4.93709879590679	4.38618786547226
**ACVR1B**	4.23466736766278	4.70492535377776	5.49939847592025
**NTRK1**	3.26268174383238	2.89757043172015	3.35644052207821
**BRCA2**	3.46913262129981	2.89757043172015	3.57883294341466
**TNFRSF10A**	2.46913262129981	2.0495735251652	3.47191773949814
**FGF17**	3.26268174383238	2.0495735251652	3.67836861696557
**CARD11**	2.64970486694163	2.18707704891514	4.19774277605915
**TNFRSF10C**	2.46913262129981	3.68184174066471	2.94140302279937
**ITGB7**	3.14720452641244	3.63453602588636	4.47191773949814
**NFKB1**	4.31712952785476	3.97557294372143	4.35644052207821
**CCNB1**	4.97163296182899	3.58562642540541	4.35644052207821
**MMP3**	5.23466736766278	1.312607930999	1.09340611624442
**IL6**	2.14720452641244	1.72764543027784	0.356440522078209
**IL1B**	-1.43775797430871	1.312607930999	1.77147802135705
**IFNA17**	1.7321670271336	1.89757043172015	1.09340611624442
**FGF23**	2.14720452641244	3.89757043172015	1.35644052207821
**FANCA**	3.14720452641244	1.72764543027784	0.356440522078209
**CCNB3**	2.02167364432859	0.72764543027784	2.94140302279937
**PRMT8**	2.26268174383238	0.312607930998996	3.29503997741407
**CBLC**	1.88417012057865	1.312607930999	3.57883294341466
**IL12A**	2.36959694774889	2.0495735251652	2.94140302279937
**IL10**	1.36959694774889	1.0495735251652	0.771478021357053
**CRLF2**	0.562242025691289	2.81510827152818	1.77147802135705
**BCL2A1**	1.36959694774889	2.53500035233544	2.35644052207821
**PLA2G5**	1.7321670271336	2.42808514841893	1.57883294341466
**IL12RB2**	1.88417012057865	2.81510827152818	1.94140302279937
**COL4A3**	1.14720452641244	1.89757043172015	3.23090963999435
**CALML6**	1.88417012057865	1.53500035233544	2.94140302279937
**FIGF**	2.02167364432859	1.89757043172015	1.94140302279937
**RET**	2.02167364432859	1.0495735251652	1.94140302279937
**EGF**	2.46913262129981	0.312607930998996	1.94140302279937
**PTPN5**	1.36959694774889	1.312607930999	2.23090963999435
**PLA2G4E**	1.88417012057865	1.0495735251652	2.23090963999435
**MPL**	2.46913262129981	0.72764543027784	0.771478021357053
**ZBTB32**	2.02167364432859	-0.27235456972216	1.09340611624442
**EFNA2**	1.14720452641244	-0.27235456972216	0.771478021357053

**DLL3**	-0.437757974308711	0.312607930998996	0.771478021357053
**CCNO**	0.884170120578651	1.89757043172015	1.77147802135705
**COL6A6**	0.884170120578651	1.0495735251652	2.23090963999435
**IL23A**	1.36959694774889	1.89757043172015	1.77147802135705
**PAK7**	0.562242025691289	2.18707704891514	1.35644052207821
**CEBPE**	2.46913262129981	1.89757043172015	0.771478021357053
**IL24**	2.26268174383238	1.0495735251652	0.356440522078209
**CDC25C**	2.02167364432859	1.0495735251652	0.771478021357053
**PKMYT1**	3.26268174383238	2.18707704891514	1.94140302279937
**PCK1**	4.29016248025449	2.42808514841893	0.771478021357053
**IL11**	3.56224202569129	1.312607930999	1.35644052207821
**CACNG4**	4.26268174383238	1.53500035233544	2.09340611624442
**IL7**	3.02167364432859	2.18707704891514	2.94140302279937
**BIRC7**	3.31712952785476	2.0495735251652	1.77147802135705
**RPA3**	2.7321670271336	1.72764543027784	2.77147802135705
**FZD10**	2.46913262129981	2.53500035233544	2.35644052207821
**NTF3**	2.95455944847005	2.97557294372143	3.29503997741407
**MAPT**	2.02167364432859	1.72764543027784	2.47191773949815
**FGF18**	2.88417012057865	2.18707704891514	3.09340611624442
**POLD1**	3.84764424455354	3.312607930999	2.67836861696557
**POLE2**	3.81016953913487	2.81510827152818	2.94140302279937
**FEN1**	3.60663614504974	2.63453602588636	2.94140302279937
**NOG**	3.31712952785476	4.56053544444258	3.01940553480064
**FGFR4**	2.46913262129981	2.0495735251652	3.77147802135705
**FZD9**	2.46913262129981	2.72764543027784	2.57883294341466
**AMH**	2.46913262129981	3.1199628530566	2.77147802135705
**TSLP**	1.88417012057865	2.42808514841893	2.67836861696557
**ETV4**	2.14720452641244	2.42808514841893	2.77147802135705
**RXRG**	1.7321670271336	3.1199628530566	2.35644052207821
**TPO**	3.20609821546601	2.312607930999	2.67836861696557
**MYCN**	2.26268174383238	1.72764543027784	2.35644052207821
**CDC25A**	1.88417012057865	1.72764543027784	3.29503997741407
**WNT6**	2.26268174383238	1.72764543027784	2.77147802135705
**FGF22**	1.7321670271336	1.89757043172015	3.01940553480064
**ITGB6**	1.88417012057865	0.72764543027784	4.09340611624442
**IL22RA1**	0.884170120578651	0.72764543027784	4.19774277605915
**WNT10A**	1.36959694774889	1.72764543027784	2.57883294341466
**CCNE2**	3.20609821546601	1.72764543027784	2.23090963999435
**IL20RB**	3.14720452641244	1.53500035233544	2.09340611624442
**HOXA10**	2.26268174383238	2.18707704891514	2.47191773949815
**TNR**	2.26268174383238	2.312607930999	1.94140302279937
**GDF6**	0.884170120578651	1.72764543027784	3.29503997741407
**LRP2**	1.88417012057865	1.72764543027784	3.57883294341466
**OSM**	0.147204526412445	2.0495735251652	2.09340611624442
**PTPRR**	1.88417012057865	0.72764543027784	1.94140302279937
**PAX8**	2.56224202569129	0.312607930998996	1.35644052207821
**DKK4**	0.884170120578651	1.0495735251652	1.57883294341466
**FGF21**	2.02167364432859	1.72764543027784	1.35644052207821
**HOXA9**	2.88417012057865	2.0495735251652	2.57883294341466
**HMGA2**	2.36959694774889	1.89757043172015	2.35644052207821
**FGF3**	2.02167364432859	1.53500035233544	0.356440522078209
**IL3**	0.884170120578651	1.312607930999	0.356440522078209
**FGF4**	2.02167364432859	0.312607930998996	0.356440522078209
**WNT7A**	2.02167364432859	2.0495735251652	0.771478021357053
**MMP7**	1.7321670271336	2.0495735251652	1.35644052207821
**FGF20**	2.02167364432859	1.312607930999	1.94140302279937
**ACVR1C**	2.64970486694163	2.312607930999	2.23090963999435
**E2F1**	3.60663614504974	1.72764543027784	1.57883294341466
**SHC3**	2.26268174383238	2.312607930999	2.23090963999435
**COL1A1**	17.2554464985422	14.5516594291511	12.1301292222499

**COL3A1**	14.0835357374366	13.5709776664954	12.3451252088504
**COL1A2**	15.464793791666	12.7125978580102	10.7820061272435
**B2M**	12.3883932404915	12.8624727999109	13.529336767948
**RPS27A**	12.1745346198008	12.7151759254421	12.3867628047222
**SPP1**	14.2702481545874	12.8920810163425	11.74947338997
**SFRP2**	5.22045350844308	9.94802375797307	9.6866104703077
**WIF1**	6.00518552154002	10.6756475612555	10.058035782965
**SFRP4**	6.40773207663566	8.70492535377776	8.63101280773971
**LEPR**	6.9065379336071	10.0461882399806	8.82404607216121
**NR4A1**	5.11683087736893	8.24924587000157	9.18086895749476
**SETBP1**	8.66945910128267	7.26680424138587	8.6723448293378
**COL27A1**	7.94378497687587	7.77203954963629	7.12903002597514
**IL11RA**	7.32711361642738	8.04505804404271	7.74014481455226
**PBX1**	7.62023374845046	8.24531481841165	9.18404786816226
**DDIT4**	9.00518552154002	10.6182897138969	9.76229110051066
**MYC**	9.13209763402224	9.0393937452828	8.0568802402193
**NFKBIA**	9.21598280439786	10.0234143646983	10.059767363538
**SOST**	9.41399106710735	11.2485096132666	10.6317890762765
**PIK3R1**	7.32046524041801	9.9938463427768	9.86026626007396
**PML**	8.57067064776187	8.67015993561708	8.93386935011396
**HSPB1**	7.9175931221161	8.71916727635353	9.33562751134279
**AKT2**	8.74587740716451	8.62397783418778	9.23602377169099
**GNA11**	8.62833121614906	8.5253069561316	8.93889616718879
**ATRX**	8.67598419174048	8.56842335387289	8.83487310264556
**IGF1R**	8.49887996469386	8.50736478542125	9.88652167151876
**SF3B1**	8.63638348844379	8.24531481841165	9.30966695340947
**CCND1**	9.16494089070925	7.88751676705623	8.75161759899621
**JAK1**	8.38401600766186	8.44189094794396	8.50109876491009
**NCOR1**	8.24874255287451	8.03598446041725	8.85761424638436
**BAP1**	7.97586995471546	7.62246319358578	8.25327745302281
**SMARCB1**	7.83370505359566	8.03370711970618	8.66781042526699
**SRSF2**	8.15469906295937	7.85692844722281	8.43325611912904
**VEGFA**	8.20970045214621	7.60722867989062	8.87738652992821
**ETS2**	8.17511052298233	8.45045296144739	8.98822387955236
**H3F3C**	8.83370505359566	9.69270818702247	9.86159044102134
**SKP1**	9.50475653103053	9.30034565676513	9.5053407410356
**ID1**	9.8066058608118	8.57783226792361	8.93764110400317
**PDGFRA**	9.57626249600622	8.92854403531629	9.04760242663129
**STAT3**	9.33455659961294	9.21749339071714	8.80765163391054
**SHC1**	9.18612351570475	9.60799423843387	8.81860193347108
**NOTCH2**	9.47961410516813	9.81444511590129	9.10239489947167
**AKT1**	9.5720706430594	8.86591723045006	8.95014287236353
**U2AF1**	8.86716369527296	8.56211648026206	9.08549472525841
**PDGFRB**	8.27476902613111	8.90631028128431	8.96253723588871
**CAPN2**	8.66421769664052	8.85692844722281	9.44301908806894
**NOTCH3**	8.95025931103642	9.89882222792961	9.06954058907607
**PLAT**	8.69152504263626	9.71063900497215	9.05456637438106
**TGFBR2**	8.48408296276578	9.66869303661842	9.18086895749476
**FGFR3**	10.5233299156886	9.24629858595123	8.84159896583388
**COL5A2**	11.2792757751822	9.02111707911597	7.666295784665
**IGFBP3**	8.33373149519189	9.26194831273247	8.20610624899378
**LIFR**	10.347285740471	10.5301617953991	9.48572353902318
**GNAS**	10.5153471762826	9.91437871940671	9.44567028950274
**FLNA**	10.1349422521786	10.8694332719488	9.93512769737288
**FN1**	11.1681844653167	11.2376677890916	9.94015013948928
**HSPA1A**	6.84301279582189	7.69631222347305	8.3658026252819
**GADD45B**	8.36959694774889	9.04052838556219	8.66781042526699
**FOS**	8.98640831450939	8.80312457976586	11.2645829138962
**UBB**	10.4691326212998	10.4787710136451	11.186955729055
**CTNNB1**	11.1185085692743	10.5656674885824	10.7448166421574

**H3F3A**	11.2375375247853	10.4119557414022	10.9854935127679
**TGFB1**	10.9217170745408	10.3773506957493	9.49257721006424
**FGFR1**	10.9919102908244	10.9844438163572	9.94640370385773
**MMP9**	12.3878964959902	9.20337886124424	7.47538159480172
**THBS1**	11.0425273466277	10.8014522774563	9.29895502741745
**COL11A1**	10.7384151747988	9.55816063725468	8.29307846108078
**COL5A1**	13.0675573818275	10.7727224822128	9.26733304824422
**CHAD**	13.2594268089158	11.5036671455307	10.2984662267891
**IL8**	1.88417012057865	3.48253293244131	4.35644052207821
**PAX3**	2.36959694774889	6.7219988671367	7.66022127025531
**ZBTB16**	4.05409512202096	10.2614618197112	6.78270527678031
**WEE1**	5.14720452641244	7.73326997947172	8.36953052151865
**EYA1**	6.11683087736893	6.67015993561708	7.70221535891994
**ITGA6**	6.40144581378823	7.56999577369165	7.61382836477086
**SPRY1**	6.00518552154002	7.50243248987901	7.82404607216121
**TNFSF10**	4.79106071618717	6.45556588484104	8.82404607216121
**EGFR**	5.49297936325418	7.25902689079415	8.50449334304302
**ITGB4**	5.35665789204139	7.77203954963629	8.5213474487539
**NR4A3**	3.64970486694163	6.09396764452366	6.59802650864797
**FGF7**	4.49297936325418	8.24728168312105	7.52636552352052
**PLCB1**	5.1769518698065	6.25120738633485	6.44390336332855
**MAP3K14**	5.24874255287451	6.01304764914009	6.68436735758702
**COMP**	3.7321670271336	6.19525098036084	5.14651745270398
**CACNA1G**	6.42642817034557	5.89757043172015	5.97115036619342
**PLCE1**	5.36959694774889	5.1199628530566	6.79938401792694
**FGF2**	4.34360173921595	5.44189094794396	5.666295784665
**EFNA5**	4.02167364432859	7.32010246754592	5.78270527678031
**AR**	5.48110526296588	6.9273177751142	6.48572353902318
**RASGRP2**	3.91979403030937	6.80979447163171	6.52636552352052
**BMP5**	2.64970486694163	6.73326997947172	6.17235745763924
**CNTFR**	3.20609821546601	6.50243248987901	6.12903002597514
**ANGPT1**	6.10913648557892	7.07637358450892	6.62945901648462
**SFRP1**	5.83836643096553	7.54462905353322	7.94140302279937
**NOTCH1**	6.26960115777217	6.34969724973422	6.73147995342513
**IKBKB**	6.63370438824791	6.48919666272232	7.36766777750146
**TSPAN7**	4.29016248025449	7.54462905353322	6.57237792127736
**ID4**	4.23466736766278	7.72764543027784	6.82132657080762
**NTRK2**	4.51643833607816	6.79910779283446	7.01465200483
**KDM6A**	6.89763238038521	7.44189094794396	7.16808280253891
**MAP2K1**	7.32046524041801	7.01304764914009	6.67836861696557
**DUSP6**	6.52802631035338	6.70492535377776	7.18510595038123
**MAPK1**	6.57905031337784	6.46235505050368	7.00988276068213
**TP53**	7.71198914519597	7.17472865648749	6.78270527678031
**RB1**	7.27648754335741	6.93709879590679	7.03826456205196
**GSK3B**	6.87512498097564	6.71063900497215	6.95138711137199
**TCF7L1**	6.27648754335741	7.45215928339779	7.18086895749476
**CDKN1C**	6.78624369988939	7.17472865648749	7.85894086260739
**MAP3K1**	6.51643833607816	6.79910779283446	7.48572353902318
**TIAM1**	7.18795086875575	8.49913489977844	7.45447260503874
**MYD88**	7.79106071618717	7.46573768989833	6.91102937375585
**SMARCA4**	7.67858598692876	6.57313548122221	7.05224879148766
**DAXX**	6.69666834591222	6.71633011705001	6.73726230601914
**PLCB4**	6.62293795737884	5.61028847963968	7.65106127096984
**PIK3R3**	5.78141054615345	5.38585691302963	6.66022127025531
**SKP2**	6.64439106704516	6.81510827152818	7.08436097664141
**TSC1**	6.35014458508272	6.37150162005256	6.98579714215782
**TET2**	7.05409512202096	6.72764543027784	7.59165698377224
**DNMT3A**	7.13209763402224	6.11134972275189	6.53302925380153
**SP1**	7.24874255287451	7.38585691302964	7.08436097664141
**NSD1**	7.10913648557893	7.29369946844893	6.97604916560626

**XPA**	6.85686277458291	6.66428336928041	6.85894086260739
**MDC1**	6.46913262129981	6.69918898422861	6.53966234613398
**CHUK**	6.62293795737884	6.37150162005256	6.666295784665
**TNFRSF10B**	7.01756924599585	6.34969724973422	6.26333111768673
**KAT2B**	6.3887905129822	6.62851223825859	6.83756721181483
**PPP3CC**	6.0858039817483	6.37150162005256	6.86423516227691
**PIK3CA**	6.81490745814154	7.03142617845494	7.13342179509229
**FZD3**	6.7321670271336	6.83093323868986	6.83217395304461
**POLD4**	6.81962986838394	6.08519743489592	6.91613626418894
**NTHL1**	6.33042635046821	6.57313548122221	6.74302157530783
**DNMT1**	6.64439106704516	6.18707704891514	6.71399252669629
**MTOR**	6.32379325813577	6.15391018497994	6.76016270812922
**JAK3**	6.30370901209244	5.20337886124424	5.78270527678031
**SHC2**	5.88417012057865	5.41414595746106	5.91102937375585
**MAP3K12**	5.57346928111454	5.96605016960292	6.41533421113178
**DDB2**	6.30370901209244	5.58562642540541	6.4509581208625
**THEM4**	5.57346928111454	5.70492535377776	5.980931386986
**PIK3CD**	5.63905762274212	5.86719678267663	6.47883715343794
**EPOR**	5.64970486694163	6.14549794516374	5.88000247813522
**FANCC**	6.06208791277449	5.72764543027784	5.82676045685824
**DLL4**	4.84764424455354	5.37150162005256	5.41533421113178
**KIT**	5.44488507505313	5.58562642540541	6.3489068493928
**PLD1**	5.69152504263625	5.87739254978252	6.29503997741407
**RAC3**	6.01345313752362	5.41414595746106	5.38618786547226
**PBX3**	5.43260674527469	6.18707704891514	5.8049010228945
**IGF1**	5.79106071618717	7.25902689079415	6.03826456205195
**DTX3**	6.46913262129981	6.40712552978329	6.610681809454
**CBL**	6.56224202569129	6.0495735251652	6.23090963999435
**COL24A1**	8.164012814099	5.70492535377776	5.84829361840788
**FUT8**	7.10527384594653	6.48919666272232	6.29503997741407
**STAG2**	7.24172212519673	6.82567751323837	7.17235745763924
**HIST1H3H**	7.38560926573752	6.69918898422861	6.51967087094651
**FZD8**	7.21687805421926	7.5573681653639	6.74875794485697
**NUPR1**	7.29016248025449	6.10268486162476	5.74875794485697
**EIF4EBP1**	7.06606776368704	5.80446102732867	5.99064654181921
**PTCH1**	6.83370505359566	6.49582975505477	6.53302925380153
**PBRM1**	6.52224395775937	6.57313548122221	6.45797854854027
**PPP3CA**	7.51060925727597	6.94681395074	6.20610624899378
**KRAS**	7.10913648557893	6.73326997947172	6.08436097664141
**SPRY4**	6.02984757577429	6.52854533019814	5.83756721181483
**FOXO4**	6.1769518698065	6.49582975505477	6.60436803552179
**TCF3**	6.57905031337784	6.61028847963968	6.36393505862513
**IL7R**	7.82433687106147	5.46911241667899	6.65412107071889
**CDC14A**	6.26268174383238	5.86719678267663	7.43325611912904
**BCOR**	6.11683087736893	6.01304764914009	5.76016270812922
**BNIP3**	6.82902856638619	5.87739254978252	6.54626508095823
**VEGFC**	6.5452356003856	6.25902689079415	6.18086895749475
**SIN3A**	6.13209763402224	6.48919666272232	6.4081026419007
**MSH6**	6.93292943249851	6.72764543027784	6.71984525294173
**CDC14B**	6.85686277458291	6.75001324330629	6.00029671185293
**ATM**	6.83836643096553	7.23544007047654	5.8049010228945
**PRKCA**	7.14344260761625	6.97081941375079	5.70221535891994
**ARID1A**	6.61752446119248	6.44874461898502	6.56589388770716
**MAP2K4**	6.69152504263626	6.58562642540541	6.610681809454
**CDC25B**	6.23466736766278	6.53500035233544	5.980931386986
**SMO**	6.43875897225629	5.85692844722281	5.99064654181921
**POLR2D**	6.62293795737884	6.41414595746106	6.22268913318938
**DTX4**	6.43875897225629	5.78292786577903	6.65412107071889
**PDGFA**	6.91097017992237	5.88751676705623	5.666295784665
**MAD2L2**	6.87512498097564	5.67015993561708	5.38618786547226

**GLI3**	7.63905762274212	6.42113238777717	6.06609877024868
**CDK6**	7.03391724008333	5.93709879590679	6.01940553480064
**HDAC4**	6.63905762274212	6.39298134746302	6.19774277605915
**SMAD9**	7.35014458508272	6.312607930999	6.20610624899378
**FLT1**	5.96312146197347	5.75001324330629	5.88000247813522
**ITGA2**	6.49887996469386	6.58562642540541	6.70221535891994
**MAML2**	6.03797545665769	6.49582975505477	6.341333629688
**STMN1**	6.44488507505313	5.49582975505477	5.980931386986
**CDKN2C**	5.91097017992237	5.95646412077372	6.341333629688
**TLR4**	4.98850678039339	5.91746998915786	5.64184274094046
**NOS3**	5.02167364432859	5.57313548122221	5.12903002597514
**MAP2K6**	5.88417012057865	5.75001324330629	5.81587214071551
**APC**	5.66027410865181	5.89757043172015	5.41533421113178
**RIN1**	5.55092671246345	5.06749543316246	5.86951010431758
**MAP3K13**	5.1769518698065	5.18707704891514	6.23908357144005
**LEF1**	7.62023374845046	5.99443197097274	5.29503997741407
**RASGRF2**	5.56224202569129	5.94681395074	5.07525876953416
**CDK4**	6.62293795737884	6.37869712145677	5.84829361840788
**GTF2H3**	6.18429384514767	5.84658650300135	5.83756721181483
**WNT5B**	6.92418579942653	6.2114612075421	6.00988276068213
**MED12**	6.07003666588998	5.98503327297049	6.26333111768673
**MAPK8**	6.31043487528075	5.85692844722281	5.99064654181921
**FAS**	6.31712952785476	6.39298134746302	6.12020617558813
**RAD50**	5.9285642399371	6.23544007047654	6.25529379862131
**MNAT1**	5.52802631035338	5.96605016960292	6.10239489947167
**CASP3**	5.89315890380591	5.5090051438025	6.24721145232345
**ALKBH3**	5.40773207663566	5.75001324330629	6.24721145232345
**CASP7**	5.64970486694163	5.23544007047654	6.50618764158289
**BMP6**	5.31712952785476	5.81510827152818	6.03826456205195
**RRAS2**	4.97163296182899	6.13703636641554	6.2871778596411
**GADD45G**	4.26268174383238	5.99443197097274	6.29503997741407
**HSPA6**	4.86602277386839	6.30507425831359	6.23090963999435
**TLR2**	5.33042635046821	6.29750103860879	5.76016270812922
**SIX1**	5.61752446119248	5.81510827152818	6.99064654181922
**AXIN1**	5.98009454057719	6.26680424138587	6.69034125863165
**TGFB2**	5.40773207663566	7.93221657452704	7.18933053624295
**DKK1**	5.59566502722874	7.33497574402745	7.07525876953416
**TNC**	5.93728145703821	6.74445371796439	6.09340611624442
**PGF**	5.24874255287451	6.6163886791761	5.71399252669629
**PRDM1**	5.49297936325418	5.06749543316246	6.21442151720578
**GATA2**	3.26268174383238	5.32755827246497	7.08436097664141
**STAT4**	3.64970486694163	4.81510827152818	6.07525876953416
**DLL1**	4.36959694774889	5.10268486162476	6.4509581208625
**MET**	4.39513203985603	5.84658650300135	6.84829361840788
**CCND2**	4.64970486694163	6.34235527439305	6.32606687303469
**MECOM**	4.42022302081886	4.85692844722281	6.9662348757793
**ITGA3**	4.90209202857591	5.49582975505477	6.97604916560626
**RPS6KA5**	4.84764424455354	5.79373462073561	7.03357286672723
**ITGB8**	4.29016248025449	4.63453602588636	7.22680524166161
**CACNA1H**	3.64970486694163	4.97557294372143	6.01940553480064
**BCL2**	5.00518552154002	6.34969724973422	6.99547969555516
**MAP3K8**	4.14720452641244	5.38585691302963	7.01465200483
**ITGA7**	5.43260674527469	6.35700205035745	6.341333629688
**IRAK3**	5.53952194919121	6.57313548122221	6.38618786547226
**ITGA9**	5.1915986457709	7.03142617845494	6.13780023560287
**LAT**	5.63905762274212	6.09396764452366	6.59165698377224
**WHSC1**	6.00518552154002	5.68184174066471	5.97115036619342
**MSH2**	6.0936234862076	6.53500035233544	6.82132657080762
**FANCL**	5.76191437052765	6.13703636641554	6.77710257055093
**PLCG2**	5.22045350844308	6.11134972275189	6.50618764158289

**HGF**	4.23466736766278	5.65838276784073	6.20610624899378
**LFNG**	5.27648754335741	4.77203954963629	6.73147995342513
**BAMBI**	10.1714207638333	9.02226617916947	7.82404607216121
**BMP8A**	8.85456365849333	9.25022696153267	7.43325611912904
**ITGB3**	8.63370438824791	7.82040257119769	6.2871778596411
**WNT5A**	7.70179337809008	6.37150162005256	7.47538159480172
**NKD1**	7.86830371511963	7.08079225577592	6.59165698377224
**INHBA**	6.58460983871974	6.24334526856188	5.53966234613398
**BMP2**	7.81727059551002	6.95164710447594	5.74875794485697
**THBS4**	6.78141054615345	4.28223428195548	5.00029671185293
**PLAU**	7.24523660937297	4.95646412077372	3.980931386986
**TRAF7**	8.5873815879698	7.4589644613029	8.03122128504783
**MCM7**	8.31378608478039	8.01535780982729	8.10909992334956
**CIC**	7.68894649853448	7.47918948936694	7.67836861696557
**HDAC5**	8.34031915522665	7.9273177751142	7.87476582976907
**ARID2**	7.83836643096553	7.40007077224934	8.10909992334956
**NF2**	6.8705810558307	7.33867022758519	7.63566416601133
**JAK2**	7.41711040895152	7.88751676705623	8.53966234613398
**MLLT4**	6.81016953913487	7.34235527439305	8.83487310264556
**CYLD**	7.6310203036767	7.3163600658601	7.85096280518387
**KMT2D**	7.80541600916424	7.47247926777739	7.87476582976907
**KDM5C**	7.98850678039339	7.44874461898503	8.4117229575794
**WHSC1L1**	7.76925634586882	7.63152727601402	8.31063683246508
**KMT2C**	7.78141054615345	7.94196455107861	8.11132802424168
**PDGFD**	9.88754748078097	9.03370711970618	8.13998448286475
**SMAD3**	10.1004309577437	9.36245648044956	8.6798706421308
**RHOA**	9.55376387176698	9.56053544444258	8.63256492735245
**LTBP1**	9.17879086947028	9.90879768714341	9.4465529417425
**TBL1XR1**	8.51643833607816	8.85305890076116	8.84829361840788
**PTEN**	9.11106393415004	9.18399984538613	8.60120075644311
**SMAD2**	8.71198914519597	8.67601266186252	8.64338325901624
**PRKDC**	8.23289827480973	7.89003675903475	8.27331520625935
**RBX1**	8.39986995886269	8.49251702101393	8.66022127025531
**EPHA2**	9.10624047582583	7.92486212338789	7.74302157530783
**SMAD4**	8.15282907560632	7.95886661098903	8.0591904009065
**MAP2K2**	8.57346928111454	7.86719678267663	8.13342179509229
**PPP3R1**	8.7582293237198	8.76519238424002	8.16164697755724
**BCL2L1**	8.30370901209244	7.97081941375079	8.43858956343208
**GNG12**	7.60936593780531	8.33127177526403	8.45447260503874
**AKT3**	7.4540257289096	8.79104051156635	8.4874400116122
**MDM2**	7.99687025332801	8.10051049039043	8.2431532357491
**SOCS2**	8.19341908139527	8.39120353449511	8.39718686442152
**GNAQ**	8.1695723394409	8.52854533019815	8.42252971253598
**RAD21**	8.47961410516813	8.75278499255635	8.55283773488171
**IBSP**	11.4549745583031	9.06972009827698	7.76016270812922
**CREB3L1**	11.4032165955132	8.11134972275189	8.5129450077582
**COL11A2**	9.80303335785324	7.26292080689864	6.67836861696557
**SOCS3**	8.1415579632713	6.41414595746106	6.10239489947167
**SPRY2**	6.88417012057865	7.30129261777116	7.79661758363556
**DUSP5**	5.81962986838394	6.32010246754592	6.74875794485697
**NFKBIZ**	6.07794186397533	7.59183157493212	7.5129450077582
**TNFAIP3**	5.96312146197347	7.13703636641554	8.67836861696557
**RUNX1**	6.23466736766278	7.19116980354902	8.11577392927287
**LAMA5**	5.89315890380591	6.79910779283446	7.79106874971493
**RUNX1T1**	6.39513203985603	7.23147116827359	7.50618764158289
**CACNA2D1**	7.31043487528075	6.67015993561708	7.1766194844934
**FGFR2**	7.32379325813577	7.20337886124424	7.29895502741745
**IKBKG**	6.88417012057865	6.48253293244131	6.99064654181922
**SOS1**	7.44488507505313	7.14127335930201	7.62945901648463
**HDAC10**	6.11683087736893	6.73326997947172	7.31063683246508

**CASP8**	7.01345313752362	6.97557294372143	7.71692185773496
**PPP3CB**	7.24523660937297	7.21949852660751	7.23090963999435
**TFDP1**	7.50475653103053	7.87230367310972	7.83756721181483
**TGFB3**	7.69666834591222	7.8230424530704	6.76016270812922
**ERBB2**	6.71198914519597	7.05407491740014	8.45797854854027
**NGFR**	6.24874255287451	6.99443197097274	7.23500239462823
**MAPK3**	7.26268174383238	7.65245793388362	7.64184274094046
**HDAC1**	7.1584317818357	7.27453989016548	7.64799496792205
**SMC1A**	6.95455944847005	6.68184174066471	7.42968950410885
**MLH1**	6.89763238038521	6.88246353932994	6.97115036619342
**STK11**	7.27648754335741	7.35335427334231	6.97115036619342
**NUMBL**	7.22402012346328	7.13278689341418	7.33753205952815
**NBN**	7.45705978899923	7.61028847963968	7.5163118588566
**VHL**	7.60116101498359	7.75278499255635	7.13780023560287
**ATR**	6.6811830984148	6.08519743489592	7.16808280253891
**POLR2H**	6.95455944847005	6.53500035233544	6.93638494803274
**HDAC6**	6.70690026852317	6.61028847963968	7.3489068493928
**RAD52**	6.64970486694163	6.48919666272232	7.18933053624295
**JAG2**	7.6417268095181	6.44189094794396	7.57883294341466
**IDH2**	7.85456365849333	7.11566271562298	7.47883715343794
**GRB2**	7.5395219491912	6.76106843181529	7.29111427420027
**PIK3R2**	7.59014802226117	6.48919666272232	7.04294104926143
**BAD**	7.56224202569129	6.90257111277852	7.13780023560287
**BMP4**	8.11106393415004	7.08519743489592	7.29503997741407
**AXIN2**	6.73716770819197	6.79373462073561	7.6723448293378
**FUBP1**	7.39829238074936	6.98974027564802	7.42611404988502
**KITLG**	6.61752446119248	8.33682416841982	8.30480775366289
**STAT1**	7.4540257289096	7.81775784994213	8.16164697755724
**NFE2L2**	7.60663614504974	7.98503327297049	8.09115014230405
**HDAC2**	7.96738348882763	8.01304764914009	7.39718686442152
**RAC1**	8.23643429383697	8.62700235320095	7.72567433174393
**JAG1**	6.9065379336071	7.89506357610958	7.27132390844026
**GPC4**	7.37602321690833	7.7219988671367	6.96130258023707
**RELA**	7.40773207663566	7.52206129662795	7.07068603974433
**CCND3**	7.01756924599585	7.67015993561708	6.93134935813544
**CD14**	7.42952076540095	7.52206129662795	6.17235745763924
**HSP90B1**	8.5636502200841	7.35700205035745	7.341333629688
**IL1R1**	9.05609747493211	7.50243248987901	6.6723448293378
**FST**	6.39513203985603	8.24137302623028	7.12903002597514
**GADD45A**	5.97163296182899	8.4315490037225	7.31450984161229
**FZD7**	7.23819905863304	8.08299652670265	7.14216542816427
**GAS1**	7.99478392607955	8.66281047988154	7.4117229575794
**HES1**	6.11683087736893	6.01304764914009	7.22680524166161
**PIM1**	7.33373149519189	6.16227365791456	6.35644052207821
**ABL1**	7.37602321690833	7.08079225577592	7.40083464143666
**DDIT3**	6.9065379336071	5.89757043172015	6.93134935813544
**MEN1**	7.65235444535558	7.01304764914009	7.06149686828967
**ASXL1**	7.3887905129822	6.90755452029277	7.18086895749476
**BMP7**	7.53378557964206	7.01766427721046	7.50957028097754
**KLF4**	7.65235444535558	8.08958920401308	7.79661758363556
**JUN**	6.53378557964206	7.30129261777116	7.74014481455226
**ID2**	8.27304845939064	6.68764736234592	7.48915444442345
**CDKN1A**	7.96950978993602	7.79104051156635	6.36393505862513
**PTPN11**	7.76191437052765	7.52206129662795	7.15949530670219
**RASA4**	7.89091695301924	7.96126510703754	7.495991874477
**MGMT**	7.40773207663566	7.66428336928041	7.85362706271092
**BAX**	8.07201703001823	7.28988785449891	7.00988276068213
**PRKACA**	8.26268174383238	7.69342971493993	7.88782198259452
**NF1**	7.5076858620692	7.56999577369165	8.23704442616645
**SMC3**	7.12066273895995	7.28988785449891	7.73726230601914

**CUL1**	7.21687805421926	7.4589644613029	7.52302208044615
**PRKAR2A**	8.04202228972039	7.34969724973422	7.51967087094651
**RAF1**	7.71958896105413	7.46911241667899	7.60120075644311
**ARID1B**	7.71706013474339	7.61334180361723	7.85362706271092
**SOS2**	7.78865421848007	7.98503327297049	7.30675339797786
**EP300**	7.65763904848385	7.98031086272809	7.95138711137199
**SETD2**	7.57067064776187	7.26292080689864	7.83756721181483
**BRAF**	7.21687805421926	7.3088460122028	7.71692185773496
**PDGFC**	8.23289827480973	7.68184174066471	7.58846164461243
**PPP2CB**	8.25049233482447	8.76793515130356	8.15734042199851
**PPP2R1A**	8.41711040895152	8.34051392756888	8.09340611624441

8915-Osteome	8916-Osteome	9184-Osteome	8917-Osteome
0.249476805378731	-1.06118197805988	-0.314987883315639	-0.655501867357843
-0.750523194621269	0.523780522661279	0.685012116684361	-0.655501867357843
2.05683172743634	0.938818021940123	-0.314987883315639	0.344498132642157
0.834439306099887	0.523780522661279	2.00694021157172	-0.655501867357843
0.834439306099887	1.26074611682749	2.00694021157172	0.929460633363313
1.57140490026609	0.938818021940123	0.685012116684361	0.344498132642157
2.05683172743634	1.74617294399773	0.685012116684361	2.15185305469976
2.05683172743634	0.523780522661279	1.68501211668436	0.344498132642157
0.249476805378731	2.52378052266128	2.49236703874197	0.929460633363313
1.24947680537873	1.26074611682749	2.26997461740552	1.66642622752952
1.24947680537873	0.938818021940123	0.685012116684361	1.66642622752952
2.41940180682104	1.26074611682749	2.26997461740552	2.34449813264216
1.57140490026609	2.74617294399773	2.00694021157172	0.344498132642157
1.57140490026609	1.74617294399773	-0.314987883315639	0.929460633363313
-0.750523194621269	1.74617294399773	1.26997461740552	0.929460633363313
-0.750523194621269	0.938818021940123	1.26997461740552	1.92946063336331
0.249476805378731	1.93881802194012	2.00694021157172	1.66642622752952
1.24947680537873	2.74617294399773	3.14444373532166	2.66642622752952
1.24947680537873	2.10874302338244	3.85493711812667	3.25138872825068
3.05683172743634	2.26074611682749	3.00694021157172	3.66642622752952
2.05683172743634	2.26074611682749	3.38545183482545	3.92946063336331
1.57140490026609	2.10874302338244	3.49236703874197	3.25138872825068
1.57140490026609	1.74617294399773	3.14444373532166	3.59242564608574
1.83443930609989	1.74617294399773	3.14444373532166	3.73681555542092
1.57140490026609	2.26074611682749	3.7724749579347	3.86806008869917
1.24947680537873	2.10874302338244	3.68501211668436	3.98835432241688
0.834439306099887	2.39824964057742	3.85493711812667	3.34449813264216
1.57140490026609	1.52378052266128	3.68501211668436	3.73681555542092
0.249476805378731	1.74617294399773	3.68501211668436	3.98835432241688
0.249476805378731	2.10874302338244	3.68501211668436	3.51442313408447
0.249476805378731	2.10874302338244	3.38545183482545	2.92946063336331
-0.750523194621269	2.93881802194012	3.38545183482545	4.04493785078325
0.834439306099887	1.74617294399773	2.00694021157172	0.929460633363313
1.57140490026609	2.26074611682749	2.26997461740552	1.34449813264216
0.249476805378731	2.52378052266128	3.26997461740552	1.92946063336331
-0.750523194621269	1.52378052266128	2.68501211668436	2.15185305469976
0.834439306099887	1.26074611682749	2.49236703874197	3.59242564608574
0.834439306099887	2.10874302338244	3.14444373532166	2.80392975127945
0.249476805378731	1.52378052266128	2.49236703874197	1.66642622752952
2.24947680537873	1.52378052266128	2.26997461740552	-0.655501867357843
0.249476805378731	2.10874302338244	2.49236703874197	-0.655501867357843
1.83443930609989	0.523780522661279	2.00694021157172	3.59242564608574
1.83443930609989	0.523780522661279	0.685012116684361	2.80392975127945
2.41940180682104	0.938818021940123	2.68501211668436	2.66642622752952
1.24947680537873	1.26074611682749	2.85493711812667	0.929460633363313
2.41940180682104	-1.06118197805988	1.68501211668436	2.80392975127945
1.57140490026609	-1.06118197805988	-0.314987883315639	2.80392975127945
2.57140490026609	-1.06118197805988	2.85493711812667	0.344498132642157
1.24947680537873	-1.06118197805988	2.85493711812667	2.15185305469976
0.249476805378731	-1.06118197805988	2.68501211668436	1.66642622752952
-0.750523194621269	1.52378052266128	-0.314987883315639	1.34449813264216
-0.750523194621269	1.26074611682749	2.49236703874197	2.15185305469976
-0.750523194621269	1.74617294399773	0.685012116684361	0.929460633363313
-0.750523194621269	1.74617294399773	1.26997461740552	2.51442313408447
-0.750523194621269	0.523780522661279	0.685012116684361	1.92946063336331
0.834439306099887	-0.061181978059877	1.68501211668436	2.66642622752952
0.834439306099887	0.523780522661279	2.26997461740552	0.929460633363313
0.834439306099887	-0.061181978059877	1.68501211668436	0.929460633363313
1.24947680537873	0.523780522661279	2.00694021157172	0.929460633363313

6.70068791721106	6.55352786605533	3.14444373532166	5.34449813264216
7.05037670529904	5.68028500834127	3.00694021157172	5.20247912776973
4.74132990170841	5.41455145290652	3.68501211668436	5.01692347461365
5.24947680537873	6.63230497943945	3.14444373532166	6.88365694375019
6.97739725994193	7.31385745328705	3.7724749579347	6.81210368272515
4.94991652351982	5.39824964057742	4.38545183482545	3.73681555542092
4.64179422815749	5.68028500834127	4.63920842707124	5.01692347461365
6.0043643075422	5.84570861754864	5.68501211668436	5.38889225200061
5.93597733256195	5.97224102347757	2.49236703874197	5.01692347461365
3.70890842401603	5.85768125921472	3.14444373532166	5.1773881468069
3.70890842401603	4.66673847650332	2.85493711812667	4.55395149827111
4.24947680537873	6.09868935871851	2.68501211668436	5.32177805614207
5.0043643075422	5.47797683304815	3.49236703874197	5.12585784616682
6.57140490026609	4.33113544471888	7.79875428273355	6.92192696067791
5.24947680537873	4.93881802194012	6.11127687138646	6.09938563480562
4.86418664949394	5.34820895807782	5.68501211668436	6.04493785078325
5.29387092473718	5.84570861754864	5.81429513362933	5.53432269152217
4.86418664949394	4.98321214129858	5.43989961884783	5.49424525214684
5.24947680537873	5.85768125921472	4.93293963012795	5.1773881468069
5.65886774151643	5.29637002655821	5.38545183482545	5.47378114958712
5.70890842401603	6.04734247871829	6.06005154803129	5.49424525214684
5.67574156008083	6.1772227612652	4.85493711812667	5.86806008869917
5.57140490026609	5.62531854912334	5.54299311181193	5.04493785078325
4.57140490026609	5.36508277664222	6.06005154803129	5.78744162849088
5.55325755355583	5.08856514144481	5.63920842707124	5.80392975127945
5.74132990170841	5.75899698435531	5.66229204018428	5.41058732309993
5.92190214735023	6.06810103888509	5.49236703874196	6.00270961539395
5.77303876143574	6.03685010490065	5.93293963012795	6.12585784616682
6.22675672887865	5.86955535950301	6.16074554765076	6.27523547020504
6.04389267172884	7.06810103888509	5.61574945424725	6.39978056814335
5.74132990170841	5.65306353960625	6.09440305282206	6.53432269152217
5.15636740098725	6.1772227612652	5.68501211668436	6.18998818358653
6.06965576779392	4.86955535950301	5.91383080718024	6.1773881468069
5.08236681954347	5.47797683304815	6.34322359943616	6.0859651190433
5.70890842401603	5.82146107130196	6.04256412130245	5.55395149827111
5.89333299515346	5.26074611682749	5.5676551660462	5.4319609738925
5.27184461840719	5.69370552410359	5.59190271229288	5.09938563480562
5.93597733256195	5.74617294399773	5.91383080718024	4.89908698431979
5.64179422815749	6.03685010490065	5.85493711812667	4.92946063336331
4.94991652351982	5.99410045744131	5.7724749579347	5.49424525214684
4.33693964662907	4.66673847650332	4.89446548231331	4.89908698431979
5.94991652351982	4.4306711182698	6.09440305282206	5.34449813264216
6.0043643075422	4.82146107130196	5.81429513362933	5.34449813264216
6.01766113015566	5.34820895807782	5.29972196079957	5.4319609738925
4.94991652351982	6.11872711195506	5.26997461740552	6.1773881468069
5.03083651890339	4.4306711182698	5.239600968362	5.34449813264216
5.03083651890339	4.72017773546478	5.95179865737926	5.47378114958712
4.53487902424098	5.55352786605533	5.20857407274137	4.83635122897183
5.24947680537873	6.12864258082014	4.93293963012795	4.55395149827111
4.60702880999682	4.96118583496858	4.26997461740552	5.25138872825068
4.53487902424098	4.18674553538371	4.26997461740552	4.83635122897183
3.89333299515346	4.61124336391162	4.72940623604281	4.25138872825068
5.03083651890339	5.27866802482475	5.14444373532166	5.47378114958712
4.57140490026609	5.18674553538371	4.43989961884783	5.04493785078325
4.83443930609989	4.893014332327	4.68501211668436	4.83635122897183
4.41940180682104	4.86955535950301	5.38545183482545	4.98835432241688
4.67574156008083	5.02628086319046	5.239600968362	5.38889225200061
4.20367311576561	4.58267421171485	4.63920842707124	4.20247912776973
4.45893017100768	4.26074611682749	3.85493711812667	3.98835432241688
6.06965576779392	4.18674553538371	3.85493711812667	4.29869444302903

5.86418664949394	3.98321214129858	3.26997461740552	4.66642622752952
5.65886774151643	6.46237997799714	4.85493711812667	5.55395149827111
5.77303876143574	6.61829812144557	4.14444373532166	5.29869444302903
6.06965576779392	5.39824964057742	4.63920842707124	4.80392975127945
6.01766113015566	4.91609794544004	4.7724749579347	5.45302258942033
5.29387092473718	4.96118583496858	3.7724749579347	4.86806008869917
5.3787598223237	4.66673847650332	5.04256412130245	4.73681555542092
5.24947680537873	4.96118583496858	5.11127687138646	5.34449813264216
5.57140490026609	5.96118583496858	4.72940623604281	5.227141182004
5.45893017100768	5.88133252727936	5.11127687138646	4.98835432241688
6.21526109004082	6.21494242721436	5.46637183020902	6.04493785078325
5.96372232304485	5.167636712436	4.93293963012795	5.227141182004
5.87883342545834	5.85768125921472	5.00694021157172	5.15185305469976
5.69242030122746	5.39824964057742	5.04256412130245	5.51442313408447
6.11984152496214	6.40642357202312	5.11127687138646	5.95920797675736
5.51626334607363	6.08856514144481	6.04256412130245	5.59242564608574
5.22675672887865	5.10874302338244	5.20857407274137	4.80392975127945
5.22675672887865	5.47797683304815	5.11127687138646	5.12585784616682
5.60702880999682	5.08856514144481	5.43989961884783	6.05874365030828
5.3787598223237	5.18674553538371	4.14444373532166	6.03099865982538
7.18021414294162	4.33113544471888	5.75110130714213	4.92946063336331
6.55325755355583	6.18674553538371	6.28492495887149	5.45302258942033
5.87883342545834	6.35667053682602	4.7724749579347	6.31028241730424
6.21526109004082	5.12864258082014	5.68501211668436	5.92946063336331
5.0043643075422	5.65306353960625	5.63920842707124	5.227141182004
4.92190214735023	5.33113544471888	5.38545183482545	5.75388906877986
5.80406565705637	7.61124336391162	6.17686521301404	5.68434813552678
4.86418664949394	6.55352786605533	6.28492495887149	5.89908698431979
4.64179422815749	7.25623063570499	4.68501211668436	5.51442313408447
4.83443930609989	6.10874302338244	5.07732953946312	5.12585784616682
5.39922392488341	5.34820895807782	5.63920842707124	4.83635122897183
5.18021414294162	5.47797683304815	5.29972196079957	5.07241858720536
4.89333299515346	4.893014332327	4.68501211668436	5.20247912776973
5.15636740098725	5.0049072123979	4.81429513362933	4.83635122897183
4.86418664949394	4.74617294399773	5.07732953946312	4.25138872825068
4.97739725994193	5.14827138756907	5.239600968362	4.95920797675736
4.77303876143574	5.02628086319046	5.29972196079957	5.25138872825068
4.70890842401603	4.91609794544004	4.97041433554661	4.92946063336331
4.80406565705637	4.893014332327	4.85493711812667	5.07241858720536
4.45893017100768	4.893014332327	5.00694021157172	5.61128467333706
4.77303876143574	5.14827138756907	4.89446548231331	5.27523547020504
3.77303876143574	5.49340687361776	5.14444373532166	5.64827888081926
3.77303876143574	5.20560456263502	4.43989961884783	4.80392975127945
4.97739725994193	5.41455145290652	4.49236703874196	4.59242564608574
5.78863561648676	6.03685010490065	4.14444373532166	6.04493785078325
5.29387092473718	5.14827138756907	5.32886830645909	5.12585784616682
5.3155659958365	4.77170803610486	5.46637183020902	5.25138872825068
3.24947680537873	3.63925774008121	4.14444373532166	4.04493785078325
3.49740431882232	3.39824964057742	4.32886830645909	4.55395149827111
2.41940180682104	1.93881802194012	3.26997461740552	3.51442313408447
2.70890842401603	2.52378052266128	3.14444373532166	3.25138872825068
2.94991652351982	2.63925774008122	4.20857407274137	3.15185305469976
4.05683172743634	2.74617294399773	3.68501211668436	4.38889225200061
3.15636740098725	3.69370552410359	3.38545183482545	3.86806008869917
3.77303876143574	1.26074611682749	3.26997461740552	3.04493785078325
3.64179422815749	2.10874302338244	3.26997461740552	2.80392975127945
2.94991652351982	3.33113544471888	4.32886830645909	3.73681555542092
2.24947680537873	4.58267421171485	3.93293963012795	4.04493785078325
4.1074578005063	3.18674553538371	4.32886830645909	3.73681555542092
2.83443930609989	2.84570861754864	3.38545183482545	4.04493785078325

4.20367311576561	4.39824964057742	1.68501211668436	2.80392975127945
5.29387092473718	3.39824964057742	2.00694021157172	2.92946063336331
4.41940180682104	2.63925774008122	2.26997461740552	2.92946063336331
2.70890842401603	3.98321214129858	2.49236703874197	3.80392975127945
3.41940180682104	3.79679901706769	2.26997461740552	2.51442313408447
5.58932680826336	4.06810103888509	2.85493711812667	3.92946063336331
6.27184461840719	4.18674553538371	2.85493711812667	2.80392975127945
4.05683172743634	5.38176151778885	4.20857407274137	4.04493785078325
3.41940180682104	7.62181260562181	5.75110130714213	4.34449813264216
4.0043643075422	6.81533496850512	5.85493711812667	5.20247912776973
5.29387092473718	5.12864258082014	4.32886830645909	4.51442313408447
3.57140490026609	5.31385745328705	4.00694021157172	2.92946063336331
4.80406565705637	5.92750270871229	3.00694021157172	3.59242564608574
4.24947680537873	5.893014332327	3.85493711812667	3.80392975127945
5.03083651890339	3.18674553538371	4.54299311181193	5.53432269152217
4.97739725994193	3.02628086319046	4.07732953946312	5.68434813552678
5.3787598223237	4.63925774008121	3.49236703874197	5.47378114958712
4.45893017100768	2.52378052266128	3.85493711812667	5.53432269152217
4.33693964662907	2.63925774008122	3.14444373532166	5.80392975127945
4.05683172743634	3.69370552410359	3.49236703874197	2.66642622752952
4.24947680537873	3.18674553538371	3.14444373532166	3.66642622752952
2.83443930609989	3.69370552410359	5.38545183482545	4.4319609738925
2.70890842401603	2.93881802194012	5.54299311181193	4.15185305469976
2.41940180682104	3.74617294399773	4.20857407274137	3.86806008869917
1.83443930609989	3.33113544471888	4.49236703874196	4.20247912776973
2.70890842401603	3.46237997799714	4.20857407274137	3.86806008869917
3.41940180682104	4.02628086319046	3.49236703874197	5.20247912776973
3.05683172743634	3.79679901706769	4.32886830645909	4.73681555542092
2.94991652351982	4.10874302338244	4.00694021157172	4.25138872825068
3.24947680537873	3.93881802194012	4.00694021157172	3.98835432241688
3.05683172743634	4.63925774008121	4.20857407274137	3.34449813264216
2.94991652351982	4.22422024080237	4.14444373532166	3.59242564608574
3.15636740098725	4.49340687361776	3.7724749579347	4.04493785078325
2.57140490026609	4.06810103888509	2.85493711812667	4.34449813264216
4.74132990170841	3.39824964057742	4.07732953946312	4.29869444302903
5.55325755355583	2.74617294399773	3.49236703874197	4.47378114958712
3.49740431882232	3.46237997799714	3.59190271229288	4.25138872825068
3.77303876143574	4.10874302338244	2.49236703874197	4.95920797675736
3.64179422815749	3.79679901706769	3.14444373532166	4.80392975127945
2.57140490026609	3.18674553538371	3.38545183482545	2.80392975127945
3.41940180682104	3.74617294399773	3.59190271229288	3.04493785078325
3.24947680537873	3.69370552410359	4.14444373532166	3.92946063336331
2.83443930609989	3.93881802194012	4.07732953946312	4.25138872825068
3.70890842401603	2.93881802194012	5.46637183020902	3.34449813264216
4.05683172743634	4.39824964057742	4.26997461740552	4.77076288734425
3.57140490026609	4.06810103888509	4.07732953946312	4.34449813264216
3.15636740098725	3.58267421171485	3.85493711812667	3.73681555542092
3.41940180682104	3.10874302338244	4.00694021157172	4.29869444302903
1.83443930609989	1.93881802194012	4.07732953946312	3.98835432241688
3.33693964662907	2.26074611682749	4.54299311181193	4.04493785078325
2.41940180682104	4.52378052266128	5.14444373532166	4.09938563480562
3.33693964662907	5.80918274152353	4.38545183482545	4.15185305469976
3.70890842401603	4.66673847650332	5.79353657346253	5.45302258942033
3.41940180682104	4.86955535950301	5.14444373532166	5.04493785078325
4.29387092473718	4.79679901706769	5.14444373532166	5.04493785078325
4.49740431882232	4.29637002655821	4.72940623604281	4.77076288734425
4.80406565705637	4.91609794544004	5.54299311181193	4.83635122897183
6.3787598223237	5.08856514144481	5.97041433554661	5.1773881468069
6.01766113015566	3.39824964057742	4.43989961884783	2.80392975127945
6.63318109785278	3.26074611682749	4.54299311181193	3.59242564608574

7.22675672887865	3.63925774008121	3.59190271229288	4.34449813264216
4.33693964662907	4.49340687361776	4.85493711812667	5.227141182004
3.70890842401603	4.06810103888509	4.43989961884783	4.86806008869917
3.89333299515346	5.12864258082014	4.54299311181193	3.66642622752952
3.83443930609989	3.84570861754864	4.63920842707124	4.09938563480562
4.15636740098725	3.58267421171485	4.68501211668436	4.73681555542092
5.05683172743634	4.22422024080237	4.72940623604281	4.66642622752952
5.13211985474057	4.14827138756907	5.00694021157172	5.27523547020504
4.29387092473718	4.55352786605533	4.81429513362933	4.62990035150441
4.1074578005063	4.46237997799714	4.38545183482545	3.4319609738925
4.57140490026609	3.79679901706769	4.59190271229288	3.66642622752952
4.24947680537873	4.02628086319046	3.7724749579347	3.73681555542092
4.24947680537873	5.12864258082014	5.43989961884783	3.59242564608574
4.05683172743634	4.58267421171485	4.20857407274137	5.07241858720536
4.29387092473718	4.46237997799714	5.04256412130245	4.80392975127945
4.41940180682104	5.80918274152353	4.38545183482545	4.80392975127945
4.80406565705637	5.27866802482475	3.49236703874197	3.73681555542092
4.74132990170841	3.98321214129858	4.38545183482545	3.92946063336331
4.92190214735023	4.77170803610486	4.20857407274137	3.80392975127945
5.51626334607363	3.98321214129858	3.49236703874197	3.73681555542092
4.94991652351982	4.22422024080237	4.26997461740552	4.70205013726024
4.3787598223237	4.10874302338244	4.26997461740552	4.98835432241688
4.57140490026609	5.08856514144481	3.93293963012795	4.47378114958712
4.70890842401603	5.06810103888509	3.93293963012795	4.86806008869917
5.22675672887865	4.72017773546478	3.49236703874197	4.62990035150441
5.51626334607363	5.29637002655821	3.38545183482545	4.92946063336331
5.1074578005063	4.39824964057742	6.25486772501531	5.57331682313804
4.24947680537873	4.72017773546478	4.38545183482545	4.38889225200061
4.05683172743634	3.893014332327	4.63920842707124	4.29869444302903
3.57140490026609	3.46237997799714	4.68501211668436	4.86806008869917
3.24947680537873	3.84570861754864	5.00694021157172	5.20247912776973
4.49740431882232	4.86955535950301	4.14444373532166	4.20247912776973
2.70890842401603	3.33113544471888	3.68501211668436	4.34449813264216
4.15636740098725	4.26074611682749	3.59190271229288	3.92946063336331
3.64179422815749	5.26074611682749	3.38545183482545	4.66642622752952
1.57140490026609	2.39824964057742	3.00694021157172	0.929460633363313
4.45893017100768	2.39824964057742	3.26997461740552	2.80392975127945
1.83443930609989	2.74617294399773	3.14444373532166	4.20247912776973
2.70890842401603	1.26074611682749	3.85493711812667	2.66642622752952
-0.750523194621269	1.93881802194012	4.00694021157172	3.59242564608574
1.83443930609989	2.52378052266128	2.00694021157172	3.25138872825068
0.249476805378731	2.26074611682749	2.68501211668436	2.51442313408447
0.249476805378731	3.18674553538371	1.26997461740552	3.04493785078325
2.41940180682104	3.69370552410359	1.68501211668436	3.51442313408447
0.834439306099887	3.39824964057742	2.00694021157172	2.92946063336331
2.24947680537873	2.52378052266128	2.26997461740552	2.80392975127945
1.57140490026609	2.26074611682749	3.14444373532166	3.04493785078325
1.57140490026609	2.74617294399773	2.68501211668436	2.92946063336331
2.57140490026609	1.93881802194012	2.68501211668436	2.51442313408447
2.57140490026609	2.39824964057742	3.49236703874197	2.80392975127945
3.70890842401603	3.10874302338244	2.68501211668436	2.80392975127945
2.83443930609989	2.93881802194012	2.68501211668436	2.92946063336331
3.77303876143574	3.58267421171485	2.68501211668436	3.15185305469976
3.49740431882232	2.26074611682749	2.49236703874197	2.34449813264216
2.94991652351982	2.74617294399773	2.49236703874197	2.51442313408447
3.57140490026609	1.26074611682749	1.26997461740552	3.51442313408447
2.24947680537873	1.26074611682749	0.685012116684361	2.15185305469976
1.57140490026609	2.10874302338244	1.68501211668436	2.34449813264216
0.834439306099887	1.93881802194012	2.26997461740552	2.15185305469976
1.83443930609989	3.10874302338244	3.49236703874197	3.51442313408447

1.57140490026609	1.93881802194012	2.26997461740552	3.51442313408447
1.57140490026609	1.93881802194012	3.00694021157172	2.34449813264216
1.57140490026609	2.10874302338244	2.68501211668436	1.92946063336331
1.83443930609989	1.93881802194012	3.14444373532166	2.51442313408447
1.57140490026609	2.39824964057742	2.85493711812667	2.51442313408447
1.83443930609989	2.52378052266128	3.26997461740552	2.80392975127945
1.24947680537873	2.63925774008122	2.49236703874197	1.66642622752952
2.24947680537873	2.63925774008122	2.26997461740552	2.15185305469976
1.57140490026609	4.26074611682749	3.85493711812667	3.98835432241688
2.05683172743634	4.29637002655821	4.00694021157172	2.80392975127945
2.57140490026609	4.82146107130196	3.7724749579347	2.92946063336331
2.70890842401603	3.26074611682749	3.49236703874197	2.51442313408447
2.24947680537873	3.02628086319046	2.26997461740552	3.86806008869917
1.83443930609989	3.18674553538371	3.00694021157172	3.04493785078325
1.57140490026609	2.74617294399773	2.49236703874197	3.15185305469976
3.89333299515346	3.26074611682749	2.85493711812667	3.51442313408447
4.29387092473718	2.52378052266128	3.68501211668436	3.34449813264216
2.83443930609989	3.39824964057742	3.38545183482545	3.51442313408447
2.83443930609989	3.33113544471888	3.14444373532166	2.80392975127945
2.57140490026609	3.46237997799714	4.26997461740552	2.80392975127945
2.57140490026609	3.58267421171485	3.49236703874197	3.66642622752952
2.83443930609989	3.74617294399773	3.14444373532166	2.51442313408447
2.24947680537873	3.26074611682749	2.68501211668436	2.92946063336331
3.24947680537873	2.52378052266128	3.14444373532166	2.80392975127945
3.05683172743634	3.63925774008121	3.38545183482545	3.25138872825068
2.41940180682104	2.84570861754864	3.85493711812667	2.92946063336331
3.70890842401603	2.26074611682749	1.68501211668436	2.51442313408447
3.05683172743634	3.10874302338244	2.00694021157172	3.25138872825068
3.94991652351982	1.74617294399773	2.49236703874197	3.80392975127945
3.77303876143574	2.52378052266128	2.68501211668436	3.4319609738925
3.24947680537873	3.33113544471888	2.26997461740552	4.04493785078325
2.83443930609989	3.33113544471888	2.49236703874197	3.51442313408447
3.49740431882232	5.52378052266128	4.97041433554661	2.34449813264216
2.05683172743634	4.96118583496858	5.29972196079957	4.20247912776973
3.64179422815749	4.06810103888509	3.59190271229288	3.51442313408447
2.24947680537873	3.39824964057742	3.26997461740552	3.15185305469976
1.83443930609989	4.39824964057742	4.00694021157172	3.15185305469976
2.05683172743634	3.52378052266128	4.32886830645909	4.51442313408447
3.15636740098725	3.39824964057742	3.85493711812667	4.25138872825068
2.41940180682104	2.84570861754864	4.07732953946312	3.51442313408447
3.05683172743634	3.18674553538371	4.07732953946312	4.34449813264216
3.70890842401603	2.63925774008122	2.68501211668436	2.66642622752952
3.70890842401603	1.74617294399773	3.14444373532166	1.34449813264216
2.57140490026609	3.02628086319046	3.59190271229288	1.34449813264216
3.05683172743634	3.84570861754864	3.00694021157172	1.92946063336331
2.83443930609989	3.52378052266128	4.43989961884783	4.15185305469976
3.70890842401603	3.18674553538371	4.07732953946312	3.51442313408447
1.83443930609989	2.74617294399773	3.93293963012795	3.66642622752952
2.41940180682104	2.52378052266128	4.43989961884783	3.73681555542092
2.83443930609989	2.74617294399773	4.32886830645909	4.29869444302903
2.05683172743634	1.74617294399773	4.97041433554661	2.80392975127945
1.57140490026609	1.93881802194012	3.00694021157172	2.80392975127945
2.24947680537873	2.10874302338244	3.59190271229288	3.34449813264216
2.41940180682104	1.93881802194012	2.85493711812667	3.34449813264216
2.05683172743634	3.10874302338244	2.49236703874197	3.98835432241688
2.05683172743634	2.26074611682749	2.85493711812667	3.73681555542092
2.24947680537873	1.74617294399773	2.26997461740552	2.34449813264216
2.24947680537873	3.18674553538371	2.85493711812667	3.15185305469976
2.05683172743634	3.26074611682749	3.49236703874197	2.66642622752952
13.3910251889661	16.9888313745742	15.9337921225016	15.205391114981

13.1989387847389	14.4028212557451	14.4151637898577	13.7798428390155
11.7666540580683	14.9391481908464	14.082085055638	13.8845656135799
13.5918293154665	13.8432174433207	12.7723091976035	13.0034905662143
12.7006879172111	12.2927918437583	10.8271191739869	11.1506449359627
10.8456665615231	13.7616377779631	11.813650929537	12.6162529382886
10.8045451967121	8.36298431075822	9.31982316685608	8.81006453745156
8.30204485618289	10.8264194464488	10.0304173634022	8.32177805614207
8.07919954046479	7.90748481513533	8.2280439369396	9.87197513870255
8.60262363087681	6.86955535950301	9.46637183020902	7.67988848733608
9.54639301225802	7.01563361899095	11.5131486008785	8.97203201711495
8.66521857366882	8.59345405046809	9.27372675226663	9.64141433952144
7.69656003158838	8.24031421692267	10.0469558904196	9.46214123403125
8.65035624166092	6.8988199540082	8.83221704162659	8.36130642032871
8.86602564915772	7.48571248182776	8.10286463157026	8.87001894173723
9.58932680826335	7.68365185943967	9.21053292577943	6.96654995209853
8.38133376598752	7.84570861754864	8.28492495887149	7.35012268183604
10.1121141629375	8.5608698413965	9.5003954124979	8.23324138154041
10.3903065761517	12.0137956842413	10.6231214429036	7.09938563480562
9.79637126526637	8.07580913402035	10.1718471382474	8.62990035150441
9.17725476746107	9.02229734927197	10.0046842376314	9.33318281941432
9.44053601991039	9.41455145290652	9.90175797487967	8.88365694375019
8.96200380581855	8.87545596094269	9.40952596980431	9.24988513766029
9.17428930898451	9.04472653051128	9.32706380961234	8.99554982382108
9.09809974580807	8.93599750287774	9.36976073710599	9.27966318224585
9.44423365980098	9.44562145973577	9.95764190166073	9.40789321393067
9.37489027586205	9.54151688695809	9.77512453634865	9.86511681319844
8.75131399028103	9.17243769869982	8.20464836952758	8.68879404055797
8.72115201977078	8.46237997799713	8.40952596980431	8.60424139633294
8.68201870576699	8.35667053682602	8.63920842707124	8.90864762262789
8.19492064175664	8.93033986801582	8.84488345346275	8.74537756892434
8.54179843818077	9.37240346309062	9.25865930417768	9.0604601228973
8.40429491443084	8.88864473269923	9.32163673722801	8.87977350926296
9.30204485618289	9.15677571980424	9.20464836952758	9.23628183586047
9.68410503301546	9.36508277664222	9.18086714357153	9.0239782321476
9.91658834745376	9.95004527736338	8.27746915395244	8.36963769492066
9.8521756703967	9.75740019942098	8.48591201660467	8.89908698431979
9.84193384264681	10.4977170030868	8.06005154803129	9.6675528929838
9.03247501429914	9.0460350975315	9.57527639370547	8.73896082725247
9.32762761311338	9.66758887148278	9.36800670036605	9.1143359762716
9.42689634336797	10.0368501049007	9.56152906324936	8.73466708884234
8.8117192295998	8.66333187506007	8.81943843690529	8.21486285222556
9.23247038007304	9.76298123168428	9.16882789394862	8.90674055686323
9.27876403234698	9.83060172515843	9.51314860087847	9.37102157516192
9.46864532584089	9.04079369288936	8.49236703874196	9.45954178280387
9.34355449105064	9.23343877083175	9.16074554765076	9.50563001562646
10.1623661416087	9.95701820075335	8.77512453634865	9.10272134736888
9.89333299515346	10.2114478069165	8.64501404875244	8.81413994988167
9.56914892632573	9.12987723647178	9.26244094472011	8.59005083889784
8.76320440133117	9.56269951195358	8.87976897110661	9.42664717399603
8.21814359857394	10.7550017033725	9.61130811146547	9.31028241730424
9.95510919274015	11.8641870135146	10.4507126043355	9.89332004110091
9.83443930609989	10.5658958624575	10.0965231046964	9.42398291646897
9.67048966115796	10.2618727822818	8.76716115803823	8.95182844639177
11.2648918577654	10.1628196961382	9.89690041123037	9.29286536422683
10.1159830176049	11.4351725105047	9.47454576165472	9.52937347555044
8.27461636765724	8.66673847650332	9.69484073405247	8.63221051219161
9.23389526417987	8.99002696285489	10.4390644842133	8.72604108382674
12.0453135463124	11.2786680248247	11.2347967846322	11.1344392610331
11.2942124314356	12.3589098265424	11.7866595667151	11.5736173533008
10.7053177145051	10.8438288843304	10.3015609604634	10.5037387830538

11.0176611301557	11.2579255977651	10.41037837458	10.6970933637765
9.49621740387187	11.4319229144793	10.792883030964	9.76445831049005
9.57027735426037	10.690780435401	10.6622920401843	9.7064419063774
7.78085826589504	13.2948572629241	10.4753606134363	10.5156749302939
10.6185288123801	8.65134502237995	11.0965231046964	9.16627211461272
9.15185191986476	8.61829812144557	11.1346766551606	10.6647345778838
9.66732932026463	11.6620523228335	9.86616437354993	10.5386393715053
8.6609877933908	8.94022621633293	8.77776925760421	10.3081167500861
5.92190214735023	7.50106044616119	6.09440305282206	6.01692347461365
8.15335865111491	7.59345405046809	9.40440093762644	8.41596049519878
9.91481272256391	4.82146107130196	9.45319644146129	6.55395149827111
8.78863561648676	7.04734247871829	8.67369680345653	7.03099865982538
7.99094379177988	6.51624684997587	7.53050216762874	7.83233816646521
8.25790542744931	6.99410045744131	7.63337934826904	7.47378114958712
8.59377271329455	6.86955535950301	8.37849907418369	7.17104661993307
8.16834004265333	7.82756127083838	6.47942798303447	7.62062253791639
8.86786230763734	7.53873086412725	7.53676115810042	7.61596116054653
8.88247200252169	7.91895759957928	7.25486772501531	8.02749271632384
8.8174328807942	4.4306711182698	8.79092062525552	7.65283716278156
8.14126050859704	5.80918274152353	8.38197964291865	8.04840170608682
7.57140490026609	6.52378052266128	7.44656334912884	7.29869444302903
6.84938964756586	6.36508277664222	7.67369680345653	7.92569871456711
4.83443930609989	7.49340687361776	9.73486066613492	3.86806008869917
6.3787598223237	4.74617294399773	8.01592899479898	6.57331682313804
6.57140490026609	5.70700234671705	5.70737992971282	6.35572538806541
6.82690563341448	4.893014332327	5.63920842707124	5.29869444302903
6.15636740098725	4.96118583496858	6.25486772501531	4.62990035150441
6.65035624166092	4.79679901706769	5.32886830645909	5.64827888081926
6.41940180682104	4.63925774008121	5.63920842707124	5.80392975127945
6.8117192295998	4.26074611682749	5.63920842707124	6.34449813264216
7.84193384264681	2.63925774008122	6.16074554765076	4.77076288734425
8.75727144557743	5.08856514144481	7.47942798303447	6.26336136991675
8.54868482376601	6.07836937433892	6.00694021157172	5.20247912776973
7.38902815777752	7.67352764216596	6.81429513362933	7.78744162849089
7.2381614921509	7.20560456263502	7.81429513362933	7.70205013726024
7.64179422815749	6.25170097722448	5.54299311181193	6.37792113417961
7.44423365980098	6.13849036677649	6.7724749579347	7.09269098223162
8.26628509306529	6.52378052266128	6.06005154803129	6.2393158959501
6.87152862483511	6.64617715402101	6.50519107909955	6.39978056814335
6.49740431882232	7.4023423952113	6.25486772501531	6.35572538806541
7.11366295003301	7.24715705207953	6.00694021157172	7.23324138154042
7.0043643075422	6.8871852535248	6.00694021157172	7.00270961539395
6.78085826589504	7.11872711195506	5.98879286486146	7.0859651190433
7.2381614921509	7.14827138756907	6.5179021308491	7.11928519224333
6.95683593745961	7.04734247871829	6.74029455218555	6.82023156360855
7.52560121065297	6.47019948245643	6.72940623604281	7.227141182004
7.94991652351982	6.96672401851001	6.32886830645909	6.72820242511621
7.23247038007304	6.83363578524807	6.07732953946312	6.89139259252979
6.32629240242956	7.19148345439037	6.66229204018428	6.71082034688797
7.06325799659577	7.8636305255459	7.04256412130245	7.04493785078325
6.99094379177988	7.32252231441417	6.45319644146129	7.03798509014148
6.96372232304485	6.97224102347757	6.78304419964489	6.85229277284085
7.78475218199953	7.4023423952113	7.16882789394862	7.65738108792651
7.32629240242956	6.79679901706769	6.71843511822181	6.88365694375019
7.04389267172884	7.01028038449675	7.05133433093018	7.19624717405821
6.84938964756586	6.65306353960625	7.04256412130245	7.10604936508664
7.0242638649799	6.68028500834127	6.19280675688306	7.26931063624794
6.74932269246194	7.02096706329399	6.54299311181193	7.14539803256246
6.97739725994193	7.04734247871829	6.79353657346253	6.89908698431979
7.1074578005063	7.15313714274089	6.98879286486146	6.38889225200061

6.57140490026609	6.36508277664222	6.29972196079957	6.75388906877986
6.77303876143574	6.61829812144557	6.89446548231331	6.74537756892434
6.90052849655766	7.18674553538371	7.38545183482545	7.37240412921204
6.49740431882232	6.96118583496858	7.35743745865586	7.4319609738925
6.95683593745961	5.98321214129858	6.46637183020902	7.32177805614207
6.61579901962455	6.65991721064731	6.97041433554661	7.26336136991675
7.09496685632311	6.83363578524807	7.26997461740552	6.87587959315847
6.41940180682104	7.38590124814978	7.46637183020902	6.98835432241688
6.49740431882232	7.12369336484841	6.8649212066993	6.89908698431979
6.42938589539367	6.26973490005474	6.71843511822181	6.86806008869917
6.33693964662907	6.97224102347757	6.5676551660462	6.72820242511621
6.65035624166092	6.83363578524807	6.91383080718024	6.84434401972536
5.57140490026609	6.68701087152958	7.02486211956899	6.90674055686323
6.32629240242956	6.4306711182698	7.79875428273355	6.9143537409731
6.48788154470381	6.56817464201973	7.29972196079957	7.27523547020504
6.59820495960981	6.5312750592082	7.05133433093018	7.45824029869135
5.97739725994193	6.26074611682749	6.47942798303447	7.1837019207391
6.26070406080199	5.80918274152353	7.18485800376757	6.98835432241688
5.69242030122746	6.2875461761712	7.07732953946312	6.42131372969299
6.01766113015566	6.50867363027107	6.81429513362933	6.73681555542092
6.82690563341448	7.43467304882729	7.34322359943616	7.29286536422683
6.11984152496214	6.63230497943945	7.64501404875244	6.61128467333706
6.70068791721106	6.40642357202312	7.2007119549684	6.82831390990641
5.62451623672566	5.86955535950301	6.98879286486146	6.15185305469976
6.21526109004082	6.14827138756907	6.6275266220236	6.21486285222556
5.99094379177988	6.26973490005474	7.73486066613492	6.77912636027888
6.55325755355583	6.57544264248377	7.37849907418369	6.62062253791639
6.46864532584089	7.62531854912334	8.06871640915841	6.98112275318581
5.69242030122746	7.8636305255459	6.61574945424725	6.49424525214684
6.72521023634513	6.54614833568973	6.47942798303447	6.00270961539395
7.19199131071797	6.81533496850512	5.93293963012795	6.78744162849088
6.43930136425875	7.53500777808453	6.00694021157172	5.68434813552678
6.05683172743634	7.12369336484841	6.16074554765076	6.227141182004
6.14429456868668	6.76536650923104	5.85493711812667	6.26336136991675
6.33693964662907	6.91609794544004	5.66229204018428	6.54417047747852
5.93597733256195	6.86955535950301	6.14444373532166	6.34449813264216
6.64179422815749	6.20560456263502	5.20857407274137	5.70205013726024
6.61579901962455	6.57544264248377	5.38545183482545	5.88365694375019
6.36841787810224	6.68028500834127	5.41293257124756	5.77076288734425
6.45893017100768	7.16281969613823	6.67369680345653	5.64827888081926
7.11984152496214	6.79056706335618	6.91383080718024	6.77076288734425
6.58932680826336	6.42263379920438	6.53050216762874	6.50436946942055
5.75727144557743	7.82146107130196	6.00694021157172	5.73681555542092
6.41940180682104	6.50867363027107	6.00694021157172	6.95920797675736
6.15636740098725	5.83363578524807	5.20857407274137	6.01692347461365
6.2381614921509	6.01563361899095	5.04256412130245	6.227141182004
6.96372232304485	6.7003692543846	6.50519107909955	5.86806008869917
6.73329258264299	6.26973490005474	5.83475923618904	6.11268245741908
6.65035624166092	6.54614833568973	5.85493711812667	6.44253021560268
6.11984152496214	5.83363578524807	6.07732953946312	4.77076288734425
6.28289980691618	6.03685010490065	6.04256412130245	5.61128467333706
6.52560121065297	7.03685010490065	6.66229204018428	5.12585784616682
6.60702880999682	6.47797683304815	6.37151264386758	6.0859651190433
6.68410503301546	6.46237997799714	6.46637183020902	5.92946063336331
5.70890842401603	6.23343877083175	6.14444373532166	6.227141182004
5.96372232304485	5.91609794544004	5.87483667556438	6.47378114958712
6.38902815777752	6.25170097722448	5.95179865737926	6.00270961539395
5.65886774151643	6.87545596094269	6.06005154803129	6.00270961539395
5.29387092473718	6.50867363027107	5.79353657346253	5.25138872825068
5.72521023634513	6.87545596094269	6.49236703874196	6.56366665310432

5.87883342545834	6.70700234671705	6.60387535395896	6.58290287196724
6.71708235546173	6.82756127083838	7.38545183482545	6.90674055686323
7.05683172743634	6.07836937433892	6.34322359943616	6.62990035150441
7.32093916793535	6.98321214129858	6.41293257124756	6.36686594567061
7.28839579467103	7.03157516285998	5.43989961884783	6.55395149827111
5.80406565705637	7.31819638901138	5.91383080718024	6.0859651190433
6.57140490026609	6.38176151778885	5.72940623604281	6.90674055686323
6.3580012621569	6.26074611682749	5.5179021308491	6.21486285222556
6.54409755427036	6.20560456263502	6.38545183482545	5.62990035150441
6.08236681954347	6.30514023618594	6.16074554765076	6.39978056814335
6.36841787810224	6.39824964057742	6.11127687138646	6.66642622752952
5.65886774151643	5.26074611682749	5.75110130714213	6.03099865982538
6.1074578005063	5.4306711182698	5.63920842707124	6.25138872825068
5.57140490026609	6.27866802482475	6.34322359943616	5.88365694375019
5.22675672887865	6.50867363027107	6.25486772501531	5.94441097482928
4.94991652351982	6.91036157589089	5.95179865737926	6.72820242511621
4.86418664949394	5.79679901706769	5.83475923618904	6.99554982382109
6.16834004265333	6.35667053682602	4.97041433554661	5.77076288734425
6.18021414294162	5.92750270871229	5.11127687138646	5.55395149827111
5.75727144557743	5.88133252727936	6.00694021157172	5.51442313408447
6.41940180682104	5.63925774008121	5.54299311181193	6.20247912776973
5.99094379177988	6.48571248182776	5.66229204018428	6.13891399899226
6.08236681954347	5.63925774008121	7.06005154803129	5.75388906877986
6.13211985474057	5.84570861754864	5.14444373532166	5.78744162849088
5.99094379177988	5.77170803610486	5.14444373532166	5.12585784616682
6.2381614921509	6.167636712436	5.5179021308491	5.88365694375019
5.75727144557743	6.26973490005474	5.75110130714213	5.83635122897183
6.13211985474057	6.39824964057742	5.5676551660462	5.80392975127945
7.33693964662907	6.44661266213882	4.97041433554661	5.09938563480562
6.42938589539367	5.63925774008121	5.81429513362933	5.32177805614207
5.67574156008083	7.26074611682748	8.06871640915841	5.04493785078325
6.24947680537873	5.56817464201973	7.74570804837192	6.95182844639177
6.19199131071797	6.09868935871851	7.22417092779239	6.15185305469976
7.40429491443084	6.5312750592082	7.76182771373519	7.47892445286308
6.65886774151643	6.55352786605533	6.76182771373519	6.83635122897183
5.43930136425875	6.64617715402101	5.00694021157172	5.227141182004
6.03083651890339	7.93881802194012	5.68501211668436	5.07241858720536
5.72521023634513	6.25170097722448	5.83475923618904	4.62990035150441
5.72521023634513	7.04734247871829	5.54299311181193	5.86806008869917
6.20367311576561	6.7843080728845	7.06871640915841	5.70205013726024
6.89333299515346	6.44661266213882	6.94239995937701	6.69322628687323
5.03083651890339	5.18674553538371	5.72940623604281	6.55395149827111
7.05037670529904	6.50867363027107	6.82456346908315	6.00270961539395
7.17428930898451	7.06293933376931	5.54299311181193	6.41058732309993
7.19784403696341	6.53873086412725	4.26997461740552	5.98835432241688
6.85680711912834	6.10874302338244	5.239600968362	5.73681555542092
7.05037670529904	7.02628086319046	5.49236703874196	6.69322628687323
6.53487902424098	6.04734247871829	6.11127687138646	6.20247912776973
7.13822005427699	6.43866390902333	4.97041433554661	6.20247912776973
7.0242638649799	5.50867363027107	5.72940623604281	5.82023156360855
6.64179422815749	5.50867363027107	6.83475923618904	6.07241858720536
7.24947680537873	5.61124336391162	6.84488345346275	6.15185305469976
7.81553084354982	5.20560456263502	6.45319644146129	6.70205013726024
6.89333299515346	5.72017773546478	7.26997461740552	6.227141182004
7.32629240242956	5.77170803610486	7.32163673722801	6.1773881468069
5.80406565705637	6.56817464201973	6.88468446152073	6.41058732309993
5.78863561648676	6.47019948245643	6.25486772501531	6.2393158959501
6.38902815777752	6.25170097722448	6.50519107909955	6.54417047747852
6.32629240242956	6.04734247871829	6.32886830645909	6.86806008869917
6.43930136425875	6.13849036677649	6.19280675688306	6.58290287196724

6.06965576779392	5.62531854912334	5.41293257124756	7.37240412921204
6.20367311576561	6.26074611682749	6.16074554765076	6.72820242511621
6.78863561648676	9.65048499550447	7.70182040437092	6.52440722265709
6.27184461840719	7.64617715402101	6.60387535395896	6.42131372969299
5.74132990170841	8.21958879207073	6.72940623604281	6.51442313408447
7.67574156008083	7.85768125921472	8.93293963012795	7.5392549870644
7.03737936477016	8.50487206011121	8.18485800376757	7.20247912776973
5.89333299515346	8.52940507185516	9.01592899479898	7.05874365030828
6.27184461840719	8.02893044160441	7.06871640915841	6.36686594567061
4.92190214735023	10.0381658323433	6.95179865737926	8.13240069203359
4.60702880999682	7.68028500834127	6.239600968362	6.88365694375019
8.13211985474057	8.92323648074126	8.75915357943687	8.80802250591334
8.11366295003301	8.53314262586497	8.90418063714652	8.96837962265561
7.75330254337448	8.35667053682602	8.55537683626777	8.17422086772821
7.70480402568329	8.79368640520036	8.40611130539155	8.1773881468069
7.88610142592238	8.03157516285998	8.06871640915841	7.92946063336331
7.90052849655766	8.41859828596922	8.32163673722801	7.94441097482928
8.24383024223759	8.34608578618485	8.62165005568693	8.31028241730424
8.0855271604368	8.8591708773552	8.47617500523938	8.51442313408447
7.87883342545834	8.43267347118094	8.2280439369396	7.73681555542092
7.9807958364038	8.04734247871829	7.94710696205454	8.48660518994471
8.16535618421451	8.07324434216105	7.5179021308491	8.65511091430169
8.18021414294162	7.75259921315716	7.89933123748513	8.18684847605596
8.07919954046479	7.8426998676763	8.02040247137829	8.07241858720536
8.26070406080198	8.53873086412725	8.78829992509638	8.26931063624794
7.99766965496819	8.41455145290652	8.66229204018428	8.20247912776973
9.15636740098725	9.3279115438446	9.17285215050741	8.00270961539395
8.98587873669702	9.09363613099223	8.71291811325425	8.05185726472304
8.802145902893	8.27420837663405	8.17285215050741	8.68434813552678
9.26628509306529	8.45451786022416	8.63920842707124	8.56849980684026
8.71708235546173	8.62181260562181	9.05133433093018	8.7064419063774
8.39668173032096	9.11499117104761	9.08589155296654	8.55638642718816
8.7413299017084	8.93033986801582	8.37501008810381	8.65511091430169
7.49740431882232	8.34183004551512	7.99789507196872	7.82831390990641
8.21814359857394	8.1137437044408	8.18883785468011	7.90674055686323
8.2007615203457	8.61829812144557	8.4197217369102	8.39162204475618
8.49265078885168	8.69870620516196	8.11546466834989	7.83233816646521
8.29387092473718	8.71853737708353	8.16882789394862	7.97749332978511
8.81933241370968	7.90460230660221	7.99335114682377	8.0239782321476
8.78280653768456	8.21261362115439	8.8423590520472	8.13891399899226
8.0043643075422	8.47603642247872	8.25486772501531	8.33601997871785
8.58039368349335	8.26749294926807	8.19280675688306	7.75812606166633
8.7453318322659	8.28089268993926	8.28492495887149	8.15827932385919
8.88247200252169	8.37135992232838	8.31800731382732	7.92192696067791
7.13211985474057	9.44363700957296	7.29972196079957	9.12749634156257
8.03083651890339	10.769333228917	8.51156060397528	8.95920797675736
5.62451623672566	10.5415168869581	9.73213602879839	8.6505598220705
9.38518609148313	9.41859828596922	7.97963286557599	8.31028241730424
7.70068791721106	7.34820895807783	7.69623937210762	6.77076288734425
7.74132990170841	7.96672401851001	8.18883785468011	7.57811780940186
8.18316746033096	7.2875461761712	9.40781964785391	8.40519406432971
8.28839579467103	7.32252231441417	8.65079640134645	8.12257526217751
7.11984152496214	8.29416911836493	8.45319644146129	8.42930652044652
8.02755393491409	7.03157516285998	7.72393110597666	7.81210368272515
7.78475218199953	6.13849036677649	7.94710696205454	8.39162204475618
7.58486716007266	7.06810103888509	7.2007119549684	7.37792113417961
7.76517664366277	6.36508277664222	7.39237124876524	8.01692347461365
7.13211985474057	7.25170097722448	7.7724749579347	7.92946063336331
7.89693523183365	7.42263379920438	7.77776925760421	8.0893319701417
7.19199131071797	7.74294904312344	8.45979917628554	8.18998818358653

7.26628509306529	7.68028500834127	8.06439048375562	7.95182844639177
7.27738280194862	7.42665805576317	8.22417092779239	7.62526890277276
7.95683593745961	8.20326062216672	8.92580344884632	7.71953756398908
7.93247138906041	8.7843080728845	8.0730294020295	8.09604219173126
7.9807958364038	7.61124336391162	7.36449221618981	7.48915637547404
7.68410503301546	6.97773701123243	6.68501211668436	7.61596116054653
7.60262363087681	7.51246520943344	6.97041433554661	7.73681555542092
7.6631047344029	7.54984281924748	7.06005154803129	7.42664717399603
7.29932535482929	7.55352786605533	7.32163673722801	7.94068788878657
6.97739725994193	7.32252231441417	7.81429513362933	7.03099865982538
7.07602529266965	7.33542280312198	7.98422013507164	7.10604936508664
6.77303876143574	7.27420837663405	7.37151264386758	7.28113607164473
7.52093983328311	7.26074611682748	7.05133433093018	7.37240412921204
7.13211985474057	7.49723873520879	7.25486772501531	7.03099865982538
7.01102803782321	7.61829812144557	7.32163673722801	7.44778594105418
6.86418664949394	7.56452686500459	6.71843511822181	7.38341712193446
7.42440248787941	7.20560456263502	7.34322359943616	7.78328998522042
6.73329258264299	6.68701087152958	7.5676551660462	7.74537756892434
7.7453318322659	8.25849014288712	7.14444373532166	7.09938563480562
7.43930136425875	8.76695450613423	7.75110130714213	7.47892445286308
7.58932680826336	8.04734247871829	7.07732953946312	7.84035315952933
6.66732932026463	7.97499163449361	7.92818610015731	7.46861944447134
7.03737936477016	7.75259921315716	7.8649212066993	6.98835432241688
6.58932680826336	7.88133252727936	7.29972196079957	7.09269098223162
7.65461826851508	7.77170803610486	8.11964034432109	7.31604168659293
7.34223394629858	7.43467304882729	7.4987933079014	7.7496395957785
7.76122945914611	7.72998091049514	7.00694021157172	7.42131372969299
7.63749409072387	8.55720352419873	7.41293257124756	6.95182844639177
8.26070406080198	7.99951395362768	7.39925763435048	7.42131372969299
7.69656003158838	7.73323388829023	6.29972196079957	6.92192696067791
8.3155659958365	7.84570861754864	6.54299311181193	6.53432269152217
7.87152862483511	7.86065895901461	6.97963286557599	7.37792113417961
7.23247038007304	7.06293933376931	6.63920842707124	6.59242564608574
7.40429491443084	7.60059611971211	7.44656334912884	6.57331682313804
7.53487902424098	7.32252231441417	7.11127687138646	6.60188597533481
7.32093916793535	8.09868935871851	7.42647910308551	7.0239782321476
7.99094379177988	8.45845427478334	7.16074554765076	7.64370615102944
6.90052849655766	8.02893044160441	7.11127687138646	7.07920775286799
6.82690563341448	5.52378052266128	8.2007119549684	7.24536494062291
7.27738280194862	6.46237997799714	7.16882789394862	6.89139259252979
7.72115201977078	8.28975720348656	8.69623937210762	7.48915637547404
7.94991652351982	6.64617715402101	9.3556683658028	7.58767211611511
7.76911305822194	7.88133252727936	6.29972196079957	7.35572538806541
6.63318109785278	7.42665805576317	6.8649212066993	7.20868427729644
8.07602529266964	7.29637002655821	7.80395318940787	7.68434813552678
7.19784403696341	7.45845427478334	7.2007119549684	7.56366665310432
7.22675672887865	7.16281969613823	6.63920842707124	7.94441097482928
7.47347847957684	7.44264375993587	7.13622322851669	8.11268245741908
6.92190214735023	8.46433883103519	7.58587892466511	6.81210368272515
8.33693964662907	7.63578554817441	7.05133433093018	7.31604168659293
8.88610142592238	8.07580913402035	7.45319644146129	7.68879404055797
8.10434518863897	8.19856128563091	6.46637183020902	6.75388906877986
8.29932535482929	8.40642357202312	7.86988745959264	6.76235064752805
7.64608158656059	7.65306353960625	7.51156060397528	7.21486285222556
8.21526109004082	7.36927057360565	6.97041433554661	7.227141182004
7.60702880999682	7.26973490005474	7.66229204018428	8.0239782321476
7.55325755355583	8.41859828596922	8.26621269860932	8.36130642032871
8.34750888833926	8.38176151778885	7.44656334912884	7.86806008869917
8.11056371137413	8.32468042258158	8.02486211956899	8.29286536422683
7.58039368349335	7.47797683304815	7.26997461740552	7.54417047747852

7.58932680826336	7.59702950469192	7.36449221618981	7.25138872825068
7.65461826851508	8.31168308205271	7.69063666587824	7.41596049519878
7.54409755427036	7.5608698413965	7.65079640134645	7.83233816646521
7.89693523183365	7.64272159538479	7.43320496627382	7.48404948504095
7.63318109785278	7.62881599335957	7.66229204018428	7.66191074640703
8.10434518863897	7.67013705296519	7.6275266220236	7.90291884591082
7.67574156008083	7.57906295816247	7.74029455218555	7.91814532013548
7.57140490026609	7.80918274152353	7.88468446152072	7.77912636027888
7.44423365980098	8.86659598402246	8.5336350571137	7.59242564608574
8.38902815777752	8.02893044160441	7.65079640134645	7.44253021560268
8.22389139518426	8.21494242721436	7.74029455218555	6.71953756398908

8919-Osteome	8911-Osteome	8909-Osteome	8908-Osteome
0.720397952037781	1.57552882987259	1.82338670891689	1.73680164359436
-1.27960204796222	1.57552882987259	2.82338670891689	1.73680164359436
0.305360452758937	1.57552882987259	4.14531480380425	1.73680164359436
0.720397952037781	3.16049133059375	4.40834920963804	4.32176414431551
1.04232604692514	1.57552882987259	3.82338670891689	4.05872973848172
0.720397952037781	3.16049133059375	1.82338670891689	1.73680164359436
1.52775287409539	2.57552882987259	1.82338670891689	1.73680164359436
2.04232604692514	1.57552882987259	1.82338670891689	2.73680164359436
1.30536045275894	3.57552882987259	1.82338670891689	1.73680164359436
1.04232604692514	3.57552882987259	2.82338670891689	1.73680164359436
0.720397952037781	7.78498219550154	4.63074163097449	5.32176414431551
2.42083767017887	4.89745692475995	1.82338670891689	4.54415656565196
1.52775287409539	4.7454538313149	2.82338670891689	5.54415656565196
1.04232604692514	3.89745692475995	3.40834920963804	4.90672664503667
2.04232604692514	4.38288375193019	3.40834920963804	2.73680164359436
1.30536045275894	3.57552882987259	4.9933117103592	4.05872973848172
1.30536045275894	3.57552882987259	4.9933117103592	4.90672664503667
1.04232604692514	3.57552882987259	5.63074163097449	5.05872973848172
2.96832546548137	4.57552882987259	5.14531480380425	5.05872973848172
3.11271537481654	4.16049133059375	3.82338670891689	5.05872973848172
3.17982957067508	4.57552882987259	2.82338670891689	5.98472915703794
2.6272885476463	4.16049133059375	2.82338670891689	4.73680164359436
2.96832546548137	4.57552882987259	3.82338670891689	5.05872973848172
2.6272885476463	3.89745692475995	3.40834920963804	4.54415656565196
3.36425414181251	5.03496044850989	5.63074163097449	5.19623326223166
3.24395990809479	4.57552882987259	5.28281832755419	6.12911906637312
3.47528545420125	5.48241942548111	4.40834920963804	5.32176414431551
3.17982957067508	4.7454538313149	5.28281832755419	5.8242644848447
3.11271537481654	3.89745692475995	5.91084955016723	5.8242644848447
2.42083767017887	4.57552882987259	5.40834920963804	6.26036359965137
3.11271537481654	4.89745692475995	4.40834920963804	4.90672664503667
3.42083767017887	4.57552882987259	3.40834920963804	5.32176414431551
0.720397952037781	4.89745692475995	5.40834920963804	4.54415656565196
1.04232604692514	3.16049133059375	5.73027730452541	5.73680164359436
1.04232604692514	3.16049133059375	5.63074163097449	6.05872973848172
1.30536045275894	4.38288375193019	4.40834920963804	5.19623326223166
1.30536045275894	3.16049133059375	4.40834920963804	5.43724136173545
2.04232604692514	2.57552882987259	5.40834920963804	4.73680164359436
1.30536045275894	2.57552882987259	4.63074163097449	3.73680164359436
1.04232604692514	1.57552882987259	5.63074163097449	4.05872973848172
1.89032295348009	2.57552882987259	6.07131422236047	5.05872973848172
1.89032295348009	3.89745692475995	5.28281832755419	5.64369223920288
2.04232604692514	3.16049133059375	4.14531480380425	4.90672664503667
-0.279602047962219	3.16049133059375	5.40834920963804	5.54415656565196
2.17982957067508	3.16049133059375	5.52382642705798	5.54415656565196
1.52775287409539	3.89745692475995	4.63074163097449	4.54415656565196
0.305360452758937	2.57552882987259	4.14531480380425	4.73680164359436
2.04232604692514	3.16049133059375	4.14531480380425	2.73680164359436
2.6272885476463	3.16049133059375	4.82338670891689	5.32176414431551
1.04232604692514	2.57552882987259	3.82338670891689	3.73680164359436
1.04232604692514	2.57552882987259	3.40834920963804	1.73680164359436
1.04232604692514	2.57552882987259	4.63074163097449	3.73680164359436
1.72039795203778	1.57552882987259	3.40834920963804	3.73680164359436
-1.27960204796222	4.38288375193019	5.28281832755419	3.73680164359436
0.720397952037781	3.57552882987259	3.82338670891689	4.05872973848172
-0.279602047962219	3.16049133059375	4.82338670891689	4.54415656565196
0.305360452758937	3.57552882987259	5.28281832755419	3.73680164359436
-0.279602047962219	2.57552882987259	4.82338670891689	1.73680164359436
-1.27960204796222	2.57552882987259	3.40834920963804	4.05872973848172

2.30536045275894	3.16049133059375	3.40834920963804	3.73680164359436
3.04232604692514	1.57552882987259	4.63074163097449	5.43724136173545
3.17982957067508	3.16049133059375	3.82338670891689	4.54415656565196
4.92985131766673	1.57552882987259	5.52382642705798	4.54415656565196
4.11271537481654	4.57552882987259	5.40834920963804	5.98472915703794
3.72039795203778	3.16049133059375	5.14531480380425	1.73680164359436
2.72039795203778	3.16049133059375	5.28281832755419	4.32176414431551
4.36425414181251	3.16049133059375	4.40834920963804	3.73680164359436
1.30536045275894	4.16049133059375	5.52382642705798	4.54415656565196
2.42083767017887	4.57552882987259	5.14531480380425	5.19623326223166
2.80786079328812	4.89745692475995	6.07131422236047	5.43724136173545
2.42083767017887	5.16049133059375	5.28281832755419	5.73680164359436
3.24395990809479	4.7454538313149	5.52382642705798	5.19623326223166
6.4346434697039	5.27596854801368	3.82338670891689	5.43724136173545
5.73162520746104	8.1902386739878	4.82338670891689	6.90672664503667
6.51481381838789	5.57552882987259	4.14531480380425	6.26036359965137
5.52775287409538	6.33041633203606	4.9933117103592	6.05872973848172
5.12978888817548	6.48241942548111	4.82338670891689	5.98472915703794
5.24395990809479	5.96784625265135	5.82338670891689	6.78119576295281
5.54057691445297	5.66299167112293	4.63074163097449	6.8242644848447
5.4346434697039	6.66299167112293	5.40834920963804	5.73680164359436
5.17982957067508	6.38288375193019	5.40834920963804	5.73680164359436
5.67459426242466	5.82345634331618	5.82338670891689	5.54415656565196
5.73162520746104	5.38288375193019	5.40834920963804	5.98472915703794
5.67459426242466	5.89745692475995	5.40834920963804	5.54415656565196
5.89032295348009	5.66299167112293	4.40834920963804	5.32176414431551
5.90030704205272	5.57552882987259	4.82338670891689	5.90672664503667
6.4346434697039	5.38288375193019	6.07131422236047	6.19623326223166
6.21225104836746	6.0990907859296	6.46724289869161	6.94625500922331
6.23609779032182	6.27596854801368	6.46724289869161	5.98472915703794
6.27498680371542	5.89745692475995	6.68136770404446	5.05872973848172
6.34244977149416	6.43350982500016	6.63074163097449	6.05872973848172
5.47528545420125	5.7454538313149	5.40834920963804	5.32176414431551
6.06024795492241	6.43350982500016	5.28281832755419	5.32176414431551
6.34244977149416	5.48241942548111	6.28281832755419	5.8242644848447
6.12127738831997	5.7454538313149	5.82338670891689	5.73680164359436
5.48858227681471	5.16049133059375	6.21570413169565	5.54415656565196
5.56588800298216	6.0990907859296	5.73027730452541	5.64369223920288
5.19613138300418	5.96784625265135	6.14531480380425	5.43724136173545
5.44831840660098	3.89745692475995	5.28281832755419	6.19623326223166
5.60304100139962	3.89745692475995	4.9933117103592	6.12911906637312
5.65113528960067	3.89745692475995	4.63074163097449	5.73680164359436
5.16334144788651	3.16049133059375	4.40834920963804	5.90672664503667
5.19613138300418	3.89745692475995	4.14531480380425	5.54415656565196
5.99652235731202	3.57552882987259	5.73027730452541	4.73680164359436
6.18800350212078	4.7454538313149	5.91084955016723	4.90672664503667
5.67459426242466	5.48241942548111	5.52382642705798	4.90672664503667
5.21225104836746	5.82345634331618	4.82338670891689	6.32176414431551
4.78648714249555	5.03496044850989	5.28281832755419	5.8242644848447
5.32031079422491	4.7454538313149	5.91084955016723	5.98472915703794
5.09543738338471	5.48241942548111	4.14531480380425	4.90672664503667
4.60304100139962	4.38288375193019	5.14531480380425	5.32176414431551
5.72039795203778	4.89745692475995	5.52382642705798	4.90672664503667
5.30536045275894	4.7454538313149	4.82338670891689	4.73680164359436
5.12978888817548	5.03496044850989	5.14531480380425	4.32176414431551
4.98718449273268	5.16049133059375	5.28281832755419	5.32176414431551
5.54057691445297	5.27596854801368	4.40834920963804	4.90672664503667
4.57837894716535	5.38288375193019	4.9933117103592	5.32176414431551
4.44831840660098	6.21938501964731	4.9933117103592	5.19623326223166
4.52775287409538	5.27596854801368	5.9933117103592	5.32176414431551

4.36425414181251	4.38288375193019	5.91084955016723	4.54415656565196
4.76479207139623	5.03496044850989	5.14531480380425	6.32176414431551
4.57837894716535	4.16049133059375	6.3469486649739	5.05872973848172
5.25955676314581	3.89745692475995	5.82338670891689	4.73680164359436
4.96832546548137	4.38288375193019	5.63074163097449	5.19623326223166
5.11271537481654	4.7454538313149	2.82338670891689	3.73680164359436
4.82892240881595	4.16049133059375	2.82338670891689	3.73680164359436
4.76479207139623	5.57552882987259	4.9933117103592	3.32176414431551
4.9102225109178	4.7454538313149	4.14531480380425	4.05872973848172
5.17982957067508	5.7454538313149	4.9933117103592	4.54415656565196
4.33510779615299	5.38288375193019	2.82338670891689	4.73680164359436
5.21225104836746	6.27596854801368	3.40834920963804	5.19623326223166
5.29025356036873	4.7454538313149	2.82338670891689	4.54415656565196
5.81843003499831	4.38288375193019	3.40834920963804	4.73680164359436
6.0513148301524	1.57552882987259	5.40834920963804	6.98472915703794
5.25955676314581	3.57552882987259	9.38562913313796	5.73680164359436
4.00580017090003	4.16049133059375	9.91084955016723	5.90672664503667
4.24395990809479	6.93308083449067	8.0132112677969	7.5696916577591
5.80786079328812	7.1902386739878	6.68136770404446	5.8242644848447
4.74276576506624	6.86093104873484	6.52382642705798	5.64369223920288
4.94921664253366	5.89745692475995	8.37797556059452	5.54415656565196
5.11271537481654	5.7454538313149	7.65627672308163	6.19623326223166
6.07794995665587	7.27596854801368	8.09017324961179	5.54415656565196
6.44831840660098	6.89745692475995	6.77758301930376	6.64369223920288
5.70908263880995	6.16049133059375	6.91084955016723	6.69099795398123
5.83933902476129	6.38288375193019	6.86778082827534	5.90672664503667
4.42083767017887	4.57552882987259	7.37797556059453	5.43724136173545
4.89032295348009	6.03496044850989	8.05220539941277	5.43724136173545
5.52775287409538	5.16049133059375	6.63074163097449	6.78119576295281
4.27498680371542	4.16049133059375	6.21570413169565	6.73680164359436
4.87014507154246	5.38288375193019	6.14531480380425	5.98472915703794
4.94921664253366	4.89745692475995	6.28281832755419	5.43724136173545
4.92985131766673	5.27596854801368	6.73027730452541	6.32176414431551
4.96832546548137	5.38288375193019	4.9933117103592	5.43724136173545
4.67459426242466	5.27596854801368	5.14531480380425	5.54415656565196
4.84968096898275	5.48241942548111	5.52382642705798	5.73680164359436
5.65113528960067	5.82345634331618	5.40834920963804	5.64369223920288
5.06024795492241	5.82345634331618	5.52382642705798	5.73680164359436
4.6272885476463	6.0990907859296	5.40834920963804	5.64369223920288
4.52775287409538	5.27596854801368	5.9933117103592	6.26036359965137
4.50175766556244	4.7454538313149	5.82338670891689	5.19623326223166
5.00580017090003	4.57552882987259	7.63074163097449	6.32176414431551
4.24395990809479	5.03496044850989	6.68136770404446	6.05872973848172
4.44831840660098	4.7454538313149	7.57827421108036	5.43724136173545
4.74276576506624	5.38288375193019	6.73027730452541	6.86608466053932
5.34975457211739	3.89745692475995	6.28281832755419	6.43724136173545
5.16334144788651	4.89745692475995	6.68136770404446	6.54415656565196
3.72039795203778	1.57552882987259	4.9933117103592	4.90672664503667
3.11271537481654	3.16049133059375	5.40834920963804	5.32176414431551
3.76479207139623	3.89745692475995	4.63074163097449	5.05872973848172
4.21225104836746	3.89745692475995	5.52382642705798	5.32176414431551
3.11271537481654	3.57552882987259	4.14531480380425	4.32176414431551
2.6272885476463	4.16049133059375	4.63074163097449	4.54415656565196
2.89032295348009	4.7454538313149	4.9933117103592	5.05872973848172
3.72039795203778	1.57552882987259	3.82338670891689	4.05872973848172
2.30536045275894	2.57552882987259	4.40834920963804	2.73680164359436
3.17982957067508	2.57552882987259	3.40834920963804	3.32176414431551
4.6976778755377	1.57552882987259	1.82338670891689	3.73680164359436
2.72039795203778	5.03496044850989	2.82338670891689	1.73680164359436
3.52775287409538	3.89745692475995	4.14531480380425	1.73680164359436

1.89032295348009	1.57552882987259	2.82338670891689	2.73680164359436
1.30536045275894	2.57552882987259	3.40834920963804	2.73680164359436
1.72039795203778	3.16049133059375	1.82338670891689	2.73680164359436
1.52775287409539	1.57552882987259	3.82338670891689	5.54415656565196
1.30536045275894	2.57552882987259	5.14531480380425	4.73680164359436
2.30536045275894	2.57552882987259	5.73027730452541	5.19623326223166
2.6272885476463	3.16049133059375	5.28281832755419	5.32176414431551
0.720397952037781	3.89745692475995	4.40834920963804	4.90672664503667
2.30536045275894	2.57552882987259	4.9933117103592	5.54415656565196
1.89032295348009	4.16049133059375	5.28281832755419	4.90672664503667
1.72039795203778	3.57552882987259	1.82338670891689	4.05872973848172
2.72039795203778	2.57552882987259	3.82338670891689	4.73680164359436
5.4346434697039	3.57552882987259	3.40834920963804	4.32176414431551
4.24395990809479	3.89745692475995	4.14531480380425	5.32176414431551
2.89032295348009	5.82345634331618	6.73027730452541	6.38065783336908
4.21225104836746	6.52972514025947	6.28281832755419	5.73680164359436
4.92985131766673	6.0990907859296	7.3469486649739	6.64369223920288
3.96832546548137	6.16049133059375	6.86778082827534	6.26036359965137
4.67459426242466	6.33041633203606	6.46724289869161	5.8242644848447
1.72039795203778	6.38288375193019	5.28281832755419	6.12911906637312
2.72039795203778	5.27596854801368	3.82338670891689	3.73680164359436
3.72039795203778	4.89745692475995	5.40834920963804	4.90672664503667
4.14666270673988	5.96784625265135	5.14531480380425	5.43724136173545
4.82892240881595	6.61992294923104	5.14531480380425	5.73680164359436
4.52775287409538	6.96784625265135	4.9933117103592	4.73680164359436
3.47528545420125	5.03496044850989	6.14531480380425	5.73680164359436
3.6272885476463	5.16049133059375	6.3469486649739	5.98472915703794
4.04232604692514	4.89745692475995	5.82338670891689	5.98472915703794
3.67459426242466	4.89745692475995	5.91084955016723	5.90672664503667
2.80786079328812	4.57552882987259	5.40834920963804	6.19623326223166
3.76479207139623	4.7454538313149	5.91084955016723	6.12911906637312
3.57837894716535	4.38288375193019	5.28281832755419	6.12911906637312
3.76479207139623	4.38288375193019	5.9933117103592	5.43724136173545
3.04232604692514	4.16049133059375	5.14531480380425	5.54415656565196
3.6272885476463	4.57552882987259	5.73027730452541	4.90672664503667
3.57837894716535	4.57552882987259	5.28281832755419	5.32176414431551
2.96832546548137	4.38288375193019	4.9933117103592	5.05872973848172
3.80786079328812	4.38288375193019	5.52382642705798	5.05872973848172
3.84968096898275	4.7454538313149	4.63074163097449	4.32176414431551
3.67459426242466	4.89745692475995	4.14531480380425	5.05872973848172
3.30536045275894	4.89745692475995	3.82338670891689	5.05872973848172
3.92985131766673	5.16049133059375	4.9933117103592	6.19623326223166
3.30536045275894	5.16049133059375	4.63074163097449	5.19623326223166
4.21225104836746	4.16049133059375	5.40834920963804	4.73680164359436
3.72039795203778	3.89745692475995	5.73027730452541	5.54415656565196
3.52775287409538	3.16049133059375	5.82338670891689	4.90672664503667
5.09543738338471	3.89745692475995	5.28281832755419	5.54415656565196
4.14666270673988	5.03496044850989	5.52382642705798	4.73680164359436
4.72039795203778	5.16049133059375	5.73027730452541	6.26036359965137
4.87014507154246	7.70481184681756	6.28281832755419	5.43724136173545
4.00580017090003	4.7454538313149	4.82338670891689	5.05872973848172
4.55328796620252	4.7454538313149	5.9933117103592	5.8242644848447
4.14666270673988	6.48241942548111	4.40834920963804	6.32176414431551
5.56588800298216	5.03496044850989	4.9933117103592	6.12911906637312
5.29025356036873	5.03496044850989	4.82338670891689	5.8242644848447
4.57837894716535	5.03496044850989	4.82338670891689	5.54415656565196
4.96832546548137	4.7454538313149	4.40834920963804	5.32176414431551
4.87014507154246	5.03496044850989	4.40834920963804	5.90672664503667
2.80786079328812	5.16049133059375	3.82338670891689	5.98472915703794
5.14666270673988	5.66299167112293	5.14531480380425	5.54415656565196

3.72039795203778	5.7454538313149	5.82338670891689	5.43724136173545
5.04232604692514	6.0990907859296	4.9933117103592	5.98472915703794
4.42083767017887	5.89745692475995	5.52382642705798	5.98472915703794
4.55328796620252	5.16049133059375	6.3469486649739	5.90672664503667
3.96832546548137	5.48241942548111	6.21570413169565	6.49168914575783
4.11271537481654	5.38288375193019	5.82338670891689	5.98472915703794
4.6976778755377	4.57552882987259	6.63074163097449	6.19623326223166
4.9102225109178	5.89745692475995	6.68136770404446	5.98472915703794
4.11271537481654	5.38288375193019	5.40834920963804	6.64369223920288
4.36425414181251	4.89745692475995	4.40834920963804	5.43724136173545
3.57837894716535	4.57552882987259	5.52382642705798	5.43724136173545
3.89032295348009	4.38288375193019	5.52382642705798	5.64369223920288
3.42083767017887	3.57552882987259	6.57827421108036	4.73680164359436
4.72039795203778	4.38288375193019	7.68136770404446	3.73680164359436
4.78648714249555	5.48241942548111	7.46724289869161	4.73680164359436
3.6272885476463	4.7454538313149	5.82338670891689	5.32176414431551
3.17982957067508	4.57552882987259	5.63074163097449	5.05872973848172
3.57837894716535	4.89745692475995	5.52382642705798	6.43724136173545
4.14666270673988	4.57552882987259	6.73027730452541	6.64369223920288
4.39282329400928	4.89745692475995	5.91084955016723	4.73680164359436
4.55328796620252	4.7454538313149	5.14531480380425	4.73680164359436
4.14666270673988	4.57552882987259	5.91084955016723	5.05872973848172
3.6272885476463	3.16049133059375	4.9933117103592	5.43724136173545
3.72039795203778	4.57552882987259	5.82338670891689	5.64369223920288
3.72039795203778	3.16049133059375	5.82338670891689	5.64369223920288
4.21225104836746	3.89745692475995	4.40834920963804	5.32176414431551
5.9102225109178	3.89745692475995	5.63074163097449	4.73680164359436
4.80786079328812	4.57552882987259	5.40834920963804	4.32176414431551
4.50175766556244	5.03496044850989	4.9933117103592	4.73680164359436
4.42083767017887	3.16049133059375	4.63074163097449	4.05872973848172
4.84968096898275	4.57552882987259	4.40834920963804	5.43724136173545
4.30536045275894	3.89745692475995	5.40834920963804	4.05872973848172
4.24395990809479	5.66299167112293	4.14531480380425	4.05872973848172
3.84968096898275	3.89745692475995	5.40834920963804	4.32176414431551
3.67459426242466	4.89745692475995	5.14531480380425	5.32176414431551
2.89032295348009	4.89745692475995	10.6435656713321	4.54415656565196
2.6272885476463	3.89745692475995	9.30720248618114	3.32176414431551
2.04232604692514	2.57552882987259	7.57827421108036	4.54415656565196
2.17982957067508	4.38288375193019	7.65627672308163	7.12911906637312
3.17982957067508	4.57552882987259	5.14531480380425	5.64369223920288
2.80786079328812	4.16049133059375	3.40834920963804	2.73680164359436
1.89032295348009	1.57552882987259	3.40834920963804	3.32176414431551
1.52775287409539	4.16049133059375	3.82338670891689	4.32176414431551
1.72039795203778	3.16049133059375	2.82338670891689	3.32176414431551
2.80786079328812	2.57552882987259	2.82338670891689	3.73680164359436
2.89032295348009	1.57552882987259	3.82338670891689	2.73680164359436
2.6272885476463	2.57552882987259	2.82338670891689	2.73680164359436
2.96832546548137	2.57552882987259	5.73027730452541	5.64369223920288
1.72039795203778	1.57552882987259	5.14531480380425	4.73680164359436
2.6272885476463	1.57552882987259	4.82338670891689	4.32176414431551
2.72039795203778	1.57552882987259	5.40834920963804	4.05872973848172
3.04232604692514	1.57552882987259	4.63074163097449	4.32176414431551
3.11271537481654	1.57552882987259	3.82338670891689	5.19623326223166
1.89032295348009	1.57552882987259	4.14531480380425	4.32176414431551
2.6272885476463	3.16049133059375	5.73027730452541	4.54415656565196
2.17982957067508	2.57552882987259	4.82338670891689	4.54415656565196
1.72039795203778	2.57552882987259	5.52382642705798	4.32176414431551
2.6272885476463	2.57552882987259	4.40834920963804	4.05872973848172
2.04232604692514	3.57552882987259	3.82338670891689	4.05872973848172
2.72039795203778	3.57552882987259	4.82338670891689	4.73680164359436

2.52775287409539	3.16049133059375	4.40834920963804	4.54415656565196
1.72039795203778	3.57552882987259	4.63074163097449	3.32176414431551
1.52775287409539	3.16049133059375	3.82338670891689	4.32176414431551
0.720397952037781	4.38288375193019	4.40834920963804	5.05872973848172
2.30536045275894	3.57552882987259	3.40834920963804	5.05872973848172
1.89032295348009	3.89745692475995	4.14531480380425	5.43724136173545
1.04232604692514	2.57552882987259	5.14531480380425	5.05872973848172
1.89032295348009	3.57552882987259	5.14531480380425	4.73680164359436
3.47528545420125	4.89745692475995	3.82338670891689	4.90672664503667
2.96832546548137	4.57552882987259	4.14531480380425	4.05872973848172
2.04232604692514	4.16049133059375	6.73027730452541	4.90672664503667
2.72039795203778	4.57552882987259	5.52382642705798	5.19623326223166
2.89032295348009	2.57552882987259	4.82338670891689	4.54415656565196
2.17982957067508	3.89745692475995	5.28281832755419	4.73680164359436
1.04232604692514	3.57552882987259	4.63074163097449	4.54415656565196
3.52775287409538	2.57552882987259	4.63074163097449	3.73680164359436
2.42083767017887	3.57552882987259	4.63074163097449	3.73680164359436
3.17982957067508	4.7454538313149	3.40834920963804	4.73680164359436
3.6272885476463	4.16049133059375	5.14531480380425	4.05872973848172
3.6272885476463	3.89745692475995	4.63074163097449	3.73680164359436
3.24395990809479	3.57552882987259	4.63074163097449	4.54415656565196
3.30536045275894	2.57552882987259	5.9933117103592	4.32176414431551
3.47528545420125	2.57552882987259	5.52382642705798	4.73680164359436
1.72039795203778	4.89745692475995	6.07131422236047	4.54415656565196
2.04232604692514	4.89745692475995	6.07131422236047	5.19623326223166
1.72039795203778	6.03496044850989	5.91084955016723	5.19623326223166
1.30536045275894	3.57552882987259	4.82338670891689	4.05872973848172
1.04232604692514	3.16049133059375	4.9933117103592	4.90672664503667
2.42083767017887	4.7454538313149	6.07131422236047	5.05872973848172
2.80786079328812	3.16049133059375	5.52382642705798	5.54415656565196
3.04232604692514	3.89745692475995	5.63074163097449	5.19623326223166
2.89032295348009	3.57552882987259	6.07131422236047	5.19623326223166
2.96832546548137	3.57552882987259	5.73027730452541	5.73680164359436
3.17982957067508	4.57552882987259	5.73027730452541	5.73680164359436
3.30536045275894	4.7454538313149	5.40834920963804	5.43724136173545
2.30536045275894	5.03496044850989	5.14531480380425	5.64369223920288
2.17982957067508	5.03496044850989	3.82338670891689	4.90672664503667
3.57837894716535	5.48241942548111	4.40834920963804	5.43724136173545
3.36425414181251	5.03496044850989	4.82338670891689	4.90672664503667
3.42083767017887	5.38288375193019	5.73027730452541	5.90672664503667
3.36425414181251	5.16049133059375	5.73027730452541	5.64369223920288
1.30536045275894	4.16049133059375	5.63074163097449	4.54415656565196
2.04232604692514	4.16049133059375	4.63074163097449	4.05872973848172
2.52775287409539	3.57552882987259	5.63074163097449	5.19623326223166
1.89032295348009	4.38288375193019	6.28281832755419	5.64369223920288
2.6272885476463	4.16049133059375	6.14531480380425	5.54415656565196
3.17982957067508	4.89745692475995	5.73027730452541	6.12911906637312
2.96832546548137	5.48241942548111	5.82338670891689	5.90672664503667
4.04232604692514	5.16049133059375	6.28281832755419	5.8242644848447
3.42083767017887	5.03496044850989	5.40834920963804	5.43724136173545
2.04232604692514	4.57552882987259	5.52382642705798	4.05872973848172
2.17982957067508	4.57552882987259	5.40834920963804	4.32176414431551
3.11271537481654	5.16049133059375	4.82338670891689	5.05872973848172
2.96832546548137	5.03496044850989	4.82338670891689	4.54415656565196
3.17982957067508	5.03496044850989	3.82338670891689	5.19623326223166
3.11271537481654	5.57552882987259	3.82338670891689	4.05872973848172
2.30536045275894	4.16049133059375	4.9933117103592	4.54415656565196
3.17982957067508	3.16049133059375	4.40834920963804	4.32176414431551
3.76479207139623	3.89745692475995	4.9933117103592	4.32176414431551
15.2670814409256	16.9066907101175	13.7459711116306	16.5154948268182

14.4721512034771	14.09541075237	9.37797556059452	12.1461925797321
13.2836538188201	14.4809158348907	11.9603778209971	13.9120392938854
13.4177456565372	11.2863352635719	12.6451606908875	12.2681831041107
11.1276657162825	10.7529483678618	12.0242853139553	10.6224980169338
12.6799471602684	10.7849821955015	12.2527934504309	10.7619412058729
10.2272013898334	8.97640826615477	5.14531480380425	6.38065783336908
10.216252978925	6.57552882987259	7.18093871353497	7.94625500922331
9.66217919431631	7.35688854339725	6.52382642705798	7.43724136173545
9.22918311410229	5.7454538313149	6.68136770404446	7.88654876309904
7.17982957067508	10.0059813815381	8.61780257526699	9.35151148770957
9.60685664774149	10.6416180203304	7.8006666324168	9.06771852170898
9.42430152548244	11.9374726036078	7.28281832755419	9.19623326223165
8.59230318969697	10.2690157873719	6.77758301930376	7.86608466053932
9.67095384908529	9.79469735033475	6.52382642705798	8.43724136173545
9.99419355125205	6.78498219550154	9.38562913313796	4.90672664503667
8.948013895452	7.87930957804969	11.0713142223605	7.40922698556585
9.73232401834459	8.0990907859296	12.4111642252451	7.75916945662281
7.59998120165057	7.82345634331618	9.84019499660344	9.48499449318382
10.0213224430141	9.76040417278087	7.73027730452541	7.73680164359436
9.47026737943462	9.67881663828461	9.52382642705798	9.77572063288666
9.53658163347012	9.78498219550154	8.95266972586185	10.7647076401642
9.65187418592457	10.0990907859296	8.21570413169565	8.79208407909555
9.05578830673171	9.58675608529584	8.49581205088838	8.66753898115724
9.14771079617275	10.0101570575093	8.74224994619148	9.30665725192531
9.83414011808697	9.05126226083899	8.55130716348009	8.84532610037253
9.76683990004509	9.41473261796953	8.84575452194534	8.6194446929562
8.90775002523828	8.64161802033036	9.02305905375325	8.07665164647898
9.14035812988567	8.31699581627374	9.13626966420124	7.84532610037253
8.99069327850982	8.93308083449067	8.36254552002492	8.12911906637312
8.57214699345384	8.90644570798721	8.98325804569528	8.16306639829646
8.84322594684545	9.01015705750931	8.46724289869161	9.05872973848172
8.81974576244097	9.47034659318053	9.29912013988329	8.74802889901761
9.8733165281744	8.91537883275721	9.98829363559258	8.81361724064519
8.72461841835598	9.28977434753871	9.72425351689764	7.66753898115724
8.31098500195282	7.59789664290104	9.53763222658301	7.5696916577591
8.78109388372534	7.55280875337251	9.24123922380279	7.94625500922331
9.43807437510418	9.75045451237327	8.55130716348009	7.98472915703794
8.70053752967694	8.63081126537378	7.18093871353497	8.27596045470239
9.036679483784	9.01847232572132	8.16323671180151	8.22865473992403
9.59614730345784	9.28977434753871	7.88947589937466	8.22865473992403
9.19308879128056	9.0990907859296	7.77758301930376	8.07665164647898
9.07684992225954	9.21938501964732	8.91084955016723	8.81361724064519
9.41475583925923	8.91537883275721	9.27459782074922	8.72548633036652
10.3714496432167	9.05126226083899	8.10878892777914	7.80289083405213
9.61899933944172	8.61992294923104	8.46724289869161	7.64369223920288
9.98776988330306	8.31699581627374	8.09017324961179	8.35151148770957
9.05019429025848	8.03496044850989	8.4527433289965	7.5696916577591
9.19207316642983	8.06738192620227	8.49581205088838	8.32176414431551
9.70481641083892	10.844655509022	7.18093871353497	9.14619257973206
9.27210621365847	10.5895493001875	8.37797556059452	9.16306639829646
9.26729241192542	8.56421351664476	9.0132112677969	7.43724136173545
9.59076267162119	8.56421351664476	9.71212995781515	9.32925868086244
9.12127738831996	9.01847232572132	9.31523980524656	8.88654876309904
10.6314153598348	8.06738192620227	9.07131422236047	8.02220386245661
10.6742306341831	8.98491976601029	10.2869110821881	8.22865473992403
8.8637811660276	9.16049133059375	12.7999508323322	13.6361585665175
10.4698484176077	9.77520117470895	12.7174465551909	10.4916891457578
8.73859813085101	9.95056826121952	11.9253623798661	11.3770465798167
11.5154229359992	10.2229872563275	10.588258299653	11.0129260488686
11.6123714710633	9.59233711755915	10.3508637149773	9.06771852170898

10.8390146413893	9.51216676887516	10.9240490479221	10.1880127554267
10.5001173071812	10.2690157873719	10.6047464224415	9.98946707604461
10.537781379935	10.08728148364	10.172114863148	8.89667298037275
8.9310692958234	9.05126226083899	12.2621785614951	10.3770465798167
11.6476203547624	9.36343138926402	10.7332797926869	9.30665725192531
11.7768663268091	10.8399714300992	8.73027730452541	10.0987454173296
10.4698484176077	9.79469735033475	8.70602975827873	9.10312385784017
10.4251661914004	8.30344928443579	10.0328400745458	10.1755934961726
6.04232604692514	5.03496044850989	15.8134906727744	5.73680164359436
8.86887453421606	3.16049133059375	4.9933117103592	4.05872973848172
8.01271958483982	7.00179358457469	7.37797556059453	4.32176414431551
8.74137789094199	5.16049133059375	7.18093871353497	5.8242644848447
7.57526633529802	5.27596854801368	6.21570413169565	7.54415656565196
7.53738157529316	5.82345634331618	6.40834920963804	6.43724136173545
7.03781056580265	5.82345634331618	6.46724289869161	6.12911906637312
7.36425414181251	5.38288375193019	6.63074163097449	6.73680164359436
7.53096958677893	7.13011768155023	6.91084955016723	7.16306639829646
7.09977631910904	6.82345634331618	6.63074163097449	7.05872973848172
6.82892240881595	7.86093104873484	7.60474642244155	7.5696916577591
8.72039795203778	8.16049133059375	5.63074163097449	6.38065783336908
7.95641214393787	8.71508018227138	6.40834920963804	7.38065783336908
8.07574904846259	8.34371315464952	6.07131422236047	7.46472209815756
5.59076267162119	8.45817187923443	6.63074163097449	6.90672664503667
6.70339152673209	9.33041633203606	4.14531480380425	6.54415656565196
6.23609779032182	8.76535338875261	5.82338670891689	5.73680164359436
5.99652235731202	7.30344928443579	4.9933117103592	4.54415656565196
6.06024795492241	5.89745692475995	5.40834920963804	5.54415656565196
5.54057691445297	6.16049133059375	5.52382642705798	5.32176414431551
6.69194150598855	5.38288375193019	4.9933117103592	5.32176414431551
6.81315509295763	7.57552882987259	5.28281832755419	6.64369223920288
5.9102225109178	8.2048854499522	4.9933117103592	4.90672664503667
6.17982957067508	4.16049133059375	5.28281832755419	6.43724136173545
5.98718449273268	4.57552882987259	5.63074163097449	6.38065783336908
7.22819259223648	7.03496044850989	6.86778082827534	7.19623326223166
7.78379303332629	7.59789664290104	5.63074163097449	7.40922698556585
7.762057103675	7.80434752036847	5.9933117103592	6.32176414431551
8.44320548320733	7.80434752036847	6.46724289869161	6.32176414431551
7.78917623002319	7.27596854801368	5.40834920963804	5.54415656565196
6.44149714074497	6.03496044850989	7.10878892777914	6.38065783336908
6.34244977149416	5.89745692475995	7.88947589937466	5.90672664503667
6.45510757226362	4.89745692475995	6.95266972586185	6.73680164359436
7.06912610626886	6.03496044850989	7.18093871353497	6.26036359965137
6.83933902476129	6.27596854801368	5.82338670891689	6.12911906637312
7.23215060580516	5.82345634331618	6.3469486649739	6.26036359965137
6.96832546548137	6.57552882987259	6.21570413169565	6.43724136173545
7.01501870092941	6.38288375193019	6.21570413169565	5.73680164359436
6.50175766556244	5.89745692475995	6.40834920963804	5.64369223920288
6.60914120093604	6.0990907859296	5.28281832755419	5.8242644848447
6.31285498930586	6.86093104873484	6.82338670891689	6.98472915703794
6.9102225109178	7.0990907859296	6.63074163097449	6.49168914575783
6.83933902476129	6.66299167112293	7.10878892777914	6.78119576295281
6.70908263880995	7.30344928443579	7.18093871353497	7.19623326223166
7.2940451395311	6.48241942548111	6.82338670891689	7.05872973848172
6.67459426242466	6.27596854801368	6.77758301930376	6.78119576295281
7.4346434697039	6.86093104873484	5.40834920963804	6.98472915703794
6.90527329494606	7.00179358457469	5.63074163097449	6.26036359965137
7.0104167989704	7.13011768155023	6.07131422236047	6.54415656565196
6.90527329494606	7.35688854339725	5.63074163097449	6.64369223920288
6.22024383912099	6.89745692475995	6.28281832755419	6.49168914575783
6.57214699345384	7.1902386739878	6.14531480380425	6.43724136173545

6.66291245737702	7.38288375193019	5.91084955016723	6.54415656565196
6.48858227681471	6.82345634331618	6.46724289869161	6.8242644848447
7.23609779032182	6.78498219550154	7.24965146361899	7.12911906637312
7.24787495809818	6.89745692475995	7.4380965530321	5.64369223920288
7.60304100139962	7.50626616743548	6.21570413169565	6.38065783336908
7.55644830709585	7.30344928443579	7.24965146361899	6.19623326223166
7.41388490953711	7.06738192620227	6.91084955016723	6.54415656565196
7.37503398056575	6.78498219550154	6.21570413169565	6.49168914575783
6.90030704205272	6.89745692475995	6.57827421108036	6.38065783336908
6.81843003499831	7.03496044850989	6.3469486649739	6.54415656565196
6.71475138889664	6.82345634331618	6.73027730452541	6.73680164359436
7.02417870021488	6.57552882987259	6.46724289869161	6.49168914575783
7.41736547827207	5.96784625265135	4.63074163097449	5.8242644848447
7.63926118931238	7.95056826121951	4.82338670891689	6.43724136173545
7.16334144788651	7.00179358457469	4.63074163097449	6.05872973848172
7.43120438573713	7.43350982500016	3.82338670891689	5.98472915703794
7.30536045275894	6.27596854801368	5.73027730452541	6.78119576295281
7.33878345429639	7.1902386739878	6.68136770404446	6.86608466053932
6.98718449273268	7.03496044850989	6.68136770404446	6.98472915703794
6.84451926386697	7.38288375193019	6.21570413169565	6.38065783336908
6.72039795203778	6.66299167112293	6.95266972586185	6.59478263872193
6.72039795203778	5.89745692475995	6.63074163097449	6.98472915703794
7.63926118931238	6.0990907859296	5.28281832755419	6.73680164359436
6.06912610626886	7.43350982500016	4.9933117103592	6.98472915703794
6.89532363453846	7.84231537056749	5.73027730452541	6.94625500922331
6.75931694133008	7.50626616743548	5.63074163097449	7.66753898115724
6.65703589104035	7.33041633203606	5.28281832755419	6.05872973848172
7.22422369003353	6.66299167112293	5.73027730452541	7.09435364821244
6.29782678007353	6.70481184681756	4.40834920963804	5.8242644848447
6.47528545420125	7.06738192620227	3.82338670891689	6.05872973848172
6.6272885476463	6.38288375193019	7.07131422236047	5.64369223920288
6.25177941255409	7.13011768155023	6.68136770404446	6.54415656565196
6.64521045564356	7.72527594937727	6.68136770404446	6.05872973848172
5.33510779615299	5.96784625265135	6.07131422236047	5.19623326223166
6.51481381838789	6.7454538313149	5.73027730452541	5.8242644848447
5.60304100139962	7.03496044850989	5.63074163097449	5.64369223920288
5.33510779615299	5.16049133059375	6.3469486649739	6.05872973848172
5.57837894716535	5.38288375193019	6.07131422236047	5.98472915703794
5.36425414181251	5.57552882987259	6.14531480380425	5.90672664503667
6.19613138300418	5.48241942548111	7.07131422236047	5.73680164359436
6.52129785195809	5.96784625265135	5.82338670891689	6.05872973848172
5.99652235731202	5.57552882987259	5.73027730452541	5.05872973848172
6.406898479221	5.16049133059375	5.52382642705798	6.94625500922331
6.29025356036873	5.82345634331618	6.52382642705798	6.05872973848172
5.67459426242466	5.96784625265135	7.10878892777914	6.12911906637312
6.52775287409538	5.89745692475995	7.14531480380425	6.05872973848172
6.29782678007353	5.89745692475995	6.3469486649739	6.59478263872193
6.25955676314581	6.21938501964731	6.82338670891689	5.90672664503667
6.44149714074497	6.16049133059375	6.82338670891689	5.90672664503667
6.18800350212078	5.82345634331618	6.73027730452541	6.78119576295281
5.46186493843893	6.43350982500016	6.07131422236047	5.90672664503667
5.95880269136286	6.33041633203606	5.91084955016723	5.90672664503667
5.85994930443657	5.96784625265135	6.46724289869161	5.8242644848447
5.77568038753897	6.52972514025947	6.28281832755419	6.38065783336908
5.67459426242466	6.48241942548111	6.14531480380425	5.64369223920288
6.12978888817548	6.7454538313149	6.40834920963804	6.19623326223166
5.88026928881617	6.89745692475995	6.68136770404446	5.98472915703794
5.27498680371542	6.38288375193019	6.14531480380425	6.86608466053932
5.09543738338471	5.82345634331618	6.40834920963804	6.26036359965137
6.36425414181251	6.78498219550154	6.82338670891689	6.05872973848172

6.12127738831997	5.96784625265135	6.82338670891689	6.26036359965137
6.39987805154323	5.89745692475995	6.52382642705798	6.26036359965137
6.51481381838789	5.89745692475995	5.14531480380425	5.64369223920288
5.87014507154246	6.16049133059375	4.82338670891689	5.8242644848447
6.20421372930204	5.03496044850989	5.9933117103592	5.90672664503667
6.12127738831997	5.7454538313149	5.63074163097449	5.32176414431551
6.35702257258143	5.82345634331618	6.14531480380425	5.19623326223166
6.31285498930586	5.16049133059375	3.82338670891689	6.59478263872193
6.09543738338471	5.03496044850989	4.40834920963804	5.90672664503667
6.42775708411866	5.48241942548111	5.40834920963804	6.19623326223166
6.73720623972434	6.03496044850989	4.82338670891689	6.64369223920288
6.28264037625885	6.52972514025947	4.40834920963804	6.12911906637312
6.01501870092941	6.43350982500016	5.14531480380425	5.8242644848447
5.77568038753897	6.70481184681756	5.14531480380425	5.8242644848447
6.01501870092941	6.03496044850989	4.82338670891689	6.19623326223166
6.22819259223648	4.57552882987259	5.52382642705798	5.43724136173545
6.35702257258143	4.89745692475995	4.63074163097449	6.12911906637312
6.07794995665587	5.96784625265135	5.40834920963804	5.05872973848172
5.88026928881617	5.38288375193019	4.82338670891689	5.64369223920288
6.03328090732214	5.48241942548111	5.91084955016723	5.43724136173545
5.95880269136286	5.48241942548111	5.52382642705798	5.43724136173545
5.92007029687415	6.21938501964731	4.9933117103592	5.43724136173545
5.70908263880995	4.57552882987259	5.73027730452541	5.90672664503667
5.30536045275894	3.89745692475995	5.28281832755419	5.8242644848447
5.06024795492241	4.38288375193019	6.14531480380425	5.90672664503667
5.93956647249994	5.03496044850989	6.14531480380425	6.26036359965137
6.13825046692368	4.57552882987259	5.9933117103592	5.8242644848447
6.24395990809479	4.7454538313149	5.82338670891689	5.43724136173545
6.32772826578739	5.27596854801368	8.4380965530321	6.54415656565196
5.37860943478958	5.89745692475995	7.46724289869161	7.64369223920288
6.74830394860767	6.43350982500016	8.95266972586185	5.32176414431551
7.33510779615299	5.7454538313149	9.02305905375325	7.26036359965137
6.46186493843893	7.24795417184409	7.55130716348009	6.98472915703794
6.51481381838789	7.80434752036847	7.37797556059453	6.78119576295281
6.55328796620252	7.06738192620227	8.18093871353497	6.59478263872193
5.29025356036873	5.16049133059375	6.57827421108036	3.73680164359436
5.54057691445297	4.7454538313149	6.21570413169565	6.05872973848172
5.81843003499831	4.7454538313149	7.57827421108036	5.43724136173545
6.15502617967451	5.38288375193019	6.86778082827534	6.26036359965137
6.74276576506624	3.89745692475995	7.14531480380425	6.19623326223166
6.44149714074497	3.89745692475995	5.14531480380425	5.90672664503667
7.13402588106195	5.03496044850989	6.14531480380425	6.05872973848172
5.51481381838789	6.43350982500016	6.86778082827534	5.43724136173545
5.29025356036873	5.82345634331618	6.77758301930376	6.59478263872193
6.13825046692368	5.96784625265135	5.73027730452541	4.54415656565196
5.67459426242466	5.96784625265135	4.63074163097449	6.32176414431551
5.76479207139623	6.16049133059375	4.9933117103592	6.19623326223166
6.28264037625885	6.16049133059375	5.73027730452541	5.8242644848447
6.17982957067508	5.38288375193019	6.68136770404446	5.73680164359436
6.39987805154323	5.96784625265135	5.63074163097449	5.43724136173545
6.22819259223648	5.48241942548111	6.07131422236047	6.32176414431551
6.55960174013472	5.48241942548111	6.68136770404446	6.12911906637312
7.25567332865858	6.16049133059375	6.28281832755419	6.43724136173545
6.77568038753897	6.57552882987259	6.73027730452541	6.54415656565196
7.29025356036873	6.52972514025947	6.21570413169565	6.8242644848447
6.32772826578739	6.16049133059375	5.91084955016723	6.78119576295281
6.09543738338471	5.82345634331618	4.9933117103592	6.49168914575783
6.49518501163895	6.0990907859296	5.73027730452541	6.19623326223166
6.88026928881617	5.7454538313149	4.82338670891689	6.05872973848172
7.03328090732214	5.27596854801368	5.63074163097449	5.8242644848447

6.6976778755377	6.16049133059375	4.9933117103592	7.38065783336908
6.85994930443657	7.24795417184409	5.14531480380425	6.78119576295281
7.78109388372534	4.7454538313149	8.05220539941277	6.73680164359436
7.22819259223648	5.48241942548111	5.9933117103592	5.64369223920288
6.25177941255409	6.27596854801368	5.91084955016723	5.90672664503667
7.86250500934033	4.38288375193019	6.68136770404446	7.12911906637312
5.19613138300418	4.57552882987259	5.40834920963804	5.8242644848447
7.44149714074497	6.38288375193019	9.09951111419113	6.38065783336908
5.47528545420125	5.03496044850989	10.3741334943001	5.43724136173545
7.3678563784927	4.16049133059375	7.63074163097449	8.291390495272
6.63328728826774	6.66299167112293	6.3469486649739	6.78119576295281
8.84063582937974	9.42101888081697	8.48159819166868	8.87635299599315
8.79721354908861	8.91537883275721	8.48159819166868	8.92662620247438
8.07794995665586	9.5585224045669	8.12716745709399	8.17974513944309
8.06024795492241	8.86093104873484	7.77758301930376	8.46472209815756
7.96595065829346	8.31699581627374	7.21570413169565	8.16306639829646
8.06691168520342	8.26202935705581	7.52382642705798	7.90672664503667
8.67168266700475	8.73540016665098	7.57827421108036	8.49168914575783
8.24787495809818	8.00179358457469	7.91084955016723	7.94625500922331
7.99652235731202	8.21938501964732	9.40834920963804	8.45104716126048
8.58613122288954	8.11468764098062	8.23277764505459	7.54415656565196
8.70908263880995	7.96784625265135	8.48159819166868	7.5696916577591
8.14875812474207	8.03496044850989	7.88947589937466	7.78119576295281
8.27306704955205	8.61992294923104	8.29912013988329	8.02220386245661
9.43635994229292	8.42101888081697	7.55130716348009	7.291390495272
8.26536238482702	8.75543791988752	7.28281832755419	7.66753898115724
7.95401762879748	7.64161802033036	8.10878892777914	7.69099795398123
9.00232797899323	7.48241942548111	7.95266972586185	6.90672664503667
8.87394998374589	7.98491976601029	7.88947589937466	8.05872973848172
8.53578124785132	7.86093104873484	7.86778082827534	8.11184107494128
8.49518501163896	8.89745692475995	6.63074163097449	8.60716636317776
8.72461841835598	8.95056826121952	7.52382642705798	8.50498596837128
8.83022860631657	8.52972514025947	7.65627672308163	8.19623326223165
8.7441523053372	7.27596854801368	7.93191116569506	7.05872973848172
8.17777883111032	7.93308083449067	7.28281832755419	7.64369223920288
8.43292495247761	8.52972514025947	7.4380965530321	7.75916945662281
8.04907287936573	8.13011768155023	8.23277764505459	7.75916945662281
7.56902089246712	7.82345634331618	8.12716745709399	7.19623326223166
8.50175766556244	8.2048854499522	8.24965146361899	7.09435364821244
8.69194150598855	7.93308083449067	7.21570413169565	7.80289083405213
8.68906474523299	7.59789664290104	7.49581205088838	6.78119576295281
9.03214626704274	7.21938501964731	8.55130716348009	7.71408156709427
7.78917623002319	7.66299167112293	8.21570413169565	7.75916945662281
8.43120438573713	7.61992294923104	8.03284007454584	7.73680164359436
8.29025356036873	7.66299167112293	9.19842614026381	8.53121750994447
7.63627733087355	8.64161802033036	7.40834920963804	8.59478263872193
7.75931694133008	7.91537883275721	7.73027730452541	9.41628174309981
7.25177941255409	5.57552882987259	10.1984261402638	9.02220386245661
6.76479207139624	7.48241942548111	10.5782742110804	7.64369223920288
7.13825046692368	7.43350982500016	10.5782742110804	7.75916945662281
9.01960597042506	7.24795417184409	9.74819921252267	8.54415656565196
8.48858227681471	7.03496044850989	11.2070910013909	7.66753898115724
8.10626035267924	8.55280875337251	8.10878892777914	7.05872973848172
7.93714381023309	7.70481184681756	7.91084955016723	8.02220386245661
7.70623988904112	9.21938501964732	6.28281832755419	7.80289083405213
7.98014121572856	9.64161802033036	6.52382642705798	7.40922698556585
8.06247262003692	9.89294144363746	6.57827421108036	8.04058239177146
7.8236857604498	8.45817187923443	7.46724289869161	7.94625500922331
8.0104167989704	9.09122866815663	7.49581205088838	8.12911906637312
8.59230318969697	8.445893549456	6.57827421108036	8.4782686299955

8.02189414702033	7.86093104873484	6.63074163097449	7.69099795398123
8.00348630506178	8.51804333521183	5.91084955016723	8.00358818428926
7.69481254184331	7.72527594937727	6.3469486649739	7.8242644848447
8.02189414702033	9.1902386739878	6.40834920963804	7.19623326223166
7.25567332865858	7.38288375193019	4.82338670891689	6.64369223920288
8.17160906387011	7.06738192620227	6.63074163097449	7.38065783336908
8.240034204881	7.0990907859296	5.9933117103592	6.98472915703794
7.69194150598855	6.33041633203606	6.57827421108036	6.49168914575783
7.87521606108989	6.89745692475995	6.28281832755419	6.78119576295281
7.78379303332629	7.93308083449067	6.73027730452541	7.59478263872193
7.20421372930204	7.72527594937727	6.91084955016723	7.92662620247438
7.68618223669987	8.26202935705581	7.07131422236047	7.05872973848172
7.43807437510418	7.52972514025947	6.95266972586185	7.12911906637312
7.14246271821059	7.45817187923443	7.21570413169565	6.98472915703794
7.39282329400928	7.40841884403733	6.28281832755419	7.26036359965137
6.87521606108989	7.1902386739878	5.82338670891689	7.02220386245661
7.96118928419974	7.16049133059375	6.28281832755419	6.59478263872193
7.9895246311872	7.68405328665076	6.63074163097449	7.09435364821244
6.82892240881595	6.52972514025947	6.07131422236047	7.49168914575783
7.33142274934513	7.40841884403733	7.24965146361899	7.64369223920288
7.65113528960067	7.03496044850989	7.28281832755419	7.26036359965137
7.34244977149416	8.26202935705581	6.3469486649739	7.38065783336908
7.48858227681471	7.40841884403733	6.28281832755419	6.78119576295281
6.99186097994216	7.72527594937727	6.40834920963804	6.59478263872193
6.70339152673209	7.52972514025947	6.68136770404446	6.49168914575783
7.60304100139962	7.93308083449067	6.95266972586185	6.64369223920288
7.29782678007353	5.89745692475995	8.37797556059452	6.78119576295281
7.51481381838789	5.96784625265135	7.03284007454584	6.90672664503667
7.47862116676451	5.96784625265135	7.55130716348009	6.54415656565196
7.091085358845	6.48241942548111	7.18093871353497	6.69099795398123
6.78109388372534	6.16049133059375	7.31523980524656	6.49168914575783
7.22024383912099	6.27596854801368	7.18093871353497	6.05872973848172
7.36064288826013	7.00179358457469	7.3469486649739	6.54415656565196
6.69194150598855	6.96784625265135	7.97313382842157	6.69099795398123
6.77024650148834	6.57552882987259	7.24965146361899	6.38065783336908
5.93956647249994	6.0990907859296	7.55130716348009	7.26036359965137
7.66876518362246	6.38288375193019	8.53763222658301	6.64369223920288
6.94439962623589	6.0990907859296	7.75412404647977	6.26036359965137
8.80520633984214	7.38288375193019	7.28281832755419	6.26036359965137
7.19613138300418	7.21938501964731	8.65627672308163	6.12911906637312
7.42775708411866	6.21938501964731	8.61780257526699	6.12911906637312
8.37682281531556	6.70481184681756	9.42329955110402	6.26036359965137
7.67168266700475	8.7454538313149	7.55130716348009	5.64369223920288
6.81843003499831	7.7454538313149	9.32323259600009	6.86608466053932
8.00348630506178	7.70481184681756	9.04255522937905	7.05872973848172
7.51805947789154	7.64161802033036	8.18093871353497	6.73680164359436
6.98249279740796	7.7454538313149	7.91084955016723	7.16306639829646
7.88278928079469	7.50626616743548	8.14531480380425	7.12911906637312
6.29782678007353	5.82345634331618	8.18093871353497	6.43724136173545
6.59076267162119	6.61992294923104	9.77758301930376	7.6194446929562
7.47528545420125	6.38288375193019	9.86778082827534	7.64369223920288
7.36064288826013	6.16049133059375	9.20709100139094	6.43724136173545
6.87521606108989	7.30344928443579	9.03284007454584	7.02220386245661
7.06912610626886	7.13011768155023	8.52382642705798	7.22865473992403
6.25955676314581	9.22658052105152	8.09017324961179	7.8242644848447
8.60914120093604	7.45817187923443	6.63074163097449	7.19623326223166
8.50830051142921	7.59789664290104	6.9933117103592	6.94625500922331
8.18596435684718	7.43350982500016	7.52382642705798	6.94625500922331
8.38039384446776	7.33041633203606	7.10878892777914	7.16306639829646
7.65997716635247	6.86093104873484	7.49581205088838	7.66753898115724

7.77296600284193	6.43350982500016	8.3469486649739	7.12911906637312
7.63328728826774	7.57552882987259	7.68136770404446	7.16306639829646
7.9102225109178	7.50626616743548	7.97313382842157	6.64369223920288
7.35702257258143	7.43350982500016	7.55130716348009	6.86608466053932
7.58148485803318	7.84231537056749	8.18093871353497	7.6194446929562
7.73162520746104	7.80434752036847	8.29912013988329	7.54415656565196
7.90527329494606	7.96784625265135	8.21570413169565	7.35151148770957
7.98484055226438	7.55280875337251	7.84575452194534	7.84532610037253
7.38573386922296	7.13011768155023	7.28281832755419	6.54415656565196
7.92252177586824	7.16049133059375	8.12716745709399	6.43724136173545
7.22819259223648	7.0990907859296	7.73027730452541	6.86608466053932

9181-Osteome	
3.86471431160683	**8908-Osteome_WT**
3.86471431160683	**8910-Osteome**
5.18664240649419	**8911-Osteome_WT**
4.44967681232799	**8916-Osteome**
3.86471431160683	**8917-Osteome**
4.44967681232799	**8919-Osteome**
3.86471431160683	**9184-Osteome**
2.86471431160683	**8909-Osteome_WT**
4.44967681232799	**8914-Osteome**
4.86471431160683	**8915-Osteome**
4.44967681232799	**9181-Osteome_WT**
4.86471431160683	**9185-Osteome**
5.18664240649419	
5.86471431160683	
5.18664240649419	
4.86471431160683	
4.44967681232799	
4.44967681232799	
6.18664240649419	
5.44967681232799	
5.44967681232799	
5.86471431160683	
4.86471431160683	
3.86471431160683	
6.03463931304914	
6.03463931304914	
5.18664240649419	
5.18664240649419	
4.86471431160683	
5.18664240649419	
4.86471431160683	
5.67206923366443	
5.44967681232799	
4.86471431160683	
5.44967681232799	
4.44967681232799	
5.67206923366443	
5.18664240649419	
5.18664240649419	
5.18664240649419	
5.18664240649419	
5.67206923366443	
6.18664240649419	
5.18664240649419	
5.86471431160683	
5.67206923366443	
5.67206923366443	
5.18664240649419	
5.44967681232799	
4.44967681232799	
2.86471431160683	
2.86471431160683	
4.44967681232799	
4.86471431160683	
2.86471431160683	
2.86471431160683	
2.86471431160683	
2.86471431160683	
2.86471431160683	

5.67206923366443
5.18664240649419
5.67206923366443
7.77160490721535
7.90910843096528
5.44967681232799
4.86471431160683
5.86471431160683
7.11264182505042
6.18664240649419
5.44967681232799
6.67206923366443
6.18664240649419
5.18664240649419
5.86471431160683
4.86471431160683
4.86471431160683
4.86471431160683
5.86471431160683
6.18664240649419
4.44967681232799
5.44967681232799
5.44967681232799
4.86471431160683
4.44967681232799
5.18664240649419
5.18664240649419
7.25703173438559
6.77160490721535
6.44967681232799
6.44967681232799
5.67206923366443
6.32414593024413
6.03463931304914
5.86471431160683
6.67206923366443
6.67206923366443
6.18664240649419
6.18664240649419
5.44967681232799
5.18664240649419
3.86471431160683
5.86471431160683
4.86471431160683
4.86471431160683
4.86471431160683
5.44967681232799
2.86471431160683
4.44967681232799
4.86471431160683
5.18664240649419
4.44967681232799
4.86471431160683
5.18664240649419
4.86471431160683
3.86471431160683
3.86471431160683
3.86471431160683
3.86471431160683
4.44967681232799

3.86471431160683
7.03463931304914
6.56515402974792
6.86471431160683
5.18664240649419
4.86471431160683
4.44967681232799
5.86471431160683
5.18664240649419
4.86471431160683
5.67206923366443
5.18664240649419
5.18664240649419
6.03463931304914
6.18664240649419
4.86471431160683
5.18664240649419
6.56515402974792
5.44967681232799
4.86471431160683
4.44967681232799
5.18664240649419
4.86471431160683
6.18664240649419
5.44967681232799
5.86471431160683
5.18664240649419
6.95217715285717
5.44967681232799
6.44967681232799
6.44967681232799
4.86471431160683
5.67206923366443
5.44967681232799
5.18664240649419
6.18664240649419
5.44967681232799
5.18664240649419
6.32414593024413
6.03463931304914
5.86471431160683
5.67206923366443
5.67206923366443
5.86471431160683
6.32414593024413
6.32414593024413
6.95217715285717
5.67206923366443
5.44967681232799
5.18664240649419
5.18664240649419
4.44967681232799
4.44967681232799
4.44967681232799
3.86471431160683
2.86471431160683
2.86471431160683
5.18664240649419
4.44967681232799
5.18664240649419

2.86471431160683
4.44967681232799
3.86471431160683
5.67206923366443
4.44967681232799
5.67206923366443
5.67206923366443
6.03463931304914
6.03463931304914
6.56515402974792
2.86471431160683
3.86471431160683
4.86471431160683
3.86471431160683
5.67206923366443
3.86471431160683
5.67206923366443
6.67206923366443
5.67206923366443
6.03463931304914
5.44967681232799
5.86471431160683
5.44967681232799
4.86471431160683
6.03463931304914
6.77160490721535
5.44967681232799
5.67206923366443
5.86471431160683
6.03463931304914
6.03463931304914
6.32414593024413
4.44967681232799
4.86471431160683
5.44967681232799
5.67206923366443
5.67206923366443
5.18664240649419
4.44967681232799
5.44967681232799
4.86471431160683
5.44967681232799
4.86471431160683
5.86471431160683
5.18664240649419
4.86471431160683
5.44967681232799
5.44967681232799
6.56515402974792
4.86471431160683
6.03463931304914
6.18664240649419
5.67206923366443
5.67206923366443
6.95217715285717
6.32414593024413
5.18664240649419
6.03463931304914
5.18664240649419
5.67206923366443

5.67206923366443
6.03463931304914
6.56515402974792
6.56515402974792
6.77160490721535
6.32414593024413
6.56515402974792
6.56515402974792
5.18664240649419
5.86471431160683
6.18664240649419
5.44967681232799
4.86471431160683
4.44967681232799
5.44967681232799
6.03463931304914
5.44967681232799
5.67206923366443
6.67206923366443
5.18664240649419
5.67206923366443
5.18664240649419
5.18664240649419
4.86471431160683
6.18664240649419
5.67206923366443
5.67206923366443
4.44967681232799
4.44967681232799
5.67206923366443
4.86471431160683
2.86471431160683
3.86471431160683
2.86471431160683
3.86471431160683
6.03463931304914
4.44967681232799
5.18664240649419
6.86471431160683
6.03463931304914
2.86471431160683
3.86471431160683
4.44967681232799
3.86471431160683
3.86471431160683
4.44967681232799
5.44967681232799
5.67206923366443
4.86471431160683
5.44967681232799
4.44967681232799
5.44967681232799
5.44967681232799
5.18664240649419
3.86471431160683
3.86471431160683
4.44967681232799
5.44967681232799
4.44967681232799
5.18664240649419

5.18664240649419
5.67206923366443
4.86471431160683
4.86471431160683
4.86471431160683
3.86471431160683
4.86471431160683
4.44967681232799
4.86471431160683
3.86471431160683
5.18664240649419
4.44967681232799
3.86471431160683
2.86471431160683
3.86471431160683
3.86471431160683
3.86471431160683
2.86471431160683
2.86471431160683
4.86471431160683
5.18664240649419
5.44967681232799
4.44967681232799
2.86471431160683
5.18664240649419
4.86471431160683
5.18664240649419
4.44967681232799
4.86471431160683
5.18664240649419
4.44967681232799
4.44967681232799
5.67206923366443
5.67206923366443
6.56515402974792
5.67206923366443
5.67206923366443
6.77160490721535
6.44967681232799
7.03463931304914
6.03463931304914
5.18664240649419
5.44967681232799
5.44967681232799
5.86471431160683
5.18664240649419
5.44967681232799
3.86471431160683
5.44967681232799
4.44967681232799
4.44967681232799
6.18664240649419
5.86471431160683
5.44967681232799
5.86471431160683
5.44967681232799
5.44967681232799
5.18664240649419
5.18664240649419
11.8870821246353

9.44967681232799
8.90910843096528
11.3485300888711
10.0346393130491
9.973238768385
6.44967681232799
8.18664240649419
7.38827626766384
6.32414593024413
9.68489327402202
8.69760432577157
8.22226631622491
6.95217715285717
9.20456431449145
8.64607402513149
7.18664240649419
7.90910843096528
10.6328986363838
8.32414593024413
8.7226953067344
8.86471431160683
9.6196018137703
8.56515402974792
8.53713965357833
9.44967681232799
8.18664240649419
7.67206923366443
7.95217715285717
8.29097906630893
8.07416767723578
8.32414593024413
8.9939973285518
8.56515402974792
8.64607402513149
8.47942415572204
7.50857050138155
8.7226953067344
7.44967681232799
7.11264182505042
8.41930316328447
8.03463931304914
7.77160490721535
7.50857050138155
8.22226631622491
8.69760432577157
7.25703173438559
8.18664240649419
8.15011653046908
9.3565674079365
7.86471431160683
7.90910843096528
9.01446143111151
7.90910843096528
8.973238768385
7.67206923366443
10.1684950597839
10.2134424658379
10.0545388704868
7.67206923366443
9.89813731314428

9.63289863638376
8.69760432577157
9.7226953067344
7.25703173438559
9.03463931304914
8.59263476617003
8.22226631622491
8.38827626766384
6.18664240649419
7.7226953067344
7.38827626766384
8.22226631622491
6.86471431160683
7.7226953067344
7.18664240649419
7.03463931304914
7.50857050138155
7.67206923366443
7.81891062199371
6.86471431160683
6.77160490721535
7.38827626766384
6.32414593024413
6.44967681232799
6.56515402974792
5.44967681232799
5.18664240649419
5.86471431160683
6.77160490721535
6.95217715285717
4.86471431160683
5.18664240649419
5.18664240649419
4.44967681232799
3.86471431160683
2.86471431160683
5.86471431160683
5.18664240649419
6.18664240649419
6.44967681232799
6.67206923366443
7.03463931304914
6.95217715285717
6.77160490721535
6.44967681232799
7.32414593024413
6.56515402974792
6.56515402974792
7.11264182505042
7.44967681232799
6.77160490721535
7.11264182505042
7.18664240649419
6.56515402974792
6.18664240649419
6.03463931304914
6.56515402974792
6.44967681232799
6.03463931304914
6.32414593024413

5.44967681232799
5.67206923366443
6.03463931304914
6.03463931304914
6.44967681232799
6.86471431160683
7.18664240649419
7.18664240649419
7.77160490721535
7.18664240649419
6.77160490721535
6.86471431160683
6.67206923366443
7.18664240649419
5.86471431160683
5.86471431160683
7.32414593024413
6.86471431160683
7.03463931304914
7.03463931304914
5.86471431160683
5.44967681232799
5.44967681232799
5.44967681232799
5.44967681232799
5.67206923366443
5.18664240649419
4.86471431160683
4.86471431160683
4.44967681232799
4.86471431160683
6.44967681232799
5.18664240649419
4.44967681232799
4.86471431160683
4.44967681232799
5.18664240649419
6.03463931304914
6.32414593024413
5.18664240649419
5.67206923366443
5.44967681232799
7.03463931304914
7.90910843096528
6.86471431160683
6.67206923366443
6.86471431160683
7.32414593024413
6.86471431160683
7.18664240649419
6.03463931304914
6.18664240649419
5.67206923366443
6.03463931304914
5.67206923366443
5.86471431160683
6.03463931304914
6.77160490721535
6.03463931304914
6.32414593024413

5.86471431160683
6.44967681232799
6.32414593024413
6.67206923366443
6.32414593024413
6.56515402974792
6.56515402974792
6.03463931304914
4.86471431160683
5.44967681232799
6.18664240649419
6.03463931304914
5.86471431160683
5.67206923366443
5.44967681232799
5.86471431160683
6.18664240649419
5.86471431160683
5.18664240649419
6.32414593024413
6.67206923366443
6.67206923366443
6.56515402974792
6.03463931304914
6.32414593024413
5.44967681232799
6.18664240649419
6.67206923366443
6.67206923366443
7.67206923366443
7.11264182505042
7.03463931304914
8.22226631622491
7.03463931304914
6.86471431160683
7.18664240649419
6.44967681232799
6.18664240649419
6.77160490721535
6.18664240649419
5.67206923366443
6.03463931304914
6.44967681232799
5.18664240649419
6.03463931304914
5.86471431160683
6.77160490721535
6.03463931304914
6.95217715285717
6.67206923366443
7.18664240649419
6.95217715285717
6.18664240649419
7.03463931304914
6.44967681232799
6.18664240649419
6.32414593024413
7.11264182505042
6.86471431160683
7.11264182505042

6.32414593024413
6.03463931304914
7.38827626766384
6.18664240649419
5.44967681232799
6.18664240649419
6.18664240649419
4.44967681232799
6.32414593024413
5.86471431160683
5.44967681232799
7.77160490721535
7.03463931304914
7.56515402974792
7.18664240649419
7.9939973285518
7.86471431160683
8.22226631622491
7.95217715285717
7.32414593024413
8.03463931304914
8.03463931304914
8.07416767723578
8.18664240649419
6.32414593024413
6.86471431160683
7.11264182505042
7.86471431160683
7.6196018137703
6.86471431160683
6.95217715285717
7.11264182505042
6.56515402974792
6.44967681232799
6.03463931304914
6.44967681232799
6.44967681232799
6.86471431160683
6.77160490721535
7.18664240649419
8.11264182505042
7.9939973285518
7.7226953067344
7.44967681232799
5.18664240649419
7.90910843096528
7.6196018137703
7.81891062199371
7.56515402974792
7.81891062199371
7.7226953067344
8.86471431160683
8.32414593024413
8.64607402513149
6.95217715285717
6.95217715285717
7.50857050138155
7.18664240649419
7.25703173438559
7.38827626766384

7.25703173438559
6.95217715285717
6.03463931304914
6.32414593024413
7.11264182505042
5.67206923366443
6.67206923366443
6.44967681232799
6.18664240649419
7.32414593024413
7.7226953067344
7.25703173438559
7.11264182505042
7.03463931304914
7.50857050138155
7.25703173438559
7.6196018137703
6.95217715285717
6.56515402974792
5.86471431160683
6.18664240649419
6.95217715285717
6.44967681232799
6.67206923366443
6.32414593024413
5.67206923366443
6.86471431160683
7.38827626766384
6.77160490721535
6.77160490721535
6.03463931304914
6.44967681232799
6.32414593024413
6.56515402974792
6.67206923366443
5.67206923366443
5.44967681232799
5.86471431160683
5.86471431160683
5.86471431160683
4.86471431160683
6.56515402974792
6.95217715285717
6.18664240649419
6.18664240649419
7.18664240649419
7.11264182505042
7.44967681232799
8.38827626766384
7.44967681232799
7.6196018137703
7.11264182505042
7.32414593024413
6.77160490721535
7.7226953067344
7.77160490721535
7.18664240649419
6.95217715285717
7.32414593024413
7.56515402974792

7.56515402974792
7.50857050138155
7.56515402974792
7.38827626766384
7.56515402974792
7.50857050138155
6.86471431160683
7.50857050138155
7.95217715285717
7.81891062199371
7.25703173438559

Gene	ProbeID	Codeset.Name	Probe.Label
**RAD51**	NM_133487.2:566	NS_CANCERPATH_C2535	RAD51
**GRIN2B**	NM_000834.3:1144	NS_CANCERPATH_C2535	GRIN2B
**IL19**	NM_013371.3:1030	NS_CANCERPATH_C2535	IL19
**IFNG**	NM_000619.2:970	NS_CANCERPATH_C2535	IFNG
**RASAL1**	NM_004658.1:2900	NS_CANCERPATH_C2535	RASAL1
**RASGRF1**	NM_153815.2:2443	NS_CANCERPATH_C2535	RASGRF1
**CLCF1**	NM_013246.2:1296	NS_CANCERPATH_C2535	CLCF1
**PRKCG**	NM_002739.3:445	NS_CANCERPATH_C2535	PRKCG
**TSHR**	NM_001018036.2:735	NS_CANCERPATH_C2535	TSHR
**SMC1B**	NM_148674.3:3600	NS_CANCERPATH_C2535	SMC1B
**WNT16**	NM_057168.1:1621	NS_CANCERPATH_C2535	WNT16
**ALK**	NM_004304.3:3495	NS_CANCERPATH_C2535	ALK
**IL13RA2**	NM_000640.2:400	NS_CANCERPATH_C2535	IL13RA2
**SSX1**	NM_005635.2:148	NS_CANCERPATH_C2535	SSX1
**FGF6**	NM_020996.1:420	NS_CANCERPATH_C2535	FGF6
**GNGT1**	NM_021955.3:260	NS_CANCERPATH_C2535	GNGT1
**GATA3**	NM_001002295.1:2835	NS_CANCERPATH_C2535	GATA3
**PRL**	NM_000948.3:835	NS_CANCERPATH_C2535	PRL
**WNT2**	NM_003391.2:2014	NS_CANCERPATH_C2535	WNT2
**LAMA1**	NM_005559.2:5230	NS_CANCERPATH_C2535	LAMA1
**MPO**	NM_000250.1:545	NS_CANCERPATH_C2535	MPO
**PPP2R2B**	NM_181676.2:690	NS_CANCERPATH_C2535	PPP2R2B
**FGF16**	NM_003868.1:504	NS_CANCERPATH_C2535	FGF16
**CALML5**	NM_017422.4:136	NS_CANCERPATH_C2535	CALML5
**PRKACG**	NM_002732.2:775	NS_CANCERPATH_C2535	PRKACG
**IL23R**	NM_144701.2:710	NS_CANCERPATH_C2535	IL23R
**CACNG1**	NM_000727.2:464	NS_CANCERPATH_C2535	CACNG1
**HNF1A**	NM_000545.4:2125	NS_CANCERPATH_C2535	HNF1A
**NODAL**	NM_018055.3:320	NS_CANCERPATH_C2535	NODAL
**LEFTY1**	NM_020997.2:1405	NS_CANCERPATH_C2535	LEFTY1
**GATA1**	NM_002049.2:1001	NS_CANCERPATH_C2535	GATA1
**IL22RA2**	NM_181309.1:290	NS_CANCERPATH_C2535	IL22RA2
**IL1A**	NM_000575.3:1085	NS_CANCERPATH_C2535	IL1A
**PTCRA**	NM_138296.2:507	NS_CANCERPATH_C2535	PTCRA
**HOXA11**	NM_005523.5:1525	NS_CANCERPATH_C2535	HOXA11
**WT1**	NM_000378.3:2160	NS_CANCERPATH_C2535	WT1
**IFNA2**	NM_000605.3:11	NS_CANCERPATH_C2535	IFNA2
**CACNA2D4**	NM_001005737.1:990	NS_CANCERPATH_C2535	CACNA2D4
**UTY**	NM_007125.3:450	NS_CANCERPATH_C2535	UTY
**LEP**	NM_000230.2:1875	NS_CANCERPATH_C2535	LEP
**FGF19**	NM_005117.2:786	NS_CANCERPATH_C2535	FGF19
**TLX1**	NM_005521.3:1915	NS_CANCERPATH_C2535	TLX1
**PLA2G3**	NM_015715.3:2415	NS_CANCERPATH_C2535	PLA2G3
**HPGD**	NM_001145816.2:96	NS_CANCERPATH_C2535	HPGD
**PPP2R2C**	NM_181876.2:2215	NS_CANCERPATH_C2535	PPP2R2C
**IL13**	NM_002188.2:516	NS_CANCERPATH_C2535	IL13
**IFNA7**	NM_021057.2:0	NS_CANCERPATH_C2535	IFNA7
**EPO**	NM_000799.2:1055	NS_CANCERPATH_C2535	EPO
**PAX5**	NM_016734.1:2288	NS_CANCERPATH_C2535	PAX5
**PPP3R2**	NM_147180.2:1165	NS_CANCERPATH_C2535	PPP3R2
**LAMB4**	NM_007356.2:2852	NS_CANCERPATH_C2535	LAMB4
**CSF2**	NM_000758.2:475	NS_CANCERPATH_C2535	CSF2
**FGF5**	NM_004464.3:4785	NS_CANCERPATH_C2535	FGF5
**CSF3**	NM_000759.3:851	NS_CANCERPATH_C2535	CSF3
**CREB3L3**	NM_001271995.1:1050	NS_CANCERPATH_C2535	CREB3L3
**GRIN1**	NM_000832.5:1290	NS_CANCERPATH_C2535	GRIN1
**IL12B**	NM_002187.2:1435	NS_CANCERPATH_C2535	IL12B
**TCL1B**	NM_004918.2:319	NS_CANCERPATH_C2535	TCL1B
**FGF8**	NM_033163.3:544	NS_CANCERPATH_C2535	FGF8

**CDH1**	NM_004360.2:1230	NS_CANCERPATH_C2535	CDH1
**COL4A5**	NM_033381.1:5360	NS_CANCERPATH_C2535	COL4A5
**LAMC2**	NM_005562.2:2819	NS_CANCERPATH_C2535	LAMC2
**BAIAP3**	NM_003933.4:1950	NS_CANCERPATH_C2535	BAIAP3
**PROM1**	NM_006017.1:925	NS_CANCERPATH_C2535	PROM1
**WNT4**	NM_030761.3:625	NS_CANCERPATH_C2535	WNT4
**MYB**	NM_005375.2:3145	NS_CANCERPATH_C2535	MYB
**PLA1A**	NM_015900.2:1250	NS_CANCERPATH_C2535	PLA1A
**TMPRSS2**	NM_005656.2:1290	NS_CANCERPATH_C2535	TMPRSS2
**IL5RA**	NM_000564.3:210	NS_CANCERPATH_C2535	IL5RA
**FGF14**	NM_004115.3:725	NS_CANCERPATH_C2535	FGF14
**CACNG6**	NM_145814.1:1745	NS_CANCERPATH_C2535	CACNG6
**SFN**	NM_006142.3:579	NS_CANCERPATH_C2535	SFN
**CACNA2D3**	NM_018398.2:205	NS_CANCERPATH_C2535	CACNA2D3
**GRIA3**	NM_000828.4:426	NS_CANCERPATH_C2535	GRIA3
**CSF3R**	NM_156038.2:90	NS_CANCERPATH_C2535	CSF3R
**CDK2**	NM_001798.2:220	NS_CANCERPATH_C2535	CDK2
**NPM1**	NM_002520.5:10	NS_CANCERPATH_C2535	NPM1
**FANCF**	NM_022725.2:845	NS_CANCERPATH_C2535	FANCF
**ACVR2A**	NM_001616.3:1245	NS_CANCERPATH_C2535	ACVR2A
**AMER1**	NM_152424.3:5755	NS_CANCERPATH_C2535	AMER1
**ENDOG**	NM_004435.2:694	NS_CANCERPATH_C2535	ENDOG
**PCNA**	NM_002592.2:280	NS_CANCERPATH_C2535	PCNA
**PRKX**	NM_005044.1:2590	NS_CANCERPATH_C2535	PRKX
**CDKN1B**	NM_004064.2:365	NS_CANCERPATH_C2535	CDKN1B
**MAP3K5**	NM_005923.3:2415	NS_CANCERPATH_C2535	MAP3K5
**PHF6**	NM_032335.3:85	NS_CANCERPATH_C2535	PHF6
**LIG4**	NM_002312.3:2623	NS_CANCERPATH_C2535	LIG4
**RFC3**	NM_002915.3:740	NS_CANCERPATH_C2535	RFC3
**EFNA1**	NM_004428.2:650	NS_CANCERPATH_C2535	EFNA1
**CACNA1C**	NM_199460.2:4785	NS_CANCERPATH_C2535	CACNA1C
**NFATC1**	NM_172389.1:1984	NS_CANCERPATH_C2535	NFATC1
**CACNB3**	NM_000725.2:1115	NS_CANCERPATH_C2535	CACNB3
**FANCG**	NM_004629.1:1900	NS_CANCERPATH_C2535	FANCG
**PTTG2**	NM_006607.2:5	NS_CANCERPATH_C2535	PTTG2
**ERCC6**	NM_000124.2:3235	NS_CANCERPATH_C2535	ERCC6
**PIK3CB**	NM_006219.1:2945	NS_CANCERPATH_C2535	PIK3CB
**HDAC11**	NM_024827.3:2601	NS_CANCERPATH_C2535	HDAC11
**IL1RAP**	NM_002182.2:460	NS_CANCERPATH_C2535	IL1RAP
**IL2RB**	NM_000878.2:1980	NS_CANCERPATH_C2535	IL2RB
**PRKCB**	NM_212535.1:1750	NS_CANCERPATH_C2535	PRKCB
**IL15**	NM_172174.1:1685	NS_CANCERPATH_C2535	IL15
**MFNG**	NM_002405.2:1681	NS_CANCERPATH_C2535	MFNG
**CD40**	NM_001250.4:1265	NS_CANCERPATH_C2535	CD40
**SOCS1**	NM_003745.1:1025	NS_CANCERPATH_C2535	SOCS1
**FGF1**	NM_033137.1:315	NS_CANCERPATH_C2535	FGF1
**FBXW7**	NM_018315.4:1480	NS_CANCERPATH_C2535	FBXW7
**SYK**	NM_003177.3:1685	NS_CANCERPATH_C2535	SYK
**IRS1**	NM_005544.2:6224	NS_CANCERPATH_C2535	IRS1
**E2F5**	NM_001951.3:444	NS_CANCERPATH_C2535	E2F5
**RFC4**	NM_181573.2:1035	NS_CANCERPATH_C2535	RFC4
**PRKAR1B**	NM_001164759.1:1112	NS_CANCERPATH_C2535	PRKAR1B
**XRCC4**	NM_003401.3:772	NS_CANCERPATH_C2535	XRCC4
**HRAS**	NM_005343.2:396	NS_CANCERPATH_C2535	HRAS
**MCM5**	NM_006739.3:1580	NS_CANCERPATH_C2535	MCM5
**MAPK12**	NM_002969.3:425	NS_CANCERPATH_C2535	MAPK12
**ERCC2**	NM_000400.2:240	NS_CANCERPATH_C2535	ERCC2
**ALKBH2**	NM_001001655.2:907	NS_CANCERPATH_C2535	ALKBH2
**GHR**	NM_000163.2:1835	NS_CANCERPATH_C2535	GHR
**LAMA3**	NM_000227.3:4260	NS_CANCERPATH_C2535	LAMA3

**PRKAR2B**	NM_002736.2:1350	NS_CANCERPATH_C2535	PRKAR2B
**BMPR1B**	NM_001203.1:430	NS_CANCERPATH_C2535	BMPR1B
**MLF1**	NM_022443.3:720	NS_CANCERPATH_C2535	MLF1
**ETV1**	NM_004956.4:1719	NS_CANCERPATH_C2535	ETV1
**HELLS**	NM_018063.3:2040	NS_CANCERPATH_C2535	HELLS
**PRKACB**	NM_182948.2:805	NS_CANCERPATH_C2535	PRKACB
**CASP9**	NM_001229.2:1805	NS_CANCERPATH_C2535	CASP9
**MLLT3**	NM_004529.2:1480	NS_CANCERPATH_C2535	MLLT3
**MAPK9**	NM_139068.2:365	NS_CANCERPATH_C2535	MAPK9
**MCM4**	NM_182746.1:1200	NS_CANCERPATH_C2535	MCM4
**CACNA1D**	NM_000720.2:5195	NS_CANCERPATH_C2535	CACNA1D
**ZAK**	NM_016653.2:995	NS_CANCERPATH_C2535	ZAK
**IDH1**	NM_005896.2:105	NS_CANCERPATH_C2535	IDH1
**POLB**	NM_002690.1:145	NS_CANCERPATH_C2535	POLB
**GNG4**	NM_004485.2:215	NS_CANCERPATH_C2535	GNG4
**LIF**	NM_002309.3:1240	NS_CANCERPATH_C2535	LIF
**FOSL1**	NM_005438.2:280	NS_CANCERPATH_C2535	FOSL1
**HMGA1**	NM_145904.1:871	NS_CANCERPATH_C2535	HMGA1
**FANCE**	NM_021922.2:1275	NS_CANCERPATH_C2535	FANCE
**PDGFB**	NM_033016.2:1480	NS_CANCERPATH_C2535	PDGFB
**FLNC**	NM_001127487.1:7144	NS_CANCERPATH_C2535	FLNC
**TNFRSF10D**	NM_003840.3:2380	NS_CANCERPATH_C2535	TNFRSF10D
**SOX9**	NM_000346.2:2135	NS_CANCERPATH_C2535	SOX9
**PPARG**	NM_015869.3:1035	NS_CANCERPATH_C2535	PPARG
**CREB5**	NM_182898.2:1885	NS_CANCERPATH_C2535	CREB5
**POLR2J**	NM_006234.4:618	NS_CANCERPATH_C2535	POLR2J
**HSPA2**	NM_021979.3:2095	NS_CANCERPATH_C2535	HSPA2
**INHBB**	NM_002193.2:1969	NS_CANCERPATH_C2535	INHBB
**HIST1H3B**	NM_003537.3:335	NS_CANCERPATH_C2535	HIST1H3B
**RAC2**	NM_002872.3:1069	NS_CANCERPATH_C2535	RAC2
**APH1B**	NM_001145646.1:2930	NS_CANCERPATH_C2535	APH1B
**NRAS**	NM_002524.3:877	NS_CANCERPATH_C2535	NRAS
**BID**	NM_197966.1:2095	NS_CANCERPATH_C2535	BID
**PLA2G4C**	NM_003706.2:2310	NS_CANCERPATH_C2535	PLA2G4C
**CREBBP**	NM_004380.2:8855	NS_CANCERPATH_C2535	CREBBP
**SUV39H2**	NM_024670.3:2035	NS_CANCERPATH_C2535	SUV39H2
**BRCA1**	NM_007305.2:1275	NS_CANCERPATH_C2535	BRCA1
**MUTYH**	NM_012222.2:412	NS_CANCERPATH_C2535	MUTYH
**FGF11**	NM_004112.2:1670	NS_CANCERPATH_C2535	FGF11
**CDC7**	NM_003503.2:805	NS_CANCERPATH_C2535	CDC7
**WNT2B**	NM_024494.1:1530	NS_CANCERPATH_C2535	WNT2B
**EZH2**	NM_004456.3:190	NS_CANCERPATH_C2535	EZH2
**CDKN2D**	NM_001800.3:870	NS_CANCERPATH_C2535	CDKN2D
**H2AFX**	NM_002105.2:1392	NS_CANCERPATH_C2535	H2AFX
**HHEX**	NM_002729.4:1479	NS_CANCERPATH_C2535	HHEX
**PIK3CG**	NM_002649.2:2125	NS_CANCERPATH_C2535	PIK3CG
**PIK3R5**	NM_001142633.1:3335	NS_CANCERPATH_C2535	PIK3R5
**GZMB**	NM_004131.3:540	NS_CANCERPATH_C2535	GZMB
**TNF**	NM_000594.2:1010	NS_CANCERPATH_C2535	TNF
**FASLG**	NM_000639.1:625	NS_CANCERPATH_C2535	FASLG
**CD19**	NM_001770.4:1770	NS_CANCERPATH_C2535	CD19
**CCR7**	NM_001838.2:1610	NS_CANCERPATH_C2535	CCR7
**CASP12**	NM_001191016.1:595	NS_CANCERPATH_C2535	CASP12
**CXXC4**	NM_025212.1:293	NS_CANCERPATH_C2535	CXXC4
**FLT3**	NM_004119.1:580	NS_CANCERPATH_C2535	FLT3
**PAK3**	NM_002578.2:1830	NS_CANCERPATH_C2535	PAK3
**RNF43**	NM_017763.4:725	NS_CANCERPATH_C2535	RNF43
**RASGRP1**	NM_005739.3:365	NS_CANCERPATH_C2535	RASGRP1
**CACNA2D2**	NM_001005505.1:2045	NS_CANCERPATH_C2535	CACNA2D2
**NPM2**	NM_182795.1:745	NS_CANCERPATH_C2535	NPM2

**LAMB3**	NM_000228.2:695	NS_CANCERPATH_C2535	LAMB3
**COL4A6**	NM_001847.2:1070	NS_CANCERPATH_C2535	COL4A6
**HHIP**	NM_022475.1:2135	NS_CANCERPATH_C2535	HHIP
**IL20RA**	NM_014432.2:535	NS_CANCERPATH_C2535	IL20RA
**PLA2G10**	NM_003561.1:779	NS_CANCERPATH_C2535	PLA2G10
**PRKAA2**	NM_006252.2:975	NS_CANCERPATH_C2535	PRKAA2
**PRLR**	NM_001204318.1:563	NS_CANCERPATH_C2535	PRLR
**WNT7B**	NM_058238.1:1535	NS_CANCERPATH_C2535	WNT7B
**COL2A1**	NM_001844.4:4745	NS_CANCERPATH_C2535	COL2A1
**RELN**	NM_005045.2:345	NS_CANCERPATH_C2535	RELN
**WNT11**	NM_004626.2:960	NS_CANCERPATH_C2535	WNT11
**CSF1R**	NM_005211.2:3775	NS_CANCERPATH_C2535	CSF1R
**NGF**	NM_002506.2:100	NS_CANCERPATH_C2535	NGF
**PLA2G4A**	NM_024420.2:1763	NS_CANCERPATH_C2535	PLA2G4A
**CALML3**	NM_005185.2:685	NS_CANCERPATH_C2535	CALML3
**PLA2G4F**	NM_213600.2:3140	NS_CANCERPATH_C2535	PLA2G4F
**ZIC2**	NM_007129.2:1849	NS_CANCERPATH_C2535	ZIC2
**LEFTY2**	NM_003240.2:1283	NS_CANCERPATH_C2535	LEFTY2
**PLA2G2A**	NM_000300.2:715	NS_CANCERPATH_C2535	PLA2G2A
**TNN**	NM_022093.1:3580	NS_CANCERPATH_C2535	TNN
**FGF13**	NM_033642.1:620	NS_CANCERPATH_C2535	FGF13
**BDNF**	NM_170732.4:730	NS_CANCERPATH_C2535	BDNF
**DKK2**	NM_014421.2:1135	NS_CANCERPATH_C2535	DKK2
**PITX2**	NM_000325.5:1381	NS_CANCERPATH_C2535	PITX2
**CAMK2B**	NM_001220.3:365	NS_CANCERPATH_C2535	CAMK2B
**WNT10B**	NM_003394.2:2070	NS_CANCERPATH_C2535	WNT10B
**HES5**	NM_001010926.3:1160	NS_CANCERPATH_C2535	HES5
**BRIP1**	NM_032043.1:1130	NS_CANCERPATH_C2535	BRIP1
**SIRT4**	NM_012240.1:915	NS_CANCERPATH_C2535	SIRT4
**DTX1**	NM_004416.2:2855	NS_CANCERPATH_C2535	DTX1
**CHEK1**	NM_001114121.1:2225	NS_CANCERPATH_C2535	CHEK1
**TTK**	NM_003318.3:1200	NS_CANCERPATH_C2535	TTK
**CCNE1**	NM_001238.1:1635	NS_CANCERPATH_C2535	CCNE1
**UBE2T**	NM_014176.3:595	NS_CANCERPATH_C2535	UBE2T
**FZD2**	NM_001466.2:845	NS_CANCERPATH_C2535	FZD2
**CCNA1**	NM_003914.3:1605	NS_CANCERPATH_C2535	CCNA1
**CACNB4**	NM_001005747.2:1483	NS_CANCERPATH_C2535	CACNB4
**FGF12**	NM_004113.4:685	NS_CANCERPATH_C2535	FGF12
**FANCB**	NM_152633.2:2470	NS_CANCERPATH_C2535	FANCB
**CDKN2A**	NM_000077.3:975	NS_CANCERPATH_C2535	CDKN2A
**COL4A4**	NM_000092.4:8255	NS_CANCERPATH_C2535	COL4A4
**CASP10**	NM_032977.3:20	NS_CANCERPATH_C2535	CASP10
**MAPK8IP2**	NM_012324.2:1885	NS_CANCERPATH_C2535	MAPK8IP2
**IL1R2**	NM_173343.1:113	NS_CANCERPATH_C2535	IL1R2
**IL2RA**	NM_000417.1:1000	NS_CANCERPATH_C2535	IL2RA
**SGK2**	NM_170693.1:1485	NS_CANCERPATH_C2535	SGK2
**ETV7**	NM_016135.2:794	NS_CANCERPATH_C2535	ETV7
**WNT3**	NM_030753.3:1335	NS_CANCERPATH_C2535	WNT3
**CACNA1E**	NM_000721.2:9325	NS_CANCERPATH_C2535	CACNA1E
**FOXL2**	NM_023067.2:1290	NS_CANCERPATH_C2535	FOXL2
**LAMC3**	NM_006059.3:2090	NS_CANCERPATH_C2535	LAMC3
**HIST1H3G**	NM_003534.2:348	NS_CANCERPATH_C2535	HIST1H3G
**GLI1**	NM_005269.1:2885	NS_CANCERPATH_C2535	GLI1
**CDKN2B**	NM_004936.3:1175	NS_CANCERPATH_C2535	CDKN2B
**CCNA2**	NM_001237.2:1210	NS_CANCERPATH_C2535	CCNA2
**SPOP**	NM_001007226.1:2726	NS_CANCERPATH_C2535	SPOP
**BIRC3**	NM_182962.1:3	NS_CANCERPATH_C2535	BIRC3
**PPARGC1A**	NM_013261.3:1505	NS_CANCERPATH_C2535	PPARGC1A
**FGF10**	NM_004465.1:85	NS_CANCERPATH_C2535	FGF10
**GRIN2A**	NM_000833.3:4497	NS_CANCERPATH_C2535	GRIN2A

**ITGA8**	NM_003638.1:1215	NS_CANCERPATH_C2535	ITGA8
**CHEK2**	NM_007194.3:140	NS_CANCERPATH_C2535	CHEK2
**C19orf40**	NM_152266.3:376	NS_CANCERPATH_C2535	C19orf40
**CDC6**	NM_001254.3:1300	NS_CANCERPATH_C2535	CDC6
**EFNA3**	NM_004952.4:1672	NS_CANCERPATH_C2535	EFNA3
**CREB3L4**	NM_130898.2:1505	NS_CANCERPATH_C2535	CREB3L4
**NASP**	NM_172164.1:2970	NS_CANCERPATH_C2535	NASP
**GNG7**	NM_052847.1:3920	NS_CANCERPATH_C2535	GNG7
**MAPK8IP1**	NM_005456.2:259	NS_CANCERPATH_C2535	MAPK8IP1
**SHC4**	NM_203349.2:1570	NS_CANCERPATH_C2535	SHC4
**ARNT2**	NM_014862.3:5260	NS_CANCERPATH_C2535	ARNT2
**RPS6KA6**	NM_014496.1:2250	NS_CANCERPATH_C2535	RPS6KA6
**DUSP2**	NM_004418.3:1235	NS_CANCERPATH_C2535	DUSP2
**IRAK2**	NM_001570.3:1285	NS_CANCERPATH_C2535	IRAK2
**DUSP10**	NM_144728.2:1176	NS_CANCERPATH_C2535	DUSP10
**MAPK10**	NM_002753.2:2080	NS_CANCERPATH_C2535	MAPK10
**DUSP4**	NM_057158.2:3115	NS_CANCERPATH_C2535	DUSP4
**FGF9**	NM_002010.2:1565	NS_CANCERPATH_C2535	FGF9
**DUSP8**	NM_004420.2:2165	NS_CANCERPATH_C2535	DUSP8
**SOX17**	NM_022454.3:1374	NS_CANCERPATH_C2535	SOX17
**IL6R**	NM_000565.2:993	NS_CANCERPATH_C2535	IL6R
**IL3RA**	NM_002183.2:745	NS_CANCERPATH_C2535	IL3RA
**MCM2**	NM_004526.2:2945	NS_CANCERPATH_C2535	MCM2
**CEBPA**	NM_004364.2:1320	NS_CANCERPATH_C2535	CEBPA
**CACNB2**	NM_000724.3:2610	NS_CANCERPATH_C2535	CACNB2
**ACVR1B**	NM_004302.3:1700	NS_CANCERPATH_C2535	ACVR1B
**NTRK1**	NM_001012331.1:1365	NS_CANCERPATH_C2535	NTRK1
**BRCA2**	NM_000059.3:115	NS_CANCERPATH_C2535	BRCA2
**TNFRSF10A**	NM_003844.2:950	NS_CANCERPATH_C2535	TNFRSF10A
**FGF17**	NM_003867.2:279	NS_CANCERPATH_C2535	FGF17
**CARD11**	NM_032415.2:1075	NS_CANCERPATH_C2535	CARD11
**TNFRSF10C**	NM_003841.2:5	NS_CANCERPATH_C2535	TNFRSF10C
**ITGB7**	NM_000889.1:1278	NS_CANCERPATH_C2535	ITGB7
**NFKB1**	NM_003998.2:1675	NS_CANCERPATH_C2535	NFKB1
**CCNB1**	NM_031966.2:715	NS_CANCERPATH_C2535	CCNB1
**MMP3**	NM_002422.3:25	NS_CANCERPATH_C2535	MMP3
**IL6**	NM_000600.1:220	NS_CANCERPATH_C2535	IL6
**IL1B**	NM_000576.2:840	NS_CANCERPATH_C2535	IL1B
**IFNA17**	NM_021268.2:291	NS_CANCERPATH_C2535	IFNA17
**FGF23**	NM_020638.2:1390	NS_CANCERPATH_C2535	FGF23
**FANCA**	NM_000135.2:265	NS_CANCERPATH_C2535	FANCA
**CCNB3**	NM_033671.1:35	NS_CANCERPATH_C2535	CCNB3
**PRMT8**	NM_019854.3:1900	NS_CANCERPATH_C2535	PRMT8
**CBLC**	NM_012116.3:652	NS_CANCERPATH_C2535	CBLC
**IL12A**	NM_000882.2:775	NS_CANCERPATH_C2535	IL12A
**IL10**	NM_000572.2:230	NS_CANCERPATH_C2535	IL10
**CRLF2**	NM_001012288.1:605	NS_CANCERPATH_C2535	CRLF2
**BCL2A1**	NM_004049.2:80	NS_CANCERPATH_C2535	BCL2A1
**PLA2G5**	NM_000929.2:1375	NS_CANCERPATH_C2535	PLA2G5
**IL12RB2**	NM_001559.2:1315	NS_CANCERPATH_C2535	IL12RB2
**COL4A3**	NM_000091.3:5630	NS_CANCERPATH_C2535	COL4A3
**CALML6**	NM_138705.2:682	NS_CANCERPATH_C2535	CALML6
**FIGF**	NM_004469.2:580	NS_CANCERPATH_C2535	FIGF
**RET**	NM_020630.4:2911	NS_CANCERPATH_C2535	RET
**EGF**	NM_001963.3:3930	NS_CANCERPATH_C2535	EGF
**PTPN5**	NM_001039970.1:2354	NS_CANCERPATH_C2535	PTPN5
**PLA2G4E**	NM_001206670.1:4174	NS_CANCERPATH_C2535	PLA2G4E
**MPL**	NM_005373.2:895	NS_CANCERPATH_C2535	MPL
**ZBTB32**	NM_014383.1:1620	NS_CANCERPATH_C2535	ZBTB32
**EFNA2**	NM_001405.3:1538	NS_CANCERPATH_C2535	EFNA2

**DLL3**	NM_203486.2:1842	NS_CANCERPATH_C2535	DLL3
**CCNO**	NM_021147.3:45	NS_CANCERPATH_C2535	CCNO
**COL6A6**	NM_001102608.1:5926	NS_CANCERPATH_C2535	COL6A6
**IL23A**	NM_016584.2:411	NS_CANCERPATH_C2535	IL23A
**PAK7**	NM_177990.1:615	NS_CANCERPATH_C2535	PAK7
**CEBPE**	NM_001805.2:1096	NS_CANCERPATH_C2535	CEBPE
**IL24**	NM_181339.1:1016	NS_CANCERPATH_C2535	IL24
**CDC25C**	NM_001790.2:1055	NS_CANCERPATH_C2535	CDC25C
**PKMYT1**	NM_004203.3:780	NS_CANCERPATH_C2535	PKMYT1
**PCK1**	NM_002591.2:1870	NS_CANCERPATH_C2535	PCK1
**IL11**	NM_000641.2:1145	NS_CANCERPATH_C2535	IL11
**CACNG4**	NM_014405.2:1030	NS_CANCERPATH_C2535	CACNG4
**IL7**	NM_000880.2:38	NS_CANCERPATH_C2535	IL7
**BIRC7**	NM_022161.2:1168	NS_CANCERPATH_C2535	BIRC7
**RPA3**	NM_002947.3:990	NS_CANCERPATH_C2535	RPA3
**FZD10**	NM_007197.2:1810	NS_CANCERPATH_C2535	FZD10
**NTF3**	NM_002527.4:151	NS_CANCERPATH_C2535	NTF3
**MAPT**	NM_016834.3:1205	NS_CANCERPATH_C2535	MAPT
**FGF18**	NM_003862.1:850	NS_CANCERPATH_C2535	FGF18
**POLD1**	NM_002691.2:2392	NS_CANCERPATH_C2535	POLD1
**POLE2**	NM_002692.2:1420	NS_CANCERPATH_C2535	POLE2
**FEN1**	NM_004111.4:425	NS_CANCERPATH_C2535	FEN1
**NOG**	NM_005450.4:1543	NS_CANCERPATH_C2535	NOG
**FGFR4**	NM_002011.3:1585	NS_CANCERPATH_C2535	FGFR4
**FZD9**	NM_003508.2:1320	NS_CANCERPATH_C2535	FZD9
**AMH**	NM_000479.3:1626	NS_CANCERPATH_C2535	AMH
**TSLP**	NM_033035.4:899	NS_CANCERPATH_C2535	TSLP
**ETV4**	NM_001079675.1:1535	NS_CANCERPATH_C2535	ETV4
**RXRG**	NM_006917.3:1105	NS_CANCERPATH_C2535	RXRG
**TPO**	NM_175722.1:2400	NS_CANCERPATH_C2535	TPO
**MYCN**	NM_005378.4:1545	NS_CANCERPATH_C2535	MYCN
**CDC25A**	NM_001789.2:690	NS_CANCERPATH_C2535	CDC25A
**WNT6**	NM_006522.3:1200	NS_CANCERPATH_C2535	WNT6
**FGF22**	NM_020637.1:206	NS_CANCERPATH_C2535	FGF22
**ITGB6**	NM_001282353.1:4124	NS_CANCERPATH_C2535	ITGB6
**IL22RA1**	NM_021258.2:2524	NS_CANCERPATH_C2535	IL22RA1
**WNT10A**	NM_025216.2:2255	NS_CANCERPATH_C2535	WNT10A
**CCNE2**	NM_057735.1:50	NS_CANCERPATH_C2535	CCNE2
**IL20RB**	NM_144717.2:1575	NS_CANCERPATH_C2535	IL20RB
**HOXA10**	NM_018951.3:1503	NS_CANCERPATH_C2535	HOXA10
**TNR**	NM_003285.2:3154	NS_CANCERPATH_C2535	TNR
**GDF6**	NM_001001557.2:1908	NS_CANCERPATH_C2535	GDF6
**LRP2**	NM_004525.2:12505	NS_CANCERPATH_C2535	LRP2
**OSM**	NM_020530.3:1662	NS_CANCERPATH_C2535	OSM
**PTPRR**	NM_001207015.1:1652	NS_CANCERPATH_C2535	PTPRR
**PAX8**	NM_013953.3:3465	NS_CANCERPATH_C2535	PAX8
**DKK4**	NM_014420.2:640	NS_CANCERPATH_C2535	DKK4
**FGF21**	NM_019113.2:125	NS_CANCERPATH_C2535	FGF21
**HOXA9**	NM_152739.3:1015	NS_CANCERPATH_C2535	HOXA9
**HMGA2**	NM_003484.1:328	NS_CANCERPATH_C2535	HMGA2
**FGF3**	NM_005247.2:692	NS_CANCERPATH_C2535	FGF3
**IL3**	NM_000588.3:130	NS_CANCERPATH_C2535	IL3
**FGF4**	NM_002007.2:688	NS_CANCERPATH_C2535	FGF4
**WNT7A**	NM_004625.3:325	NS_CANCERPATH_C2535	WNT7A
**MMP7**	NM_002423.3:311	NS_CANCERPATH_C2535	MMP7
**FGF20**	NM_019851.1:390	NS_CANCERPATH_C2535	FGF20
**ACVR1C**	NM_145259.2:5168	NS_CANCERPATH_C2535	ACVR1C
**E2F1**	NM_005225.1:935	NS_CANCERPATH_C2535	E2F1
**SHC3**	NM_016848.5:6155	NS_CANCERPATH_C2535	SHC3
**COL1A1**	NM_000088.3:5210	NS_CANCERPATH_C2535	COL1A1

**COL3A1**	NM_000090.3:180	NS_CANCERPATH_C2535	COL3A1
**COL1A2**	NM_000089.3:2635	NS_CANCERPATH_C2535	COL1A2
**B2M**	NM_004048.2:25	NS_CANCERPATH_C2535	B2M
**RPS27A**	NM_002954.5:650	NS_CANCERPATH_C2535	RPS27A
**SPP1**	NM_000582.2:760	NS_CANCERPATH_C2535	SPP1
**SFRP2**	NM_003013.2:840	NS_CANCERPATH_C2535	SFRP2
**WIF1**	NM_007191.2:765	NS_CANCERPATH_C2535	WIF1
**SFRP4**	NM_003014.2:1060	NS_CANCERPATH_C2535	SFRP4
**LEPR**	NM_001003679.1:2000	NS_CANCERPATH_C2535	LEPR
**NR4A1**	NM_173157.1:1575	NS_CANCERPATH_C2535	NR4A1
**SETBP1**	NM_015559.2:704	NS_CANCERPATH_C2535	SETBP1
**COL27A1**	NM_032888.2:5120	NS_CANCERPATH_C2535	COL27A1
**IL11RA**	NM_147162.1:400	NS_CANCERPATH_C2535	IL11RA
**PBX1**	NM_002585.2:368	NS_CANCERPATH_C2535	PBX1
**DDIT4**	NM_019058.2:85	NS_CANCERPATH_C2535	DDIT4
**MYC**	NM_002467.3:1610	NS_CANCERPATH_C2535	MYC
**NFKBIA**	NM_020529.1:945	NS_CANCERPATH_C2535	NFKBIA
**SOST**	NM_025237.2:980	NS_CANCERPATH_C2535	SOST
**PIK3R1**	NM_181504.2:1105	NS_CANCERPATH_C2535	PIK3R1
**PML**	NM_002675.3:281	NS_CANCERPATH_C2535	PML
**HSPB1**	NM_001540.3:374	NS_CANCERPATH_C2535	HSPB1
**AKT2**	NM_001626.2:1450	NS_CANCERPATH_C2535	AKT2
**GNA11**	NM_002067.1:555	NS_CANCERPATH_C2535	GNA11
**ATRX**	NM_000489.3:139	NS_CANCERPATH_C2535	ATRX
**IGF1R**	NM_000875.2:455	NS_CANCERPATH_C2535	IGF1R
**SF3B1**	NM_001005526.1:0	NS_CANCERPATH_C2535	SF3B1
**CCND1**	NM_053056.2:690	NS_CANCERPATH_C2535	CCND1
**JAK1**	NM_002227.1:285	NS_CANCERPATH_C2535	JAK1
**NCOR1**	NM_006311.3:1390	NS_CANCERPATH_C2535	NCOR1
**BAP1**	NM_004656.2:240	NS_CANCERPATH_C2535	BAP1
**SMARCB1**	NM_003073.3:1060	NS_CANCERPATH_C2535	SMARCB1
**SRSF2**	NM_003016.3:312	NS_CANCERPATH_C2535	SRSF2
**VEGFA**	NM_001025366.1:1325	NS_CANCERPATH_C2535	VEGFA
**ETS2**	NM_005239.4:1175	NS_CANCERPATH_C2535	ETS2
**H3F3C**	NM_001013699.2:829	NS_CANCERPATH_C2535	H3F3C
**SKP1**	NM_170679.2:630	NS_CANCERPATH_C2535	SKP1
**ID1**	NM_002165.2:345	NS_CANCERPATH_C2535	ID1
**PDGFRA**	NM_006206.3:1925	NS_CANCERPATH_C2535	PDGFRA
**STAT3**	NM_139276.2:4535	NS_CANCERPATH_C2535	STAT3
**SHC1**	NM_183001.4:3355	NS_CANCERPATH_C2535	SHC1
**NOTCH2**	NM_024408.3:2842	NS_CANCERPATH_C2535	NOTCH2
**AKT1**	NM_005163.2:1772	NS_CANCERPATH_C2535	AKT1
**U2AF1**	NM_001025203.1:400	NS_CANCERPATH_C2535	U2AF1
**PDGFRB**	NM_002609.3:840	NS_CANCERPATH_C2535	PDGFRB
**CAPN2**	NM_001748.4:2085	NS_CANCERPATH_C2535	CAPN2
**NOTCH3**	NM_000435.2:1965	NS_CANCERPATH_C2535	NOTCH3
**PLAT**	NM_000931.2:1334	NS_CANCERPATH_C2535	PLAT
**TGFBR2**	NM_001024847.1:1760	NS_CANCERPATH_C2535	TGFBR2
**FGFR3**	NM_022965.2:3170	NS_CANCERPATH_C2535	FGFR3
**COL5A2**	NM_000393.3:4075	NS_CANCERPATH_C2535	COL5A2
**IGFBP3**	NM_000598.4:1255	NS_CANCERPATH_C2535	IGFBP3
**LIFR**	NM_002310.3:2995	NS_CANCERPATH_C2535	LIFR
**GNAS**	NM_080425.1:1910	NS_CANCERPATH_C2535	GNAS
**FLNA**	NM_001456.3:7335	NS_CANCERPATH_C2535	FLNA
**FN1**	NM_212482.1:1776	NS_CANCERPATH_C2535	FN1
**HSPA1A**	NM_005345.5:98	NS_CANCERPATH_C2535	HSPA1A
**GADD45B**	NM_015675.2:365	NS_CANCERPATH_C2535	GADD45B
**FOS**	NM_005252.2:1475	NS_CANCERPATH_C2535	FOS
**UBB**	NM_018955.2:15	NS_CANCERPATH_C2535	UBB
**CTNNB1**	NM_001904.3:2265	NS_CANCERPATH_C2535	CTNNB1

**H3F3A**	NM_002107.3:190	NS_CANCERPATH_C2535	H3F3A
**TGFB1**	NM_000660.3:1260	NS_CANCERPATH_C2535	TGFB1
**FGFR1**	NM_015850.2:1335	NS_CANCERPATH_C2535	FGFR1
**MMP9**	NM_004994.2:1530	NS_CANCERPATH_C2535	MMP9
**THBS1**	NM_003246.2:3465	NS_CANCERPATH_C2535	THBS1
**COL11A1**	NM_001854.3:674	NS_CANCERPATH_C2535	COL11A1
**COL5A1**	NM_000093.3:6345	NS_CANCERPATH_C2535	COL5A1
**CHAD**	NM_001267.2:810	NS_CANCERPATH_C2535	CHAD
**IL8**	NM_000584.2:25	NS_CANCERPATH_C2535	IL8
**PAX3**	NM_013942.3:705	NS_CANCERPATH_C2535	PAX3
**ZBTB16**	NM_006006.4:1585	NS_CANCERPATH_C2535	ZBTB16
**WEE1**	NM_003390.3:1225	NS_CANCERPATH_C2535	WEE1
**EYA1**	NM_172059.2:1090	NS_CANCERPATH_C2535	EYA1
**ITGA6**	NM_000210.1:3065	NS_CANCERPATH_C2535	ITGA6
**SPRY1**	NM_005841.1:810	NS_CANCERPATH_C2535	SPRY1
**TNFSF10**	NM_003810.2:115	NS_CANCERPATH_C2535	TNFSF10
**EGFR**	NM_201282.1:360	NS_CANCERPATH_C2535	EGFR
**ITGB4**	NM_001005731.1:4151	NS_CANCERPATH_C2535	ITGB4
**NR4A3**	NM_173198.1:2590	NS_CANCERPATH_C2535	NR4A3
**FGF7**	NM_002009.3:190	NS_CANCERPATH_C2535	FGF7
**PLCB1**	NM_182734.1:170	NS_CANCERPATH_C2535	PLCB1
**MAP3K14**	NM_003954.1:620	NS_CANCERPATH_C2535	MAP3K14
**COMP**	NM_000095.2:1744	NS_CANCERPATH_C2535	COMP
**CACNA1G**	NM_198397.1:1380	NS_CANCERPATH_C2535	CACNA1G
**PLCE1**	NM_001165979.1:392	NS_CANCERPATH_C2535	PLCE1
**FGF2**	NM_002006.4:620	NS_CANCERPATH_C2535	FGF2
**EFNA5**	NM_001962.2:5035	NS_CANCERPATH_C2535	EFNA5
**AR**	NM_001011645.1:810	NS_CANCERPATH_C2535	AR
**RASGRP2**	NM_001098670.1:1978	NS_CANCERPATH_C2535	RASGRP2
**BMP5**	NM_021073.2:1570	NS_CANCERPATH_C2535	BMP5
**CNTFR**	NM_147164.1:784	NS_CANCERPATH_C2535	CNTFR
**ANGPT1**	NM_001146.3:2080	NS_CANCERPATH_C2535	ANGPT1
**SFRP1**	NM_003012.3:3320	NS_CANCERPATH_C2535	SFRP1
**NOTCH1**	NM_017617.3:735	NS_CANCERPATH_C2535	NOTCH1
**IKBKB**	NM_001556.1:1995	NS_CANCERPATH_C2535	IKBKB
**TSPAN7**	NM_004615.3:725	NS_CANCERPATH_C2535	TSPAN7
**ID4**	NM_001546.2:2048	NS_CANCERPATH_C2535	ID4
**NTRK2**	NM_001007097.1:1605	NS_CANCERPATH_C2535	NTRK2
**KDM6A**	NM_021140.2:2590	NS_CANCERPATH_C2535	KDM6A
**MAP2K1**	NM_002755.2:970	NS_CANCERPATH_C2535	MAP2K1
**DUSP6**	NM_001946.2:1535	NS_CANCERPATH_C2535	DUSP6
**MAPK1**	NM_138957.2:430	NS_CANCERPATH_C2535	MAPK1
**TP53**	NM_000546.2:1330	NS_CANCERPATH_C2535	TP53
**RB1**	NM_000321.1:2110	NS_CANCERPATH_C2535	RB1
**GSK3B**	NM_002093.2:925	NS_CANCERPATH_C2535	GSK3B
**TCF7L1**	NM_031283.1:2215	NS_CANCERPATH_C2535	TCF7L1
**CDKN1C**	NM_000076.2:1605	NS_CANCERPATH_C2535	CDKN1C
**MAP3K1**	NM_005921.1:2525	NS_CANCERPATH_C2535	MAP3K1
**TIAM1**	NM_003253.2:5620	NS_CANCERPATH_C2535	TIAM1
**MYD88**	NM_002468.3:2145	NS_CANCERPATH_C2535	MYD88
**SMARCA4**	NM_003072.3:5400	NS_CANCERPATH_C2535	SMARCA4
**DAXX**	NM_001350.3:1875	NS_CANCERPATH_C2535	DAXX
**PLCB4**	NM_000933.3:215	NS_CANCERPATH_C2535	PLCB4
**PIK3R3**	NM_003629.3:1800	NS_CANCERPATH_C2535	PIK3R3
**SKP2**	NM_005983.2:615	NS_CANCERPATH_C2535	SKP2
**TSC1**	NM_000368.3:100	NS_CANCERPATH_C2535	TSC1
**TET2**	NM_001127208.2:2882	NS_CANCERPATH_C2535	TET2
**DNMT3A**	NM_022552.3:3835	NS_CANCERPATH_C2535	DNMT3A
**SP1**	NM_003109.1:5970	NS_CANCERPATH_C2535	SP1
**NSD1**	NM_022455.4:10655	NS_CANCERPATH_C2535	NSD1

**XPA**	NM_000380.3:265	NS_CANCERPATH_C2535	XPA
**MDC1**	NM_014641.2:6719	NS_CANCERPATH_C2535	MDC1
**CHUK**	NM_001278.3:860	NS_CANCERPATH_C2535	CHUK
**TNFRSF10B**	NM_003842.3:565	NS_CANCERPATH_C2535	TNFRSF10B
**KAT2B**	NM_003884.3:1220	NS_CANCERPATH_C2535	KAT2B
**PPP3CC**	NM_005605.3:1460	NS_CANCERPATH_C2535	PPP3CC
**PIK3CA**	NM_006218.2:2445	NS_CANCERPATH_C2535	PIK3CA
**FZD3**	NM_017412.2:435	NS_CANCERPATH_C2535	FZD3
**POLD4**	NM_021173.2:470	NS_CANCERPATH_C2535	POLD4
**NTHL1**	NM_002528.5:476	NS_CANCERPATH_C2535	NTHL1
**DNMT1**	NM_001379.2:1495	NS_CANCERPATH_C2535	DNMT1
**MTOR**	NM_004958.2:5095	NS_CANCERPATH_C2535	MTOR
**JAK3**	NM_000215.2:1715	NS_CANCERPATH_C2535	JAK3
**SHC2**	NM_012435.2:698	NS_CANCERPATH_C2535	SHC2
**MAP3K12**	NM_006301.2:800	NS_CANCERPATH_C2535	MAP3K12
**DDB2**	NM_000107.1:840	NS_CANCERPATH_C2535	DDB2
**THEM4**	NM_053055.4:764	NS_CANCERPATH_C2535	THEM4
**PIK3CD**	NM_005026.3:95	NS_CANCERPATH_C2535	PIK3CD
**EPOR**	NM_000121.2:1295	NS_CANCERPATH_C2535	EPOR
**FANCC**	NM_000136.2:310	NS_CANCERPATH_C2535	FANCC
**DLL4**	NM_019074.2:893	NS_CANCERPATH_C2535	DLL4
**KIT**	NM_000222.1:5	NS_CANCERPATH_C2535	KIT
**PLD1**	NM_002662.3:1265	NS_CANCERPATH_C2535	PLD1
**RAC3**	NM_005052.2:702	NS_CANCERPATH_C2535	RAC3
**PBX3**	NM_006195.5:2050	NS_CANCERPATH_C2535	PBX3
**IGF1**	NM_000618.3:491	NS_CANCERPATH_C2535	IGF1
**DTX3**	NM_178502.2:1620	NS_CANCERPATH_C2535	DTX3
**CBL**	NM_005188.2:7485	NS_CANCERPATH_C2535	CBL
**COL24A1**	NM_152890.5:4752	NS_CANCERPATH_C2535	COL24A1
**FUT8**	NM_004480.4:2841	NS_CANCERPATH_C2535	FUT8
**STAG2**	NM_001042749.1:4040	NS_CANCERPATH_C2535	STAG2
**HIST1H3H**	NM_003536.2:355	NS_CANCERPATH_C2535	HIST1H3H
**FZD8**	NM_031866.1:890	NS_CANCERPATH_C2535	FZD8
**NUPR1**	NM_001042483.1:829	NS_CANCERPATH_C2535	NUPR1
**EIF4EBP1**	NM_004095.3:363	NS_CANCERPATH_C2535	EIF4EBP1
**PTCH1**	NM_000264.3:5420	NS_CANCERPATH_C2535	PTCH1
**PBRM1**	NM_181042.3:6100	NS_CANCERPATH_C2535	PBRM1
**PPP3CA**	NM_000944.4:3920	NS_CANCERPATH_C2535	PPP3CA
**KRAS**	NM_004985.3:1790	NS_CANCERPATH_C2535	KRAS
**SPRY4**	NM_030964.3:1900	NS_CANCERPATH_C2535	SPRY4
**FOXO4**	NM_005938.2:35	NS_CANCERPATH_C2535	FOXO4
**TCF3**	NM_003200.2:4325	NS_CANCERPATH_C2535	TCF3
**IL7R**	NM_002185.2:1610	NS_CANCERPATH_C2535	IL7R
**CDC14A**	NM_033313.2:1400	NS_CANCERPATH_C2535	CDC14A
**BCOR**	NM_001123383.1:1630	NS_CANCERPATH_C2535	BCOR
**BNIP3**	NM_004052.2:325	NS_CANCERPATH_C2535	BNIP3
**VEGFC**	NM_005429.2:565	NS_CANCERPATH_C2535	VEGFC
**SIN3A**	NM_015477.1:1605	NS_CANCERPATH_C2535	SIN3A
**MSH6**	NM_000179.1:3525	NS_CANCERPATH_C2535	MSH6
**CDC14B**	NM_003671.3:4940	NS_CANCERPATH_C2535	CDC14B
**ATM**	NM_138292.3:6688	NS_CANCERPATH_C2535	ATM
**PRKCA**	NM_002737.2:5560	NS_CANCERPATH_C2535	PRKCA
**ARID1A**	NM_006015.4:5495	NS_CANCERPATH_C2535	ARID1A
**MAP2K4**	NM_003010.2:2830	NS_CANCERPATH_C2535	MAP2K4
**CDC25B**	NM_021873.2:3045	NS_CANCERPATH_C2535	CDC25B
**SMO**	NM_005631.3:1615	NS_CANCERPATH_C2535	SMO
**POLR2D**	NM_004805.3:1476	NS_CANCERPATH_C2535	POLR2D
**DTX4**	NM_015177.1:4820	NS_CANCERPATH_C2535	DTX4
**PDGFA**	NM_002607.5:2460	NS_CANCERPATH_C2535	PDGFA
**MAD2L2**	NM_001127325.1:290	NS_CANCERPATH_C2535	MAD2L2

**GLI3**	NM_000168.5:4769	NS_CANCERPATH_C2535	GLI3
**CDK6**	NM_001259.5:15	NS_CANCERPATH_C2535	CDK6
**HDAC4**	NM_006037.3:6965	NS_CANCERPATH_C2535	HDAC4
**SMAD9**	NM_005905.2:1595	NS_CANCERPATH_C2535	SMAD9
**FLT1**	NM_002019.4:530	NS_CANCERPATH_C2535	FLT1
**ITGA2**	NM_002203.2:475	NS_CANCERPATH_C2535	ITGA2
**MAML2**	NM_032427.1:4125	NS_CANCERPATH_C2535	MAML2
**STMN1**	NM_203401.1:478	NS_CANCERPATH_C2535	STMN1
**CDKN2C**	NM_001262.2:1295	NS_CANCERPATH_C2535	CDKN2C
**TLR4**	NM_138554.2:2570	NS_CANCERPATH_C2535	TLR4
**NOS3**	NM_000603.4:1456	NS_CANCERPATH_C2535	NOS3
**MAP2K6**	NM_002758.3:555	NS_CANCERPATH_C2535	MAP2K6
**APC**	NM_000038.3:6850	NS_CANCERPATH_C2535	APC
**RIN1**	NM_004292.2:2572	NS_CANCERPATH_C2535	RIN1
**MAP3K13**	NM_004721.3:965	NS_CANCERPATH_C2535	MAP3K13
**LEF1**	NM_016269.3:1165	NS_CANCERPATH_C2535	LEF1
**RASGRF2**	NM_006909.1:2675	NS_CANCERPATH_C2535	RASGRF2
**CDK4**	NM_000075.2:1055	NS_CANCERPATH_C2535	CDK4
**GTF2H3**	NM_001516.3:70	NS_CANCERPATH_C2535	GTF2H3
**WNT5B**	NM_032642.2:1745	NS_CANCERPATH_C2535	WNT5B
**MED12**	NM_005120.2:375	NS_CANCERPATH_C2535	MED12
**MAPK8**	NM_002750.2:945	NS_CANCERPATH_C2535	MAPK8
**FAS**	NM_152876.1:1740	NS_CANCERPATH_C2535	FAS
**RAD50**	NM_005732.2:5397	NS_CANCERPATH_C2535	RAD50
**MNAT1**	NM_002431.2:975	NS_CANCERPATH_C2535	MNAT1
**CASP3**	NM_032991.2:685	NS_CANCERPATH_C2535	CASP3
**ALKBH3**	NM_139178.3:690	NS_CANCERPATH_C2535	ALKBH3
**CASP7**	NM_001227.3:915	NS_CANCERPATH_C2535	CASP7
**BMP6**	NM_001718.2:1045	NS_CANCERPATH_C2535	BMP6
**RRAS2**	NM_001102669.2:1785	NS_CANCERPATH_C2535	RRAS2
**GADD45G**	NM_006705.3:250	NS_CANCERPATH_C2535	GADD45G
**HSPA6**	NM_002155.3:2018	NS_CANCERPATH_C2535	HSPA6
**TLR2**	NM_003264.3:180	NS_CANCERPATH_C2535	TLR2
**SIX1**	NM_005982.3:25	NS_CANCERPATH_C2535	SIX1
**AXIN1**	NM_181050.1:135	NS_CANCERPATH_C2535	AXIN1
**TGFB2**	NM_003238.2:1125	NS_CANCERPATH_C2535	TGFB2
**DKK1**	NM_012242.2:75	NS_CANCERPATH_C2535	DKK1
**TNC**	NM_002160.3:4	NS_CANCERPATH_C2535	TNC
**PGF**	NM_002632.5:1047	NS_CANCERPATH_C2535	PGF
**PRDM1**	NM_182907.1:310	NS_CANCERPATH_C2535	PRDM1
**GATA2**	NM_032638.3:1495	NS_CANCERPATH_C2535	GATA2
**STAT4**	NM_003151.2:789	NS_CANCERPATH_C2535	STAT4
**DLL1**	NM_005618.3:2580	NS_CANCERPATH_C2535	DLL1
**MET**	NM_000245.2:405	NS_CANCERPATH_C2535	MET
**CCND2**	NM_001759.2:5825	NS_CANCERPATH_C2535	CCND2
**MECOM**	NM_005241.2:3355	NS_CANCERPATH_C2535	MECOM
**ITGA3**	NM_005501.2:1138	NS_CANCERPATH_C2535	ITGA3
**RPS6KA5**	NM_004755.2:855	NS_CANCERPATH_C2535	RPS6KA5
**ITGB8**	NM_002214.2:2609	NS_CANCERPATH_C2535	ITGB8
**CACNA1H**	NM_021098.2:3329	NS_CANCERPATH_C2535	CACNA1H
**BCL2**	NM_000657.2:5	NS_CANCERPATH_C2535	BCL2
**MAP3K8**	NM_005204.2:2050	NS_CANCERPATH_C2535	MAP3K8
**ITGA7**	NM_002206.1:1170	NS_CANCERPATH_C2535	ITGA7
**IRAK3**	NM_007199.1:1735	NS_CANCERPATH_C2535	IRAK3
**ITGA9**	NM_002207.2:2380	NS_CANCERPATH_C2535	ITGA9
**LAT**	NM_001014987.1:1290	NS_CANCERPATH_C2535	LAT
**WHSC1**	NM_007331.1:1260	NS_CANCERPATH_C2535	WHSC1
**MSH2**	NM_000251.1:2105	NS_CANCERPATH_C2535	MSH2
**FANCL**	NM_001114636.1:446	NS_CANCERPATH_C2535	FANCL
**PLCG2**	NM_002661.2:525	NS_CANCERPATH_C2535	PLCG2

**HGF**	NM_000601.4:550	NS_CANCERPATH_C2535	HGF
**LFNG**	NM_001040168.1:717	NS_CANCERPATH_C2535	LFNG
**BAMBI**	NM_012342.2:832	NS_CANCERPATH_C2535	BAMBI
**BMP8A**	NM_181809.3:5015	NS_CANCERPATH_C2535	BMP8A
**ITGB3**	NM_000212.2:4485	NS_CANCERPATH_C2535	ITGB3
**WNT5A**	NM_003392.3:475	NS_CANCERPATH_C2535	WNT5A
**NKD1**	NM_033119.3:2325	NS_CANCERPATH_C2535	NKD1
**INHBA**	NM_002192.2:490	NS_CANCERPATH_C2535	INHBA
**BMP2**	NM_001200.2:1515	NS_CANCERPATH_C2535	BMP2
**THBS4**	NM_003248.3:985	NS_CANCERPATH_C2535	THBS4
**PLAU**	NM_002658.2:793	NS_CANCERPATH_C2535	PLAU
**TRAF7**	NM_032271.2:1105	NS_CANCERPATH_C2535	TRAF7
**MCM7**	NM_182776.1:1325	NS_CANCERPATH_C2535	MCM7
**CIC**	NM_015125.3:1290	NS_CANCERPATH_C2535	CIC
**HDAC5**	NM_005474.4:3160	NS_CANCERPATH_C2535	HDAC5
**ARID2**	NM_152641.2:3355	NS_CANCERPATH_C2535	ARID2
**NF2**	NM_181828.2:455	NS_CANCERPATH_C2535	NF2
**JAK2**	NM_004972.2:455	NS_CANCERPATH_C2535	JAK2
**MLLT4**	NM_005936.2:1560	NS_CANCERPATH_C2535	MLLT4
**CYLD**	NM_015247.1:2890	NS_CANCERPATH_C2535	CYLD
**KMT2D**	NM_003482.3:6070	NS_CANCERPATH_C2535	KMT2D
**KDM5C**	NM_004187.2:1170	NS_CANCERPATH_C2535	KDM5C
**WHSC1L1**	NM_017778.2:485	NS_CANCERPATH_C2535	WHSC1L1
**KMT2C**	NM_170606.2:12505	NS_CANCERPATH_C2535	KMT2C
**PDGFD**	NM_025208.4:1120	NS_CANCERPATH_C2535	PDGFD
**SMAD3**	NM_005902.3:4220	NS_CANCERPATH_C2535	SMAD3
**RHOA**	NM_001664.2:1230	NS_CANCERPATH_C2535	RHOA
**LTBP1**	NM_000627.3:4124	NS_CANCERPATH_C2535	LTBP1
**TBL1XR1**	NM_024665.4:915	NS_CANCERPATH_C2535	TBL1XR1
**PTEN**	NM_000314.3:1675	NS_CANCERPATH_C2535	PTEN
**SMAD2**	NM_001003652.1:4500	NS_CANCERPATH_C2535	SMAD2
**PRKDC**	NM_006904.6:12750	NS_CANCERPATH_C2535	PRKDC
**RBX1**	NM_014248.2:162	NS_CANCERPATH_C2535	RBX1
**EPHA2**	NM_004431.2:1525	NS_CANCERPATH_C2535	EPHA2
**SMAD4**	NM_005359.3:1370	NS_CANCERPATH_C2535	SMAD4
**MAP2K2**	NM_030662.2:1325	NS_CANCERPATH_C2535	MAP2K2
**PPP3R1**	NM_000945.3:2385	NS_CANCERPATH_C2535	PPP3R1
**BCL2L1**	NM_138578.1:1560	NS_CANCERPATH_C2535	BCL2L1
**GNG12**	NM_018841.3:245	NS_CANCERPATH_C2535	GNG12
**AKT3**	NM_181690.1:755	NS_CANCERPATH_C2535	AKT3
**MDM2**	NM_006878.2:280	NS_CANCERPATH_C2535	MDM2
**SOCS2**	NM_003877.3:1020	NS_CANCERPATH_C2535	SOCS2
**GNAQ**	NM_002072.2:1100	NS_CANCERPATH_C2535	GNAQ
**RAD21**	NM_006265.2:1080	NS_CANCERPATH_C2535	RAD21
**IBSP**	NM_004967.3:876	NS_CANCERPATH_C2535	IBSP
**CREB3L1**	NM_052854.1:195	NS_CANCERPATH_C2535	CREB3L1
**COL11A2**	NM_001163771.1:760	NS_CANCERPATH_C2535	COL11A2
**SOCS3**	NM_003955.3:1870	NS_CANCERPATH_C2535	SOCS3
**SPRY2**	NM_005842.2:85	NS_CANCERPATH_C2535	SPRY2
**DUSP5**	NM_004419.3:675	NS_CANCERPATH_C2535	DUSP5
**NFKBIZ**	NM_001005474.1:2030	NS_CANCERPATH_C2535	NFKBIZ
**TNFAIP3**	NM_006290.2:260	NS_CANCERPATH_C2535	TNFAIP3
**RUNX1**	NM_001754.4:635	NS_CANCERPATH_C2535	RUNX1
**LAMA5**	NM_005560.3:787	NS_CANCERPATH_C2535	LAMA5
**RUNX1T1**	NM_004349.2:1085	NS_CANCERPATH_C2535	RUNX1T1
**CACNA2D1**	NM_000722.2:335	NS_CANCERPATH_C2535	CACNA2D1
**FGFR2**	NM_000141.4:647	NS_CANCERPATH_C2535	FGFR2
**IKBKG**	NM_003639.2:470	NS_CANCERPATH_C2535	IKBKG
**SOS1**	NM_005633.2:1635	NS_CANCERPATH_C2535	SOS1
**HDAC10**	NM_032019.5:932	NS_CANCERPATH_C2535	HDAC10

**CASP8**	NM_001228.4:301	NS_CANCERPATH_C2535	CASP8
**PPP3CB**	NM_001142354.1:1690	NS_CANCERPATH_C2535	PPP3CB
**TFDP1**	NM_007111.4:1826	NS_CANCERPATH_C2535	TFDP1
**TGFB3**	NM_003239.2:2825	NS_CANCERPATH_C2535	TGFB3
**ERBB2**	NM_004448.2:2380	NS_CANCERPATH_C2535	ERBB2
**NGFR**	NM_002507.1:2705	NS_CANCERPATH_C2535	NGFR
**MAPK3**	NM_001040056.1:580	NS_CANCERPATH_C2535	MAPK3
**HDAC1**	NM_004964.2:785	NS_CANCERPATH_C2535	HDAC1
**SMC1A**	NM_006306.2:2320	NS_CANCERPATH_C2535	SMC1A
**MLH1**	NM_000249.2:1605	NS_CANCERPATH_C2535	MLH1
**STK11**	NM_000455.4:2060	NS_CANCERPATH_C2535	STK11
**NUMBL**	NM_004756.3:591	NS_CANCERPATH_C2535	NUMBL
**NBN**	NM_001024688.1:1105	NS_CANCERPATH_C2535	NBN
**VHL**	NM_000551.2:1280	NS_CANCERPATH_C2535	VHL
**ATR**	NM_001184.2:565	NS_CANCERPATH_C2535	ATR
**POLR2H**	NM_001278698.1:940	NS_CANCERPATH_C2535	POLR2H
**HDAC6**	NM_006044.2:536	NS_CANCERPATH_C2535	HDAC6
**RAD52**	NM_134424.2:297	NS_CANCERPATH_C2535	RAD52
**JAG2**	NM_145159.1:4225	NS_CANCERPATH_C2535	JAG2
**IDH2**	NM_002168.2:944	NS_CANCERPATH_C2535	IDH2
**GRB2**	NM_002086.4:412	NS_CANCERPATH_C2535	GRB2
**PIK3R2**	NM_005027.2:3100	NS_CANCERPATH_C2535	PIK3R2
**BAD**	NM_004322.3:652	NS_CANCERPATH_C2535	BAD
**BMP4**	NM_001202.2:490	NS_CANCERPATH_C2535	BMP4
**AXIN2**	NM_004655.3:1035	NS_CANCERPATH_C2535	AXIN2
**FUBP1**	NM_003902.3:820	NS_CANCERPATH_C2535	FUBP1
**KITLG**	NM_003994.4:1155	NS_CANCERPATH_C2535	KITLG
**STAT1**	NM_007315.2:205	NS_CANCERPATH_C2535	STAT1
**NFE2L2**	NM_006164.3:995	NS_CANCERPATH_C2535	NFE2L2
**HDAC2**	NM_001527.1:930	NS_CANCERPATH_C2535	HDAC2
**RAC1**	NM_198829.1:1250	NS_CANCERPATH_C2535	RAC1
**JAG1**	NM_000214.2:915	NS_CANCERPATH_C2535	JAG1
**GPC4**	NM_001448.2:820	NS_CANCERPATH_C2535	GPC4
**RELA**	NM_021975.3:1990	NS_CANCERPATH_C2535	RELA
**CCND3**	NM_001760.2:1215	NS_CANCERPATH_C2535	CCND3
**CD14**	NM_000591.2:885	NS_CANCERPATH_C2535	CD14
**HSP90B1**	NM_003299.1:160	NS_CANCERPATH_C2535	HSP90B1
**IL1R1**	NM_000877.2:4295	NS_CANCERPATH_C2535	IL1R1
**FST**	NM_006350.2:575	NS_CANCERPATH_C2535	FST
**GADD45A**	NM_001924.2:865	NS_CANCERPATH_C2535	GADD45A
**FZD7**	NM_003507.1:1890	NS_CANCERPATH_C2535	FZD7
**GAS1**	NM_002048.2:1525	NS_CANCERPATH_C2535	GAS1
**HES1**	NM_005524.2:860	NS_CANCERPATH_C2535	HES1
**PIM1**	NM_002648.2:1630	NS_CANCERPATH_C2535	PIM1
**ABL1**	NM_005157.3:3200	NS_CANCERPATH_C2535	ABL1
**DDIT3**	NM_004083.4:40	NS_CANCERPATH_C2535	DDIT3
**MEN1**	NM_130802.2:2480	NS_CANCERPATH_C2535	MEN1
**ASXL1**	NM_001164603.1:472	NS_CANCERPATH_C2535	ASXL1
**BMP7**	NM_001719.1:525	NS_CANCERPATH_C2535	BMP7
**KLF4**	NM_004235.4:1980	NS_CANCERPATH_C2535	KLF4
**JUN**	NM_002228.3:140	NS_CANCERPATH_C2535	JUN
**ID2**	NM_002166.4:505	NS_CANCERPATH_C2535	ID2
**CDKN1A**	NM_000389.2:1975	NS_CANCERPATH_C2535	CDKN1A
**PTPN11**	NM_002834.3:1480	NS_CANCERPATH_C2535	PTPN11
**RASA4**	NM_001079877.2:5330	NS_CANCERPATH_C2535	RASA4
**MGMT**	NM_002412.3:323	NS_CANCERPATH_C2535	MGMT
**BAX**	NM_138761.3:342	NS_CANCERPATH_C2535	BAX
**PRKACA**	NM_002730.3:400	NS_CANCERPATH_C2535	PRKACA
**NF1**	NM_000267.2:1035	NS_CANCERPATH_C2535	NF1
**SMC3**	NM_005445.3:3525	NS_CANCERPATH_C2535	SMC3

**CUL1**	NM_003592.2:1487	NS_CANCERPATH_C2535	CUL1
**PRKAR2A**	NM_004157.2:1590	NS_CANCERPATH_C2535	PRKAR2A
**RAF1**	NM_002880.2:1990	NS_CANCERPATH_C2535	RAF1
**ARID1B**	NM_020732.3:6335	NS_CANCERPATH_C2535	ARID1B
**SOS2**	NM_006939.2:3845	NS_CANCERPATH_C2535	SOS2
**EP300**	NM_001429.2:715	NS_CANCERPATH_C2535	EP300
**SETD2**	NM_014159.6:6160	NS_CANCERPATH_C2535	SETD2
**BRAF**	NM_004333.3:565	NS_CANCERPATH_C2535	BRAF
**PDGFC**	NM_016205.1:10	NS_CANCERPATH_C2535	PDGFC
**PPP2CB**	NM_001009552.1:1075	NS_CANCERPATH_C2535	PPP2CB
**PPP2R1A**	NM_014225.3:1440	NS_CANCERPATH_C2535	PPP2R1A

Analyte.Type	Is.Control	Control.Type	Probe.Annotation
mRNA	0		DNA Damage - Repair
mRNA	0		Ras
mRNA	0		JAK-STAT
mRNA	0		JAK-STAT
mRNA	0		Ras
mRNA	0		Ras
mRNA	0		JAK-STAT
mRNA	0		Ras
mRNA	0		Driver Gene
mRNA	0		Cell Cycle - Apoptosis
mRNA	0		Transcriptional Misregulation
mRNA	0		Driver Gene
mRNA	0		JAK-STAT
mRNA	0		Transcriptional Misregulation
mRNA	0		Ras
mRNA	0		Ras
mRNA	0		Driver Gene
mRNA	0		PI3K
mRNA	0		Hedgehog
mRNA	0		PI3K
mRNA	0		Transcriptional Misregulation
mRNA	0		PI3K
mRNA	0		Ras
mRNA	0		Ras
mRNA	0		Cell Cycle - Apoptosis
mRNA	0		JAK-STAT
mRNA	0		MAPK
mRNA	0		Driver Gene
mRNA	0		TGF-beta
mRNA	0		TGF-beta
mRNA	0		Driver Gene
mRNA	0		JAK-STAT
mRNA	0		Cell Cycle - Apoptosis
mRNA	0		Transcriptional Misregulation
mRNA	0		Transcriptional Misregulation
mRNA	0		Transcriptional Misregulation
mRNA	0		PI3K
mRNA	0		MAPK
mRNA	0		Transcriptional Misregulation
mRNA	0		JAK-STAT
mRNA	0		Ras
mRNA	0		Transcriptional Misregulation
mRNA	0		Ras
mRNA	0		Transcriptional Misregulation
mRNA	0		PI3K
mRNA	0		JAK-STAT
mRNA	0		PI3K
mRNA	0		PI3K
mRNA	0		Transcriptional Misregulation
mRNA	0		Cell Cycle - Apoptosis
mRNA	0		PI3K
mRNA	0		JAK-STAT
mRNA	0		Ras
mRNA	0		PI3K
mRNA	0		PI3K
mRNA	0		Ras
mRNA	0		JAK-STAT
mRNA	0		PI3K
mRNA	0		Ras

mRNA	0	Driver Gene
mRNA	0	PI3K
mRNA	0	PI3K
mRNA	0	Transcriptional Misregulation
mRNA	0	Transcriptional Misregulation
mRNA	0	Hedgehog
mRNA	0	PI3K
mRNA	0	Ras
mRNA	0	Transcriptional Misregulation
mRNA	0	JAK-STAT
mRNA	0	Ras
mRNA	0	MAPK
mRNA	0	Cell Cycle - Apoptosis
mRNA	0	MAPK
mRNA	0	Transcriptional Misregulation
mRNA	0	PI3K
mRNA	0	Cell Cycle - Apoptosis
mRNA	0	Driver Gene
mRNA	0	DNA Damage - Repair
mRNA	0	TGF-beta
mRNA	0	Driver Gene
mRNA	0	Cell Cycle - Apoptosis
mRNA	0	Cell Cycle - Apoptosis
mRNA	0	Cell Cycle - Apoptosis
mRNA	0	Cell Cycle - Apoptosis
mRNA	0	MAPK
mRNA	0	Driver Gene
mRNA	0	DNA Damage - Repair
mRNA	0	DNA Damage - Repair
mRNA	0	Ras
mRNA	0	MAPK
mRNA	0	MAPK
mRNA	0	MAPK
mRNA	0	DNA Damage - Repair
mRNA	0	Cell Cycle - Apoptosis
mRNA	0	DNA Damage - Repair
mRNA	0	Cell Cycle - Apoptosis
mRNA	0	Chromatin Modification
mRNA	0	Cell Cycle - Apoptosis
mRNA	0	PI3K
mRNA	0	Ras
mRNA	0	JAK-STAT
mRNA	0	Notch
mRNA	0	Transcriptional Misregulation
mRNA	0	JAK-STAT
mRNA	0	Ras
mRNA	0	Driver Gene
mRNA	0	PI3K
mRNA	0	PI3K
mRNA	0	Cell Cycle - Apoptosis
mRNA	0	DNA Damage - Repair
mRNA	0	Cell Cycle - Apoptosis
mRNA	0	DNA Damage - Repair
mRNA	0	Ras
mRNA	0	Cell Cycle - Apoptosis
mRNA	0	MAPK
mRNA	0	DNA Damage - Repair
mRNA	0	DNA Damage - Repair
mRNA	0	PI3K
mRNA	0	PI3K

mRNA	0	Cell Cycle - Apoptosis
mRNA	0	TGF-beta
mRNA	0	Transcriptional Misregulation
mRNA	0	Transcriptional Misregulation
mRNA	0	Chromatin Modification
mRNA	0	Cell Cycle - Apoptosis
mRNA	0	Cell Cycle - Apoptosis
mRNA	0	Transcriptional Misregulation
mRNA	0	Ras
mRNA	0	Cell Cycle - Apoptosis
mRNA	0	MAPK
mRNA	0	MAPK
mRNA	0	Driver Gene
mRNA	0	DNA Damage - Repair
mRNA	0	Ras
mRNA	0	JAK-STAT
mRNA	0	Wnt
mRNA	0	Chromatin Modification
mRNA	0	DNA Damage - Repair
mRNA	0	Ras
mRNA	0	MAPK
mRNA	0	Cell Cycle - Apoptosis
mRNA	0	Driver Gene
mRNA	0	Transcriptional Misregulation
mRNA	0	PI3K
mRNA	0	DNA Damage - Repair
mRNA	0	MAPK
mRNA	0	TGF-beta
mRNA	0	Transcriptional Misregulation
mRNA	0	Ras
mRNA	0	Notch
mRNA	0	Ras
mRNA	0	Cell Cycle - Apoptosis
mRNA	0	Ras
mRNA	0	Cell Cycle - Apoptosis
mRNA	0	Chromatin Modification
mRNA	0	PI3K
mRNA	0	DNA Damage - Repair
mRNA	0	Ras
mRNA	0	Cell Cycle - Apoptosis
mRNA	0	Hedgehog
mRNA	0	Driver Gene
mRNA	0	Cell Cycle - Apoptosis
mRNA	0	DNA Damage - Repair
mRNA	0	Transcriptional Misregulation
mRNA	0	Cell Cycle - Apoptosis
mRNA	0	Cell Cycle - Apoptosis
mRNA	0	Transcriptional Misregulation
mRNA	0	Cell Cycle - Apoptosis
mRNA	0	Cell Cycle - Apoptosis
mRNA	0	PI3K
mRNA	0	Transcriptional Misregulation
mRNA	0	Cell Cycle - Apoptosis
mRNA	0	Wnt
mRNA	0	Transcriptional Misregulation
mRNA	0	Ras
mRNA	0	Driver Gene
mRNA	0	Ras
mRNA	0	MAPK
mRNA	0	Chromatin Modification

mRNA	0	PI3K
mRNA	0	PI3K
mRNA	0	Hedgehog
mRNA	0	JAK-STAT
mRNA	0	Ras
mRNA	0	PI3K
mRNA	0	PI3K
mRNA	0	Hedgehog
mRNA	0	PI3K
mRNA	0	PI3K
mRNA	0	Hedgehog
mRNA	0	Ras
mRNA	0	Cell Cycle - Apoptosis
mRNA	0	Ras
mRNA	0	Ras
mRNA	0	Ras
mRNA	0	Hedgehog
mRNA	0	TGF-beta
mRNA	0	Ras
mRNA	0	PI3K
mRNA	0	Ras
mRNA	0	MAPK
mRNA	0	Wnt
mRNA	0	TGF-beta
mRNA	0	Wnt
mRNA	0	Hedgehog
mRNA	0	Notch
mRNA	0	DNA Damage - Repair
mRNA	0	Chromatin Modification
mRNA	0	Notch
mRNA	0	Cell Cycle - Apoptosis
mRNA	0	Cell Cycle - Apoptosis
mRNA	0	Cell Cycle - Apoptosis
mRNA	0	DNA Damage - Repair
mRNA	0	Wnt
mRNA	0	Cell Cycle - Apoptosis
mRNA	0	MAPK
mRNA	0	Ras
mRNA	0	DNA Damage - Repair
mRNA	0	Cell Cycle - Apoptosis
mRNA	0	PI3K
mRNA	0	Cell Cycle - Apoptosis
mRNA	0	MAPK
mRNA	0	MAPK
mRNA	0	PI3K
mRNA	0	PI3K
mRNA	0	Transcriptional Misregulation
mRNA	0	Hedgehog
mRNA	0	MAPK
mRNA	0	Driver Gene
mRNA	0	PI3K
mRNA	0	Transcriptional Misregulation
mRNA	0	Hedgehog
mRNA	0	Cell Cycle - Apoptosis
mRNA	0	Cell Cycle - Apoptosis
mRNA	0	Driver Gene
mRNA	0	Cell Cycle - Apoptosis
mRNA	0	Chromatin Modification
mRNA	0	Ras
mRNA	0	Ras

mRNA	0	PI3K
mRNA	0	Cell Cycle - Apoptosis
mRNA	0	DNA Damage - Repair
mRNA	0	Cell Cycle - Apoptosis
mRNA	0	Ras
mRNA	0	PI3K
mRNA	0	Chromatin Modification
mRNA	0	Ras
mRNA	0	MAPK
mRNA	0	Ras
mRNA	0	Transcriptional Misregulation
mRNA	0	MAPK
mRNA	0	MAPK
mRNA	0	Cell Cycle - Apoptosis
mRNA	0	MAPK
mRNA	0	Ras
mRNA	0	MAPK
mRNA	0	Ras
mRNA	0	MAPK
mRNA	0	Wnt
mRNA	0	PI3K
mRNA	0	Cell Cycle - Apoptosis
mRNA	0	Cell Cycle - Apoptosis
mRNA	0	Transcriptional Misregulation
mRNA	0	MAPK
mRNA	0	TGF-beta
mRNA	0	Cell Cycle - Apoptosis
mRNA	0	DNA Damage - Repair
mRNA	0	Cell Cycle - Apoptosis
mRNA	0	Ras
mRNA	0	Driver Gene
mRNA	0	Cell Cycle - Apoptosis
mRNA	0	PI3K
mRNA	0	Cell Cycle - Apoptosis
mRNA	0	Cell Cycle - Apoptosis
mRNA	0	Transcriptional Misregulation
mRNA	0	PI3K
mRNA	0	Cell Cycle - Apoptosis
mRNA	0	PI3K
mRNA	0	Ras
mRNA	0	DNA Damage - Repair
mRNA	0	Cell Cycle - Apoptosis
mRNA	0	Chromatin Modification
mRNA	0	JAK-STAT
mRNA	0	JAK-STAT
mRNA	0	JAK-STAT
mRNA	0	JAK-STAT
mRNA	0	Transcriptional Misregulation
mRNA	0	Ras
mRNA	0	JAK-STAT
mRNA	0	PI3K
mRNA	0	Ras
mRNA	0	Ras
mRNA	0	Driver Gene
mRNA	0	Ras
mRNA	0	MAPK
mRNA	0	Ras
mRNA	0	JAK-STAT
mRNA	0	DNA Damage - Repair
mRNA	0	Ras

mRNA	0	Notch
mRNA	0	DNA Damage - Repair
mRNA	0	PI3K
mRNA	0	JAK-STAT
mRNA	0	Ras
mRNA	0	Transcriptional Misregulation
mRNA	0	JAK-STAT
mRNA	0	Cell Cycle - Apoptosis
mRNA	0	Cell Cycle - Apoptosis
mRNA	0	PI3K
mRNA	0	JAK-STAT
mRNA	0	MAPK
mRNA	0	PI3K
mRNA	0	Cell Cycle - Apoptosis
mRNA	0	DNA Damage - Repair
mRNA	0	Wnt
mRNA	0	MAPK
mRNA	0	MAPK
mRNA	0	Ras
mRNA	0	DNA Damage - Repair
mRNA	0	DNA Damage - Repair
mRNA	0	DNA Damage - Repair
mRNA	0	TGF-beta
mRNA	0	Ras
mRNA	0	Wnt
mRNA	0	TGF-beta
mRNA	0	JAK-STAT
mRNA	0	Transcriptional Misregulation
mRNA	0	Transcriptional Misregulation
mRNA	0	JAK-STAT
mRNA	0	Transcriptional Misregulation
mRNA	0	Cell Cycle - Apoptosis
mRNA	0	Hedgehog
mRNA	0	Ras
mRNA	0	PI3K
mRNA	0	JAK-STAT
mRNA	0	Hedgehog
mRNA	0	Cell Cycle - Apoptosis
mRNA	0	JAK-STAT
mRNA	0	Transcriptional Misregulation
mRNA	0	PI3K
mRNA	0	TGF-beta
mRNA	0	Hedgehog
mRNA	0	PI3K
mRNA	0	MAPK
mRNA	0	Transcriptional Misregulation
mRNA	0	Wnt
mRNA	0	Ras
mRNA	0	Transcriptional Misregulation
mRNA	0	Transcriptional Misregulation
mRNA	0	Ras
mRNA	0	Cell Cycle - Apoptosis
mRNA	0	Ras
mRNA	0	Hedgehog
mRNA	0	Wnt
mRNA	0	Ras
mRNA	0	TGF-beta
mRNA	0	Cell Cycle - Apoptosis
mRNA	0	Ras
mRNA	0	PI3K

mRNA	0	PI3K
mRNA	0	PI3K
mRNA	0	Driver Gene
mRNA	0	DNA Damage - Repair
mRNA	0	PI3K
mRNA	0	Wnt
mRNA	0	Wnt
mRNA	0	Wnt
mRNA	0	JAK-STAT
mRNA	0	PI3K
mRNA	0	Driver Gene
mRNA	0	PI3K
mRNA	0	JAK-STAT
mRNA	0	Transcriptional Misregulation
mRNA	0	PI3K
mRNA	0	Cell Cycle - Apoptosis
mRNA	0	Cell Cycle - Apoptosis
mRNA	0	Wnt
mRNA	0	Cell Cycle - Apoptosis
mRNA	0	Transcriptional Misregulation
mRNA	0	MAPK
mRNA	0	Cell Cycle - Apoptosis
mRNA	0	Driver Gene
mRNA	0	Driver Gene
mRNA	0	Ras
mRNA	0	Driver Gene
mRNA	0	Cell Cycle - Apoptosis
mRNA	0	PI3K
mRNA	0	Transcriptional Misregulation
mRNA	0	Driver Gene
mRNA	0	Driver Gene
mRNA	0	Driver Gene
mRNA	0	Ras
mRNA	0	Ras
mRNA	0	Transcriptional Misregulation
mRNA	0	Cell Cycle - Apoptosis
mRNA	0	TGF-beta
mRNA	0	Ras
mRNA	0	JAK-STAT
mRNA	0	Ras
mRNA	0	Notch
mRNA	0	Cell Cycle - Apoptosis
mRNA	0	Driver Gene
mRNA	0	Ras
mRNA	0	Cell Cycle - Apoptosis
mRNA	0	Notch
mRNA	0	Transcriptional Misregulation
mRNA	0	MAPK
mRNA	0	Ras
mRNA	0	PI3K
mRNA	0	Transcriptional Misregulation
mRNA	0	JAK-STAT
mRNA	0	Driver Gene
mRNA	0	MAPK
mRNA	0	PI3K
mRNA	0	MAPK
mRNA	0	Cell Cycle - Apoptosis
mRNA	0	MAPK
mRNA	0	DNA Damage - Repair
mRNA	0	Wnt

mRNA	0	Transcriptional Misregulation
mRNA	0	Cell Cycle - Apoptosis
mRNA	0	Ras
mRNA	0	Transcriptional Misregulation
mRNA	0	PI3K
mRNA	0	PI3K
mRNA	0	PI3K
mRNA	0	PI3K
mRNA	0	Transcriptional Misregulation
mRNA	0	Transcriptional Misregulation
mRNA	0	Transcriptional Misregulation
mRNA	0	Cell Cycle - Apoptosis
mRNA	0	Transcriptional Misregulation
mRNA	0	PI3K
mRNA	0	JAK-STAT
mRNA	0	Cell Cycle - Apoptosis
mRNA	0	Ras
mRNA	0	PI3K
mRNA	0	Transcriptional Misregulation
mRNA	0	Ras
mRNA	0	Wnt
mRNA	0	Cell Cycle - Apoptosis
mRNA	0	PI3K
mRNA	0	MAPK
mRNA	0	Ras
mRNA	0	Ras
mRNA	0	Ras
mRNA	0	Driver Gene
mRNA	0	Ras
mRNA	0	TGF-beta
mRNA	0	JAK-STAT
mRNA	0	Ras
mRNA	0	Wnt
mRNA	0	Notch
mRNA	0	Cell Cycle - Apoptosis
mRNA	0	Transcriptional Misregulation
mRNA	0	TGF-beta
mRNA	0	MAPK
mRNA	0	Transcriptional Misregulation
mRNA	0	Ras
mRNA	0	MAPK
mRNA	0	Ras
mRNA	0	Cell Cycle - Apoptosis
mRNA	0	Cell Cycle - Apoptosis
mRNA	0	Cell Cycle - Apoptosis
mRNA	0	Wnt
mRNA	0	Cell Cycle - Apoptosis
mRNA	0	MAPK
mRNA	0	Ras
mRNA	0	Cell Cycle - Apoptosis
mRNA	0	Driver Gene
mRNA	0	MAPK
mRNA	0	Wnt
mRNA	0	Cell Cycle - Apoptosis
mRNA	0	Cell Cycle - Apoptosis
mRNA	0	PI3K
mRNA	0	Driver Gene
mRNA	0	Driver Gene
mRNA	0	TGF-beta
mRNA	0	Chromatin Modification

mRNA	0	DNA Damage - Repair
mRNA	0	DNA Damage - Repair
mRNA	0	Cell Cycle - Apoptosis
mRNA	0	Cell Cycle - Apoptosis
mRNA	0	Notch
mRNA	0	Cell Cycle - Apoptosis
mRNA	0	Cell Cycle - Apoptosis
mRNA	0	Wnt
mRNA	0	DNA Damage - Repair
mRNA	0	DNA Damage - Repair
mRNA	0	Driver Gene
mRNA	0	PI3K
mRNA	0	PI3K
mRNA	0	Ras
mRNA	0	MAPK
mRNA	0	DNA Damage - Repair
mRNA	0	PI3K
mRNA	0	Cell Cycle - Apoptosis
mRNA	0	PI3K
mRNA	0	DNA Damage - Repair
mRNA	0	Notch
mRNA	0	Ras
mRNA	0	Ras
mRNA	0	Ras
mRNA	0	Transcriptional Misregulation
mRNA	0	Ras
mRNA	0	Notch
mRNA	0	JAK-STAT
mRNA	0	PI3K
mRNA	0	Transcriptional Misregulation
mRNA	0	Cell Cycle - Apoptosis
mRNA	0	Transcriptional Misregulation
mRNA	0	Wnt
mRNA	0	Transcriptional Misregulation
mRNA	0	PI3K
mRNA	0	Hedgehog
mRNA	0	Driver Gene
mRNA	0	Cell Cycle - Apoptosis
mRNA	0	Ras
mRNA	0	JAK-STAT
mRNA	0	Ras
mRNA	0	Transcriptional Misregulation
mRNA	0	PI3K
mRNA	0	Cell Cycle - Apoptosis
mRNA	0	Driver Gene
mRNA	0	Chromatin Modification
mRNA	0	Ras
mRNA	0	Transcriptional Misregulation
mRNA	0	Driver Gene
mRNA	0	Cell Cycle - Apoptosis
mRNA	0	Cell Cycle - Apoptosis
mRNA	0	Ras
mRNA	0	Chromatin Modification
mRNA	0	MAPK
mRNA	0	Cell Cycle - Apoptosis
mRNA	0	Hedgehog
mRNA	0	DNA Damage - Repair
mRNA	0	Notch
mRNA	0	Ras
mRNA	0	Cell Cycle - Apoptosis

mRNA	0	Hedgehog
mRNA	0	Cell Cycle - Apoptosis
mRNA	0	Chromatin Modification
mRNA	0	TGF-beta
mRNA	0	Ras
mRNA	0	PI3K
mRNA	0	Notch
mRNA	0	MAPK
mRNA	0	Cell Cycle - Apoptosis
mRNA	0	PI3K
mRNA	0	PI3K
mRNA	0	MAPK
mRNA	0	Wnt
mRNA	0	Ras
mRNA	0	MAPK
mRNA	0	Wnt
mRNA	0	Ras
mRNA	0	Cell Cycle - Apoptosis
mRNA	0	DNA Damage - Repair
mRNA	0	Hedgehog
mRNA	0	Driver Gene
mRNA	0	Ras
mRNA	0	Cell Cycle - Apoptosis
mRNA	0	DNA Damage - Repair
mRNA	0	DNA Damage - Repair
mRNA	0	Cell Cycle - Apoptosis
mRNA	0	DNA Damage - Repair
mRNA	0	Cell Cycle - Apoptosis
mRNA	0	TGF-beta
mRNA	0	Ras
mRNA	0	Cell Cycle - Apoptosis
mRNA	0	MAPK
mRNA	0	PI3K
mRNA	0	Transcriptional Misregulation
mRNA	0	Wnt
mRNA	0	Cell Cycle - Apoptosis
mRNA	0	Wnt
mRNA	0	PI3K
mRNA	0	Ras
mRNA	0	Driver Gene
mRNA	0	Driver Gene
mRNA	0	JAK-STAT
mRNA	0	Notch
mRNA	0	Ras
mRNA	0	Cell Cycle - Apoptosis
mRNA	0	MAPK
mRNA	0	PI3K
mRNA	0	MAPK
mRNA	0	PI3K
mRNA	0	MAPK
mRNA	0	Cell Cycle - Apoptosis
mRNA	0	MAPK
mRNA	0	PI3K
mRNA	0	Cell Cycle - Apoptosis
mRNA	0	PI3K
mRNA	0	Ras
mRNA	0	Transcriptional Misregulation
mRNA	0	Driver Gene
mRNA	0	DNA Damage - Repair
mRNA	0	Ras

mRNA	0	Ras
mRNA	0	Notch
mRNA	0	TGF-beta
mRNA	0	TGF-beta
mRNA	0	PI3K
mRNA	0	Hedgehog
mRNA	0	Wnt
mRNA	0	TGF-beta
mRNA	0	TGF-beta
mRNA	0	PI3K
mRNA	0	Transcriptional Misregulation
mRNA	0	Driver Gene
mRNA	0	Cell Cycle - Apoptosis
mRNA	0	Driver Gene
mRNA	0	Chromatin Modification
mRNA	0	Driver Gene
mRNA	0	Driver Gene
mRNA	0	PI3K
mRNA	0	Ras
mRNA	0	Driver Gene
mRNA	0	Driver Gene
mRNA	0	Driver Gene
mRNA	0	Chromatin Modification
mRNA	0	Driver Gene
mRNA	0	Ras
mRNA	0	Cell Cycle - Apoptosis
mRNA	0	Ras
mRNA	0	TGF-beta
mRNA	0	Wnt
mRNA	0	PI3K
mRNA	0	Cell Cycle - Apoptosis
mRNA	0	Cell Cycle - Apoptosis
mRNA	0	Cell Cycle - Apoptosis
mRNA	0	Ras
mRNA	0	Cell Cycle - Apoptosis
mRNA	0	Ras
mRNA	0	Cell Cycle - Apoptosis
mRNA	0	Cell Cycle - Apoptosis
mRNA	0	Ras
mRNA	0	Cell Cycle - Apoptosis
mRNA	0	Cell Cycle - Apoptosis
mRNA	0	JAK-STAT
mRNA	0	Driver Gene
mRNA	0	Cell Cycle - Apoptosis
mRNA	0	PI3K
mRNA	0	PI3K
mRNA	0	PI3K
mRNA	0	JAK-STAT
mRNA	0	JAK-STAT
mRNA	0	MAPK
mRNA	0	Transcriptional Misregulation
mRNA	0	Driver Gene
mRNA	0	Transcriptional Misregulation
mRNA	0	PI3K
mRNA	0	Transcriptional Misregulation
mRNA	0	MAPK
mRNA	0	Ras
mRNA	0	Cell Cycle - Apoptosis
mRNA	0	Ras
mRNA	0	Chromatin Modification

mRNA	0	Cell Cycle - Apoptosis
mRNA	0	Cell Cycle - Apoptosis
mRNA	0	Cell Cycle - Apoptosis
mRNA	0	Cell Cycle - Apoptosis
mRNA	0	Driver Gene
mRNA	0	Ras
mRNA	0	Ras
mRNA	0	Cell Cycle - Apoptosis
mRNA	0	Cell Cycle - Apoptosis
mRNA	0	Driver Gene
mRNA	0	PI3K
mRNA	0	Notch
mRNA	0	DNA Damage - Repair
mRNA	0	Driver Gene
mRNA	0	Cell Cycle - Apoptosis
mRNA	0	DNA Damage - Repair
mRNA	0	Chromatin Modification
mRNA	0	DNA Damage - Repair
mRNA	0	Notch
mRNA	0	Driver Gene
mRNA	0	Ras
mRNA	0	Cell Cycle - Apoptosis
mRNA	0	Cell Cycle - Apoptosis
mRNA	0	TGF-beta
mRNA	0	Wnt
mRNA	0	Driver Gene
mRNA	0	Ras
mRNA	0	JAK-STAT
mRNA	0	Driver Gene
mRNA	0	Cell Cycle - Apoptosis
mRNA	0	Ras
mRNA	0	Notch
mRNA	0	Wnt
mRNA	0	Cell Cycle - Apoptosis
mRNA	0	Cell Cycle - Apoptosis
mRNA	0	MAPK
mRNA	0	PI3K
mRNA	0	Cell Cycle - Apoptosis
mRNA	0	TGF-beta
mRNA	0	Cell Cycle - Apoptosis
mRNA	0	Wnt
mRNA	0	Hedgehog
mRNA	0	Notch
mRNA	0	JAK-STAT
mRNA	0	Cell Cycle - Apoptosis
mRNA	0	MAPK
mRNA	0	Transcriptional Misregulation
mRNA	0	Driver Gene
mRNA	0	TGF-beta
mRNA	0	Driver Gene
mRNA	0	MAPK
mRNA	0	TGF-beta
mRNA	0	Cell Cycle - Apoptosis
mRNA	0	Ras
mRNA	0	Ras
mRNA	0	DNA Damage - Repair
mRNA	0	Cell Cycle - Apoptosis
mRNA	0	Cell Cycle - Apoptosis
mRNA	0	Ras
mRNA	0	Cell Cycle - Apoptosis

mRNA	0	Cell Cycle - Apoptosis
mRNA	0	Cell Cycle - Apoptosis
mRNA	0	Ras
mRNA	0	Driver Gene
mRNA	0	Ras
mRNA	0	Cell Cycle - Apoptosis
mRNA	0	Driver Gene
mRNA	0	MAPK
mRNA	0	Ras
mRNA	0	PI3K
mRNA	0	PI3K

Gene Name	Gene	Order selected by geNorm
**PIK3R4**	NM_014602.1:3620	1
**MRPS5**	NM_031902.3:390	2
**PIAS1**	NM_016166.1:1870	3
**SLC4A1AP**	NM_018158.2:980	4
**EIF2B4**	NM_172195.3:1390	5
**PRPF38A**	NM_032864.3:335	6
**C10orf76**	NM_024541.2:3750	7
**TMUB2**	NM_024107.2:1485	8
**DDX50**	NM_024045.1:1185	9
**TLK2**	NM_006852.2:2335	10
**VPS33B**	NM_018668.3:2140	11
**CNOT10**	NM_001256741.1:1962	12
**FCF1**	NM_015962.4:1022	13
**ZNF346**	NM_012279.2:2260	14
**SAP130**	NM_024545.3:3090	15
**AGK**	NM_018238.3:816	16
**AMMECR1L**	NM_001199140.1:3564	17
**HDAC3**	NM_003883.2:1455	18
**DHX16**	NM_001164239.1:2490	19
**MTMR14**	NM_022485.3:720	20
**COG7**	NM_153603.3:1492	21
**ZNF384**	NM_133476.3:300	22
**TTC31**	NR_027749.1:2720	23
**ERCC3**	NM_000122.1:1950	24
**ACAD9**	NM_014049.4:1935	25
**CNOT4**	NM_001190848.1:795	26
**ZC3H14**	NM_001160103.1:2690	27
**ZNF143**	NM_003442.5:925	28
**SF3A3**	NM_006802.2:2060	29
**EDC3**	NM_001142443.1:925	30
**DNAJC14**	NM_032364.5:1166	31
**CC2D1B**	NM_032449.2:4182	32
**ZKSCAN5**	NM_014569.3:3688	33
**FTSJ2**	NM_013393.1:1435	34
**USP39**	NM_001256725.1:806	35
**TRIM39**	NM_021253.3:3140	36
**GPATCH3**	NM_022078.2:1685	37
**RBM45**	NM_152945.2:1080	discarded
**NOL7**	NM_016167.3:335	discarded
**NUBP1**	NM_001278506.1:304	discarded

SD after normalization
0.192
0.285
0.281
0.279
0.266
0.302
0.294
0.294
0.253
0.328
0.346
0.337
0.354
0.322
0.353
0.386
0.418
0.386
0.395
0.452
0.473
0.45
0.39
0.48
0.463
0.478
0.463
0.51
0.51
0.607
0.543
0.535
0.563
0.576
0.607
0.611
0.623
0.683
0.704
0.836
